# From Heteroaromatic Acids and Imines to Azaspirocycles: Stereoselective Synthesis and 3D Shape Analysis

**DOI:** 10.1002/chem.201600823

**Published:** 2016-03-23

**Authors:** Sarah J. Chambers, Graeme Coulthard, William P. Unsworth, Peter O'Brien, Richard J. K. Taylor

**Affiliations:** ^1^ Department of ChemistryUniversity of YorkYorkUK

**Keywords:** 3D space, dearomatisation, lead identification, *N-*acyliminium ions, spirocycles

## Abstract

Heteroaromatic carboxylic acids have been directly coupled with imines using propylphosphonic anhydride (T3P) and NEt(*i*Pr)_2_ to form azaspirocycles via intermediate *N*‐acyliminium ions. Spirocyclic indolenines (3*H*‐indoles), azaindolenines, 2*H*‐pyrroles and 3*H*‐pyrroles were all accessed using this metal‐free approach. The reactions typically proceed with high diastereoselectivity and 3D shape analysis confirms that the products formed occupy areas of chemical space that are under‐represented in existing drugs and high throughput screening libraries.

In recent years, the biological evaluation of under‐explored regions of chemical space has attracted significant attention in the search for new pharmaceutical lead compounds. In particular, rigid, three‐dimensional scaffolds have been targeted, as they are generally poorly represented in current drugs and screening libraries.^1^ With this in mind, functionalised spirocycles are of much current interest and efficient methods to generate such compounds are of high value.^1^, ^2^

In this paper, the formation and 3D shape analysis of spirocyclic indolenines and related azaspirocycles are described. Spirocyclic indolenines (also known as 3*H*‐indoles)^3^ are important scaffolds in their own right, being present in a number of bioactive natural products, and also since they serve as precursors to other privileged heterocycles including β‐carbolines,^4^ oxindoles^5^ and indolines.^6^ The most common synthetic strategies currently used to generate spirocyclic indolenines are shown in Figure 1 A. Interrupted Fisher‐indole reactions (**1**→**4**)^7^ and intramolecular imine condensation routes (**2**→**4**)^8^ have each been well used over the years, whereas dearomatising spirocyclisation reactions (**3**→**4**)^9^ are of particular current interest^10^ and underpin the approach described herein.

**Figure 1:**
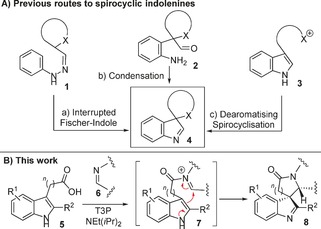
Previous and new strategies for spirocyclic indolenine synthesis.

Our new, connective method is based on the coupling of aromatic carboxylic acids **5** with imines **6** to form reactive *N*‐acyliminium ions **7** 11, 12 in situ, that can then be intercepted by intramolecular nucleophilic attack, exemplified in Figure 1 B by the formation of spirocyclic indolenines **8**.^13^ The high electrophilicity of the *N*‐acyliminium ion intermediate is a key design feature, as it means sufficiently mild conditions can be used to the allow the products to be isolated, without competing 1,2‐migration and dimerisation/trimerisation reactions taking place.^3^ Herein we report the successful implementation of this strategy, which allows indoles and other simple, electron‐rich aromatics to be converted into complex azaspirocycles, in a one‐pot, metal‐free, stereoselective process. Furthermore, 3D shape analysis,1b, 14 using the principal moments of inertia (PMI) method,^15^ shows that most of the products formed occupy interesting and under‐exploited regions of “3D chemical space”.

To explore the viability of this new approach, the reaction between 2‐methyl‐3‐indole acetic acid **5 a** and imine **6 a** was first examined (Scheme 1), by stirring these compounds in the presence of NEt(*i*Pr)_2_ and propylphosphonic anhydride (T3P) in THF at RT. Pleasingly, this led to the formation of the expected spirocycle as a mixture of diastereoisomers (**8 a**:**9 a**, 11:1), through a process that is conceptually similar to an interrupted Pictet–Spenger reaction.^16^ The diastereomeric products were partially separable by column chromatography, and isolated in 92 % overall yield (Scheme 1). The stereochemistry of the major diastereoisomer **8 a** was confirmed by X‐ray crystallography (Figure 2, see later).^17^ Following a temperature and solvent screen (see the Supporting Information), a range of other 2‐methyl indole acetic acid derivatives (**5 b**–**5 f**)^18^ were also coupled with imine **6 a** under the optimised conditions; substitution on all positions of the indole ring was examined and the desired spirocyclic indolenines were formed in good to excellent overall yield (**8/9 b**–**f**, 81–96 %). The diastereoselectivity was universally high (d.r. 6:1–13:1), with the same major diastereoisomer being formed in all cases.^19^

**Scheme 1:**
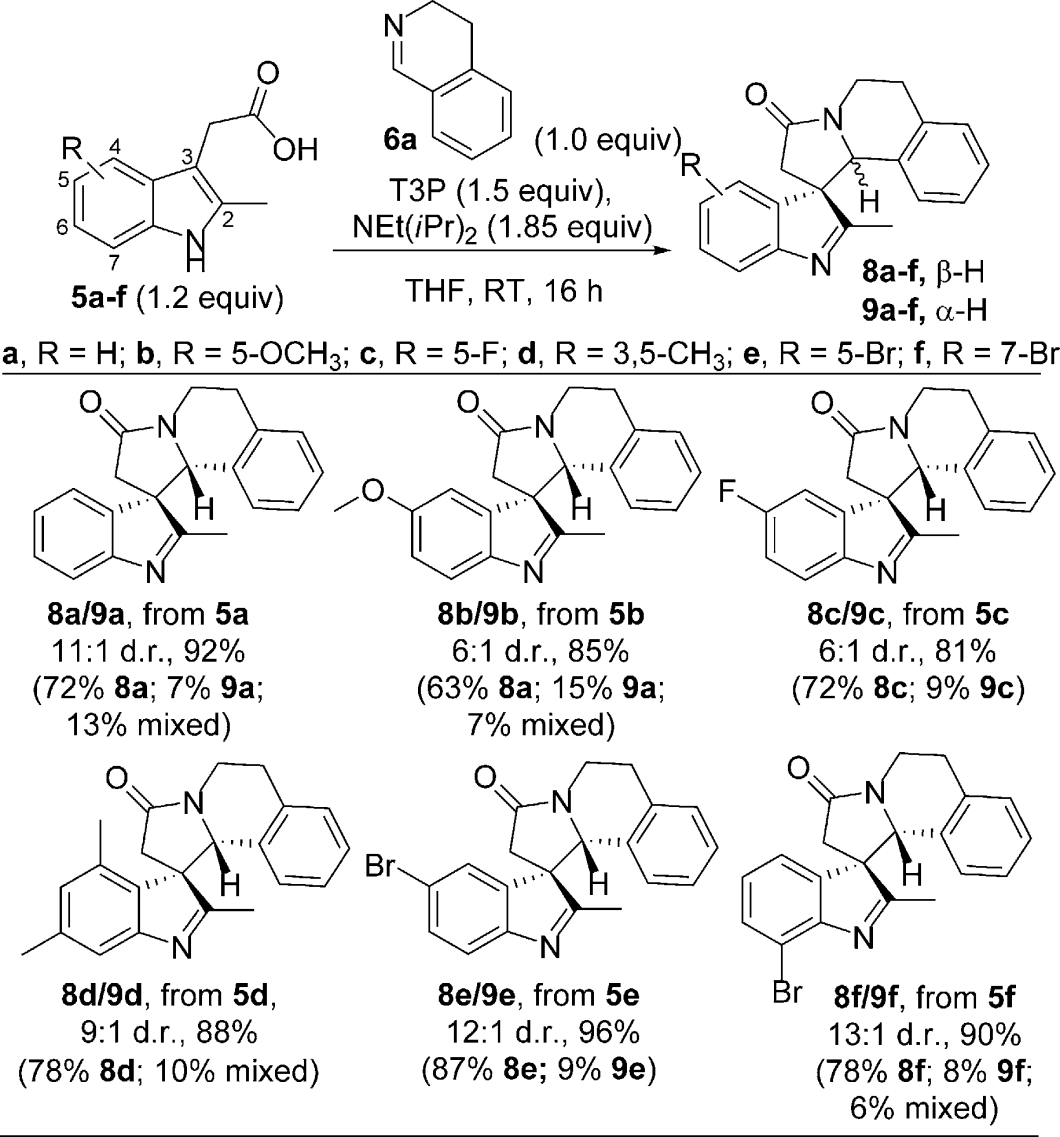
Spirocyclisation with 2‐methyl 3‐indole acetic acid derivatives. For full experimental details, see the Supporting Information; major diastereoisomer is shown, d.r. values based on ^1^H NMR analysis before chromatography; yields obtained following column chromatography.

**Figure 2:**
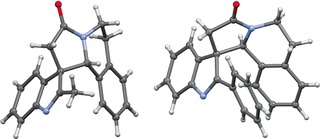
X‐ray images for spirocycles **8 a** (left) and **9 h** (right).

Indole acetic acid itself (**5 g**) was also compatible with the standard procedure, furnishing spirocycles **8 g**/**9 g** in good yield (Scheme 2), demonstrating that substitution on the indole 2‐position is not a requirement, which is pleasing given the propensity for related compounds to undergo 1,2‐migration reactions.^20^ Phenyl substitution at the 2‐position (acid **5 h**) was also well‐tolerated, with spirocycles **8 h**/**9 h** being formed in good yield, and interestingly the major product in this case was **9 h** (confirmed by X‐ray crystallography, Figure 2), which shows opposite diastereoselectivity to the previous examples.^17^ Finally, six‐membered ring spirocyclic lactams **8 i**/**9 i** were formed in good overall yield, using higher homologue **5 i**.

**Scheme 2:**
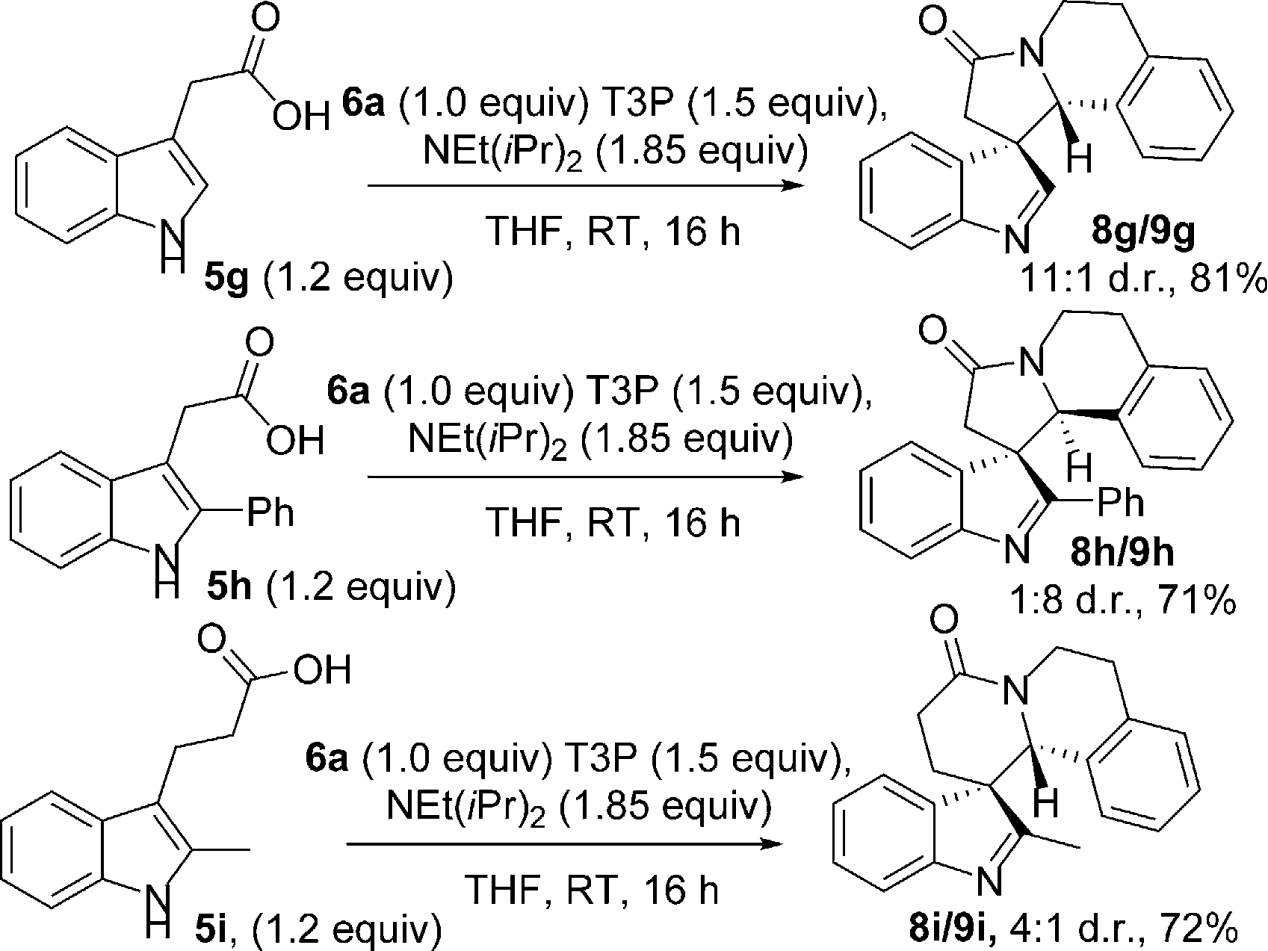
Additional acid substrates in the spirocyclisation with imine **6 a**; for full experimental details see the Supporting Information.

A plausible explanation for the observed diastereoselectivities is depicted in Figure 3, using the reaction of indole **5 a** and imine **6 a** as an example. The reaction is thought to proceed through activation of the carboxylic acid with T3P, followed by *N‐*acylation to generate a reactive *N*‐acyliminium ion **7 a**. Assuming that this is correct, the stereoselectivity is then determined by the facial selectivity of the nucleophilic attack onto the *N*‐acyliminium ion (**7 a→8 a**/**9 a**). In **A**, the benzenoid rings of the imine and indole components appear to be relatively close together in space and look well‐suited to experience a stabilising π‐stacking interaction, whereas in **B**, this interaction is absent, and replaced by a potentially destabilising steric clash between the imine and the indole 2‐methyl group. These transition‐state models also offer a plausible explanation for the switch in stereoselectivity in products **8 h**/**9 h**; in this case, as the indole 2‐position is substituted with a phenyl group rather than a methyl, a stabilising π‐stacking interaction now appears to be viable in model **B**. The reactions are believed to be under kinetic control, based on the fact that re‐subjecting a purified sample of spirocycle **8 a** to the optimised reaction conditions led to no change in the d.r., indicating that the spirocyclisation is not reversible in this case.

**Figure 3:**
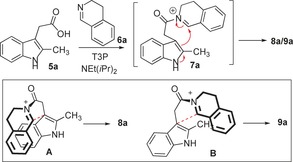
Stereochemical model for the spirocyclisation of indole acetic acid derivatives with imine **6 a**.

The scope of the reaction with respect to the imine coupling partner was examined next, with the imines used (**6 b**–**6 g**)^21^ shown in Figure 4 and spirocyclisation results in Scheme 3. Dimethoxy‐substituted imine **6 b** successfully gave the expected products **8 j** and **9 j** in moderate diastereoselectivity. Tetra‐substitution around the aromatic ring of the imine did not hinder the reaction as 2,5‐dibromo‐3,4‐dimethoxy‐substituted substrate **6 c** gave products **8 k** and **9 k** in good yield and diastereoselectivity. Thiophene‐ and pyrrole‐fused imines **6 d** and **6 e** were also suitable substrates, as was benzylated imine **6 f**, all forming the expected spirocycles **8 l**/**9 l**–**8 n**/**9 n** with generally good diastereoselectivity and in good yield. Acyclic imines, which are often avoided in related methods based on *N‐*acyliminium ion chemistry due to their tendency to hydrolyse,^22^ are also well‐tolerated, with spirocyclic products **8 o**/**9 o** and **8 p**/**9 p** each isolated in good overall yields. The major diastereoisomer formed in each case was assigned based on ^1^H NMR spectroscopy,^19^ and in the case of spirocycles **8 n** and **8 o**, confirmed by X‐ray crystallography (Scheme 3).^17^

**Figure 4:**
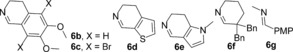
Imines **6 b**–**6 g**.

**Scheme 3:**
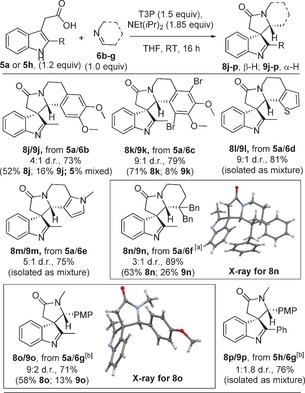
Spirocyclisation with imines **6 b**–**g**. For full experimental details, see the Supporting Information; major diastereoisomer shown, d.r. values based on ^1^H NMR analysis before chromatography; yields obtained following column chromatography. [a] Reaction performed at 90 °C in toluene for 18 h; [b] reaction performed at 70 °C in THF for 1 h.

Preliminary work also confirms that this method can be extended to other heterocyclic systems. Aza‐indoles are important structures in medicinal chemistry^23^ and pleasingly we found that aza‐indoleacetic acid **10** 18b reacted with imine **6 a** under the usual conditions to give the spirocyclic product **11** in excellent yield as a single diastereoisomer (Scheme 4), with the stereoselectivity seemingly being consistent with the analogous indole examples. Dearomatising via the 2‐position of pyrroles **12** and **13** 24 is also possible;^25^ on these systems, only a small amount of the desired product was formed when the standard conditions were used, but by switching the reaction solvent to CHCl_3_ and increasing the temperature to 70 °C, spirocycles **14** and **15** were each formed in good yield, with good to excellent diastereoselectivity. In the phenyl‐substituted case, the major diastereoisomer **15** was separable by chromatography and X‐ray crystallography was used to assign the configuration depicted.^17^, ^26^ Finally, this same modified set of conditions was used to form spirocycle **17** through the reaction of pyrrole **16** with imine **6 a**; this example is noteworthy, given that 3*H*‐pyrroles are known to be unstable and their synthesis is a considerable challenge using existing methods.^27^

**Scheme 4:**
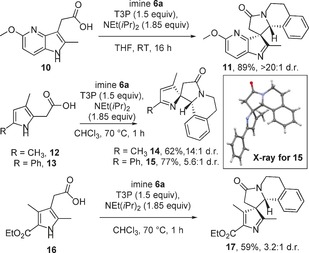
Other heterocycles in the spirocyclisation; for full experimental details see the Supporting Information.

The dearomatisation reactions described allow access to a diverse array of spirocyclic scaffolds, and the products formed are well primed to undergo further transformations, allowing additional structural diversity to be introduced or the properties of the compounds to be tuned. This is exemplified using spirocycles **8 a** and **8 g** (Scheme 5), and it is likely that similar processes (and many more) will be broadly applicable across the other spirocyclic products described in this paper. For example, indolenines **8 a** and **8 g** were both reduced to indolines **18** and **19** respectively by sodium borohydride in refluxing methanol. In the case of product **18**, the reduction was completely diastereoselective, with the hydride source approaching the indolenine from the less sterically hindered side (i.e., away from the two benzenoid rings, verified by X‐ray crystallography).^17^ Indolenines **18** and **19** could also be reduced further, forming products **20** and **21**, upon reaction with lithium aluminium hydride in refluxing THF. Products with complementary relative stereochemistry to indoline **18** could also be obtained through the addition of carbon‐based nucleophiles; products **22** and **23** were formed, again with complete diastereoselectivity, through the addition of pyrrole and methyl magnesium bromide respectively to indolenine **8 g**.^28^

**Scheme 5:**
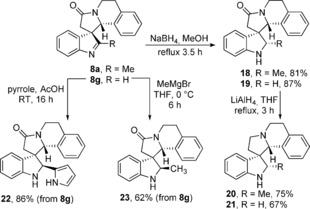
Modification of spirocycles **8 a** and **8 g**.

Finally, the principal moments of inertia (PMI) method1b, 14 was used in order to characterise the 3D shape of the azaspirocycles produced.^29^ The PMI method utilises the molecular mechanics‐generated lowest‐energy conformation and the normalized principal moments of inertia ratios, NPR1 and NPR2, are displayed on a triangular plot, with the three vertices corresponding to rod‐, disc‐ and spherical‐shaped molecules. A PMI plot containing the major diastereoisomeric forms of all of the azaspirocyclic products synthesised during the course of our study is shown in Figure 5. As this plot highlights, 88 % (22 out of 25, compounds **8 a**–**g**, **8 i**–**p**, **9 h**, **14**, **15**, **17**–**21**, **23**) of the new azaspirocycles occupy the highlighted ‘3D region’ (blue triangle) and have values of (NPR1+NPR2)>1.2. These 3D shape properties are in stark contrast to the majority of current drugs, most of which lie close to the rod‐disc axis. For example, PMI analysis of 1439 FDA‐approved small molecule drugs^30^ shows that just 23 % are found within the (NPR1+NPR2)>1.2 area (see the Supporting Information), and most drug‐screening libraries have a similar shape distribution.^31^ Hence, these results are significant, in view of the current interest in the synthesis of compounds that populate under‐explored regions of chemical space, especially spherical areas (e.g., azaspirocycles **8 b**, **8 c**, **8 e**, **8 n**, **8 p**, **9 h**, **11**), in pharmaceutical lead‐identification programs.

**Figure 5:**
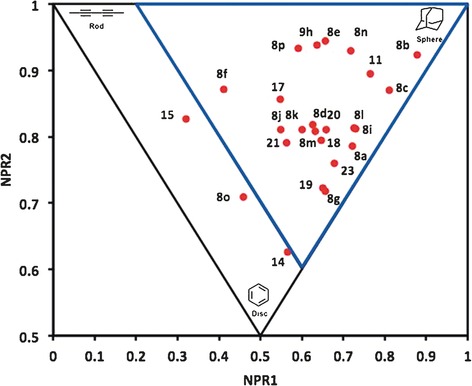
PMI analysis of spirocycles **8 a**–**g**, **8 i**–**p**, **9 h**, **14**, **15**, **17**–**21** and **23**.

In conclusion, a new, metal‐free, connective method for the synthesis of a range of 3D spirocyclic scaffolds has been reported, starting from far simpler 2D building blocks. The reactions proceeded in moderate to excellent yields and are diastereoselective, with the major diastereoisomers isolable in good overall yield in the majority of cases. This study focused predominantly on the synthesis of spirocyclic indolenines, but the successful results obtained using azaindoleacetic acid, as well as 2‐ and 3‐substituted pyrrole acetic acids, indicate that the process is much more general. 3D shape analysis indicates that a high percentage of the compounds generated in this study occupy underexplored regions of chemical space, and the ability to modify the scaffolds further has also been demonstrated, meaning that their desirable spatial and physicochemical properties can be further tuned. Future applications in the generation of medicinally relevant scaffolds/lead compounds and natural products are anticipated, and the development of asymmetric variants of these reactions will also be explored.^32^

## Supporting information

As a service to our authors and readers, this journal provides supporting information supplied by the authors. Such materials are peer reviewed and may be re‐organized for online delivery, but are not copy‐edited or typeset. Technical support issues arising from supporting information (other than missing files) should be addressed to the authors.

Supplementary
