# Supplementary material for: From Heteroaromatic Acids and Imines to Azaspirocycles: Stereoselective Synthesis and 3D Shape Analysis
Source: Chemistry. 2016 Mar 23;22(19):6496–500. doi: 10.1002/chem.201600823 (PMC5071705; doi:10.1002/chem.201600823)
Supplement: Supplementary file 1 — Supplementary [file CHEM-22-6496-s001.pdf]

CHEMISTRY
A European Journal

Supporting Information

From Heteroaromatic Acids and Imines to Azaspirocycles: Stereoselective Synthesis and 3D Shape Analysis

Sarah J. Chambers, Graeme Coulthard, William P. Unsworth, Peter O'Brien, and Richard J. K. Taylor*[a]

chem_201600823_sm_miscellaneous_information.pdf

Supporting Information

Table of Contents | **Page**

Table of Contents	Page
(1) General information	S2
(2) Literature procedures for acid and imine substrates	S2
(3) General procedures	S3
(4) Solvent and temperature screen of spirocyclisation reaction	S4
(5) Synthesis of acid substrates	S5
(6) Synthesis of imine substrates	S18
(7) Acid scope in the DIA spirocyclisation reaction	S22
(8) Imine scope in the DIA spirocyclisation reaction	S31
(9) Other heterocycle scope in the DIA spirocyclisation reaction	S40
(10) Procedures for the modification of spirocycles **8a** and **8g**	S43
(11) ^{1}H and ^{13}C NMR spectra	S49
(12) 3-Dimensional shape analysis of spirocyclic products and FDA approved drugs	S97
(13) References	S99

(1) General Information

Except where stated, all reagents were purchased from commercial sources and used without further purification. Except where stated, all experimental procedures were carried out under an atmosphere of argon. Anhydrous CH_2Cl_2 and toluene were obtained from an Innovative Inc. PureSolv® solvent purification system. Anhydrous THF was obtained by distillation over sodium benzophenone ketyl immediately before use. Chloroform was used as supplied without additional drying. ^{1}H NMR and ^{13}C NMR spectra were recorded on a JEOL ECX400 or JEOL ECS400 spectrometer, operating at 400 MHz and 100 MHz, respectively. All spectral data were acquired at 295 K. Chemical shifts (δ) are quoted in parts per million (ppm). The residual solvent peaks, δ_H 7.26 and δ_C 77.0 for $CDCl_3$ and δ_H 2.50 and δ_C 39.5 for DMSO-d6 were used as a reference. Coupling constants (*J*) are reported in Hertz (Hz) to the nearest 0.5 Hz. The multiplicity abbreviations used are: s singlet, d doublet, t triplet, q quartet, m multiplet. Signal assignment was achieved by analysis of DEPT, COSY, NOESY, HMBC and HSQC experiments where required. Infrared (IR) spectra were recorded on a PerkinElmer UATR two spectrometer as a thin film. Mass-spectra (low and high-resolution) were obtained by the University of York Mass Spectrometry Servive, using electrospray ionisation (ESI) on a Bruker Daltonics, Micro-TOF spectrometer. The melting points were determined using Gallenkamp apparatus and are uncorrected. Thin layer chromatography was carried out on Merck silica gel $60F_{254}$ pre-coated aluminium foil sheets and were visualised using UV light (254 nm) and stained with either basic aqueous potassium permanganate or ethanolic *p*-anisaldehyde as appropriate. Flash column chromatography was carried out using slurry packed silica gel (SiO_2), 35-75 µm particle size, 60 Å pore size, under a light positive pressure, eluting with the specified solvent system. Petrol refers to petroleum ether 40–60 °C. Microwave experiments were carried out using sealed vessels in a CEM Discover microwave reactor with variable power output (0-200 W). Pressure was recorded by the cavity lid, which resisted the deformation of the vial cap as pressure increased.

(2) Literature procedures for the preparation of indole acid and imine substrates

Acids **5a** and **5g** are commercially available and were used as supplied. Acids **5c**[1] and **5h**[2] and imines **6a**,[3] **6c**,[4] **6f**,[5] and **6g**[6] were prepared by the literature methods cited.

(3) General procedures

General Procedure A: Spirocycle synthesis from *N*-acyliminium ions

DIPEA (1.85 eq, 245 µL, 1.41 mmol) and T3P (1.5 eq, 725 mg of a 50% w/v solution in THF, 1.14 mmol) were added to a stirred solution of imine (1 eq, 0.762 mmol) and acid (1.2 eq, 0.909 mmol) in solvent (4 mL) at a specified temperature. The resulting solution was stirred in a sealed vessel for a specified time. The solution was diluted with CH_2Cl_2 (10 mL) and poured into sat. $NaHCO_{3(aq)}$ (10 mL). The two layers were separated and the aqueous layer was extracted with CH_2Cl_2 (3 x 10 mL). The combined organics were dried ($MgSO_4$) and evaporated under reduced pressure to give the crude product.

General Procedure B: $NaBH_4$ reduction of spirocyclic indolenine

$NaBH_4$ (4 eq, 1.32 mmol) was added portionwise (CARE: effervescence) to a stirred solution of imine (1 eq, 0.33 mmol) in MeOH (5 mL) at 0 °C under Ar. The reaction mixture was heated to reflux and stirred for 3.5 h and then allowed to cool to rt. The solvent was evaporated under reduced pressure and the resulting residue taken up in H_2O (10 mL) and CH_2Cl_2 (10 mL). The two layers were separated and the aqueous layer was extracted with CH_2Cl_2 (3 x 10 mL). The combined organics were dried ($MgSO_4$) and evaporated under reduced pressure to give the crude product.

General Procedure C: $LiAlH_4$ reduction of spirocyclic indoline

$LiAlH_4$ (4 eq, 2.00 mmol) was added to a stirred solution of amide (1 eq, 0.50 mmol) in dry THF (15 mL) at 0 °C under Ar. The resulting suspension was heated to reflux and stirred for 2.5 h, then the reaction was allowed to cool to rt. The reaction was diluted with EtOAc (5 mL) and H_2O was carefully added until effervescence ceased. $NaSO_4$ was added and the resulting suspension stirred for 30 min, then the mixture was filtered and the solvent evaporated under reduced pressure to give the crude product.

(4) Solvent and temperature screen of spirocyclisation reaction

General procedure A was followed, using imine **6a** (50 mg, 0.381 mmol) and acid **5a** (87 mg, 0.457 mmol) in the specified solvent (2 mL) for 16 h at a specified temperature. The diastereomeric ratio was determined by analysis of the unpurified reaction by ^{1}H NMR spectroscopy. Purification by flash column chromatography using EtOAc as eluent provided **8a** and **9a** to give a combined yield (Table 1).

Table 1: Solvent screen conditions and results

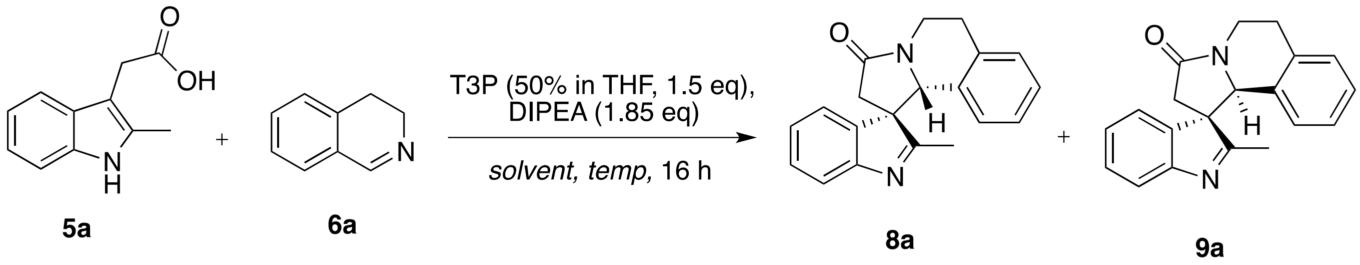

Entry	Solvent	Temp	d.r.	Yield (%)
1	$CHCl_3$	RT	6:1	95
2	CH_2Cl_2	RT	7.5:1	91
3	Toluene	RT	7:1	79
4	MeCN	RT	5.5:1	61
5	THF	RT	9:1	82
6	2-MeTHF	RT	7.8:1	74
7	TBME	RT	1:1.1	42
8	Et_2O	RT	1:1.4	40
9	THF	0 °C[a]	10.3:1	44
10	THF	40 °C	8.5:1	91
11	THF	60 °C	7:1	82

[a] Reaction run for 3 hours.

(5) Synthesis of the acid substrates

(5-Methoxy-2-methyl-1*H*-indol-3-yl)acetic acid (5b)

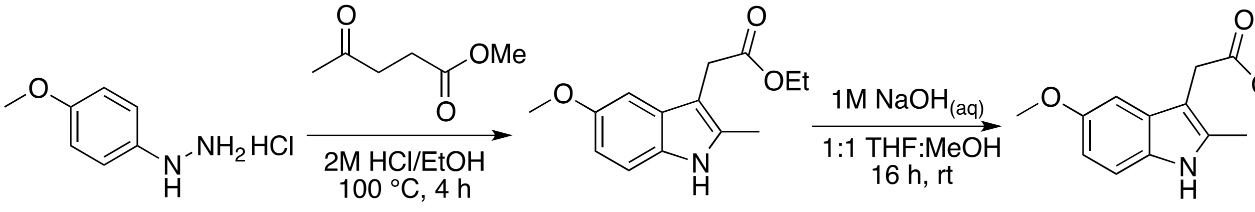

SI-1 **5b**

The following procedure was adapted from a literature protol.[7] A mixture of methyl 4-oxopentanoate (354 μL, 2.86 mmol) and 4-methoxyphenylhydrazine hydrochloride (500 mg, 2.86 mmol) in 2 M HCl/EtOH (20 mL) was stirred at 100 °C for 4 h. After cooling to room temperature the solvent was concentrated until a solid precipitate was observed. The precipitate was removed by filtration and the filtrate concentrated under reduced pressure. The residue was dissolved in CH_2Cl_2 (50 mL) and washed with H_2O (50 mL). The aqueous layer was extracted with CH_2Cl_2 (4 × 50 mL). The combined organics were dried ($MgSO_4$) and concentrated under reduced pressure. Purification by flash column chromatography on silica with hexane-acetone (9:1 then 8:2) as eluent gave indole ethyl ester **SI-1** (404 mg, 57%) as a brown oil. R_f (8:2 hexane/acetone) 0.10; ν_{max} (thin film)/cm^{-1} 3397 (NH), 2982, 1724 (C=O), 1627, 1592, 1485, 1216, 1174, 1031, 799; δ_H (400 MHz, $CDCl_3$) 7.74 (s, 1H), 7.15 (d, *J* = 8.5 Hz, 1H), 7.00 (d, *J* = 2.5 Hz, 1H), 6.77 (dd, *J* = 8.5, 2.5 Hz, 1H), 4.13 (q, *J* = 7.0 Hz, 2H), 3.86 (s, 3H), 3.64 (s, 2H), 2.40 (s, 3H), 1.24 (t, *J* = 7.0 Hz, 3H); δ_C (100 MHz, $CDCl_3$) 172.0 (C=O), 154.1 (C), 133.4 (C), 130.1 (C), 129.0 (C), 111.0 (CH), 110.9 (CH), 104.6 (C), 100.5 (CH), 60.6 (CH_2), 55.9 (CH_3), 30.6 (CH_2), 14.3 (CH_3), 11.8 (CH_3); HRMS (ESI) *m/z* calcd for $C_{14}H_{17}NO_3$ $(M + H)^+$ 248.1281, found 248.1274 (+3.0 ppm error). These data were consistent with the literature values.[8]

1 M NaOH (10 mL) was added to a stirred solution of ethyl 2-(5-methoxy-2-methyl-1*H*-indol-3-yl)acetate **SI-1** (395 mg, 1.60 mmol) in 1:1 THF/MeOH (40 mL). The resulting solution was stirred at room temperature for 16 h. The reaction mixture was concentrated under reduced pressure and the resulting solid residue dissolved in water (5 mL). The solution was acidified (~pH 1) using conc. HCl resulting in the formation of an oily residue. CH_2Cl_2 (30 mL) and 1 M HCl (40 mL) were added and the two layers were separated. The aqueous layer was extracted with CH_2Cl_2 (2 × 30 mL) and the combined organics were dried ($MgSO_4$) and concentrated under reduced pressure to give the indole acetic acid **5b** (331 mg, 95%) as a green/brown solid, mp 141–143 °C (lit.[9] 162 °C (from EtOH)); ν_{max} (thin film)/cm^{-1} 3353

(NH), 3104 (OH), 2903, 1723 (C=O), 1589, 1485, 1309, 1209, 1167, 1016, 804, 647; δ_H (DMSO-d_6) 12.03 (s, 1H), 10.65 (s, 1H), 7.12 (d, *J* = 8.5 Hz, 1H), 6.88 (d, *J* = 2.5 Hz, 1H), 6.62 (dd, *J* = 8.5, 2.5, 1H), 3.72 (s, 3H) 3.52 (s, 2H), 2.29 (s, 3H); δ_C (100 MHz, DMSO-d_6); 173.3 (C=O), 153.0 (C), 133.7 (C), 130.1 (C), 128.7 (C), 110.9 (CH), 109.5 (CH), 103.8 (C), 100.1 (CH), 55.3 (OCH_3), 29.9 (CH_2), 11.4 (CH_3); HRMS (ESI) *m/z* calcd for $C_{12}H_{13}NO_3$ $(M + H)^+$ 220.0968, found 220.0967 (+0.6 ppm error). These data are consistent with the literature values.[9]

(5-Fluoro-2-methyl-1*H*-indol-3-yl)acetic acid (5c)

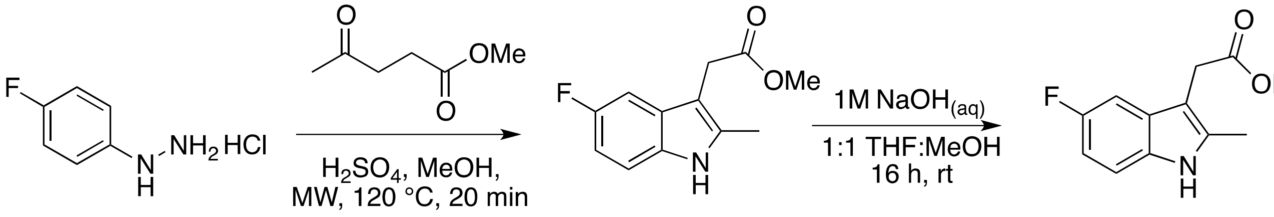

SI-2 **5c**

SI-2 was synthesised following a literature procedure.[1] A mixture of methyl 4-oxopentanoate (136 μL, 1.10 mmol) and 4-fluorophenylhydrazine hydrochloride (163 mg, 1.00 mmol) in MeOH (3 mL) and conc. H_2SO_4 (120 μL) was stirred for 10 min at 120 °C in a microwave synthesiser. After being allowed to cool to room temperature, H_2O (10 mL) was added and the reaction was extracted with CH_2Cl_2 (3 × 10 mL). The combined organics were dried ($MgSO_4$) and concentrated under reduced pressure. Purification by flash column chromatography on silica using CH_2Cl_2 as eluent gave the substituted indole **SI-2** (194 mg, 87%) as a pale yellow oil, R_f (CH_2Cl_2) 0.30; δ_H (400 MHz, DMSO-d_6) 10.99 (s, 1H), 7.22 (dd, *J* = 9.0, 4.5 Hz, 1H), 7.11 (dd, *J* = 10.0, 2.5 Hz, 1H), 6.82 (ddd, *J* = 9.0, 9.0, 2.5 Hz, 1H), 3.66 (s, 2H), 3.58 (s, 3H), 2.32 (s, 3H); δ_C (100 MHz, DMSO-d_6) 171.9 (C=O), 156.8 (d, *J* = 230.5 Hz, C), 135.6 (C), 131.6 (C), 128.6 (d, *J* = 10.0 Hz, C), 111.2 (d, *J* = 10.0 Hz, CH), 107.9 (d, *J* = 25.5 Hz, CH), 103.7 (d, *J* = 4.5 Hz, C), 102.4 (d, *J* = 23.5 Hz, CH), 51.5 (OCH_3), 29.8 (CH_2), 11.4 (CH_3); ^{19}F NMR (376 MHz, d6-DMSO) δ −125.50 (ddd, *J* = 10.0, 10.0, 4.5 Hz); HRMS (ESI) *m/z* calcd for $C_{12}H_{12}FNO_2$ $(M + H)^+$ 222.0925, found 222.0932 (−3.3 ppm error). These data are consistent with the literature values.[1]

1 M $NaOH_{(aq)}$ (10 mL) was added to a stirred solution of methyl 2-(5-fluoro-2-methyl-1*H*-indol-3-yl)acetate **SI-2** (448 mg, 2.03 mmol) in 1:1 THF/MeOH (40 mL). The resulting solution was stirred at room temperature for 16 h. The reaction mixture was concentrated under reduced pressure and the resulting solid residue dissolved in water (5 mL). Conc. HCl was added dropwise until a solid precipitate was observed. The solid was isolated by vacuum filtration, washed with water (5 mL) and hexane (5 mL)

and air dried to give the indole acetic acid **5c** (369 mg, 87%) as a pale orange solid, mp 164–166 °C [lit.[10] 179–182 °C (from MeCN)]; ν_{max} (thin film)/cm^{-1} 3411 (NH), 2908 (OH), 2736, 2631, 1715 (C=O), 1587, 1481, 1241, 920, 840, 605; δ_H (400 MHz, DMSO-*d*$_6$) 12.11 (s, 1H), 10.94 (s, 1H), 7.22 (dd, *J* = 9.0, 4.5 Hz, 1H), 7.12 (dd, *J* = 10.0, 2.5 Hz, 1H), 6.81 (ddd, *J* = 9.0, 9.0, 2.5, 1H), 3.54 (s, 2H), 2.31 (s, 3H); δ_C (100 MHz, DMSO-*d*$_6$) 173.1 (C=O), 156.8 (d, *J* = 230.5 Hz, C), 135.3 (C), 131.6 (C), 128.7 (d, *J* = 10.0 Hz, C), 111.1 (d, *J* = 10.0 Hz, CH), 107.8 (d, *J* = 26.0 Hz, CH), 104.4 (d, *J* = 4.5 Hz, C), 102.5 (d, *J* = 23.0 Hz, CH), 29.8 (CH_2), 11.4 (CH_3); ^{19}F NMR (376 MHz, $CDCl_3$) δ −125.70 (ddd, *J* = 10.0, 10.0, 4.5 Hz); HRMS (ESI) *m/z* calcd for $C_{11}H_{10}FNO_2$ $(M + H)^+$ 208.0768, found 208.0771 (−1.4 ppm error).

(2,5,7-Trimethyl-1*H*-indol-3-yl))acetic acid (5d)

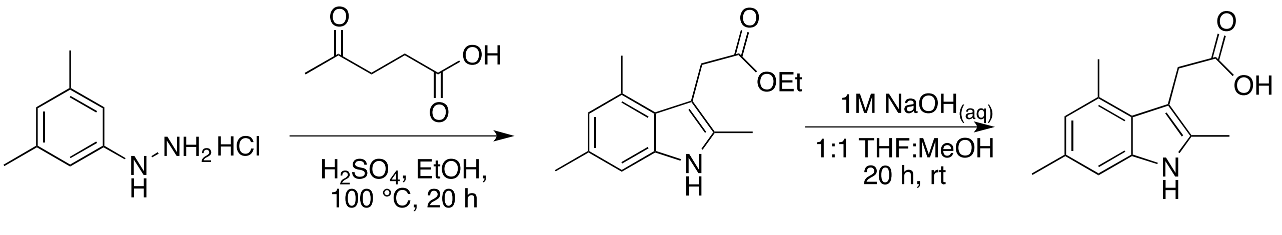

SI-3 **5d**

The following procedure was adapted from a literature protol.[11,12] A mixture of 4-oxopentanoic acid (0.59 mL, 5.79 mmol) and 3,5-dimethylphenylhydrazine hydrochloride (1.00 g, 5.79 mmol) in 2M H_2SO_4/EtOH (40 mL) was stirred at 100 °C for 20 h. The reaction mixture was allowed to cool to rt and the solvent was evaporated under reduced pressure. The resulting residue was diluted with water (20 mL) and extracted into EtOAc (50 mL). The two layers were separated and the aqueous extracted with EtOAc (2 x 50 mL). The combined organics were washed with sat. $NaHCO_{3\,(aq)}$ (10 mL), then dried ($MgSO_4$) and the solvent evaporated under reduced pressure to give the crude product. Purification by flash column chromatography on silica using CH_2Cl_2 as eluent gave the indole ester **SI-3** (1.035 g, 72%) as a pale yellow solid, mp 84–86 °C (lit.[13] 101–102 °C); R_f (CH_2Cl_2) 0.33; ν_{max} (thin film)/cm^{-1} 3353 (NH), 2978, 2909, 1710 (C=O), 1624, 1467, 1442, 1330, 1302, 1233, 1157, 1027, 828; δ_H (400 MHz, $CDCl_3$) 7.75 (s, 1H), 6.85 (d, *J* = 1.0 Hz, 1H), 6.67 (s, 1H), 4.14 (q, *J* = 7.0 Hz, 2H), 3.81 (s, 2H), 2.64 (s, 3H), 2.39 (s, 3H), 2.33 (s, 3H), 1.25 (t, *J* = 7.0 Hz, 3H); δ_C (100.6 MHz, $CDCl_3$) 172.7 (C=O), 135.7 (C), 132.0 (C), 130.7 (C), 129.1 (C), 124.6 (C), 123.1 (CH), 108.3 (CH), 104.7 (C), 60.6 (CH_2), 31.7 (CH_2), 21.3 (CH_3), 19.8 (CH_3), 14.2 (CH_3), 11.6 (CH_3); HRMS (ESI) *m/z* calcd for $C_{15}H_{19}NO_2$ $(M + H)^+$ 246.1489, found 246.1494 (−2.1 ppm error).

1 M $NaOH_{(aq)}$ (20 mL) was added to a stirred solution of the dimethyl-indole ester **SI-3** (930 mg, 3.79 mmol) in 1:1 MeOH-THF (80 mL) at rt. The resulting solution was stirred at rt for 20 h and then the solvent was evaporated under reduced pressure to give the crude product. The residue was taken up in a minimum volume of water (5 mL) and conc. $HCl_{(aq)}$ was added dropwise until a solid precipitate was observed. The solid was isolated by vacuum filtration, washed with water (5 mL) and hexane (5 mL) and air dried to give the indole acetic acid **5d** (651 mg, 79%) as a pale red solid, mp 190–192 °C (lit.[13] 203.5–204.5 °C); ν_{max} (thin film)/cm^{-1} 3398 (NH), 2918, 1706 (C=O), 1622, 1561, 1467, 1436, 1400, 1215, 843; δ_H (400 MHz, DMSO-d_6) 10.62 (s, 1H), 6.85 (s, 1H), 6.47 (s, 1H), 3.65 (s, 2H), 2.49 (s, 3H), 2.29 (s, 3H), 2.26 (s, 3H); δ_C (100.6 MHz, $CDCl_3$) 174.1 (C=O), 135.6 (C), 132.2 (C), 128.7 (C), 128.0 (C), 124.6 (C), 121.9 (CH), 108.4 (CH), 104.2 (C), 31.2 (CH_2), 21.2 (CH_3), 19.5 (CH_3), 11.2 (CH_3); HRMS (ESI) *m/z* calcd for $C_{13}H_{15}NO_2$ $(M + H)^+$ 218.1176, found 218.1175 (+0.4 ppm error).

(5-Bromo-2-methyl-1*H*-indol-3-yl)acetic acid (5e)

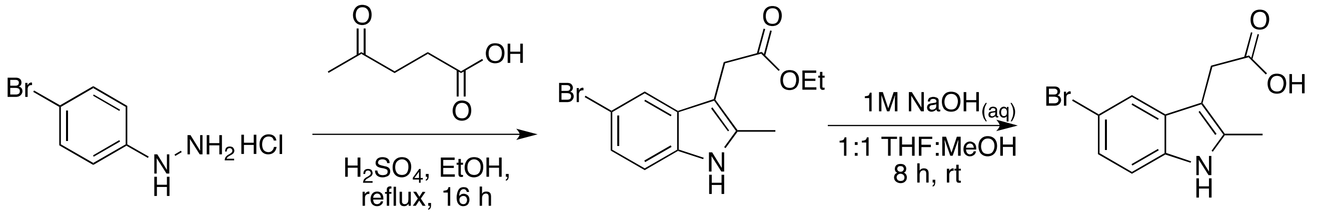

SI-4 **5e** \

The following procedure was adapted from a literature protol.[14] A mixture of 4-oxopentanoic acid (0.46 mL, 4.47 mmol) and 4-bromophenylhydrazine hydrochloride (1.00 g, 4.47 mmol) in a solution of EtOH (6 mL) and conc. $H_2SO_{4\,(aq)}$ (0.45 mL) was stirred at reflux for 16 h. The reaction mixture was allowed to cool to room temperature, then water (20 mL) and EtOAc (20 mL) was added. The two layers were separated and the aqueous layer was extracted with EtOAc (1 × 10 mL). The combined organic phases were washed with 10% HCl (10 mL) and then sat. $NaHCO_3$ (10 mL), dried ($MgSO_4$) and concentrated under reduced pressure. Purification by flash column chromatography on silica using CH_2Cl_2 as eluent gave the indole ester **SI-4** (834 mg, 63%) as an orange solid, mp 59–61 °C [lit.[15] 65–68 °C]; R_f (CH_2Cl_2) 0.30; ν_{max} (thin film)/cm^{-1} 3355 (NH), 2981, 2901, 1716 (C=O), 1578, 1469, 1434, 1368, 1304, 1260, 1162, 1030, 793; δ_H (400 MHz, $CDCl_3$) 7.98 (s, 1H), 7.64 (d, J = 2.0 Hz, 1H), 7.17 (dd, J = 8.5, 2.0 Hz, 1H), 7.07 (d, J = 8.5 Hz, 1H), 4.15 (q, J = 7.0 Hz, 2H), 3.62 (s, 2H), 2.35 (s, 3H), 1.26 (t, J = 7.0 Hz, 3H); δ_C (100 MHz, $CDCl_3$) 171.8 (C=O), 134.1 (C), 133.7 (C), 130.2 (C), 123.9 (CH), 120.7 (CH), 112.7 (C), 111.6 (CH), 104.3 (C), 60.8 (CH_2), 30.2 (CH_2), 14.2 (CH_2), 11.7 (CH_3); HRMS (ESI) *m/z* calcd for $C_{13}H_{14}{}^{79}BrNO_2$ $(M + H)^+$ 296.0281, found 296.0279 (+0.7 ppm error). These data are consistent with the literature values.[16]

1 M $NaOH_{(aq)}$ (10 mL) was added to a stirred solution of bromo-substituted indole ester **SI-4** (622 mg, 2.10 mmol) in 1:1 THF/MeOH (42 mL). The resulting solution was stirred at room temperature for 8 h. The reaction mixture was concentrated under reduced pressure and the residue dissolved in water (5 mL). The solution was made acidic (~pH 1) with conc. HCl resulting in the formation of a gummy precipitate. The resulting mixture was extracted into EtOAc (30 mL) and the two layers separated. The aqueous was extracted into EtOAc (2 x 20 mL) and the combined organics dried ($MgSO_4$) and the solvent evaporated under reduced pressure to give the indole acid **5e** (513 mg, 91%) as a brown solid, mp 159–161 °C [lit.,[10] 189–191 °C]; δ_H (400 MHz, DMSO-d_6) 12.12 (br s, 1H), 11.08 (s, 1H), 7.54 (d, J = 2.0 Hz, 1H), 7.21 (d, J = 8.5 Hz, 1H), 7.09 (dd, J = 8.5, 2.0 Hz, 1H), 3.56 (s, 2H), 2.32 (s, 3H); δ_C (100 MHz, DMSO-d_6) 173.0 (C=O), 135.0 (C), 133.7 (C), 130.2 (C), 122.4 (CH), 120.0 (CH), 112.4 (CH), 111.0 (C), 103.9 (C), 29.7 (CH_2), 11.3 (CH_3); HRMS (ESI) *m/z* calcd for $C_{11}H_{10}{}^{79}BrNO_2$ $(M + H)^+$ 267.9968, found 267.9982 (+0.7 ppm error). These data are consistent with the literature values.[17]

(7-Bromo-2-methyl-1*H*-indol-3-yl)acetic acid (5f)

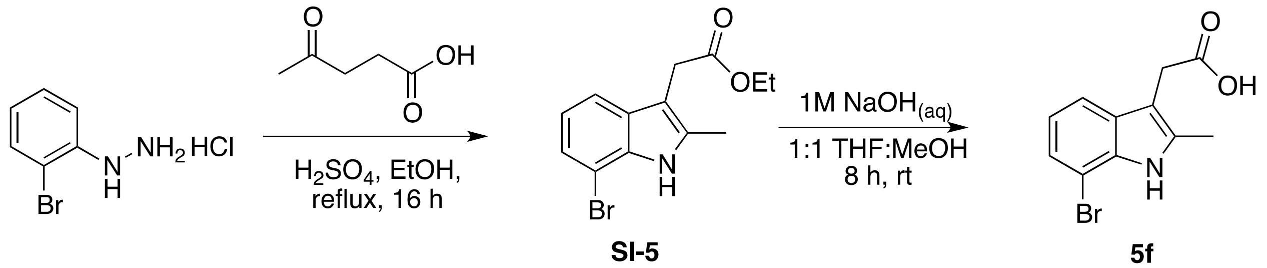

The following procedure was adapted from a literature protol.[18] A mixture of 4-oxopentanoic acid (0.46 mL, 4.47 mmol) and 2-bromophenylhydrazine hydrochloride (1.0 g, 4.47 mmol) in a solution of EtOH (6 mL) and conc. $H_2SO_{4\,(aq)}$ (0.45 mL) was stirred at reflux for 16 h. The reaction mixture was allowed to cool to rt and water (20 mL) was added. The resulting mixture was extracted into EtOAc (20 mL), the two layers separated and the aqueous extracted with EtOAc (10 mL). The combined organics were washed with 10% $HCl_{(aq)}$ (10 mL) and sat. $NaHCO_{3\,(aq)}$ (10 mL), then dried ($MgSO_4$) and the solvent evaporated under reduced pressure to give the crude product. Purification by flash column chromatography on silica using CH_2Cl_2 as eluent gave the indole ester **SI-5** (550 mg, 42%) as an orange solid, mp 64–66 °C; R_f (CH_2Cl_2) 0.44; ν_{max} (thin film)/cm^{-1} 3350 (NH), 2980, 2912, 1718 (C=O), 1623, 1583, 1491, 1446, 1368, 1304, 1296, 1151, 1030, 772; δ_H (400 MHz, $CDCl_3$) 8.05 (s, 1H), 7.47 (d, J = 8.0 Hz, 1H), 7.27–7.25 (m, 1H), 6.98 (dd, J = 8.0, 8.0 Hz, 1H), 4.13 (q, J = 7.0 Hz, 2H), 3.66 (s, 2H), 2.44 (s, 3H), 1.24 (t, J = 7.0 Hz, 3H); δ_C (100.6 MHz, $CDCl_3$) 171.7 (C=O), 133.7 (C), 133.5 (C), 129.6 (C), 123.5 (CH), 120.7 (CH),

117.4 (CH), 106.0 (C), 103.9 (C), 60.8 (CH_2), 30.6 (CH_2), 14.2 (CH_3), 11.8 (CH_3); HRMS (ESI) *m/z* calcd for $C_{13}H_{14}{}^{79}BrNO_2$ (M + H)$^+$ 296.0281, found 296.0275 (+1.8 ppm error). These data are consistent with the literature values.[19]

1 M $NaOH_{(aq)}$ (8 mL) was added to a stirred solution of the bromo-substituted indole ester **SI-5** (459 mg, 1.55 mmol) in 1:1 MeOH-THF (30 mL) at rt. The resulting solution was stirred at rt for 8 h and then the solvent was evaporated under reduced pressure to give the crude product. The residue was taken up in a minimum amount of water (5 mL) and the resulting solution was taken to pH 1 with conc. $HCl_{(aq)}$ resulting in an oily mixture. The resulting mixture was extracted into CH_2Cl_2 (50 mL) and the two layers separated. The aqueous was extracted into CH_2Cl_2 (2 x 50 mL) and the combined organics dried ($MgSO_4$) and the solvent evaporated under reduced pressure to give the indole acid **5f** (360 mg, 86%) as a brown solid, mp 128–131 °C (lit.[20] 160–161 °C); ν_{max} (thin film)/cm^{-1} 3380 (NH), 2902, 2634, 1690 (C=O), 1625, 1590, 1558, 1456, 1401, 1225, 1191, 768; δ_H (400 MHz, DMSO-d_6) 12.13 (br s, 1H), 11.02 (s, 1H), 7.39 (d, J = 8.0 Hz, 1H), 7.21–7.19 (m, 1H), 6.89 (dd, J = 8.0, 8.0 Hz, 1H), 3.57 (s, 2H), 2.35 (s, 3H); δ_C (100.6 MHz, DMSO-d_6) 173.0 (C=O), 135.0 (C), 133.4 (C), 130.0 (C), 122.6 (CH), 119.8 (CH), 117.1 (CH), 105.5 (C), 103.3 (C), 29.9 (CH_2), 11.24 (CH_3); HRMS (ESI) *m/z* calcd for $C_{11}H_{10}{}^{79}BrNO_2$ (M + H)$^+$ 267.9968, found 267.9974 (−2.4 ppm error).

3-(2-Methyl-1*H*-indol-3-yl)propanoic acid (5i)

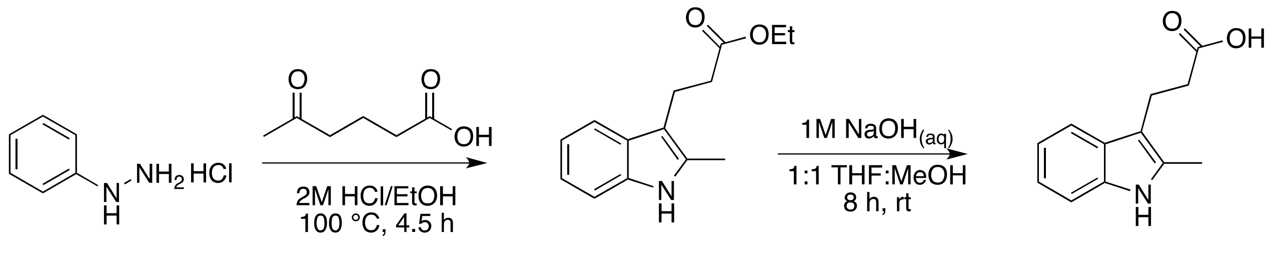

The following procedure was adapted from a literature protol.[7] A mixture of 5-oxohexanoic acid (0.83 mL, 6.92 mmol) and phenylhydrazine hydrochloride (1.0 g, 6.92 mmol) in a 2M HCl/EtOH solution (6 mL) was stirred at 100 °C for 4.5 h. The reaction mixture was allowed to cool to rt and the solvent was evaporated under reduced pressure. The resulting mixture was diluted with water (200 mL) and extracted into EtOAc (100 mL). The two layers were separated and the aqueous extracted with EtOAc (2 x 100 mL). The combined organics were dried ($MgSO_4$) and the solvent evaporated under reduced pressure to give the crude product. Purification by flash column chromatography on silica using CH_2Cl_2 as eluent gave the indole ester **SI-6** (948 mg, 59%) as an orange oil, R$_f$ (CH_2Cl_2) 0.30; ν_{max} (thin film)/cm^{-1} 3398 (NH),

2980, 2919, 1712 (C=O), 1622, 1362, 1443, 1371, 1299, 1159, 1049, 859; δ_H (400 MHz, $CDCl_3$) 7.76 (br s, 1H), 7.51 (d, *J* = 7.0 Hz, 1H), 7.27–7.25 (m, 1H), 7.14–7.06 (m, 2H), 4.12 (q, *J* = 7.0 Hz, 2H), 3.04 (t, *J* = 8.0 Hz, 2H), 2.62 (t, *J* = 8.0 Hz, 2H), 2.39 (s, 3H), 1.23 (t, *J* = 7.0 Hz, 3H); δ_C (100.6 MHz, $CDCl_3$) 173.5 (C=O), 135.1 (C), 131.3 (C), 128.1 (C), 120.8 (CH), 119.0 (CH), 117.7 (CH), 110.2 (CH), 109.9 (C), 60.3 (CH_2), 35.1 (CH_2), 19.6 (CH_2), 14.1 (CH_3), 11.3 (CH_3); HRMS (ESI) *m/z* calcd for $C_{14}H_{17}NO_2$ $(M + H)^+$ 232.1332, found 232.1334 (−0.7 ppm error). These data are consistent with the literature values.[21]

1 M $NaOH_{(aq)}$ (20 mL) was added to a stirred solution of the dimethyl-indole ester **SI-6** (888 mg, 3.84 mmol) in 1:1 MeOH-THF (80 mL) at rt. The resulting solution was stirred at rt for 8 h and then the solvent was evaporated under reduced pressure. The resulting residue was taken up in a minimum amount of water (5 mL) and the resulting solution was taken to pH 1 with conc. $HCl_{(aq)}$ resulting in an oily residue. Water (40 mL) was added and the resulting mixture was extracted into CH_2Cl_2 (50 mL) and the two layers separated. The aqueous was extracted into CH_2Cl_2 (2 x 50 mL) and the combined organics dried ($MgSO_4$) and the solvent evaporated under reduced pressure to give the crude product. Purification by flash column chromatography on silica using hexane-EtOAc-AcOH (60:39.5:0.5) as eluent gave the indole acid **5i** (476 mg, 61%) as a yellow solid, mp 121–123 °C (lit.[22] 132–134 °C); ν_{max} (thin film)/cm^{-1} 3384 (NH), 2929, 2634, 1692 (C=O), 1464, 1406, 1308, 1294, 1211, 920; δ_H (400 MHz, DMSO-d_6) 12.02 (s, 1H), 10.68 (s, 1H), 7.39 (d, *J* = 7.5 Hz, 1H), 7.22–7.20 (m, 1H), 6.97 (ddd, *J* = 7.5, 7.5, 1.0 Hz, 1H), 6.91 (ddd, *J* = 7.5, 7.5, 1.0 Hz, 1H), 2.87 (t, *J* = 7.5 Hz, 2H), 2.45 (t, *J* = 7.5, 2H), 2.31 (s, 3H); δ_C (100.6 MHz, DMSO-d_6) 174.2 (C=O), 135.2 (C), 131.7 (C), 127.9 (C), 119.9 (CH), 118.1 (CH), 117.3 (CH), 110.4 (CH), 108.9 (C), 35.1 (CH_2), 19.4 (CH_2), 11.2 (CH_3); HRMS (ESI) *m/z* calcd for $C_{12}H_{13}NO_2$ $(M + H)^+$ 204.1019, found 204.1014 (+2.5 ppm error).

(5-Methoxy-2-methyl-1*H*-pyrrolo[3,2-b]pyridin-3-yl)acetic acid (10)

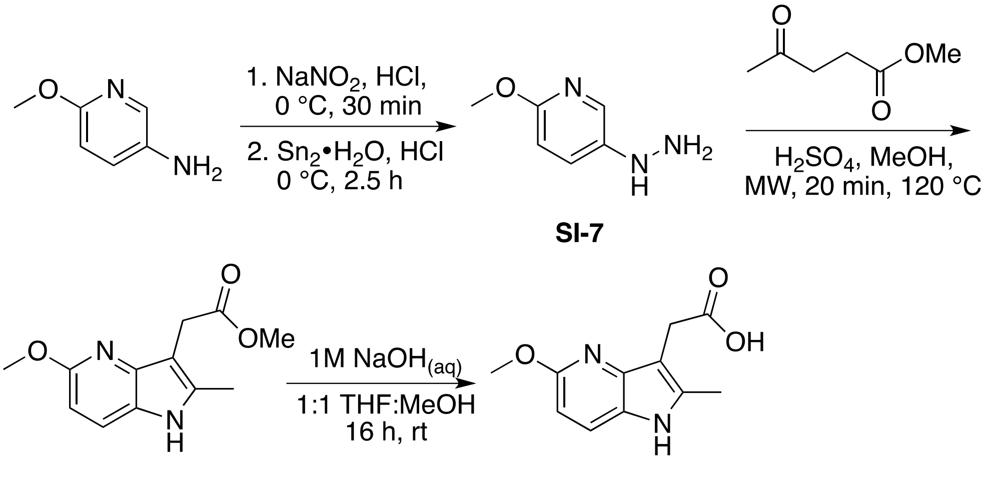

SI-7

SI-8 10

The following procedure was adapted from a literature protol.[1] A mixture of methyl 4-oxopentanoate (136 μL, 1.08 mmol) and 5-hydrazinyl-2-methoxypyridine **SI-7 (**synthesised following a literature procedure,[23] 140 mg, 1.00 mmol) in MeOH (3 mL) and conc. $H_2SO_{4(aq)}$ (120 μL) was placed in a suitable microwave vessel. The vessel was sealed and the mixture stirred for 10 min at 120 °C in a microwave synthesiser. After being allowed to cool to rt, the mixture was diluted with EtOAc (20 mL) and washed with 10% aq. HCl (20 mL). The two layers were separated and the aqueous layer was taken to pH 7 with sat. $NaHCO_{3(aq)}$. The aqueous layer was extracted into EtOAc (3 x 20 mL) and the combined organics were dried ($MgSO_4$) and the solvent evaporated under reduced pressure to give the indole ester **SI-8** (181 mg, 77%) as a brown oil, ν_{max} (thin film)/cm^{-1} 3344 (NH), 2949, 1722 (C=O), 1619, 1579, 1475, 1436, 1288, 1241, 1166, 1100, 1023, 803; δ_H (400 MHz, $CDCl_3$) 8.46 (s, 1H), 7.32 (d, *J* = 8.5 Hz, 1H), 6.42 (d, *J* = 8.5 Hz, 1H), 3.94 (s, 3H), 3.77 (s, 2H), 3.69 (s, 3H), 2.31 (s, 3H); δ_C (100.6 MHz, $CDCl_3$) 173.1 (C=O), 159.6 (C), 142.4 (C), 135.9 (C), 123.6 (C), 120.8 (CH), 104.6 (C), 103.4 (CH), 53.3 (CH_3), 51.9 (CH_3), 28.4 (CH_2), 12.2 (CH_3); HRMS (ESI) *m/z* calcd for $C_{12}H_{14}N_2O_3$ $(M + H)^+$ 235.1077, found 235.1083 (−2.6 ppm error).

1 M $NaOH_{(aq)}$ (4 mL) was added to a stirred solution of the azaindole ester **SI-8** (183mg, 0.781 mmol) in 1:1 MeOH-THF (15 mL) at rt. The resulting solution was stirred at rt for 16 h and then the solvent was evaporated under reduced pressure. The resulting residue was taken up in a minimum amount of water (5 mL) and the resulting solution was taken to pH 7 with 5M $HCl_{(aq)}$. The mixture was extracted into EtOAc (3 x 20 mL), the combined organics dried ($MgSO_4$) and the solvent evaporated under reduced pressure to give azaindole acid **10** (140 mg, 81%) as a yellow solid, 191–193 °C; ν_{max} (thin film)/cm^{-1} 3192 (NH),

2968, 1697 (C=O), 1623, 1581, 1447, 1405, 1315, 1250, 1107, 1017, 818, 592; δ_H (400 MHz, DMSO-d_6) 12.08 (s, 1H), 10.91 (s, 1H), 7.53 (d, J = 8.5 Hz, 1H), 6.42 (d, J = 8.5 Hz, 1H), 3.83 (s, 3H), 3.58 (s, 2H), 2.32 (s, 3H); δ_C (100.6 MHz, DMSO-d_6) 173.1 (C=O), 158.6 (C), 142.3 (C), 136.3 (C), 123.3 (C), 121.0 (CH), 104.3 (C), 102.7 (CH), 52.4 (CH_3), 28.3 (CH_2), 12.0 (CH_3); HRMS (ESI) *m/z* calcd for $C_{11}H_{12}N_2O_3$ $(M + H)^+$ 221.0921, found 221.0922 (−0.6 ppm error).

(3,5-Dimethyl-1*H*-pyrrol-2-yl)acetic acid (12)

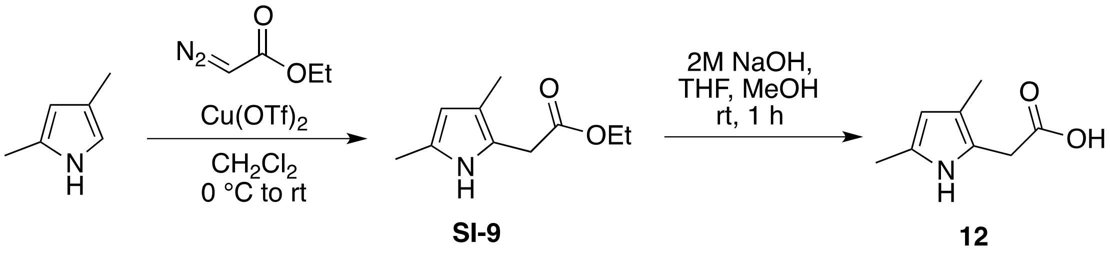

2,4-Dimethylpyrrole (1.00 g, 10.5 mmol) and ethyl diazoacetate (1.20 g, 1.11 ml, 10.5 mmol) were dissolved in CH_2Cl_2 and the resulting solution cooled to 0 °C. $Cu(OTf)_2$ (190 mg, 0.526 mmol) was added in portions and effervescence noted. After complete addition the reaction was stirred at r.t. for 1 h. The reaction mixture was concentrated and purification by column chromatography (9:1 hexane:EtOAc) gave the ester **SI-9** (831 mg, 44%) as an orange oil, R_f (9:1 hexane:EtOAc) 0.30; ν_{max} (thin film)/cm^{-1} 3384, 2981, 2924, 2868, 1720, 1399, 1368, 1300, 1239, 1163, 1114, 1027, 782, 638; δ_H (400 MHz, $CDCl_3$) 8.16 (br s, 1H), 5.68 (br s, 1H), 4.17 (q, J = 7.0 Hz, 2H), 3.54 (s, 2H), 2.23 (s, 3H), 2.00 (s, 3H), 1.29 (t, J = 7.0 Hz, 3H); δ_C (100 MHz, $CDCl_3$) 171.5 (C=O), 126.5 (C), 117.7 (C), 115.9 (C), 107.7 (CH), 60.9 (CH_2), 31.2 (CH_2), 14.1 (CH_3), 12.9 (CH_3), 10.6 (CH_3); HRMS (ESI): *m/z* calcd for $C_{10}H_{15}NO_2$ $[M + H]^+$ 182.1176, found 182.1175 (−0.2 ppm error).

2 M $NaOH_{(aq)}$ (20 mL) was added to a stirred solution of the pyrrole ester **SI-9** (600 mg, 3.31 mmol) in 1:10 MeOH-THF (2.5:25 mL) at rt and the resulting solution was stirred at rt for 1 h. H_2O (20 mL) was added and the reaction was washed with EtOAc (50 mL). The aqueous layer was acidified (pH 1) with conc. $HCl_{(aq)}$ and extracted with EtOAc (3 x 20 mL). The combined organics were dried ($MgSO_4$) and the solvent evaporated under reduced pressure to give the pyrrole acid **12** as a brown oil; (310 mg), δ_H (400 MHz, $CDCl_3$) 8.04 (br s, 1H), 5.69 (d, J = 2.5 Hz, 1H), 3.59 (s, 2H), 2.23 (s, 3H), 2.01 (s, 3H). The unpurified acid **12** was used directly in the subsequent spirocyclisation with imine **6a**, using general procedure A.

(3-Methyl-5-phenyl-1*H*-pyrrol-2-yl)acetic acid (13)

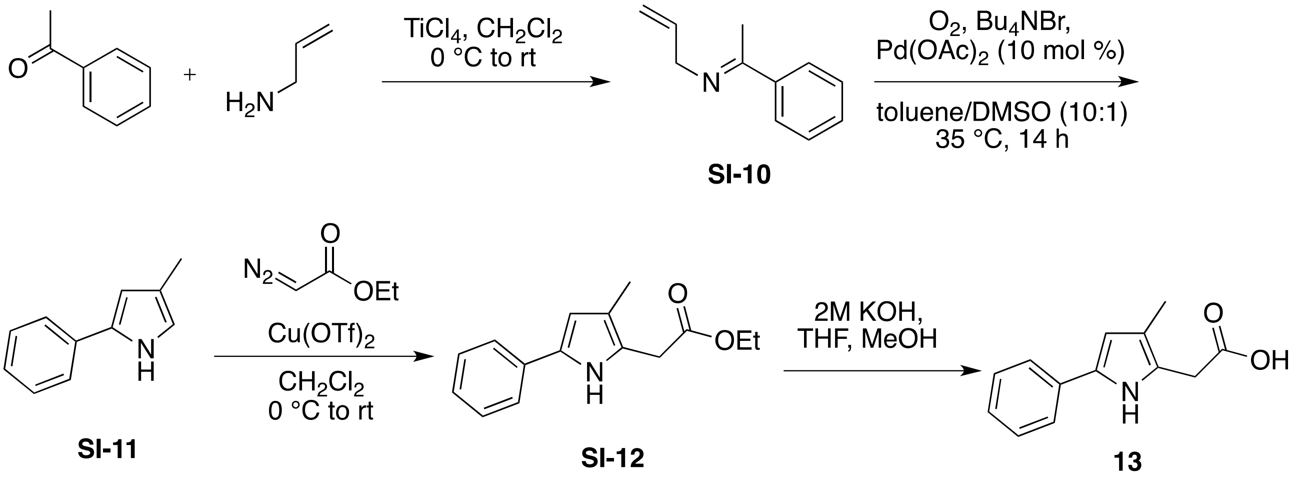

A solution of acetophenone (2.00 g, 1.94 mL, 16.6 mmol) and allylamine (4.75 g, 6.24 mL, 83.2 mmol) in CH_2Cl_2 (50 mL) was cooled to 0 °C. $TiCl_4$ (1 M in CH_2Cl_2) solution (10.7 mL, 10.7 mmol) was added dropwise over 40 min. The reaction mixture was then stirred at room temperature for 2 h and the resultant precipitate filtered. The filtrate was washed with sat. NaCl (100 mL), dried ($MgSO_4$), filtered and concentrated to give the imine **SI-10** (2.57 g, 97%) as an orange liquid, ν_{max} (thin film)/cm^{-1} δ_H (400 MHz, $CDCl_3$) 7.78–7.85 (m, 2H), 7.42–7.35 (m, 3H), 6.19–6.07 (m, 1H), 5.27 (ddt, *J* = 17.0, 1.5, 1.5 Hz, 1H), 5.16 (ddt, *J* = 10.5, 1.5, 1.5 Hz, 1H), 4.22–4.17 (m, 2H), 2.26 (s, 3H); δ_C (100 MHz, $CDCl_3$) 166.2 (C=N), 141.1 (C), 136.0 (CH), 129.5 (CH), 128.2 (2 × CH), 126.6 (2 × CH), 115.1 (CH_2), 54.6 (CH_2), 15.6 (CH_3); HRMS (ESI) *m/z* calcd for $C_{11}H_{13}N$ $[M + H]^+$ 160.1121, found 160.1126. These data are consistent with the literature values.[24]

Following a modified procedure of Yoshikai;[24] Imine **SI-10** (2.50 g, 15.7 mmol) was placed in a round bottomed flask with a stirrer bar and the flask flushed with O_2. Toluene (18.8 mL) and DMSO (1.88 mL) were added followed by Bu_4NBr (5.06 g, 15.7 mmol) and $Pd(OAc)_2$ (106 mg, 1.57 mmol). The flask was flushed a few times with O_2 and then the flask was sealed with a suba seal and a balloon of O_2 put in place. The reaction was stirred at 35 °C for 14 h. Water (100 mL) was added to the reaction and the mixture extracted with Et_2O (3 × 100 mL). The combined organic phases were dried ($MgSO_4$), filtered, and concentrated to give the crude product. Purification by column chromatography (9:1 hexane:EtOAc) gave the pyrrole **SI-11** (959 mg, 39%) as a pink solid, δ_H (400 MHz, $CDCl_3$) 8.18 (br s, 1H), 7.48–7.43 (m, 2H), 7.39–7.33 (m, 2H), 7.23–7.17 (m, 1H), 6.66–6.62 (m, 1H), 6.40–6.36 (m, 1H), 2.17 (m, 3H);

HRMS (ESI) *m/z* calcd for $C_{11}H_{11}N$ $[M + H]^+$ 158.0964, found 158.0962. These data are consistent with the literature values.[24]

Pyrrole **SI-11** (750 mg, 4.77 mmol) and ethyl diazoactetate (544 mg, 502 µL, 4.77 mmol) were dissolved in CH_2Cl_2 (10 ml) and stirred at 0 °C. $Cu(OTf)_2$ (86.3 mg, 0.239 mmol) was added carefully in portions. The reaction was stirred at 0 °C for 1 h and then allowed to warm to room temperature being concentrated to give the crude product. This was purified by column chromatography (9:1 hexane:EtOAc) to give the ester **SI-12** (390 mg, 34%) as a pink solid, R_f (9:1 hexane:ethyl acetate) 0.30; ν_{max} (thin film)/cm^{-1} 3362, 2977, 2936, 2865, 1704 (C=O), 1593, 1517, 1471, 1291, 1260, 1168, 1126, 1027, 805, 795, 757, 695; δ_H (400 MHz, $CDCl_3$) 8.84 (br s, 1H), 7.48–7.43 (m, 2H), 7.38–7.31 (m, 2H), 7.21–7.15 (m, 1H), 6.31 (d, *J* = 2.5 Hz, 1H), 4.21 (q, *J* = 7.0 Hz, 2H), 3.65 (s, 2H), 2.08 (s, 3H), 1.31 (t, *J* = 7.0 Hz, 3H); δ_C (100 MHz, $CDCl_3$) 171.3 (C=O), 132.7 (C), 130.7 (C), 128.7 (2 × CH), 125.8 (CH), 123.4 (2 × CH), 120.8 (C), 117.6 (C), 107.5 (CH), 61.1 (CH_2), 31.3 (CH_2), 14.1 (CH_3), 10.8 (CH_3); HRMS (ESI): calcd for $C_{15}H_{17}NO_2$ $[M + H]^+$ 244.1332, found 244.1334 (−0.9 ppm error).

Ester **SI-12** (340 mg, 1.397 mmol) was dissolved in THF (1.79 mL) and MeOH (199 µL) and 2M KOH (2.98 mL) was added. The reaction was stirred for 16 h at rt, water (10 mL) was added and the reaction washed with EtOAc (10 mL). The aqueous layer was acidified (pH 1) with conc. $HCl_{(aq)}$ and extracted with EtOAc (3 x 10 mL). The combined organics were dried ($MgSO_4$) and the solvent evaporated under reduced pressure to give the crude product. Flash column chromatography on silica using EtOAc as eluent gave the pyrrole acid **13** (194 mg, 64%) as a yellow oil, R_f (EtOAc) 0.35; ν_{max} (thin film)/cm^{-1} 3424, 2912, 2866, 1696, 1518, 1390, 1264, 1221, 918, 809, 761, 694, 523; δ_H (400 MHz, $CDCl_3$) 8.63 (br s, 1H), 7.48–7.41 (m, 2H), 7.38–7.31 (m, 2H), 7.22–7.15 (m, 1H), 6.32 (d, *J* = 3.0 Hz, 1H), 3.70 (s, 2H), 2.08 (s, 3H); δ_C (100 MHz, $CDCl_3$) 176.9 (C=O), 132.6 (C), 131.1 (C), 128.8 (2 × CH), 126.0 (CH), 123.5 (2 × CH), 119.9 (C), 118.3 (C), 107.7 (CH), 31.3 (CH_2), 10.8 (CH_3); HRMS (ESI): calcd for $C_{13}H_{13}NO_2$ $[M + Na]^+$ 238.0838, found 238.0838.

[5-(Ethoxycarbonyl)-2,4-dimethyl-1*H*-pyrrol-3-yl]acetic acid (16)

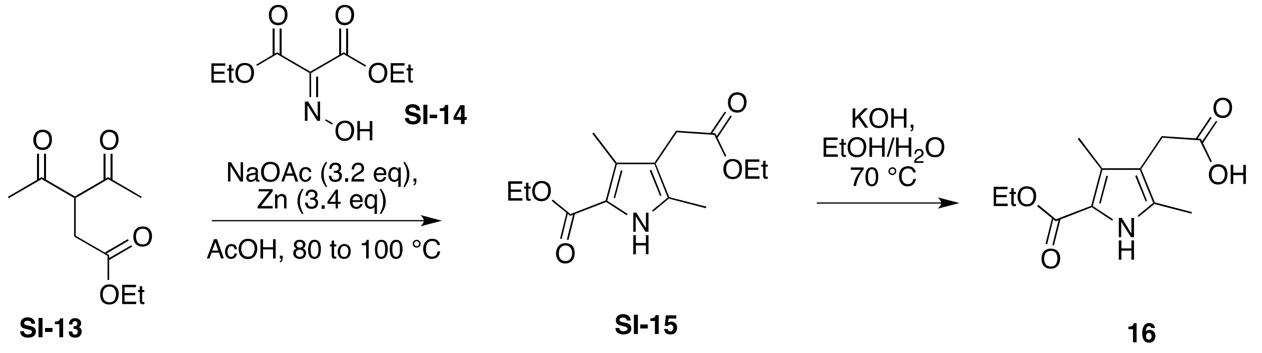

Following a modified procedure of Lightner and Holmes;[25] Diketo ester **SI-13**[26] (4.43 g, 23.788 mmol) was dissolved in acetic acid (12.6 mL) and the solution stirred at 80 °C. At this temperature NaOAc (6.24 g, 76.122 mmol, 3.2 eq.) and zinc dust (5.29 g, 80.880 mmol, 3.4 eq.) were added as solids. The reaction temperature was increased to 95 °C and at this temperature a solution of diethyl oximinomalonate **SI-14**[27] (4.50 g, 23.788 mmol, 1 eq.) in AcOH / H_2O (5.71 mL / 2.38 mL) was added dropwise over 30 min. The reaction mixture was then heated at 100 °C for 1 h before being allowed to cool. Ice was added and the reaction mixture shaken and then stored in a fridge overnight. The resultant solids were filtered and washed with H_2O. Purification by column chromatography (9:1 to 4:1 hexane:EtOAc) gave the pyrrole **SI-15** (1.67 g, 28%) as a white solid, mp 82–82 °C [lit.,[25] 93–95 °C (from EtOH)]; R_f (4:1 hexane:EtOAc) 0.25; ν_{max} (thin film)/cm^{-1} 3294, 2984, 2956, 2903, 1729, 1663 (C=O), 1444, 1264, 1252, 1218, 1170, 1093, 1022, 918, 774, 751; δ_H (400 MHz, $CDCl_3$) 8.73 (br s, 1H), 4.30 (q, J = 7.0 Hz, 2H), 4.13 (q, J = 7.0 Hz, 2H), 3.37 (s, 2H), 2.29 (s, 3H), 2.24 (s, 3H), 1.35 (t, J = 7.0 Hz, 3H), 1.25 (t, J = 7.0 Hz, 3H); δ_C (100 MHz, $CDCl_3$) 171.8 (C=O), 161.7 (C=O), 130.8 (C), 127.5 (C), 117.1 (C), 114.5 (C), 60.6 (CH_2), 59.7 (CH_2), 30.2 (CH_2), 14.5 (CH_3), 14.2 (CH_3), 11.5 (CH_3), 10.6 (CH_3); HRMS (ESI): calcd for $C_{13}H_{19}NO_4$ $[M + Na]^+$ 276.1206, found 276.1201. These data are consistent with the literature values.[25]

The pyrrole diester **SI-15** was dissolved in water (3.2 mL) and EtOH (1.6 mL) and heated to 70 °C. The reaction was stirred for 30 min then allowed to cool to rt. 10 % $HCl_{(aq)}$ (10 mL) and the reaction was extracted with EtOAc (3 x 10 mL). The combined organics were dried ($MgSO_4$) and the solvent evaporated under reduced pressure to give the acid **16** (192 mg, 54%) as a brown solid, mp 160–162 °C (lit.[25] 194–196 °C) R_f (EtOAc) 0.55; ν_{max} (thin film)/cm^{-1} 3304, 2985, 2917, 1706, 1670 (C=O), 1444, 1274, 1218, 1172, 1091, 770, 722, 699, 623; δ_H (400 MHz, DMSO-d_6) 12.07 (br s, 1H), 11.15 (br s, 1H), 4.19 (q, J = 7.0 Hz, 2H), 3.24 (s, 2H), 2.15 (s, 3H), 2.11 (s, 3H), 1.26 (t, J = 7.0 Hz, 3H); δ_C (100 MHz, DMSO-d_6) 173.0 (C=O), 160.9 (C=O), 131.5 (C), 126.5 (C), 116.0 (C), 114.6 (C), 58.8 (CH_2), 29.7

(CH_2), 14.6 (CH_3), 10.9 (CH_3), 10.5 (CH_3); HRMS (ESI): calcd for $C_{11}H_{15}NO_4$ $[M + Na]^+$ 248.0893, found 248.0892. These data were consistent with the literature.[25]

(6) Synthesis of the imine substrates

6,7-Dimethoxy-3,4-dihydroisoquinoline (6b)

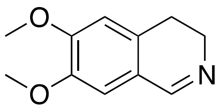

6b

N-Bromosuccinimide (840 mg, 4.72 mmol) was added portionwise to a stirred solution of 6,7-dimethoxy-1,2,3,4-tetrahydroisoquinoline **6b** (830 mg, 4.30 mmol) in CH_2Cl_2 (20 mL) at 0 °C under Ar. The resulting solution was stirred at 0 °C for 1 h. $NaOH_{(aq)}$ (30% w/v, 9 mL) was added and the reaction was stirred at rt for 2 h. The two layers were separated and the organic layer was washed with water (50 mL), dried ($MgSO_4$) and evaporated under reduced pressure to give the crude product. Purification by flash column chromatography on silica using EtOAc-MeOH (98:2) as eluent gave the imine **6b** (766 mg, 93%) as a pale yellow oil, R_f (95:5 EtOAc-MeOH) 0.15; δ_H (400 MHz, $CDCl_3$) 8.23 (s, 1H), 6.81 (s, 1H), 6.67 (s, 1H), 3.91 (s, 3H), 3.90 (s, 3H), 3.75–3.70 (m, 2H), 2.70–2.66 (m, 2H); δ_C (100.6 MHz, $CDCl_3$) 159.6 (C=N), 151.1 (C), 147.8 (C), 129.8 (C), 121.5 (C), 110.3 (CH), 110.3 (CH), 56.1 (CH_3), 56.0 (CH_3), 47.3 (CH_2), 24.7 (CH_2). These data are consistent with the literature values.[28]

4,5-Dihydrothieno[2,3-c]pyridine (6d)

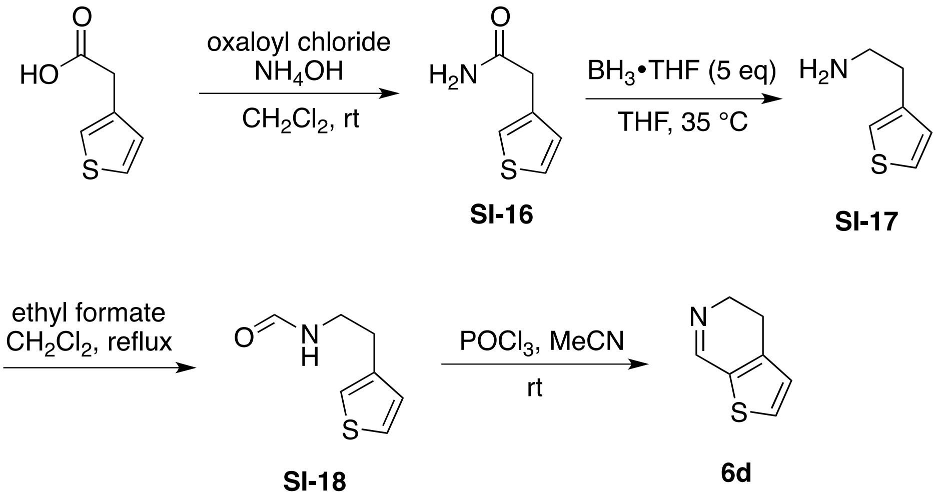

3-Thiophene acetic acid (3.80 g, 26.7 mmol) was dissolved in CH_2Cl_2 (45 mL) and stirred at room temperature. Oxalyl chloride (2.49 mL, 29.4 mmol, 1.1 eq.) was added and the reaction stirred at room

temperature overnight. Aqueous ammonium hydroxide (16 mL) was added slowly, in portions. Water (100 mL), sat. $NaHCO_3$ (100 mL), and EtOAc (200 mL) were added. The organic phase was isolated and the aqueous phase was extracted with EtOAc (2 × 100 mL). The combined organics were dried ($MgSO_4$), filtered, and concentrated to give the amide **SI-16** (3.19 g, 85%) as a white solid, R_f (EtOAc) 0.30; ν_{max} (thin film)/cm^{-1} 3344, 3159, 1631 (C=O), 1409, 1282, 1243, 831, 783, 734, 648, 596, 585; δ_H (400 MHz, $CDCl_3$) 7.36 (dd, *J* = 5.0, 3.0 Hz, 1H), 7.19–7.16 (m, 1H), 7.03 (dd, *J* = 5.0, 1.5 Hz, 1H), 5.72 (br s, 1H), 5.50 (br s, 1H), 3.63 (s, 2H); HRMS (ESI) *m/z* calcd for C_6H_7NOS $[M + H]^+$ 142.0321, found 142.0319 (+1.3 ppm error). These data are consistent with the literature values.[29]

A suspension of amide **SI-16** (2.00 g, 14.2mmol) in THF (12 mL) was cooled to 0 °C. BH_3·THF (1 M) (70.8 mL, 70.8 mmol, 5 eq.) was added in portions. The reaction was stirred at 35 °C overnight before being allowed to cool to room temperature. MeOH (50 mL) was added (note: effervescence) followed by 6 M HCl (50 mL) and the reaction mixture stirred for 2 h. The reaction was then diluted with H_2O (50 mL) and the resulting solution washed with EtOAc (50 mL). The aqueous phase was isolated, made basic with 2 M NaOH, and extracted with EtOAc (4 × 50 mL). The combined organic phases were dried ($MgSO_4$), filtered, and concentrated to give the crude product, which contained amine **SI-17**, as a colourless oil (1.10 g), δ_H (400 MHz, $CDCl_3$) 7.29 (dd, *J* = 5.0, 3.0 Hz, 1H), 7.03–6.99 (m, 1H), 6.97 (dd, *J* = 5.0, 1.5 Hz, 1H), 2.98 (m, 2H), 2.80 (t, *J* = 7.0 Hz, 2H); HRMS (ESI) *m/z* calcd for C_6H_9NS $[M + H]^+$ 128.0528, found 128.0534. These data are consistent with the literature values.[30]

The crude material, containing amine **SI-17**, was dissolved in ethyl formate (25 mL) and stirred at reflux for 5 h. The reaction mixture was concentrated, dissolved in CH_2Cl_2 (50 mL) and washed with 10% HCl (50 mL) and then sat. $NaHCO_3$ (50 mL). The organic phase was dried ($MgSO_4$), filtered, and concentrated to give the crude product, which contained formate **SI18**, as a brown oil (616 mg), δ_H (400 MHz, $CDCl_3$) 8.15 (s, 1H), 7.31 (dd, *J* = 5.0, 3.0 Hz, 1H), 7.05–7.02 (m, 1H), 6.97 (dd, *J* = 5.0, 1.5 Hz, 1H), 5.64 (br s, 1H), 3.58 (m, 2H), 2.89 (t, *J* = 7.0 Hz, 2H).

The crude material, containing formate **SI-18**, was dissolved in MeCN (8.57 mL). $POCl_3$ (429 µL) was added and the reaction mixture stirred at room temperature for 4 h. The reaction mixture was concentrated, dissolved in EtOAc (50 mL), and washed with sat. $NaHCO_3$ (50 mL). The organic phase was dried ($MgSO_4$), filtered, and concentrated to give the imine **6d** (351 mg, 18% over 3 steps) as a brown oil, δ_H (400 MHz, $CDCl_3$) 8.29 (s, 1H), 7.38 (d, *J* = 5.0 Hz, 1H), 6.93 (d, *J* = 5.0 Hz, 1H), 3.81 (td, *J* = 8.5, 2.0 Hz, 2H), 2.77 (t, *J* = 8.5 Hz, 2H); δ_C (100 MHz, $CDCl_3$) 152.5 (CH), 140.7 (C), 129.5 (C), 127.7 (CH),

126.6 (CH), 47.6 (CH_2), 22.7 (CH_2); HRMS (ESI): calcd for C_7H_7NS $[M + H]^+$ 138.0372, found 138.0376 (−3.1 ppm error). These data are consistent with the literature values.[31]

1-Methyl-6,7-dihydro-1*H*-pyrrolo[3,2-c]pyridine (6e)

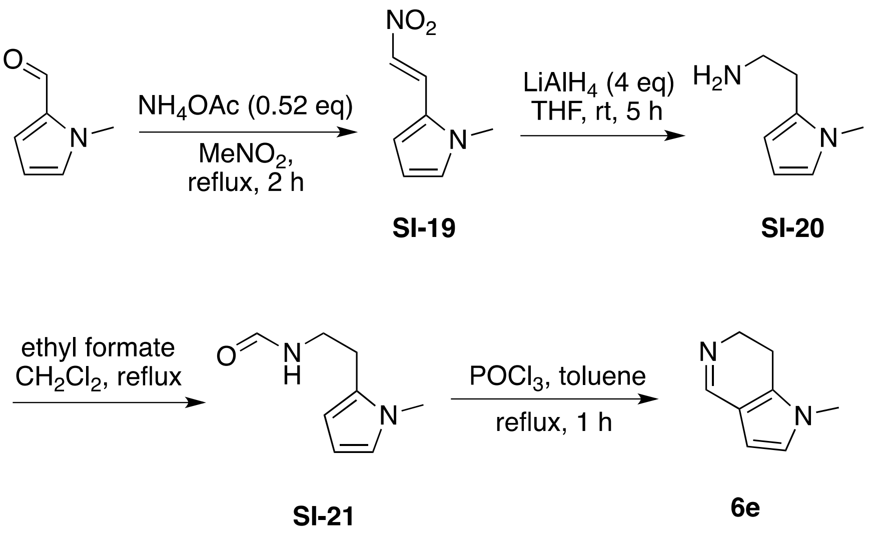

N-Methyl-2-pyrrole carboxaldehyde (10.0 g, 91.634 mmol), NH_4OAc (3.91 g, 47.650 mmol), and $MeNO_2$ (50 mL) were stirred at reflux for 2 h. The mixture was allowed to cool and the $MeNO_2$ removed under vacuum. The residue was dissolved in CH_2Cl_2 (100 mL), washed with water (2 × 100 mL), dried ($MgSO_4$), filtered, and concentrated to give the crude product (14.9 g), which contained nitrostyrene **SI-19**, δ_H (400 MHz, $CDCl_3$) 8.00 (d, *J* = 13.0 Hz, 1H), 7.47 (d, *J* = 13.0 Hz, 1H), 6.97–6.93 (m, 1H), 6.81 (dd, *J* = 4.0, 1.5 Hz, 1H), 6.28 (dd, *J* = 4.0, 2.5 Hz, 1H), 3.78 (s, 3H); HRMS (ESI): calcd for $C_7H_8N_2O_2$ $[M + Na]^+$ 175.0478, found 175.0483. These data are consistent with the literature values.[32]

Following a modified procedure of Glennon;[33] the crude material from above, containing nitroalkene **SI-19** (14.9 g, 97.930 mmol) was dissolved in THF (400 mL) and $LiAlH_4$ (11.15 g, 293.789 mmol, 3.0 eq.) was added in portions very carefully. The resulting mixture was stirred at room temperature for 5 h. TLC at this stage showed the starting material to be consumed. The reaction mixture was cooled to 0 °C and quenched very carefully with H_2O before being filtered through a pad of celite. The celite was washed with EtOAc (2 × 200 mL). The filtrate was treated with 10% aq. HCl (200 mL) and the aqueous phase isolated. This was basified with 2 M NaOH and extracted with EtOAc (3 × 200 mL). The combined organic phases were dried ($MgSO_4$), filtered, and concentrated to give the crude product, which contained amine **SI-20**, as a brown oil, δ_H (400 MHz, $CDCl_3$) 6.57 (dd, *J* = 2.5, 2.0 Hz, 1H), 6.09–6.06 (m, 1H),

5.95–5.92 (m, 1H), 3.56 (s, 3H), 2.97 (t, J = 7.0 Hz, 2H), 2.72 (t, J = 7.0 Hz, 2H); HRMS (ESI): calcd for $C_7H_{12}N_2$ $[M + H]^+$ 125.1073, found 125.1068. These data are consistent with the literature values.[34]

The crude material, containing amine **SI-20**, was dissolved in ethyl formate (10 mL) and stirred at reflux for 5 h. The reaction mixture was concentrated, dissolved in CH_2Cl_2 (25 mL) and washed with 10% HCl (25 mL) and then sat. $NaHCO_3$ (25 mL). The organic phase was dried ($MgSO_4$), filtered, and concentrated to give the crude product (8.21 g), as a brown oil. Purification by column chromatography (EtOAc) gave the formate **SI-21** (2.33 g, 17% over 3 steps), δ_H (400 MHz, $CDCl_3$) 8.16 (s, 1H), 6.59 (m, 1H), 6.08 (t, J = 3.0 Hz, 1H), 5.95 (m, 1H), 5.76 (br s, 1H), 3.60–3.53 (m, 2H), 3.57 (s, 3H), 2.82 (t, J = 7.0 Hz, 2H); HRMS (ESI) m/z calcd for $C_8H_{12}N_2O$ $[M + Na]^+$ 175.0842, found 175.0839. These data are consistent with the literature values.[33]

Following a modified procedure of Glennon;[33] formate **SI-21** (500 mg, 3.285 mmol) was dissolved in toluene (15.0 mL). The reaction was heated to reflux and $POCl_3$ (500 µL) added dropwise. The reaction was stirred at reflux for 1 h. The mixture was allowed to cool and washed with hot water (3 × 25 mL). The combined aqueous layers were washed with CH_2Cl_2 (3 × 15 mL) and basified by the addition of KOH. This alkaline aqueous phase was extracted with CH_2Cl_2 (3 × 25 mL), dried ($MgSO_4$), filtered, and concentrated to give imine **6e** (115 mg, 26%) as a yellow film, δ_H (400 MHz, $CDCl_3$) 8.21 (t, J = 2.0 Hz, 1H), 6.50 (d, J = 3.0 Hz, 1H), 6.18 (d, J = 3.0 Hz, 1H), 3.85–3.78 (m, 2H), 3.55 (s, 3H), 2.70–2.63 (m, 2H); HRMS (ESI): calcd for $C_8H_{10}N$ $[M + H]^+$ 135.0917, found 135.0918. These data are consistent with the literature values.[33]

(7) Acid scope in the DIA spirocyclisation reaction

(1'*R,10b'*R**)-2-Methyl-5',6'-dihydro-2'*H*-spiro[indole-3,1'-pyrrolo[2,1-*a*]isoquinolin]-3'(10b'*H*)-one (8a) and (1'*R**,10b'*S**)-2-methyl-5',6'-dihydro-2'*H*-spiro[indole-3,1'-pyrrolo[2,1-*a*]isoquinolin]-3'(10b'*H*)-one (9a)**

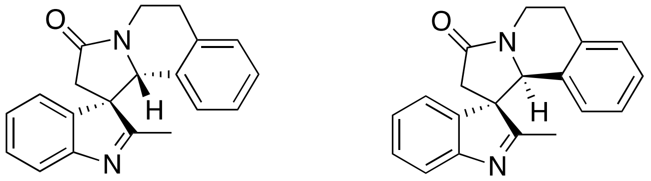

8a **9a**

General procedure A was followed using imine **6a** (100 mg, 0.762 mmol), acid **5a** (172 mg, 0.909 mmol), DIPEA (245 μL, 1.41 mmol) and T3P (725 mg of a 50% w/v solution in THF, 1.14 mmol), in THF (4 mL) for 16 h at RT. The unpurified reaction mixture contained 11:1 mixture of **8a**:**9a** based on analysis by ^{1}H NMR spectroscopy. Purification by flash column chromatography with EtOAc as eluent sequentially furnished **9a** (18 mg, 8%), a 1:3.3 mixture of **9a**:**8a** (27 mg, 12%) and **8a** (166 mg, 72%). Total combined yield of **8a** and **9a**: 211 mg, 92%. **8a** was recrystallised from EtOAc and an X-ray crystal structure obtained. CCDC 1436464 contains the supplementary crystallographic data for this paper The data can be obtained free of charge from The Cambridge Crystallographic Data Centre via www.ccdc.cam.ac.uk/data_request/cif

9a: Yellow oil, R_f (EtOAc) 0.25; ν_{max} (thin film)/cm^{-1} 3070, 2920, 2855, 1690 (C=O), 1578, 1458, 1424, 1305, 925, 758, 731; δ_H (400 MHz, $CDCl_3$) 7.58 (d, *J* = 7.5 Hz, 1H), 7.53 (d, *J* = 7.5 Hz, 1H), 7.46 (ddd, *J* = 7.5, 7.5, 1.0 Hz, 1H), 7.36 (ddd, *J* = 7.5, 7.5, 1.0 Hz, 1H), 7.12–7.09 (m, 2H), 6.91–6.85 (m, 1H), 5.93 (d, *J* = 7.5 Hz, 1H), 5.36 (s, 1H), 4.58–4.53 (m, 1H), 3.16–3.02 (m, 3H), 2.89–2.85 (m, 1H), 2.58 (d, *J* = 17.0 Hz, 1H), 1.85 (s, 3H); δ_C (100.6 MHz, $CDCl_3$) 182.4 (C=N), 170.0 (C=O), 154.8 (C), 140.2 (C), 133.0 (C), 132.0 (C), 129.3 (CH), 129.1 (CH), 127.7 (CH), 127.3 (CH), 126.3 (CH), 123.7 (CH), 121.6 (CH), 120.6 (CH), 61.7 (C), 61.6 (CH), 40.1 (CH_2), 37.1 (CH_2), 28.5 (CH_2), 17.2 (CH_3); HRMS (ESI) *m/z* calcd for $C_{20}H_{18}N_2O$ $(M + H)^+$ 303.1492, found 303.1497 (−1.8 ppm error).

8a: Yellow solid, mp 177–179 °C; R_f (EtOAc) 0.15; ν_{max} (thin film)/cm^{-1} 3070, 2921, 1690 (C=O), 1577, 1458, 1429, 1414, 1305, 909, 726; δ_H (400 MHz, $CDCl_3$) 7.40 (d, *J* = 7.5 Hz, 1H), 7.11 (ddd, *J* = 7.5, 7.5, 1.0 Hz, 1H), 6.99–6.91 (m, 3H), 6.86 (ddd, *J* = 7.5, 7.5, 1.0 Hz, 1H), 6.78 (dd, *J* = 7.5, 7.5 Hz, 1H), 6.32 (d, *J* = 7.5 Hz, 1H), 5.27 (s, 1H), 4.58–4.53 (m, 1H), 3.10 (d, *J* = 16.0 Hz, 1H), 3.09–2.97 (m, 2H), 2.82–

2.77 (m, 1H), 2.60 (s, 3H), 2.44 (d, J = 16.0 Hz, 1H); ^{13}C NMR (100.6 MHz, $CDCl_3$) 180.4 (C=N), 170.4 (C=O), 153.7 (C), 140.3 (C), 133.3 (C), 132.0 (C), 129.0 (CH), 128.4 (CH), 127.1 (CH), 126.6 (CH), 125.5 (CH), 123.5 (CH), 120.9 (CH), 120.1 (CH), 63.3 (C), 60.4 (CH), 39.9 (CH_2), 37.3 (CH_2), 28.9 (CH_2), 16.4 (CH_3); HRMS (ESI) *m/z* calcd for $C_{20}H_{18}N_2O$ $(M + H)^+$ 303.1492, found 303.1496 (−1.4 ppm error).

(1'*R,10b'*R**)-5-Methoxy-2-methyl-5',6'-dihydro-2'*H*-spiro[indole-3,1'-pyrrolo[2,1-*a*]isoquinolin]-3'(10b'*H*)-one (8b) and (1'*R**,10b'*S**)-5-methoxy-2-methyl-5',6'-dihydro-2'*H*-spiro[indole-3,1'-pyrrolo[2,1-*a*]isoquinolin]-3'(10b'*H*)-one (9b)**

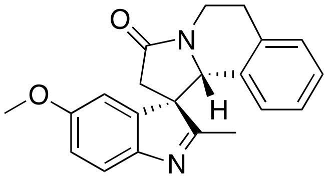

8b

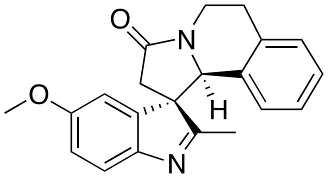

9b

General procedure A was followed using imine **6a** (100 mg, 0.762 mmol), acid **5b** (199 mg, 0.907 mmol), DIPEA (245 µL, 1.41 mmol) and T3P (725 mg of a 50% w/v solution in THF, 1.14 mmol), in THF (4 mL) for 16 h at RT. The unpurified reaction mixture contained 6:1 mixture of **8b**:**9b** based on analysis by ^{1}H NMR spectroscopy. Purification by flash column chromatography with EtOAc as eluent sequentially furnished **9b** (38 mg, 15%), a 1:3 mixture of **9b**:**8b** (18 mg, 7%) and **8b** (160 mg, 63%). Total combined yield of **8b** and **9b**: 216 mg, 85%.

9b: Pale orange solid, mp 168-171°C; R_f (EtOAc) 0.25; ν_{max} (thin film)/cm^{-1} 2923, 2836, 1690 (C=O), 1596, 1576, 1473, 1431, 1305, 1026, 909, 727; δ_H (400 MHz, $CDCl_3$) 7.48 (d, J = 8.5 Hz, 1H), 7.12–7.10 (m, 2H), 7.08 (d, J = 2.5 Hz, 1H), 6.95 (dd, J = 8.5, 2.5 Hz, 1H), 6.92–6.88 (m, 1H), 5.99 (d, J = 8.0 Hz, 1H), 5.30 (s, 1H), 4.56–4.52 (m, 1H), 3.88 (s, 3H), 3.15–3.02 (m, 3H), 2.92-2.84 (m, 1H), 2.59 (d, J = 17.0 Hz, 1H), 1.81 (s, 3H); δ_C (100.6 MHz, $CDCl_3$) 180.2 (C=N), 170.0 (C=O), 158.7 (C), 148.3 (C), 141.8 (C), 133.0 (C), 132.1 (C), 129.2 (CH), 127.7 (CH), 127.3 (CH), 123.8 (CH), 121.0 (CH), 113.4 (CH), 108.4 (CH), 61.9 (C), 61.7 (CH), 55.8 (CH_3), 40.5 (CH_2), 37.1 (CH_2), 28.5 (CH_2), 17.1 (CH_3); HRMS (ESI) *m/z* calcd for $C_{21}H_{20}N_2O_2$ $(M + H)^+$ 333.1598, found 333.1594 (+0.3 ppm error)

8b: Pale orange solid, mp 121–124 °C; R_f (EtOAc) 0.15; ν_{max} (thin film)/cm^{-1} 2934, 2836, 1689, 1610, 1579, 1470, 1431, 1305, 1178, 907, 724; δ_H (400 MHz, $CDCl_3$) 7.28 (d, J = 8.5 Hz, 1H), 6.99–6.92 (m,

2H), 6.82–6.78 (m, 1H), 6.62 (dd, J = 8.5, 2.5 Hz, 1H), 6.52 (d, J = 2.5 Hz, 1H), 6.33 (d, J = 8.0 Hz, 1H), 5.25 (s, 1H), 4.56–4.51 (m, 1H), 3.57 (s, 3H), 3.11–2.95 (m, 3H), 2.83–2.78 (m, 1H), 2.55 (s, 3H), 2.43 (d, J = 16.5 Hz, 1H); δ_C (100.6 MHz, $CDCl_3$) 178.0 (C=N), 170.4 (C=O), 157.8 (C), 147.5 (C), 141.8 (C), 133.2 (C), 132.0 (C), 129.0 (CH), 127.1 (CH), 126.7 (CH), 123.6 (CH), 120.3 (CH), 113.2 (CH), 107.5 (CH), 63.3 (C), 60.7 (CH), 55.5 (CH_3), 40.0 (CH_2), 37.3 (CH_2), 29.0 (CH_2), 16.2 (CH_3); HRMS (ESI) *m/z* calcd for $C_{21}H_{20}N_2O_2$ $(M + H)^+$ 333.1598, found 333.1595 (+0.8 ppm error).

(1'*R,10b'*R**)-5-Fluoro-2-methyl-5',6'-dihydro-2'*H*-spiro[indole-3,1'-pyrrolo[2,1-*a*]isoquinolin]-3'(10b'*H*)-one (8c) and (1'*R**,10b'*S**)-5-fluoro-2-methyl-5',6'-dihydro-2'*H*-spiro[indole-3,1'-pyrrolo[2,1-*a*]isoquinolin]-3'(10b'*H*)-one (9c)**

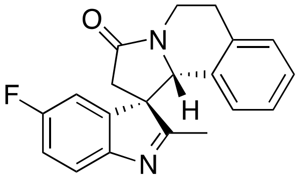

8c

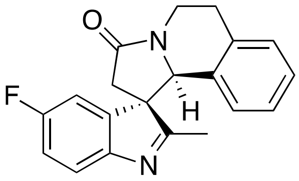

9c

General procedure A was followed using imine **6a** (100 mg, 0.762 mmol), acid **5c** (188 mg, 0.907 mmol), DIPEA (245 µL, 1.41 mmol) and T3P (725 mg of a 50% w/v solution in THF, 1.14 mmol), in THF (4 mL) for 16 h at RT. The unpurified reaction mixture contained 6:1 mixture of **8c**:**9c** based on analysis by ^1H NMR spectroscopy. Purification by flash column chromatography with EtOAc as eluent sequentially furnished **9c** (21 mg, 9%) and **8c** (177 mg, 72%). Total combined yield of **8c** and **9c**: 198 mg, 81%.

9c: Yellow oil, R_f (EtOAc) 0.25; ν_{max} (thin film)/cm^{-1} 2925, 1696 (C=O), 1601, 1466, 1421, 1306, 1156, 732; δ_H (400 MHz, $CDCl_3$) 7.53 (dd, J = 8.5, 4.5 Hz, 1H), 7.25 (dd, J = 7.5, 2.5 Hz, 1H), 7.17–7.12 (m, 3H), 6.93–6.89 (m, 1H), 5.96 (d, J = 8.0 Hz, 1H), 5.32 (s, 1H), 4.58–4.53 (m, 1H), 3.16–3.02 (m, 3H), 2.90–2.85 (m, 1H), 2.60 (d, J = 17.0 Hz, 1H), 1.84 (s, 3H); δ_C NMR (100.6 MHz, $CDCl_3$) 182.3 (C=N), 169.7 (C=O), 161.6 (d, J = 245.5 Hz, CF), 150.7 (d, J = 2.0 Hz, C), 142.2 (d, J = 8.5 Hz, C), 133.1 (C), 131.8 (C), 129.4 (CH), 127.9 (CH), 127.4 (CH), 123.6 (CH), 121.5 (d, J = 8.5 Hz, CH), 115.8 (d, J = 23.5 Hz, CH), 109.5 (d, J = 25.0 Hz, CH), 62.1 (d, J = 2.0 Hz, C), 61.6 (CH), 40.1 (CH_2), 37.2 (CH_2), 28.5 (CH_2), 17.2 (CH_3); ^{19}F NMR (376 MHz, $CDCl_3$) δ −115.00 (ddd, J = 8.5, 8.5, 4.5 Hz); HRMS (ESI) *m/z* calcd for $C_{20}H_{17}FN_2O$ $(M + H)^+$ 321.1398, found 321.1383 (+4.5 ppm error).

8c: Yellow solid, mp 193–196 °C; R_f (EtOAc) 0.15; ν_{max} (thin film)/cm^{-1} 2924, 1694 (C=O), 1598, 1462, 1415, 1178, 920, 727; δ_H (400 MHz, $CDCl_3$) 7.33 (dd, *J* = 8.5, 4.5 Hz, 1H), 7.02–6.95 (m, 2H), 6.83–6.78 (m, 2H), 6.68 (dd, *J* = 8.0, 2.5 Hz, 1H), 6.32 (d, *J* = 8.0 Hz, 1H), 5.26 (s, 1H), 4.56–4.52 (m, 1H), 3.12–2.96 (m, 3H), 2.84–2.79 (m, 1H), 2.58 (s, 3H), 2.43 (d, *J* = 16.5 Hz, 1H); δ_C (100.6 MHz, $CDCl_3$) 180.3 (C=N), 170.1 (C=O), 160.8 (d, *J* = 245.5 Hz, CF), 149.8 (d, *J* = 2.0 Hz, C), 142.1 (d, *J* = 9.0 Hz, C), 133.3 (C), 131.6 (C), 129.3 (CH), 127.3 (CH), 126.8 (CH), 123.4 (CH), 120.8 (d, *J* = 9.0 Hz, CH), 115.1 (d, *J* = 23.5 Hz, CH), 108.9 (d, *J* = 25.5 Hz, CH), 63.8 (d, *J* = 2.0 Hz, C), 60.3 (CH), 39.8 (CH_2), 37.3 (CH_2), 28.9 (CH_2), 16.4 (CH_3); ^{19}F NMR (376 MHz, $CDCl_3$) δ −115.90 (ddd, *J* = 8.5, 8.5, 4.5 Hz); HRMS (ESI) *m/z* calcd for $C_{20}H_{17}FN_2O$ $(M + H)^+$ 321.1398, found 321.1384 (+4.3 ppm error).

(1'*R,10b'*R**)-2,4,6-Trimethyl-5',6'-dihydro-2'*H*-spiro[indole-3,1'-pyrrolo[2,1-*a*]isoquinolin]-3'(10b'*H*)-one (8d) and (1'*R**,10b'*S**)-2,4,6-trimethyl-5',6'-dihydro-2'*H*-spiro[indole-3,1'-pyrrolo[2,1-*a*]isoquinolin]-3'(10b'*H*)-one (9d)**

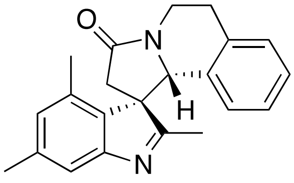

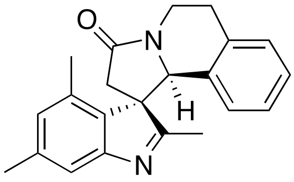

8d **9d**

General procedure A was followed using imine **6a** (100 mg, 0.762 mmol), acid **5d** (197 mg, 0.907 mmol), DIPEA (245 μL, 1.41 mmol) and T3P (725 mg of a 50% w/v solution in THF, 1.14 mmol), in THF (4 mL) for 16 h at RT. The unpurified reaction mixture contained 9:1 mixture of **8d**:**9d** based on analysis by ^{1}H NMR spectroscopy. Purification by flash column chromatography with EtOAc as eluent sequentially furnished a mixture of **9d** with minor contaminants (24 mg, 10%) and **8d** (198 mg, 78%).

8d: Pale orange solid, mp 128–130 °C; R_f (EtOAc) 0.10; ν_{max} (thin film)/cm^{-1} 3055, 2923, 1688 (C=O), 1626, 1593, 1459, 1433, 1305, 1264, 851, 729; δ_H NMR (400 MHz, $CDCl_3$) 7.16 (s, 1H), 7.02–6.97 (m, 2H), 6.75–6.71 (m, 1H), 6.50 (s, 1H), 6.04 (d, *J* = 8.0 Hz, 1H), 5.21 (s, 1H), 4.59–4.54 (m, 1H), 3.23–3.08 (m, 2H), 2.99 (d, *J* = 17.5 Hz, 1H), 2.83–2.78 (m, 1H), 2.65 (d, *J* = 17.5 Hz, 1H), 2.52 (s, 3H), 2.24 (s, 3H), 1.83 (s, 3H); δ_C (100.6 MHz, $CDCl_3$) 182.5 (C=N), 170.6 (C=O), 154.9 (C), 138.3 (C), 136.1 (C), 133.2 (C), 132.8 (C), 132.7 (C), 128.9 (CH), 128.8 (CH), 127.1 (CH), 126.2 (CH), 125.0 (CH), 118.7 (CH), 61.3 (C), 59.7 (CH), 37.9 (2 x CH_2), 27.6 (CH_2), 21.1 (CH_3), 19.2 (CH_3), 16.0 (CH_3); HRMS (ESI) *m/z* calcd for $C_{22}H_{22}N_2O$ $(M + H)^+$ 331.1805, found 331.1803 (+0.6 ppm error).

(1'*R,10b'*R**)-5-Bromo-2-methyl-5',6'-dihydro-2'*H*-spiro[indole-3,1'-pyrrolo[2,1-*a*]isoquinolin]-3'(10b'*H*)-one (8e) and (1'*R**,10b'*S**)-5-bromo-2-methyl-5',6'-dihydro-2'*H*-spiro[indole-3,1'-pyrrolo[2,1-*a*]isoquinolin]-3'(10b'*H*)-one (9e)**

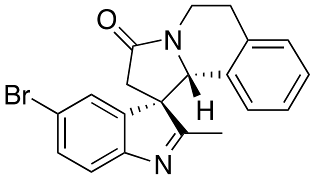

8e

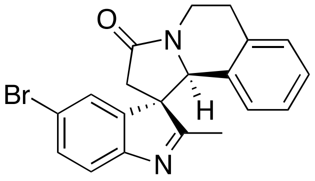

9e

General procedure A was followed using imine **6a** (100 mg, 0.762 mmol), acid **5e** (243 mg, 0.906 mmol), DIPEA (245 μL, 1.41 mmol) and T3P (725 mg of a 50% w/v solution in THF, 1.14 mmol), in THF (4 mL) for 16 h at RT. The unpurified reaction mixture contained 12:1 mixture of **8e**:**9e** based on analysis by ^{1}H NMR spectroscopy. Purification by flash column chromatography with EtOAc as eluent sequentially furnished **9e** (28 mg, 9%) and **8e** (250 mg, 87%). Total combined yield of **8e** and **9e**: 278 mg, 96%. **9e** was recrystallised from EtOAc and an X-ray crystal structure obtained.

9e: Orange oil; R_f(EtOAc) 0.25; ν_{max} (thin film)/cm^{-1} 2926, 2867, 1696 (C=O), 1604, 1578, 1455, 1423, 1305, 1186, 735; δ_H (400 MHz, $CDCl_3$) 7.67 (d, *J* = 2.0 Hz, 1H), 7.58 (dd, *J* = 8.0, 2.0 Hz, 1H), 7.45 (d, *J* = 8.0 Hz, 1H), 7.14–7.11 (m, 2H), 6.93–6.89 (m, 1H), 5.96 (d, *J* = 8.0 Hz, 1H), 5.34 (s, 1H), 4.57–4.52 (m, 1H), 3.15–3.02 (m, 3H), 2.89–2.85 (m, 1H), 2.58 (d, *J* = 17.0 Hz, 1H), 1.83 (s, 3H); δ_C NMR (100.6 MHz, $CDCl_3$) 183.0 (C=N), 169.6 (C=O), 153.8 (C), 142.4 (C), 133.1 (C), 132.7 (CH), 131.7 (C), 129.4 (CH), 127.9 (CH), 127.4 (CH), 125.1 (CH), 123.6 (CH), 122.0 (CH), 119.8 (C), 62.0 (C), 61.4 (CH), 40.0 (CH_2), 37.1 (CH_2), 28.4 (CH_2), 17.2 (CH_3); HRMS (ESI) *m/z* calcd for $C_{20}H_{17}BrN_2O$ $(M + H)^+$ 381.0597 found 381.0598 (−0.1 ppm error).

8e: Orange solid, mp 164–167 °C; R_f (EtOAc) 0.15; ν_{max} (thin film)/cm^{-1} 2918, 2852, 1695 (C=O), 1648, 1604, 1572, 1449, 1423, 1410, 1302, 1246, 1193, 803, 760, 739; δ_H (400 MHz, $CDCl_3$) 7.28–7.24 (m, 2H), 7.13–7.12 (m, 1H), 7.05–6.98 (m, 2H), 6.83 (dd, *J* = 7.5, 7.5 Hz, 1H), 6.33 (d, *J* = 8.0 Hz, 1H), 5.27 (s, 1H), 4.59–4.54 (m, 1H), 3.13–2.97 (m, 3H), 2.86–2.81 (m, 1H), 2.60 (s, 3H), 2.45 (d, *J* = 16.5 Hz, 1H); δ_C (100.6 MHz, $CDCl_3$) 180.8 (C=N), 170.0 (C=O), 152.7 (C), 142.3 (C), 133.3 (C), 131.5 (CH), 131.4 (C), 129.3 (CH), 127.5 (CH), 126.8 (CH), 124.3 (CH), 123.3 (CH), 121.4 (CH), 119.2 (C), 63.9 (C), 60.4

(CH), 39.7 (CH_2), 37.3 (CH_2), 28.9 (CH_2), 16.5 (CH_3); HRMS (ESI) *m/z* calcd for $C_{20}H_{17}BrN_2O$ (M + H)$^+$ 381.0597, found 381.0597 (−0.1 ppm error).

(1'*R,10b'*R**)-7-Bromo-2-methyl-5',6'-dihydro-2'*H*-spiro[indole-3,1'-pyrrolo[2,1-*a*]isoquinolin]-3'(10b'*H*)-one (8f) and (1'*R**,10b'*S**)-7-bromo-2-methyl-5',6'-dihydro-2'*H*-spiro[indole-3,1'-pyrrolo[2,1-*a*]isoquinolin]-3'(10b'*H*)-one (9f)**

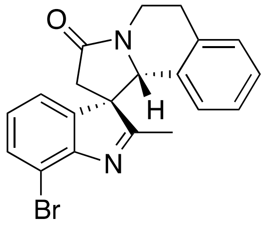

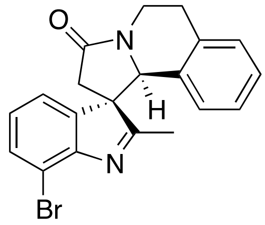

8f **9f**

General procedure A was followed using imine **6a** (100 mg, 0.762 mmol), acid **5f** (243 mg, 0.906 mmol), DIPEA (245 μL, 1.41 mmol) and T3P (725 mg of a 50% w/v solution in THF, 1.14 mmol), in THF (4 mL) for 16 h at RT**.** The unpurified reaction mixture contained 13:1 mixture of **8f**:**9f** based on analysis by ^{1}H NMR spectroscopy. Purification by flash column chromatography with EtOAc as eluent sequentially furnished **9f** (24 mg, 8%), a 1:2.3 mixture of **9f**:**8f** (11 mg, 4%) and **8f** (227 mg, 78%). Total combined yield of **8f** and **9f**: 262 mg, 90%.

9f: Orange oil, R$_f$ (4:1 EtOAc-hexane) 0.25; ν_{max} (thin film)/cm^{-1} 2933, 2861, 1690 (C=O), 1575, 1458, 1421, 1306, 1181, 783, 736; δ_H (400 MHz, $CDCl_3$) 7.61 (d, *J* = 8.0 Hz, 1H), 7.47 (d, *J* = 7.5 Hz, 1H), 7.26–7.22 (m, 1H), 7.15–7.10 (m, 2H), 6.94–6.90 (m, 1H), 5.95 (d, *J* = 8.0 Hz, 1H), 5.35 (s, 1H), 4.57-4.52 (m, 1H), 3.15–3.01 (m, 3H), 2.89–2.84 (m, 1H), 2.58 (d, *J* = 17.0 Hz, 1H), 1.89 (s, 3H); δ_C (100.6 MHz, $CDCl_3$) 184.0 (C=N), 169.6 (C=O), 153.1 (C), 142.0 (C), 133.0 (C), 132.6 (CH), 131.7 (C), 129.4 (CH), 127.9 (CH), 127.7 (CH), 127.6 (CH), 123.5 (CH), 120.6 (CH), 114.5 (C), 63.4 (C), 61.5 (CH), 40.2 (CH_2), 37.2 (CH_2), 28.5 (CH_2), 17.5 (CH_3); HRMS (ESI) *m/z* calcd for $C_{20}H_{17}{}^{79}BrN_2O$ (M + Na)$^+$ 403.0416 found 403.0400 (+4.1 ppm error).

8f: Orange solid, mp 190–192 °C; R$_f$ (4:1 EtOAc-hexane) 0.15; ν_{max} (thin film)/cm^{-1} 2940, 2858, 1692, 1571, 1456, 1431, 1417, 1302, 1195, 803, 760; δ_H (400 MHz, $CDCl_3$) 7.26 (d, *J* = 8.0 Hz, 1H), 6.99–6.93 (m, 2H), 6.90 (d, *J* = 7.5 Hz, 1H), 6.82 (dd, *J* = 7.5, 7.5 Hz, 1H), 6.73 (dd, *J* = 7.5, 7.5 Hz, 1H), 6.32 (d, *J* = 8.0 Hz, 1H), 5.28 (s, 1H), 4.55–4.51 (m, 1H), 3.11–2.95 (m, 3H), 2.82–2.78 (m, 1H), 2.65 (s, 3H), 2.41

(d, J = 16.5 Hz, 1H); δ_C (100.6 MHz, $CDCl_3$) 182.1 (C=N), 169.9 (C=O), 151.9 (C), 142.0 (C), 133.2 (C), 131.8 (CH), 131.6 (C), 129.1 (CH), 127.3 (CH), 126.9 (CH), 126.8 (CH), 123.4 (CH), 119.9 (CH), 113.9 (C), 64.9 (C), 60.3 (CH), 39.9 (CH_2), 37.3 (CH_2), 28.8 (CH_2), 16.7 (CH_3); HRMS (ESI) *m/z* calcd for $C_{20}H_{17}{}^{79}BrN_2O$ $(M + H)^+$ 381.0597, found 381.0597 (0.0 ppm error).

(1'*R,10b'*R**)-5',6'-Dihydro-2'*H*-spiro[indole-3,1'-pyrrolo[2,1-*a*]isoquinolin]-3'(10b'*H*)-one (8g) and (1'*R**,10b'*S**)-5',6'-dihydro-2'*H*-spiro[indole-3,1'-pyrrolo[2,1-*a*]isoquinolin]-3'(10b'*H*)-one (9g)**

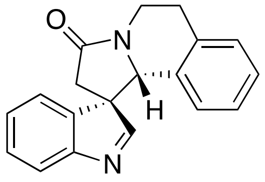

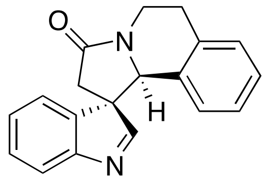

8g **9g**

General procedure A was followed using imine **6a** (100 mg, 0.762 mmol), acid **5g** (159 mg, 0.908 mmol), DIPEA (245 µL, 1.41 mmol) and T3P (725 mg of a 50% w/v solution in THF, 1.14 mmol), in THF (4 mL) for 16 h at RT. The unpurified reaction mixture contained 11:1 mixture of **8g**:**9g** based on analysis by ^1H NMR spectroscopy. Purification by flash column chromatography with EtOAc as eluent sequentially furnished a mixture of **9g** and a minor contaminant (18 mg, 8%) and **8g** (160 mg, 73%). Total combined yield of **8g** and **9g**: 178 mg, 81%.

9g: Pale yellow solid, mp 155–158 °C; R_f (EtOAc) 0.20; ν_{max} (thin film)/cm^{-1} 3026, 2925, 1687 (C=O), 1608, 1458, 1431, 1415, 1306, 923, 768, 730; δ_H (400 MHz, $CDCl_3$) 8.42 (s, 1H), 7.52 (d, J = 8.0 Hz, 1H), 7.18 (ddd, J = 7.5, 7.5, 1.5 Hz, 1H), 7.01–6.92 (m, 4H), 6.81–6.77 (m, 1H), 6.44 (d, J = 8.0 Hz, 1H), 5.50 (s, 1H), 4.58–4.54 (m, 1H), 3.32 (dd, J = 16.5, 1.5 Hz, 1H), 3.14–2.99 (m, 2H), 2.84–2.79 (m, 1H), 2.43 (d, J = 16.5 Hz, 1H); δ_C NMR (100.6 MHz, $CDCl_3$) 173.2 (C=N), 170.0 (C=O), 154.6 (C), 139.3 (C), 133.5 (C), 131.8 (C), 129.1 (CH), 128.6 (CH), 127.2 (CH), 126.7 (CH), 126.6 (CH), 123.7 (CH), 121.4 (CH), 121.2 (CH), 63.3 (C), 58.7 (CH), 37.9 (CH_2), 37.4 (CH_2), 29.1 (CH_2); HRMS (ESI) *m/z* calcd for $C_{19}H_{16}N_2O$ $(M + H)^+$ 289.1335, found 289.1333 (+0.9 ppm error).

(1'*R,10b'*S**)-2-Phenyl-5',6'-dihydro-2'*H*-spiro[indole-3,1'-pyrrolo[2,1-*a*]isoquinolin]-3'(10b'*H*)-one (9h) and (1'*R**,10b'*R**)-2-phenyl-5',6'-dihydro-2'*H*-spiro[indole-3,1'-pyrrolo[2,1-*a*]isoquinolin]-3'(10b'*H*)-one (8h)**

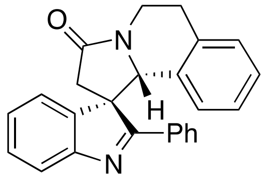

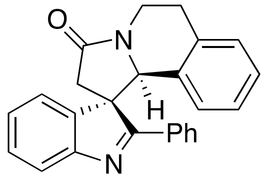

8h **9h**

General procedure A was followed using imine **6a** (53 mg, 0.404 mmol), acid **5h** (125 mg, 0.497 mmol), DIPEA (132 µL, 0.758 mmol) and T3P (394 mg of a 50% w/v solution in THF, 0.619 mmol), in THF (2 mL) for 16 h at RT. The unpurified reaction mixture contained 8:1 mixture of **9h**:**8h** based on analysis by ^{1}H NMR spectroscopy. Purification by flash column chromatography with EtOAc-hexane (1:1) as eluent sequentially furnished a 1:1.1 mixture of **8h**:**9h** (57 mg, 38%) and **9h** (49 mg, 33%). Total combined yield of **8h** and **9h**: 106 mg, 71%. **9h** was recrystallised from EtOAc and an X-ray crystal structure was obtained. CCDC 1436405 contains the supplementary crystallographic data for this paper. The data can be obtained free of charge from The Cambridge Crystallographic Data Centre via www.ccdc.cam.ac.uk/data_request/cif.

Diagnostic signals for **8h**: δ_H (400 MHz, $CDCl_3$) 6.23 (d, *J* = 8.0 Hz, 1H), 5.77 (s, 1H), 4.68-4.64 (m, 1H).

9h: Cream solid, mp 201–203 °C; R_f (EtOAc) 0.20; ν_{max} (thin film)/cm^{-1} 3062, 2931, 1689 (C=O), 1522, 1494, 1458, 1420, 1307, 910, 754, 694; δ_H (400 MHz, $CDCl_3$) 7.74 (d, *J* = 7.5 Hz, 1H), 7.59 (d, *J* = 7.0 Hz, 1H), 7.51 (ddd, *J* = 7.5, 7.5, 1.0 Hz, 1H), 7.45–7.41 (m, 1H), 7.31–7.27 (m, 1H), 7.16 (dd, *J* = 8.0, 8.0 Hz, 2H), 7.09–7.06 (m, 2H), 6.99 (dd, *J* = 7.5, 7.5 Hz, 1H), 6.87 (dd, *J* = 7.5, 7.5 Hz, 1H), 6.71 (d, *J* = 7.5 Hz, 1H), 5.98 (d, *J* = 8.0 Hz, 1H), 5.40 (s, 1H), 4.36–4.31 (m, 1H), 3.30 (dd, *J* = 17.5, 1.0 Hz, 1H), 3.21 (d, *J* = 17.5 Hz, 1H), 2.91 (ddd, *J* = 13.0, 13.0 Hz, 3.0 Hz, 1H), 2.36 (dd, *J* = 16.0, 3.0 Hz, 1H), 1.80 (ddd, *J* = 16.5, 13.0, 6.0 Hz, 1H); δ_C (100.6 MHz, $CDCl_3$) 181.0 (C=N), 170.7 (C=O), 154.4 (C), 141.5 (C), 134.7 (C), 134.1 (C), 131.6 (C), 130.1 (CH), 129.3 (CH), 128.5 (CH), 127.9 (CH), 127.5 (CH), 127.4 (CH), 127.0 (CH), 126.7 (CH), 123.6 (CH), 121.6 (CH), 121.4 (CH), 62.8 (C), 62.7 (CH), 40.9 (CH_2), 37.0 (CH_2), 27.6 (CH_2); HRMS (ESI) *m/z* calcd for $C_{25}H_{20}N_2O$ $(M + H)^+$ 365.1648, found 365.1646 (+0.6 ppm error).

(1'*R,11b'*R**)-2-Methyl-2',3',6',7'-Tetrahydrospiro[indole-3,1'-pyrido[2,1-*a*]isoquinolin]-4'(11b'*H*)-one (8i) and (1'*R**,11b'*S**)-2-methyl-2',3',6',7'-tetrahydrospiro[indole-3,1'-pyrido[2,1-*a*]isoquinolin]-4'(11b'*H*)-one (9i)**

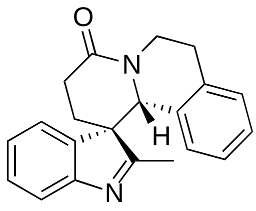

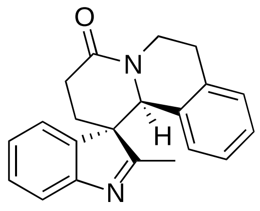

8i **9i**

General procedure A was followed using imine **6a** (100 mg, 0.762 mmol), acid **5i** (184 mg, 0.905 mmol), DIPEA (245 µL, 1.41 mmol) and T3P (725 mg of a 50% w/v solution in THF, 1.14 mmol), in THF (4 mL) for 16 h at RT. The unpurified reaction mixture contained 4:1 mixture of **8i**:**9i** based on analysis by ^{1}H NMR spectroscopy. Purification by flash column chromatography with EtOAc-MeOH (98:2) as eluent sequentially furnished a 4.8:1 mixture of **8i**:**9i** (96 mg, 40%) and **8i** (77 mg, 32%). Total combined yield of **8i** and **9i**: 173 mg, 72%.

8i: Yellow solid, mp 156–158 °C; R_f (98:2 EtOAc-MeOH) 0.20; ν_{max} (thin film)/cm^{-1} 2928, 2867, 1640 (C=O), 1577, 1455, 1432, 1410, 1247, 1047, 731; δ_H (400 MHz, $CDCl_3$) 7.35 (d, *J* = 7.5 Hz, 1H), 7.14 (ddd, *J* = 7.5, 7.5, 1.0 Hz, 1H), 7.09 (d, *J* = 7.5 Hz, 1H), 6.94 (dd, *J* = 8.0, 8.0 Hz, 1H), 6.90–6.86 (m, 2H), 6.82–6.78 (m, 1H), 6.54 (d, *J* = 8.0 Hz, 1H), 5.32 (s, 1H), 5.06–5.01 (m, 1H), 2.94–2.74 (m, 4H), 2.64–2.60 (m, 1H), 2.62 (s, 3H), 2.44 (ddd, *J* = 13.5, 9.5, 8.0 Hz, 1H), 1.46 (dd, *J* = 13.5, 5.0, 5.0 Hz, 1H); δ_C NMR (100.6 MHz, $CDCl_3$) 183.0 (C=N), 169.3 (C=O), 154.2 (C), 139.1 (C), 135.7 (C), 132.4 (C), 128.7 (CH), 128.3 (CH), 126.9 (CH), 126.0 (CH), 125.0 (CH), 123.6 (CH), 123.0 (CH), 120.3 (CH), 61.8 (C), 60.4 (CH), 39.7 (CH_2), 29.2 (2 x CH_2), 28.0 (CH_2), 17.1 (CH_3); HRMS (ESI) *m/z* calcd for $C_{21}H_{20}N_2O$ $(M + H)^+$ 317.1648 found 317.1646 (+1.1 ppm error).

Diagnostic peaks for **9i**: δ_H (400 MHz, $CDCl_3$) 5.81 (d, *J* = 8.0 Hz, 1H), 5.40 (s, 1H), 1.97 (s, 3H); HRMS (ESI) *m/z* calcd for $C_{21}H_{20}N_2O$ $(M + H)^+$ 317.1648, found 317.1645 (+0.8 ppm error).

(8) Imine scope in the DIA spirocyclisation reaction

(1'*R,10b'*R**)-8',9'-Dimethoxy-2-methyl-5',6'-dihydro-2'*H*-spiro[indole-3,1'-pyrrolo[2,1-*a*]isoquinolin]-3'(10b'*H*)-one (8j) and (1'*R**,10b'*S**)-8',9'-dimethoxy-2-methyl-5',6'-dihydro-2'*H*-spiro[indole-3,1'-pyrrolo[2,1-*a*]isoquinolin]-3'(10b'*H*)-one (9j)**

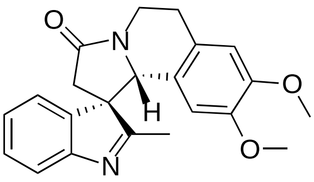

8j

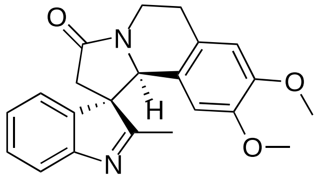

9j

General procedure A was followed using imine **6b** (145 mg, 0.762 mmol), acid **5a** (172 mg, 0.909 mmol), DIPEA (245 µL, 1.41 mmol) and T3P (725 mg of a 50% w/v solution in THF, 1.14 mmol), in THF (4 mL) for 16 h at RT**.** The unpurified reaction mixture contained 3:1 mixture of **8j**:**9j** based on analysis by ^{1}H NMR spectroscopy. Purification by flash column chromatography with EtOAc-MeOH (98:2) as eluent sequentially furnished **9j** (44 mg, 16%), a 1:3 mixture of **9j**:**8j** and **8j** (143 mg, 52%). Total combined yield of **8j** and **9j**: 202 mg, 72%.

9j: Yellow oil, R_f(EtOAc) 0.25; ν_{max} (thin film)/cm^{-1} 2932, 2853, 1692 (C=O), 1518, 1457, 1433, 1256, 1027, 800; δ_H (400 MHz, $CDCl_3$) 7.58–7.53 (m, 2H), 7.43 (ddd, *J* = 7.5, 7.5, 1.5 Hz, 1H), 7.37 (ddd, *J* = 7.5, 7.5, 1.0 Hz, 1H), 6.54 (s, 1H), 5.34 (s, 1H), 5.27 (s, 1H), 4.56–4.51 (m, 1H), 3.80 (s, 3H), 3.24 (s, 3H), 3.13–2.91 (m, 3H), 2.79–2.74 (m, 1H), 2.59 (d, *J* = 17.0 Hz, 1H), 1.90 (s, 3H); δ_C (100.6 MHz, $CDCl_3$) 182.9 (C=N), 170.1 (C=O), 155.0 (C), 148.3 (C), 147.3 (C), 140.0 (C), 129.2 (CH), 126.3 (CH), 124.9 (C), 123.5 (C), 121.8 (CH), 120.4 (CH), 111.3 (CH), 105.7 (CH), 61.9 (C), 61.8 (CH), 55.8 (CH_3), 55.0 (CH_3), 39.7 (CH_2), 37.1 (CH_2), 27.9 (CH_2), 17.4 (CH_3); HRMS (ESI) *m/z* calcd for $C_{22}H_{22}N_2O_3$ (M + H)$^+$ 363.1703, found 363.1688 (+4.3 ppm error).

8j: Yellow solid, mp 163–165 °C; R_f (EtOAc) 0.15; ν_{max} (thin film)/cm^{-1} 2933, 2835, 1693 (C=O), 1518, 1457, 1256, 1124, 770, 730; δ_H (400 MHz, $CDCl_3$) 7.42 (d, *J* = 7.5 Hz, 1H), 7.17–7.13 (m, 1H), 6.94–6.87 (m, 2H), 6.43 (s, 1H), 5.72 (s, 1H), 5.21 (s, 1H), 4.53 (dd, *J* = 13.5, 5.0 Hz, 1H), 3.73 (s, 3H), 3.48 (s, 3H), 3.12–3.08 (m, 2H), 2.95 (ddd, *J* = 13.5, 13.5, 5.0 Hz, 1H), 2.74–2.69 (m, 1H), 2.62 (s, 3H), 2.47 (d, *J* = 16.5 Hz, 1H); δ_C (100.6 MHz, $CDCl_3$) 181.0 (C=N), 170.4 (C=O), 153.8 (C), 147.7 (C), 147.6 (C), 140.5 (C), 128.6 (CH), 125.8 (CH), 125.5 (C), 124.0 (C), 121.4 (CH), 120.1 (CH), 111.1 (CH), 105.9

(CH), 63.1 (C), 60.2 (CH), 55.6 (CH_3 x 2), 39.8 (CH_2), 37.5 (CH_2), 28.3 (CH_2), 16.4 (CH_3); HRMS (ESI) *m/z* calcd for $C_{22}H_{22}N_2O_3$ (M + H)$^+$ 363.1703, found 363.1694 (+2.5 ppm error).

(1'*R,10b'*S**)-7',10'-Dibromo-8',9'-dimethoxy-2-methyl-5',6'-dihydro-2'*H*-spiro[indole-3,1'-pyrrolo[2,1-*a*]isoquinolin]-3'(10b'*H*)-one (8k) and (1'*R**,10b'*R**)-7',10'-dibromo-8',9'-dimethoxy-2-methyl-5',6'-dihydro-2'*H*-spiro[indole-3,1'-pyrrolo[2,1-*a*]isoquinolin]-3'(10b'*H*)-one (9k)**

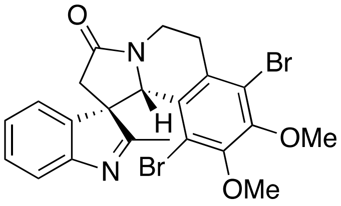

8k

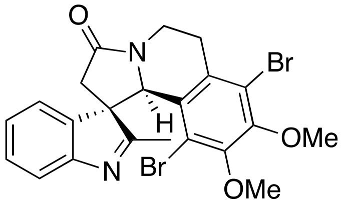

9k

General procedure A was followed using imine **6c** (210 mg, 0.602 mmol), acid **5a** (136 mg, 0.719 mmol), DIPEA (193 µL, 1.11 mmol) and T3P (572 mg of a 50% w/v solution in THF, 0.899 mmol), in THF (3.2 mL) for 1 h at 70 °C**.** The unpurified reaction mixture contained 9:1 mixture of **8k**:**9k** based on analysis by ^{1}H NMR spectroscopy. Purification by flash column chromatography with EtOAc as eluent sequentially furnished **9k** (27 mg, 8%) and **8k** (222 mg, 71%). Total combined yield of **8k** and **9k**: 249 mg, 79%.

9k: Yellow solid, mp 149–151 °C; R_f(EtOAc) 0.35; ν_{max} (thin film)/cm^{-1} 2927, 2854, 1698 (C=O), 1576, 1459, 1408, 1293, 1025, 979, 772; δ_H (400 MHz, $CDCl_3$) 7.53-7.51 (m, 1H), 7.46 (d, *J* = 7.5 Hz, 1H), 7.35 (ddd, *J* = 7.5, 7.5, 1.0 Hz, 1H), 7.29 (ddd, *J* = 7.5, 7.5, 1.0 Hz, 1H), 5.63 (s, 1H), 4.65–4.57 (m, 1H), 3.87 (s, 3H), 3.71 (s, 3H), 3.05–2.97 (m, 2H), 2.92–2.84 (m, 2H), 2.59 (d, *J* = 18.0 Hz, 1H), 1.66 (s, 3H); δ_C (100.6 MHz, $CDCl_3$) 180.5 (C=N), 170.7 (C=O), 154.3 (C), 150.4 (C), 150.1 (C), 144.4 (C), 134.0 (C), 131.3 (C), 128.6 (CH), 126.2 (CH), 121.5 (CH), 120.4 (CH), 120.2 (C), 117.1 (C), 63.4 (CH), 61.8 (C), 60.9 (CH_3), 60.7 (CH_3), 40.8 (CH_2), 37.4 (CH_2), 31.0 (CH_2), 16.7 (CH_3); HRMS (ESI) *m/z* calcd for $C_{22}H_{20}{}^{79}Br_2N_2O_3$ (M + H)$^+$ 518.9913, found 518.9928 (−2.7 ppm error).

8k: Yellow solid, mp 156–158 °C; R_f (EtOAc) 0.10; ν_{max} (thin film)/cm^{-1} 2936, 1693 (C=O), 1579, 1526, 1456, 1401, 1295, 1114, 1023, 756; δ_H (400 MHz, $CDCl_3$) 7.38 (d, *J* = 7.5 Hz, 1H), 7.11 (ddd, *J* = 7.5, 7.5, 1.0 Hz, 1H), 6.77 (dd, *J* = 7.5, 7.5 Hz, 1H), 6.36 (d, *J* = 7.5, 7.5 Hz, 1H), 5.47 (s, 1H), 4.61–4.51 (m, 1H), 3.73 (s, 3H), 3.41 (s, 3H), 3.01–2.84 (m, 4H), 2.60 (s, 3H), 2.36 (d, *J* = 17.5 Hz, 1H); δ_C (100.6 MHz,

CDCl3) 183.2 (C=N), 170.9 (C=O), 154.4 (C), 149.9 (C), 149.5 (C), 140.3 (C), 133.6 (C), 131.1 (C), 128.1 (CH), 124.9 (CH), 121.3 (CH), 120.2 (CH), 119.9 (C), 117.8 (C), 61.9 (C), 61.8 (CH), 60.7 (CH_3), 60.6 (CH_3), 40.0 (CH_2), 37.7 (CH_2), 30.9 (CH_2), 16.5 (CH_3); HRMS (ESI) *m/z* calcd for $C_{22}H_{20}{}^{79}Br_2N_2O_3$ $(M + H)^+$ 518.9913, found 518.9937 (−4.6 ppm error).

(3*R,9a'*S**)-2-Methyl-8',9a'-dihydro-4'*H*-spiro[indole-3,9'-thieno[3,2-*g*]indolizin]-7'(5'*H*)-one 8l and (3*S**,9a'*S**)-2-methyl-8',9a'-dihydro-4'*H*-spiro[indole-3,9'-thieno[3,2-*g*]indolizin]-7'(5'*H*)-one 9l**

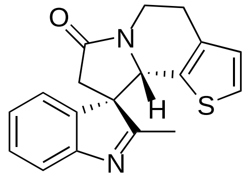

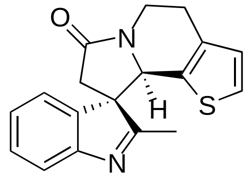

8l **9l**

General procedure A was followed using imine **6d** (105 mg, 0.762 mmol), acid **5a** (172 mg, 0.909 mmol), DIPEA (245 μL, 1.41 mmol) and T3P (725 mg of a 50% w/v solution in THF, 1.14 mmol), in THF (4 mL) for 16 h at RT**.** The unpurified reaction mixture contained 9:1 mixture of **8l**:**9l** based on analysis by ^{1}H NMR spectroscopy. Purification by flash column chromatography with EtOAc as eluent furnished an unknown mixture of diastereoisomers **8l** and **9l** (190 mg, 81%). Repeated chromatography furnished a small amount of separated diastereoisomers for characterisation purposes only.

8l: Yellow solid, mp 178–181 °C (from 2:3 hexane/EtOAc); R_f (EtOAc) 0.20; ν_{max} (thin film)/cm^{-1} 3081, 2969, 2933, 2900, 2839, 1695, 1684 (C=O), 1574, 1424, 1406, 1313, 1248, 887, 879, 759, 745; δ_H (400 MHz, $CDCl_3$) 7.48 (d, *J* = 7.5 Hz, 1H), 7.21 (ddd, *J* = 7.5, 7.5, 1.0 Hz, 1H), 7.02 (d, *J* = 7.5 Hz, 1H), 6.94 (ddd, *J* = 7.5, 7.5, 1.0 Hz, 1H), 6.90 (d, *J* = 5.0 Hz, 1H), 6.62 (d, *J* = 5.0 Hz, 1H), 5.33 (s, 1H), 4.65–4.57 (m, 1H), 3.14–3.03 (m, 1H), 3.13 (dd, *J* = 16.0, 1.5 Hz, 1H), 2.86–2.79 (m, 2H), 2.54 (s, 3H), 2.49 (d, *J* = 16.0 Hz, 1H); δ_C (100 MHz, $CDCl_3$) 179.1 (C=N), 170.5 (C=O), 154.1 (C), 139.5 (C), 134.4 (C), 130.0 (C), 128.6 (CH), 126.4 (CH), 125.5 (CH), 124.3 (CH), 121.2 (CH), 120.1 (CH), 63.1 (C), 59.9 (CH), 39.6 (CH_2), 37.3 (CH_2), 25.4 (CH_2), 16.3 (CH_3); HRMS (ESI) *m/z* calcd for $C_{18}H_{16}N_2OS$ $(M + H)^+$ 309.1056, found 309.1047 (+2.8 ppm error).

9l: Yellow oil, R_f (EtOAc) 0.25; ν_{max} (thin film)/cm^{-1}; δ_H (400 MHz, $CDCl_3$) 7.63–7.58 (m, 1H), 7.51–7.44 (m, 2H), 7.39–7.32 (m, 1H), 7.03 (d, *J* = 5.0 Hz, 1H), 6.77 (d, *J* = 5.0 Hz, 1H), 5.36 (s, 1H), 4.65–4.57 (m, 1H), 3.14–3.04 (m, 1H), 3.12 (dd, *J* = 17.0, 1.0 Hz, 1H), 2.91–2.84 (m, 2H), 2.60 (d, *J* = 17.0 Hz,

1H), 1.97 (s, 3H); δC (100 MHz, $CDCl_3$) 181.8 (C=N), 170.3 (C=O), 155.2 (C), 137.7 (C), 134.4 (C), 129.5 (CH), 129.1 (C), 126.6 (CH), 126.2 (CH), 124.9 (CH), 121.6 (CH), 120.6 (CH), 61.6 (CH), 39.5 (CH_2), 37.1 (CH_2), 25.2 (CH_2), 17.5 (CH_3); HRMS (ESI): *m/z* calcd for $C_{18}H_{16}N_2OS$ $(M + H)^+$ 309.1056, found 309.1042 (+4.4 ppm error).

(3*R,9a'*R**)-2,3'-Dimethyl-4',5',8',9a'-tetrahydrospiro[indole-3,9'-pyrrolo[2,3-*g*]indolizin]-7'(3'*H*)-one (8m) and (3*R**,9a'*S**)-2,3'-dimethyl-4',5',8',9a'-tetrahydrospiro[indole-3,9'-pyrrolo[2,3-*g*]indolizin]-7'(3'*H*)-one (9m)**

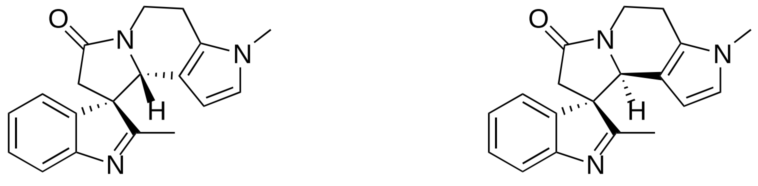

8m **9m**

General procedure A was followed using imine **6e** (105 mg, 0.762 mmol), acid **5a** (172 mg, 0.909 mmol), DIPEA (245 μL, 1.41 mmol) and T3P (725 mg of a 50% w/v solution in THF, 1.14 mmol), in THF (4 mL) for 16 h at RT**.** The unpurified reaction mixture contained 5:1 mixture of **8m**:**9m** based on analysis by ^{1}H NMR spectroscopy. Purification by flash column chromatography with EtOAc then EtOAc-MeOH (95:5) as eluent furnished a 3.5:1 mixture of **8m** and **9m** (190 mg, 81%) as a yellow solid. Recrystallization from EtOAc enabled isolation of the major diastereoisomer as a yellow solid.

8m: Yellow solid, mp 168–170 °C (EtOAc) R_f (ethyl acetate) 0.15; ν_{max} (thin film)/cm^{-1} 3385, 2916, 2847, 1688 (C=O), 1577, 1422, 1299, 1218, 751, 709; δH (400 MHz, $CDCl_3$) 7.57 (d, *J* = 8.0 Hz, 1H), 7.47 (d, *J* = 7.0 Hz, 1H), 7.45–7.38 (m, 1H), 7.31 (dd, *J* = 7.0, 7.0 Hz, 1H), 6.34 (d, *J* = 3.0 Hz, 1H), 5.21 (s, 1H), 4.95 (d, *J* = 3.0 Hz, 1H), 4.68–4.60 (m, 1H), 3.47 (s, 3H), 3.15–3.04 (m, 1H), 3.08 (d, *J* = 17.0 Hz, 1H), 2.84–2.66 (m, 2H), 2.57 (d, *J* = 17.0 Hz, 1H), 1.89 (s, 3H); δC (100 MHz, $CDCl_3$) 183.2 (C=N), 170.7 (C=O), 154.9 (C), 139.4 (C), 128.8 (CH), 125.8 (CH), 124.8 (C), 122.1 (CH), 121.6 (CH), 120.2 (CH), 113.2 (C), 102.3 (CH), 61.2 (C), 60.7 (CH), 39.6 (CH_2), 36.8 (CH_2), 33.0 (CH_3), 21.2 (CH_2), 17.7 (CH_3); HRMS (ESI): *m/z* calcd for $C_{19}H_{19}N_3O$ $(M + H)^+$ 306.1601, found 306.1612 (−3.5 ppm error).

Diagnostic signals for **9m**: δH (400 MHz, $CDCl_3$) 7.46 (d, *J* = 8.0 Hz, 1H), 7.23–7.16 (m, 1H), 6.97–6.89 (m, 2H), 6.16 (d, *J* = 3.0 Hz, 1H), 5.17 (s, 1H), 5.15 (d, *J* = 3.0 Hz, 1H), 4.68–4.60 (m, 1H), 3.37 (s, 3H),

3.15–3.04 (m, 1H), 3.08 (d, J = 16.0 Hz, 1H), 2.84–2.66 (m, 2H), 2.52 (s, 3H), 2.47 (d, J = 16.0 Hz, 1H); δ_C (100 MHz, $CDCl_3$) 180.4 (C=N), 171.0 (C=O), 153.9 (C), 140.6 (C), 127.9 (CH), 125.1 (C), 125.0 (CH), 122.0 (CH), 121.6 (CH), 119.7 (CH), 113.2 (C), 101.8 (CH), 62.7 (C), 59.1 (CH), 39.7 (CH_2), 37.1 (CH_2), 32.9 (CH_3), 21.3 (CH_2), 16.3 (CH_3).

(1'*R,8a'*R**)-8',8'-Dibenzyl-2-methyl-6',7',8',8a'-tetrahydro-2'*H*-spiro[indole-3,1'-indolizin]-3'(5'*H*)-one (8n) and (1'*R**,8a'*S**)-8',8'-dibenzyl-2-methyl-6',7',8',8a'-tetrahydro-2'*H*-spiro[indole-3,1'-indolizin]-3'(5'*H*)-one (9n)**

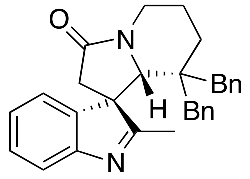

8n

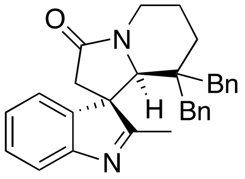

9n

General procedure A was followed using imine **6f** (60 mg, 0.228 mmol), acid **5a** (53 mg, 0.280 mmol), DIPEA (74 µL, 0.425 mmol) and T3P (219 mg of a 50% w/v solution in THF, 0.344 mmol), in toluene (1 mL) for 18 h at 90 °C**.** The unpurified reaction mixture contained 3:1 mixture of **8n**:**9n** based on analysis by ^{1}H NMR spectroscopy. Purification by flash column chromatography with EtOAc-Et_2O (10:90 then 20:80 then 50:50) as eluent sequentially furnished **9n** (26 mg, 26%) and **8n** (63 mg, 63%). Total combined yield of **8n** and **9n**: 89 mg, 89%. **8n** was recrystallised from EtOAc to obtain an X-ray crystal structure. CCDC 1436396 contains the supplementary crystallographic data for this paper The data can be obtained free of charge from The Cambridge Crystallographic Data Centre via www.ccdc.cam.ac.uk/data_request/cif.

9n: Yellow oil, R_f (EtOAc) 0.50; ν_{max} (thin film)/cm^{-1} 3029, 2932, 1686 (C=O), 1601, 1556, 1454, 1441, 1265, 732, 701; δ_H (400 MHz, $CDCl_3$) 7.69 (d, J = 7.5 Hz, 1H), 7.43 (dd, J = 7.5, 7.5 Hz, 1H), 7.35–7.20 (m, 7H), 7.02–6.98 (m, 1H), 6.90 (dd, J = 7.5, 7.5 Hz, 2H), 6.40 (d, J = 7.5 Hz, 2H), 4.39 (dd, J = 13.0, 5.0 Hz, 1H), 3.77 (s, 1H), 3.30 (d, J = 13.5 Hz, 1H), 3.00 (d, J = 13.5 Hz, 1H), 2.93–2.88 (m, 4H), 2.64 (ddd, J = 13.0, 13.0, 3.5 Hz, 1H), 2.49 (d, J = 14.0 Hz, 1H), 2.46 (d, J = 14.0 Hz, 1H), 2.39 (d, J = 16.5 Hz, 1H), 2.19–2.04 (m, 1H), 1.59–1.52 (m, 2H), 1.09–1.01 (m, 1H); δ_C (100.6 MHz, $CDCl_3$) 180.1 (C=N), 171.3 (C=O), 152.4 (C), 145.3 (C), 136.8 (C), 135.8 (C), 131.0 (CH), 130.8 (CH), 128.9 (CH), 128.3 (CH), 127.6 (CH), 126.82 (CH), 126.79 (CH), 126.2 (CH), 120.7 (CH), 120.4 (CH), 71.4 (CH), 61.2 (C),

42.5 (C), 40.9 (CH_2), 40.8 (CH_2), 39.7 (CH_2), 35.7 (CH_2), 31.0 (CH_2), 21.9 (CH_3), 20.0 (CH_2); HRMS (ESI) *m/z* calcd for $C_{30}H_{30}N_2O$ $(M + H)^+$ 435.2431, found 435.2445 (−3.3 ppm error).

8n: Brown solid, mp 225–227 °C; R_f (EtOAc) 0.35; ν_{max} (thin film)/cm^{-1} 3028, 2938, 1683 (C=O), 1589, 1454, 1422, 1283, 908, 728; δ_H (400 MHz, $CDCl_3$) 8.07 (d, *J* = 7.5 Hz, 1H), 7.71 (d, *J* = 7.5 Hz, 1H), 7.48 (dd, *J* = 7.5, 7.5 Hz, 1H), 7.40 (dd, *J* = 7.5, 7.5 Hz, 1H), 7.36–7.28 (m, 3H), 7.24-7.22 (m, 2H), 7.09–7.07 (m, 3H), 6.33–6.31 (m, 2H), 4.41 (dd, *J* = 13.0, 5.5 Hz, 1H), 3.47 (d, *J* = 13.5 Hz, 1H), 3.35–3.32 (m, 2H), 3.13 (d, *J* = 17.5 Hz, 1H), 2.54 (ddd, *J* = 13.0, 13.0, 3.5 Hz, 1H), 2.45 (d, *J* = 13.5 Hz, 1H), 2.33 (d, *J* = 17.5 Hz, 1H), 2.19 (s, 3H), 2.20–2.10 (m, 1H), 2.09 (d, *J* = 13.5 Hz, 1H), 1.56–1.50 (m, 2H), 1.24–1.17 (m, 1H); δ_C (100.6 MHz, $CDCl_3$) 185.1 (C=N), 170.6 (C=O), 156.1 (C), 137.6 (C), 136.6 (C), 135.9 (C), 130.84 (CH), 130.75 (CH), 129.5 (CH), 128.3 (CH), 127.8 (CH), 126.7 (CH), 126.7 (CH), 126.2 (CH), 125.9 (CH), 125.4 (CH), 121.2 (CH), 64.8 (CH), 60.0 (C), 43.3 (C), 40.4 (CH_2), 40.0 (CH_2), 38.7 (CH_2), 34.8 (CH_2), 31.1 (CH_2), 20.2 (CH_2), 16.0 (CH_3); HRMS (ESI) *m/z* calcd for $C_{30}H_{30}N_2O$ $(M + H)^+$ 435.2431, found 435.2447 (−3.8 ppm error).

(2'*R,3*R**)-2'-(4-Methoxyphenyl)-1',2-dimethylspiro[indole-3,3'-pyrrolidin]-5'-one (8o) and (2'*S**,3*R**)-2'-(4-methoxyphenyl)-1',2-dimethylspiro[indole-3,3'-pyrrolidin]-5'-one (9o)**

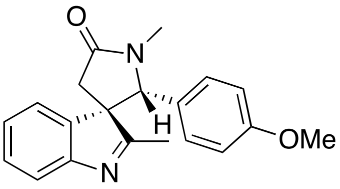

8o

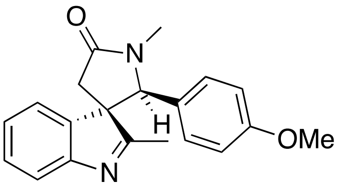

9o

General procedure A was followed using imine **6g** (113 mg, 0.757 mmol), acid **5a** (172 mg, 0.909 mmol), DIPEA (245 μL, 1.41 mmol) and T3P (725 mg of a 50% w/v solution in THF, 1.14 mmol), in THF (4 mL) for 1 h at 70 °C. The unpurified reaction mixture contained 4:1 mixture of **8o**:**9o** based on analysis by ^1H NMR spectroscopy. Purification by flash column chromatography with EtOAc as eluent sequentially furnished **9o** (32 mg, 13%) and **8o** (141 mg, 58%). Total combined yield of **8o** and **9o**: 173 mg, 71%. **8o** was recrystallised from EtOAc to obtain an X-ray crystal structure. CCDC 1436400 contains the supplementary crystallographic data for this paper The data can be obtained free of charge from The Cambridge Crystallographic Data Centre via www.ccdc.cam.ac.uk/data_request/cif.

9o: Orange oil, R_f (EtOAc) 0.40; ν_{max} (thin film)/cm^{-1} 2926, 1694 (C=O), 1612, 1573, 1513, 1459, 1396, 1250, 1176, 1031, 732; δ_H (400 MHz, $CDCl_3$) 7.51 (d, *J* = 7.5 Hz, 1H), 7.42–7.36 (m, 2H), 7.29–7.25 (m, 1H), 6.89–6.84 (m, 4H), 4.66 (s, 1H), 3.80 (s, 3H), 2.99–2.95 (m, 4H), 2.52 (d, *J* = 16.5 Hz, 1H), 1.62 (s, 3H); δ_C (100.6 MHz, $CDCl_3$) 181.4 (C=N), 173.3 (C=O), 160.0 (C), 143.8 (C), 128.8 (CH), 128.2 (C), 127.4 (CH), 127.1 (C), 126.3 (CH), 120.4 (CH), 120.2 (CH), 114.5 (CH), 70.4 (CH), 61.8 (C), 55.3 (CH_3), 36.8 (CH_2), 29.2 (CH_3), 17.7 (CH_3); HRMS (ESI) *m/z* calcd for $C_{20}H_{20}N_2O_2$ $(M + H)^+$ 321.1598, found 321.1594 (+1.0 ppm error).

8o: Orange solid, mp 166–168 °C; R_f (EtOAc) 0.20; ν_{max} (thin film)/cm^{-1} 2910, 1682 (C=O), 1608, 1583, 1511, 1455, 1248, 1171, 1023, 843, 774; δ_H (400 MHz, $CDCl_3$) 7.42 (d, *J* = 7.5 Hz, 1H), 7.18 (ddd, *J* = 7.5, 7.5, 1.0 Hz, 1H), 6.82–6.74 (m, 5H), 6.03 (d, *J* = 7.5 Hz, 1H), 4.40 (s, 1H), 3.78 (s, 3H), 2.97–2.92 (m, 4H), 2.44 (d, *J* = 17.0 Hz, 1H), 2.38 (s, 3H); δ_C (100.6 MHz, $CDCl_3$) 183.3 (C=N), 173.1 (C=O), 159.7 (C), 154.4 (C), 137.1 (C), 128.4 (CH), 128.1 (CH), 127.7 (C), 125.1 (CH), 123.8 (CH), 119.7 (CH), 114.0 (CH), 67.0 (CH), 61.0 (C), 55.3 (CH_3), 36.8 (CH_2), 29.0 (CH_3), 16.1 (CH_3); HRMS (ESI) *m/z* calcd for $C_{20}H_{20}N_2O_2$ $(M + H)^+$ 321.1598, found 321.1589 (+2.6 ppm error).

(2'*R,3*R**)-2'-(4-Methoxyphenyl)-1'-methyl-2-phenylspiro[indole-3,3'-pyrrolidin]-5'-one (8p) and (2'*S**,3*R**)-2'-(4-methoxyphenyl)-1'-methyl-2-phenylspiro[indole-3,3'-pyrrolidin]-5'-one (9p)**

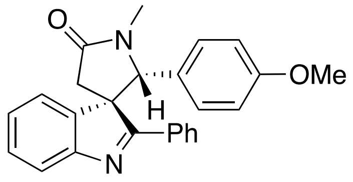

8p

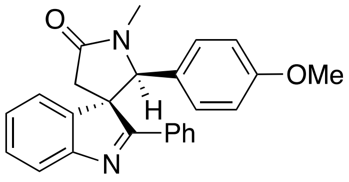

9p

General procedure A was followed using imine **6g** (45 mg, 0.302 mmol), acid **5h** (90 mg, 0.358 mmol), DIPEA (116 µL, 0.666 mmol) and T3P (286 mg of a 50% w/v solution in THF, 0.449 mmol), in THF (1.5 mL) for 1 h at 70 °C. The unpurified reaction mixture contained 1.8:1 mixture of **8p**:**9p** based on analysis by ^{1}H NMR spectroscopy. Purification by flash column chromatography with hexane-EtOAc (1:1) as eluent furnished an inseparable 1:1.2 mixture of diastereoisomers. Total yield of **8p** and **9p**: 73 mg, 76%.

8p and **9p**: Pale brown solid, mp 169–171 °C; R_f (1:1 hexane-EtOAc) 0.20; ν_{max} (thin film)/cm^{-1} 3060, 2932, 1693 (C=O), 1612, 1586, 1513, 1458, 1304, 1249, 1176, 1032, 911, 731; δ_H (400 MHz, $CDCl_3$) for

a 1.2:1 mixture of diastereoisomers 7.99–7.97 (m, 2.4H), 7.61 (m, 1H), 7.56–7.48 (m, 7.8H), 7.46–7.42 (m, 1.2H), 7.39–7.34 (m, 2H), 7.27 (dd, *J* = 7.5, 7.5 Hz, 2H), 7.20–7.16 (m, 1.2H), 6.95–6.91 (m, 1.2H), 6.71–6.64 (m, 5.8H), 6.44–6.40 (m, 2H), 6.35–6.32 (m, 2H), 3.70 (s, 3.6H), 3.64 (s, 3H), 3.44 (d, *J* = 18.0 Hz, 1H), 3.23 (d, *J* = 18.0 Hz, 1.2H), 3.06 (d, *J* = 18.0 Hz, 1H), 3.05 (d, *J* = 18.0 Hz, 1.2H), 2.92 (s, 3H), 2.84 (s, 3.6H); δ_H (100.6 MHz, $CDCl_3$) 179.6 (C=N), 179.0 (C=N), 174.4 (C=O), 173.8 (C=O), 159.4 (C), 159.3 (C), 153.6 (C), 153.4 (C), 140.6 (C), 134.6 (C), 132.4 (C), 131.1 (CH), 130.3 (CH), 129.0 (CH), 128.96 (CH), 128.3 (CH), 128.2 (CH), 128.1 (CH), 127.89 (CH), 127.88 (CH), 127.4 (CH), 127.3 (C), 126.9 (CH), 125.7 (CH), 125.4 (C), 123.5 (CH), 121.1 (CH), 120.8 (CH), 120.7 (CH), 113.6 (CH), 113.3 (CH), 71.8 (CH), 68.4 (CH), 61.7 (C), 61.1 (C), 55.2 (CH_3), 55.1 (CH_3), 39.4 (CH_2), 39.3 (CH_2), 29.7 (CH_3), 29.5 (CH_3); HRMS (ESI) *m/z* calcd for $C_{25}H_{22}N_2O_2$ $(M + H)^+$ 383.1754, found 383.1743 (+3.0 ppm error).

(1*S,10b*R**)-5'-Methoxy-2'-methyl-5,6-dihydro-2*H*-spiro[pyrrolo[2,1-*a*]isoquinoline-1,3'-pyrrolo[3,2-*b*]pyridin]-3(10b*H*)-one (11)**

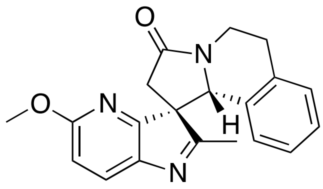

11

General procedure A was followed using imine **6a** (100 mg, 0.762 mmol), acid **10** (200 mg, 0.908 mmol), DIPEA (245 μL, 1.41 mmol) and T3P (725 mg of a 50% w/v solution in THF, 1.14 mmol), in THF (4 mL) for 16 h at RT. The unpurified reaction mixture contained a >20:1 mixture of **11** based on analysis by ^{1}H NMR spectroscopy. Purification by flash column chromatography with EtOAc as eluent furnished **11** (226 mg, 89%).

11: Yellow solid, mp 139–141 °C; R_f (EtOAc) 0.10; ν_{max} (thin film)/cm^{-1} 2939, 1687 (C=O), 1594, 1472, 1407, 1307, 1222, 1145, 1022, 909, 829, 645; δ_H (400 MHz, $CDCl_3$) 7.50 (d, *J* = 8.0 Hz, 1H), 6.96–6.92 (m, 2H), 6.82–6.78 (m, 1H), 6.39 (d, *J* = 8.5 Hz, 1H), 6.36 (d, *J* = 8.0 Hz, 1H) 5.30 (s, 1H), 4.58–4.54 (m, 1H), 3.70 (s, 3H), 3.20–3.11 (m, 1H), 3.06–2.99 (m, 2H), 2.74–2.69 (m, 1H), 2.59 (s, 3H), 2.46 (d, *J* = 16.0 Hz, 1H); δ_C (100.6 MHz, $CDCl_3$) 178.2 (C=N), 170.3 (C=O), 162.2 (C), 159.2 (C), 140.9 (C), 134.6 (C), 131.7 (C), 129.4 (CH), 128.8 (CH), 127.1 (CH), 126.5 (CH), 123.3 (CH), 109.5 (CH), 62.1 (C), 60.0

(CH), 53.5 (CH_3), 38.0 (CH_2), 37.6 (CH_2), 29.1 (CH_2), 16.8 (CH_3); HRMS (ESI) *m/z* calcd for $C_{20}H_{19}N_3O_2$ $(M + H)^+$ 334.1550, found 334.1550 (+0.1 ppm error).

(9) Other heterocycle scope in the DIA spirocyclisation reaction

(1'*R,10b'*R**)-3,5-Dimethyl-5',6'-dihydro-2'*H*-spiro[pyrrole-2,1'-pyrrolo[2,1-*a*]isoquinolin]-3'(10b'*H*)-one (*major*-14) and (1'*R**,10b'*S**)-3,5-dimethyl-5',6'-dihydro-2'*H*-spiro[pyrrole-2,1'-pyrrolo[2,1-*a*]isoquinolin]-3'(10b'*H*)-one (*minor*-14)**

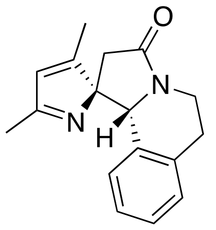

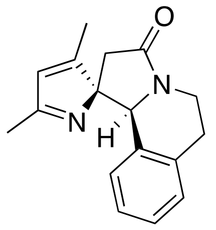

major*-14** ***minor-14

General procedure A was followed using imine **6a** (48 mg, 0.366 mmol), acid **12** (68 mg, 0.444 mmol), DIPEA (119 µL, 0.683 mmol) and T3P (353 mg of a 50% w/v solution in THF, 0.555 mmol), in $CHCl_3$ (2 mL) for 1 h at 70 °C. The unpurified reaction mixture contained a 14:1 mixture of **14** based on analysis by ^1H NMR spectroscopy. Purification by flash column chromatography with CH_2Cl_2-MeOH (95:5) as eluent furnished **14** as a 13:1 mixture (61 mg, 62%).

14: Brown oil, R_f (95:5 CH_2Cl_2-MeOH) 0.30; ν_{max} (thin film)/cm^{-1} 3052, 2921, 1687 (C=O), 1634, 1556, 1459, 1419, 1305, 1265, 931, 700 cm^{-1}; δ_H for ***major*-14** (400 MHz, $CDCl_3$) 7.15–7.02 (m, 3H), 6.63 (d, J = 8.0 Hz, 1H), 5.82 (d, J = 1.5 Hz, 1H), 5.45 (s, 1H), 4.52–4.43 (m, 1H), 3.33 (dd, J = 16.5, 1.0 Hz, 1H), 3.07–2.93 (m, 2H), 2.83–2.78 (m, 1H), 2.29 (s, 3H), 2.24 (d, J = 16.5 Hz, 1H), 1.58 (d, J = 1.5 Hz, 3H); δ_C for ***major*-14** (100.6 MHz, $CDCl_3$) 175.1 (C=N), 170.7 (C=O), 167.0 (C), 133.1 (C), 132.5 (C), 129.0 (CH), 127.1 (CH), 126.7 (2 x CH), 124.5 (CH), 85.2 (C), 59.3 (CH), 38.8 (CH_2), 36.7 (CH_2), 28.8 (CH_2), 18.9 (CH_3), 13.2 (CH_3); HRMS (ESI) *m/z* calcd for $C_{17}H_{18}N_2O$ $(M + H)^+$ 267.1492, found 267.1495 (−1.1 ppm error).

Diagnostic peaks for ***minor*-14**: δ_H (400 MHz, $CDCl_3$) 6.39 (d, J = 7.5 Hz, 1H), 6.14 (d, J = 1.5 Hz, 1H), 4.94 (s, 1H).

(1'*R,10b'*R**)-3-Methyl-5-phenyl-5',6'-dihydro-2'*H*-spiro[pyrrole-2,1'-pyrrolo[2,1-*a*]isoquinolin]-3'(10b'*H*)-one (*major*-15) and (1'*R**,10b'*S**)-3-methyl-5-phenyl-5',6'-dihydro-2'*H*-spiro[pyrrole-2,1'-pyrrolo[2,1-*a*]isoquinolin]-3'(10b'*H*)-one (*minor*-15)**

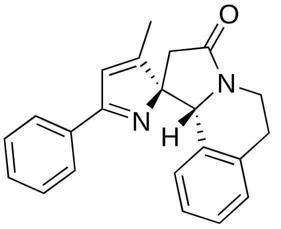

***major*-15**

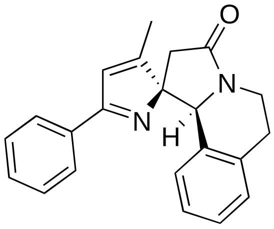

***minor*-15**

General procedure A was followed using imine **6a** (100 mg, 0.762 mmol), acid **12** (197 mg, 0.915 mmol), DIPEA (245 μL, 1.41 mmol) and T3P (725 mg of a 50% w/v solution in THF, 1.14 mmol), in $CHCl_3$ (4 mL) for 1 h at 70 °C. The unpurified reaction mixture contained a 5.6:1 mixture of **15** based on analysis by ^{1}H NMR spectroscopy. Purification by flash column chromatography with EtOAc as eluent furnished **15** as a 13:1 mixture (192 mg, 77%). Some ***major*-15** could be isolated by recrystallization (1:1 hexane-EtOAc) for characterisation. CCDC 1436465 contains the supplementary crystallographic data for this paper. The data can be obtained free of charge from The Cambridge Crystallographic Data Centre via www.ccdc.cam.ac.uk/data_request/cif.

15: Pale pink solid, mp 144–147 °C ($CDCl_3$); R_f (EtOAc) 0.20; ν_{max} (thin film)/cm^{-1} 2975, 2934, 2913, 2864, 1696 (C=O), 1627, 1424, 1409, 1361, 1310, 766, 747, 690, 667; δ_H (400 MHz, $CDCl_3$) 8.03–7.96 (m, 2H), 7.57–7.48 (m, 3H), 7.17–7.08 (m, 2H), 6.97 (ddd, *J* = 7.5, 7.5 , 2.2 Hz, 1H), 6.65 (d, *J* = 8.0 Hz, 1H), 6.44-6.43 (m, 1H), 5.63 (s, 1H), 4.61–4.49 (m, 1H), 3.51 (dd, *J* = 16.1, 1.0 Hz, 1H), 3.15–2.97 (m, 2H), 2.92–2.81 (m, 1H), 2.37 (d, *J* = 16.1 Hz, 1H), 1.71 (d, *J* = 2.0 Hz, 3H); δ_C (100 MHz, $CDCl_3$) 173.6 (C), 170.7 (C), 167.7 (C), 133.4 (C), 133.1 (C), 132.5 (C), 130.9 (CH), 128.9 (CH), 128.8 (CH), 127.7 (2 x CH), 127.1 (CH), 126.8 (CH), 124.6 (CH), 123.6 (CH), 123.5 (CH), 86.0 (C), 59.7 (CH), 39.2 (CH_2), 36.8 (CH_2), 28.8 (CH_2), 13.6 (CH_3); HRMS (ESI) *m/z* calcd for $C_{22}H_{20}N_2O$ $(M + H)^+$ 329.1648, found 329.1650 (−0.4 ppm error).

(1'*R,10b'*R**)-Ethyl 2,4-dimethyl-3'-oxo-3',5',6',10b'-tetrahydro-2'*H*-spiro[pyrrole-3,1'-pyrrolo[2,1-*a*]isoquinoline]-5-carboxylate *major*-17 and (1'*R**,10b'*S**)-ethyl 2,4-dimethyl-3'-oxo-3',5',6',10b'-tetrahydro-2'*H*-spiro[pyrrole-3,1'-pyrrolo[2,1-*a*]isoquinoline]-5-carboxylate *minor*-17**

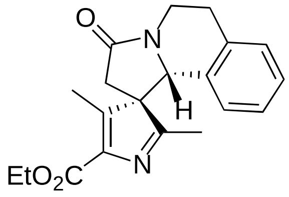

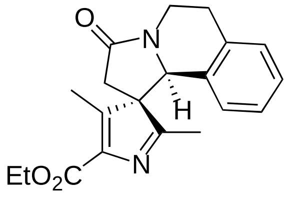

***major*-17** ***minor*-17**

General procedure A was followed using imine **6a** (34.0 mg, 0.259 mmol), acid **16** (70.0 mg, 0.311 mmol), DIPEA (83.5 µL, 0.479 mmol) and T3P (124 mg of a 50% w/v solution in THF, 0.388 mmol), in $CHCl_3$ (1.4 mL) for 1 h at 70 °C. The unpurified reaction mixture contained a 3.2:1 mixture of **17** based on analysis by ^{1}H NMR spectroscopy. Purification by flash column chromatography with EtOAc then EtOAc-MeOH (9.5:0.5) as eluent sequentially furnished ***minor*-17** (11 mg, 13%) and ***major*-17** (41 mg, 47%). Total combined yield of ***minor*-17** and ***major*-17**: 52 mg, 59%.

***Minor*-17:** Thin yellow film, R$_f$ (9.5:0.5 EtOAc/MeOH) 0.30; ν_{max} (thin film)/cm^{-1} 3304, 2927, 1660, 1633, 1436, 1269, 1208, 1174, 1088, 1025, 919, 773, 729; δ_H (400 MHz, $CDCl_3$) 7.21–7.11 (m, 2H), 7.11–7.05 (m, 1H), 6.47 (d, *J* = 7.5 Hz, 1H), 5.06 (s, 1H), 4.53–4.33 (m, 3H), 3.16–2.98 (m, 2H), 2.93–2.79 (m, 1H), 2.87 (d, *J* = 17.0 Hz, 1H), 2.48 (s, 3H), 2.30 (d, *J* = 17.0 Hz, 1H), 1.78 (s, 3H), 1.42 (t, *J* = 7.0 Hz, 3H); δ_C (100 MHz, $CDCl_3$) 182.3 (C), 169.5 (C), 163.1 (C), 147.6 (C), 140.9 (C), 133.1 (C), 132.0 (C), 129.3 (CH), 127.9 (CH), 127.8 (CH), 123.4 (CH), 67.7 (C), 61.1 (CH_2), 57.9 (CH), 37.3 (CH_2), 36.9 (CH_2), 28.3 (CH_2), 17.2 (CH_3), 14.3 (CH_3), 11.3 (CH_3); HRMS (ESI) *m/z* calcd for $C_{20}H_{22}N_2O_3$ $[M + H]^+$ 339.1703, found 339.1698 (+1.5 ppm error).

***Major*-17**: Yellow oil, R$_f$ (9.5:0.5 EtOAc/MeOH) 0.20; ν_{max} (thin film)/cm^{-1} 3301, 2980, 2930, 1655, 1435, 1270, 1213, 1174, 1092, 910, 728; δ_H (400 MHz, $CDCl_3$) 7.20–7.09 (m, 2H), 7.05 (dd, *J* = 7.0, 7.0 Hz, 1H), 6.43 (d, *J* = 7.5 Hz, 1H), 5.16 (s, 1H), 4.52–4.45 (m, 1H), 4.36–4.25 (m, 2H), 3.15–2.95 (m, 2H), 2.94 (dd, *J* = 17.0, 1.0 Hz, 1H), 2.89–2.80 (m, 1H), 2.50 (s, 3H), 2.36 (d, *J* = 17.0 Hz, 1H), 1.80 (s, 3H), 1.34 (t, *J* = 7.0 Hz, 3H); δ_C (100 MHz, $CDCl_3$) 180.06 (C), 169.5 (C), 163.0 (C), 151.2 (C), 140.1 (C), 133.1 (C), 131.5 (C), 129.2 (CH), 127.8 (CH), 127.4 (CH), 123.8 (CH), 67.4 (C), 60.8 (CH_2), 59.2 (CH), 37.6 (CH_2), 37.3 (CH_2), 28.3 (CH_2), 16.2 (CH_3), 14.3 (CH_3), 11.1 (CH_3); HRMS (ESI) *m/z* calcd for $C_{20}H_{22}N_2O_3$ $[M + H]^+$ 339.1703, found 339.1688 (+4.4 ppm error).

(10) Procedures for the modification of spirocycles 8a and 8g

(1'*S,2*S**,10b'*R**)-2-Methyl-5',6'-dihydro-2'*H*-spiro[indoline-3,1'-pyrrolo[2,1-*a*]isoquinolin]-3'(10b'*H*)-one (18)**

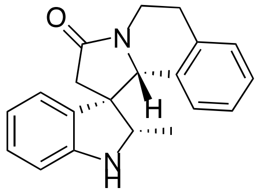

18

Using general procedure B, $NaBH_4$ (50 mg, 1.32 mmol) and spiroindolenine **8a** (100 mg, 0.331 mmol) in MeOH (5 mL) at reflux for 3.5 h gave the crude product. Purification by flash column chromatography using EtOAc-hexane (9:1) as eluent gave spiroindoline **18** (81 mg, 81%) as a cream solid, mp 229–231 °C; R_f (EtOAc) 0.50; ν_{max} (thin film)/cm^{-1} 3318 (NH), 3058, 2924, 1682 (C=O), 1606, 1578, 1459, 1305, 1143, 909, 727; δ_H (400 MHz, $CDCl_3$) 7.52 (d, *J* = 7.5 Hz, 1H), 6.97–6.89 (m, 3H), 6.83 (ddd, *J* = 7.5, 7.5, 1.0 Hz, 1H), 6.80–6.78 (m, 1H), 6.46 (d, *J* = 8.0 Hz, 1H), 6.43 (ddd, *J* = 7.5, 7.5, 1.0 Hz, 1H), 5.18 (s, 1H), 4.52–4.47 (m, 1H), 4.05 (br s, 1H), 4.01 (q, *J* = 7.0 Hz, 1H), 2.97–2.81 (m, 3H), 2.73 (dd, *J* = 16.5, 1.0 Hz, 1H), 2.69-2.65 (m, 1H), 1.64 (d, *J* = 7.0 Hz, 3H); δ_C (100.6 MHz, $CDCl_3$) 170.8 (C=O), 149.6 (C), 134.3 (C), 133.2 (C), 132.9 (C), 128.5 (CH), 128.2 (CH), 126.7 (CH), 126.4 (CH), 125.7 (CH), 122.4 (CH), 119.2 (CH), 109.4 (CH), 62.2 (CH), 61.8 (CH), 53.9 (C), 44.8 (CH_2), 37.3 (CH_2), 29.3 (CH_2), 15.7 (CH_3); HRMS (ESI) *m/z* calcd for $C_{20}H_{20}N_2O$ (M + H)$^+$ 305.1648, found 305.1645 (+1.1 ppm error). **18** was recrystallized from EtOAc and an X-ray crystal structure obtained. CCDC 1436401 contains the supplementary crystallographic data for this paper. The data can be obtained free of charge from The Cambridge Crystallographic Data Centre via www.ccdc.cam.ac.uk/data_request/cif.

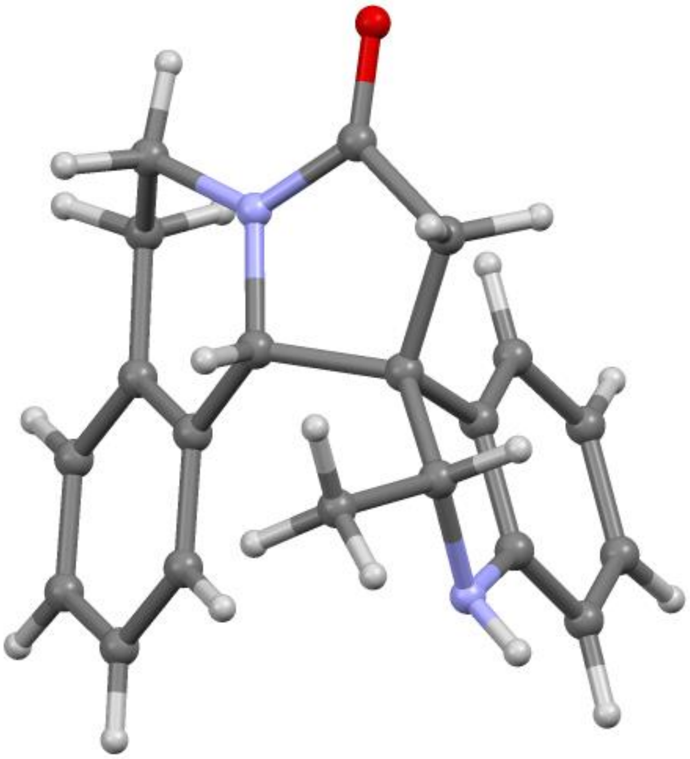

18: X-ray crystal structure

(1'*S,10b'*R**)-5',6'-Dihydro-2'*H*-spiro[indoline-3,1'-pyrrolo[2,1-*a*]isoquinolin]-3'(10b'*H*)-one (19)**

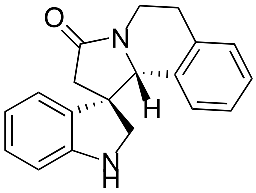

19

Using general procedure B, $NaBH_4$ (52 mg, 1.39 mmol) and spiroindolenine **8g** (100 mg, 0.347 mmol) in MeOH (5 mL) at reflux for 3.5 h gave the crude product. Purification by flash column chromatography using EtOAc as eluent gave spiroindoline 19 (87 mg, 87%) as a cream solid, mp 211–213 °C; R_f (EtOAc) 0.30; ν_{max} (thin film)/cm^{-1} 3330 (NH), 2924, 2855, 1675 (C=O), 1606, 1487, 1459, 1415, 1306, 909, 729; δ_H (400 MHz, $CDCl_3$) 7.33–7.31 (m, 1H), 7.01–6.93 (m, 3H), 6.85 (ddd, J = 7.5, 7.5, 1.0 Hz, 1H), 6.77–6.75 (m, 1H), 6.47 (d, J = 8.0 Hz, 1H), 6.44–6.40 (m, 1H), 5.04 (s, 1H), 4.51–4.47 (m, 1H), 4.09 (d, J = 10.0 Hz, 1H), 3.35 (d, J = 10.0 Hz, 1H), 2.98–2.84 (m, 4H), 2.71–2.67 (m, 1H); δ_C (100.6 MHz, $CDCl_3$) 171.3 (C=O), 150.6 (C), 134.1 (C), 132.9 (C), 131.5 (C), 128.7 (CH), 128.4 (CH), 126.7 (CH), 126.0

(CH), 125.6 (CH), 122.4 (CH), 118.8 (CH), 109.5 (CH), 68.2 (CH), 57.6 (CH_2), 52.4 (C), 46.2 (CH_2), 35.1 (CH_2), 29.2 (CH_2); HRMS (ESI) *m/z* calcd for $C_{19}H_{18}N_2O$ $(M + H)^+$ 291.1492, found 291.1486 (+1.9 ppm error).

(1'*S,2*S**,10b'*R**)-2-Methyl-3',5',6',10b'-tetrahydro-2'*H*-spiro[indoline-3,1'-pyrrolo[2,1-*a*]isoquinoline] (20)**

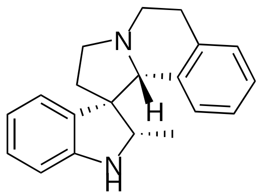

20

Using general procedure C, $LiAlH_4$ (76 mg, 2.00 mmol) and spiroindoline **18** (153 mg, 0.50 mmol) in dry THF (15 mL) under Ar at reflux for 2.5 h gave the crude product. Purification by flash column chromatography EtOAc as eluent gave spiroindoline **20** (110 mg, 75%) as a white solid, mp 141–143 °C; R_f (EtOAc) 0.45; ν_{max} (thin film)/cm^{-1} 3356 (NH), 2981, 2897, 2804, 1604, 1467, 1330, 935, 762, 556; δ_H (400 MHz, $CDCl_3$) 7.48 (d, *J* = 7.5 Hz, 1H), 7.02 (dd, *J* = 7.5, 1.0 Hz, 1H), 6.96–6.91 (m, 2H), 6.89–6.81 (m, 2H), 6.54 (d, *J* = 7.5 Hz, 1H), 6.49 (ddd, *J* = 7.5, 7.5, 1.0 Hz, 1H), 4.02 (q, *J* = 7.0 Hz, 1H), 3.64 (s, 1H), 3.30–3.22 (m, 2H), 3.14–3.06 (m, 1H), 2.67 (dd, *J* = 16.0, 4.0 Hz, 1H), 2.60–2.46 (m, 3H), 1.98–1.92 (m, 1H), 1.50 (d, *J* = 7.0 Hz, 3H); δ_C (100.6 MHz, $CDCl_3$) 150.0 (C), 136.3 (C), 136.1 (C), 135.3 (C), 128.1 (CH), 127.2 (CH), 127.1 (CH), 125.5 (CH), 125.2 (CH), 125.0 (CH), 118.1 (CH), 108.8 (CH), 69.4 (CH), 63.9 (CH), 56.7 (C), 52.2 (CH_2), 49.9 (CH_2), 41.6 (CH_2), 29.2 (CH_2), 16.1 (CH_3); HRMS (ESI) *m/z* calcd for $C_{20}H_{22}N_2$ $(M + H)^+$ 291.1856, found 291.1860 (−1.6 ppm error).

(1'*S,10b'*R**)-3',5',6',10b'-Tetrahydro-2'*H*-spiro[indoline-3,1'-pyrrolo[2,1-*a*]isoquinoline] (21)**

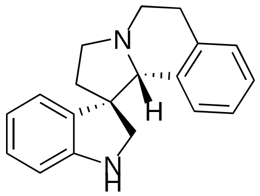

21

Using general procedure C, $LiAlH_4$ (78 mg, 2.04 mmol) and spiroindoline **19** (148 mg, 0.51 mmol) in dry THF (15 mL) under Ar at reflux for 2.5 h gave the crude product. Purification by flash column chromatography EtOAc as eluent gave spiroindoline **21** (94 mg, 67%) as a yellow oil, R_f (EtOAc) 0.10; ν_{max} (thin film)/cm^{-1} 3291 (NH), 2923, 2790, 1638, 1604, 1577, 1484, 1264, 1026, 731, 701; δ_H (400 MHz, $CDCl_3$) 7.04 (d, *J* = 8.0 Hz, 1H), 7.00–6.97 (m, 2H), 6.90 (ddd, *J* = 7.5, 7.5, 1.0 Hz, 1H), 6.83 (dd, *J* = 7.5, 7.5 Hz, 1H), 6.79–6.77 (m, 1H), 6.60 (d, *J* = 8.0 Hz, 1H), 6.47 (ddd, *J* = 7.5, 7.5, 1.0 Hz, 1H), 4.01 (d, *J* = 9.5 Hz, 1H), 3.71 (d, *J* = 9.5 Hz, 1H), 3.54 (s, 1H), 3.31–3.12 (m, 3H), 2.79–2.75 (m, 1H), 2.65–2.56 (m, 2H), 2.29 (ddd, *J* = 13.0, 9.5, 8.0 Hz, 1H), 2.17 (ddd, *J* = 13.0, 8.0, 2.5 Hz, 1H); δ_C (100.6 MHz, $CDCl_3$) 150.9 (C), 135.7 (C), 135.4 (C), 135.0 (C), 128.4 (CH), 127.2 (CH), 126.0 (CH), 125.7 (CH), 125.2 (CH), 125.0 (CH), 118.8 (CH), 109.1 (CH), 73.1 (CH), 60.7 (CH_2), 55.0 (C), 52.7 (CH_2), 49.4 (CH_2), 42.7 (CH_2), 29.4 (CH_2); HRMS (ESI) *m/z* calcd for $C_{19}H_{20}N_2$ $(M + H)^+$ 277.1699, found 277.1700 (−0.2 ppm error).

(1'*S,2*R**,10b'*R**)-2-Methyl-5',6'-dihydro-2'*H*-spiro[indoline-3,1'-pyrrolo[2,1-*a*]isoquinolin]-3'(10b'*H*)-one (23)**

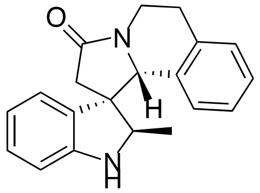

23

MeMgBr (3M in Et_2O, 0.59 mL, 1.77 mmol) was added to a stirred solution of spiroindolenine **8g** (170 mg, 0.59 mmol) in dry THF (7 mL) at 0 °C under Ar. The resulting solution was stirred at 0 °C for 6 h, then quenched with NH_4Cl (10 mL) and diluted with CH_2Cl_2 (10 mL). The two layers were separated and

the aqueous layer was extracted with CH_2Cl_2 (3 x 10 mL). The combined organics were dried ($MgSO_4$) and evaporated under reduced pressure to give the crude product. Purification by flash column chromatography using EtOAc-hexane (9:1) as eluent gave the spiroindoline **23** (112 mg, 62%) as an orange solid, mp 141-143 °C; R_f (EtOAc) 0.45; ν_{max} (thin film)/cm^{-1} 3319 (NH), 3051, 2926, 1676 (C=O), 1606, 1459, 1415, 1305, 1264, 730, 701; δ_H (400 MHz, $CDCl_3$) 7.38–7.36 (m, 1H), 7.02–6.95 (m, 2H), 6.93–6.91 (m, 1H), 6.83 (ddd, *J* = 7.5, 7.5, 1.0 Hz, 1H), 6.76 (d, *J* = 7.5 Hz, 1H), 6.43–6.39 (m, 2H), 4.94 (s, 1H), 4.49–4.45 (m, 1H), 4.32 (q, *J* = 6.5 Hz, 1H), 3.05 (dd, *J* = 16.0, 1.0 Hz, 1H), 2.96–2.82 (m, 2H), 2.71–2.64 (m, 1H), 2.52 (d, *J* = 16.0 Hz, 1H), 1.31 (d, *J* = 6.5 Hz, 3H); δ_C (100.6 MHz, $CDCl_3$) 171.8 (C=O), 148.9 (C), 134.6 (C), 132.8 (C), 130.7 (C), 128.5 (CH), 128.3 (CH), 126.6 (CH), 126.2 (CH), 125.7 (CH), 122.7 (CH), 118.4 (CH), 109.2 (CH), 67.6 (CH), 61.3 (CH), 56.7 (C), 40.4 (CH_2), 37.0 (CH_2), 29.3 (CH_2), 19.5 (CH_3); HRMS (ESI) *m/z* calcd for $C_{20}H_{20}N_2O$ $(M + H)^+$ 305.1648, found 305.1650 (−0.5 ppm error).

(1'*S,2*S**,10b'*R**)-2-(1*H*-Pyrrol-2-yl)-5',6'-dihydro-2'*H*-spiro[indoline-3,1'-pyrrolo[2,1-*a*]isoquinolin]-3'(10b'*H*)-one (22)**

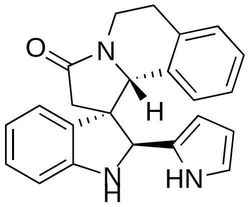

22

A flask was charged with spiroindolenine **8g** (100 mg, 0.35 mmol) and pyrrole (218 μL, 3.15 mmol). AcOH (2.5 mL) was added and the reaction was stirred for 16 h. Sat. $NaHCO_{3(aq)}$ (10 mL) and CH_2Cl_2 (10 mL) were carefully added to the reaction and the layers were separated. The aqueous layer was extracted with CH_2Cl_2 (2 x 10 mL) and the combined organics were dried ($MgSO_4$) and evaporated under reduced pressure to give the crude product. Purification by flash column chromatography on silica using EtOAc as eluent gave the spirocyclic product **22** (107 mg, 86%) as a yellow solid, mp 234–236 °C; R_f (EtOAc) 0.25; ν_{max} (thin film)/cm^{-1} 3326 (NH), 3180, 2927, 2851, 1655 (C=O), 1607, 1484, 1467, 1249, 969, 729; δ_H (400 MHz, $CDCl_3$) 10.89 (s, 1H), 7.32 (d, *J* = 7.5 Hz, 1H), 7.10–6.98 (m, 3H), 6.79 (ddd, *J* = 7.5, 7.5, 1.0 Hz, 1H), 6.75–6.73 (m, 1H), 6.46 (d, *J* = 7.5 Hz, 1H), 6.43 (d, *J* = 7.5 Hz, 1H), 6.26 (ddd, *J* = 7.5, 7.5, 1.0 Hz, 1H), 6.18 (d, *J* = 2.5 Hz, 1H), 6.07–6.05 (m, 1H), 6.03–6.02 (m, 1H), 5.42 (d, *J* = 2.5 Hz, 1H), 5.08 (s, 1H), 4.15 (ddd, *J* = 12.5, 5.0, 2.5 Hz, 1H), 3.00–2.93 (m, 1H), 2.89–2.74 (m, 2H), 2.31 (d, *J* = 16.5 Hz,

1H), 1.88 (d, *J* = 16.5 Hz, 1H); δ_C (100.6 MHz, $CDCl_3$) 170.1 (C=O), 150.2 (C), 134.9 (C), 134.0 (C), 131.3 (C), 130.6 (C), 128.9 (CH), 127.7 (CH), 126.3 (CH), 126.2 (CH), 125.8 (CH), 122.3 (CH), 117.6 (CH), 117.1 (CH), 108.4 (CH), 107.5 (CH), 106.3 (CH), 64.0 (C), 63.7 (NCH), 57.1 (C), 43.0 (CH_2), 36.5 (CH_2), 29.0 (CH_2); HRMS (ESI) *m/z* calcd for $C_{23}H_{21}N_3O$ $(M + H)^+$ 356.1757, found 356.1746 (3.3 ppm error).

(11) ^{1}H and ^{13}C Spectra

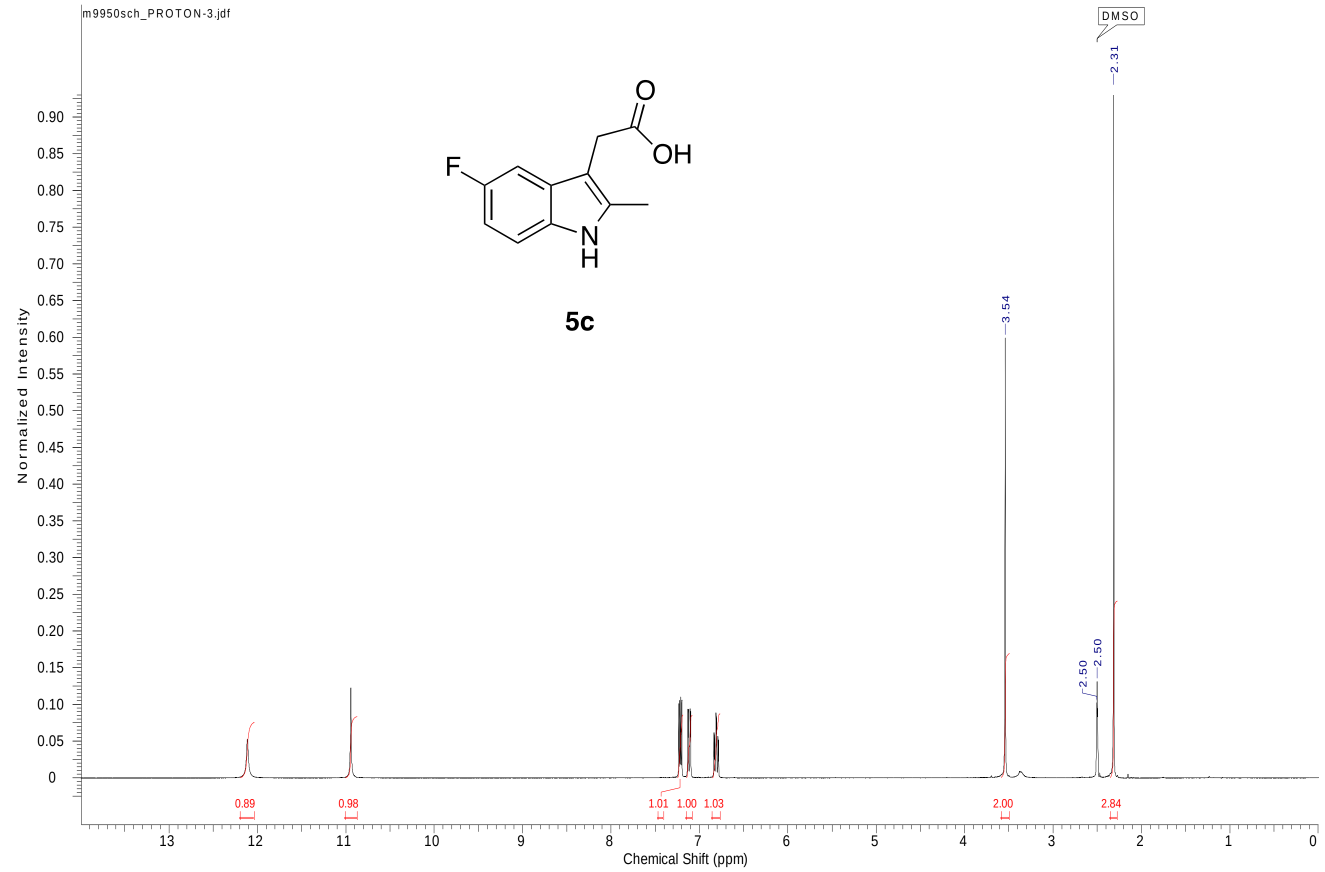

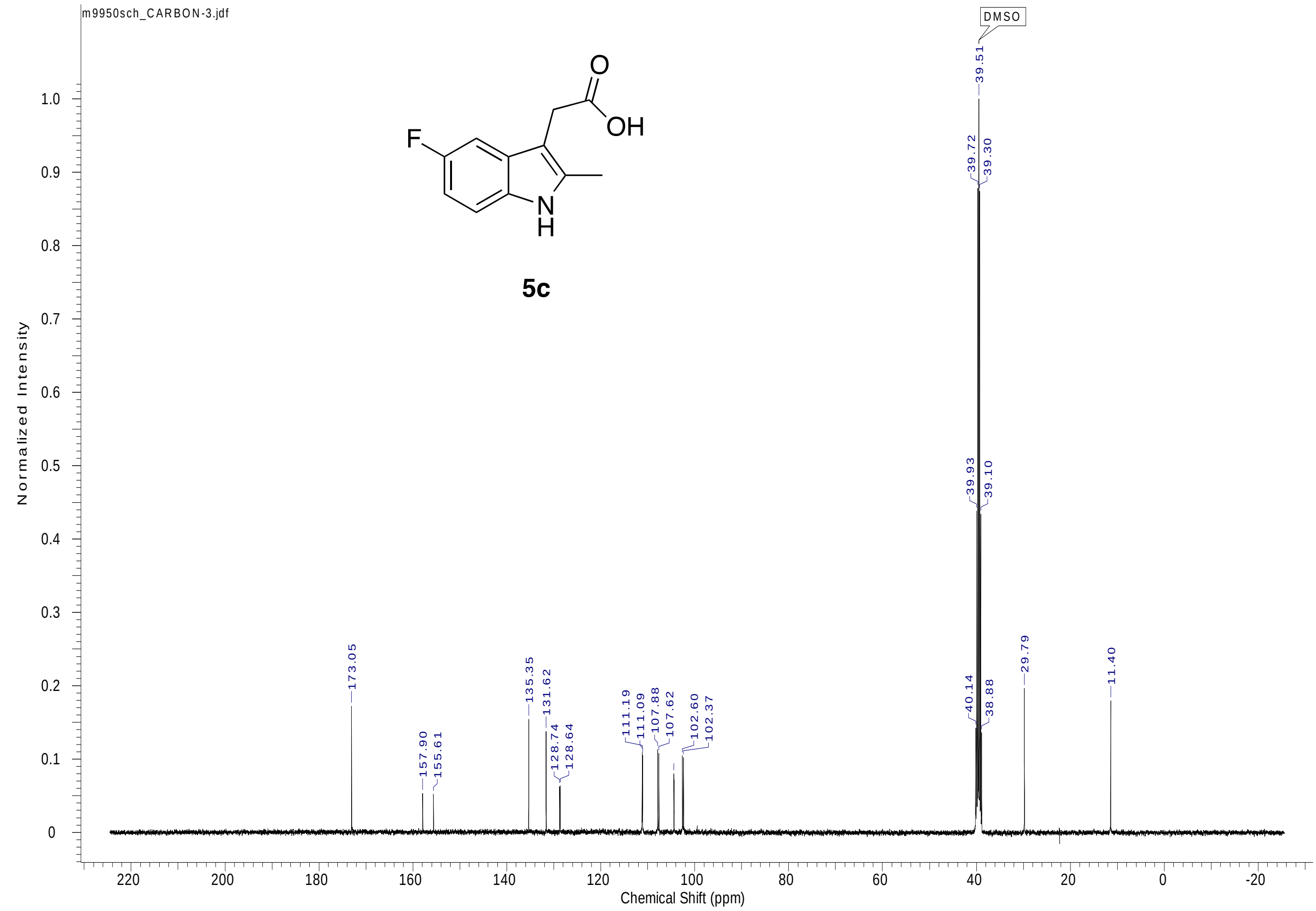

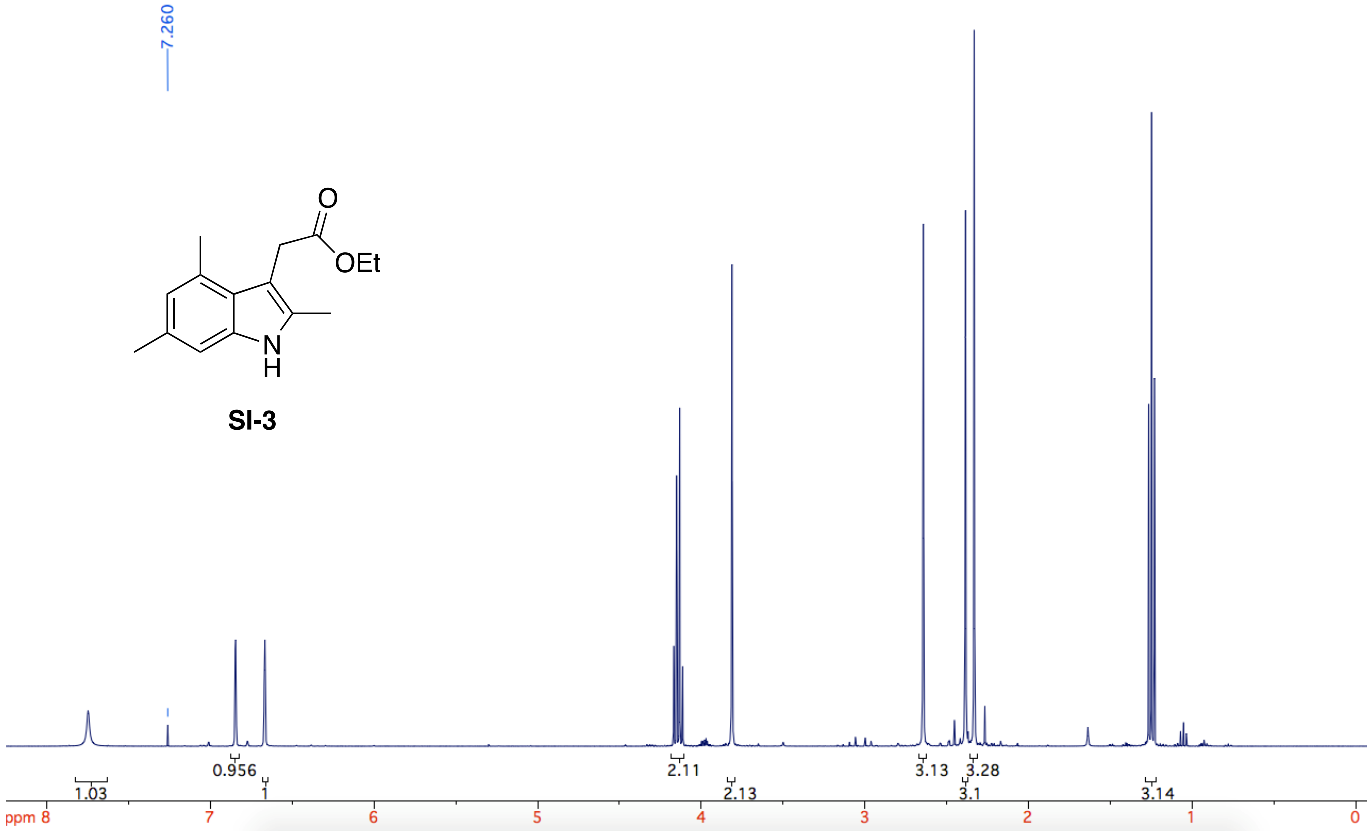
7.260
O
OEt
N
H
SI-3
1.03
0.956
1
2.11
2.13
3.13
3.1
3.28
3.14
ppm 8
7
6
5
4
3
2
1
0

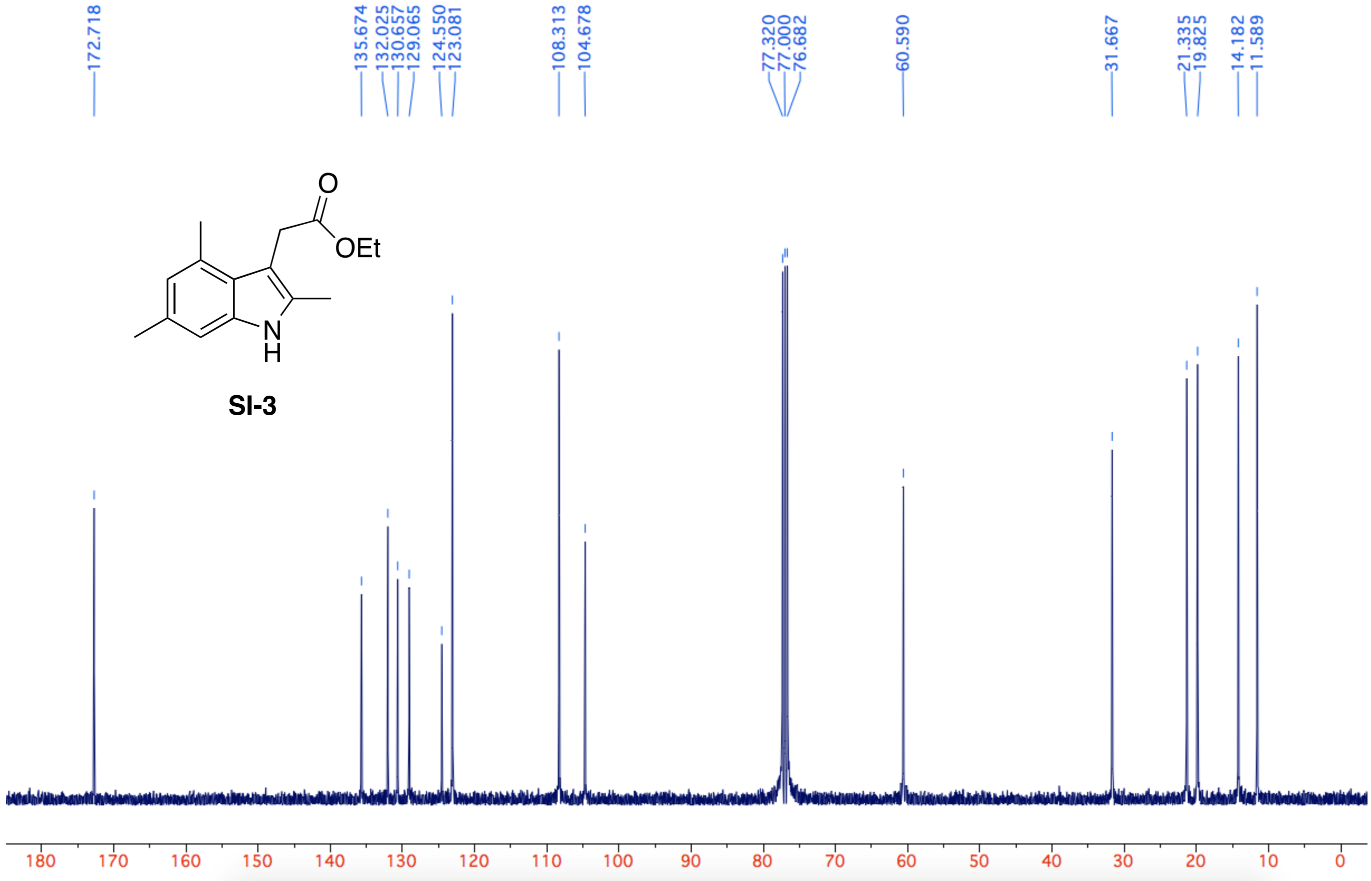
172.718
135.674
132.025
130.657
129.065
124.550
123.081
108.313
104.678
77.320
77.000
76.682
60.590
31.667
21.335
19.825
14.182
11.589
O
OEt
N
H
SI-3
180
170
160
150
140
130
120
110
100
90
80
70
60
50
40
30
20
10
0

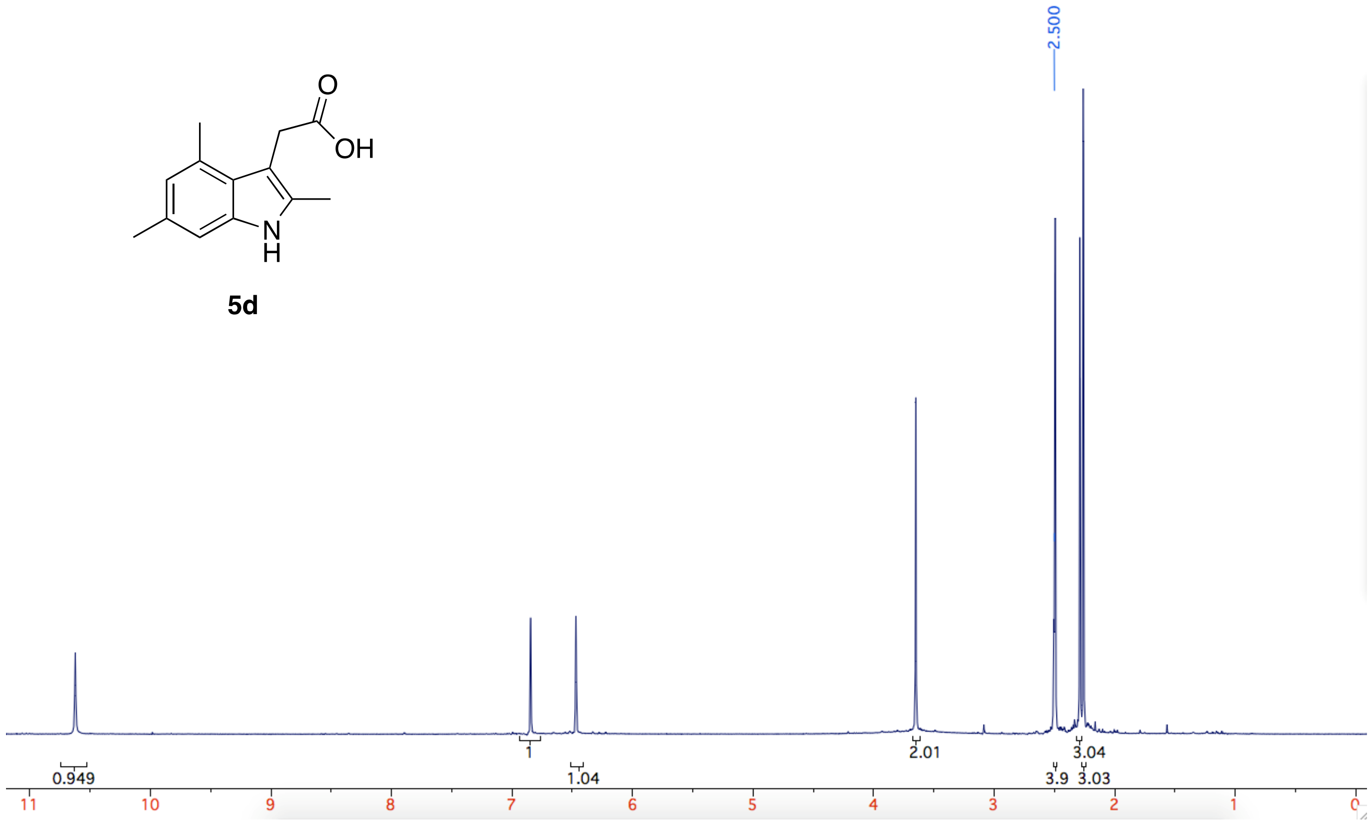

O
OH
N
H
5d
2.500
0.949
1
1.04
2.01
3.9
3.04
3.03
11
10
9
8
7
6
5
4
3
2
1
0

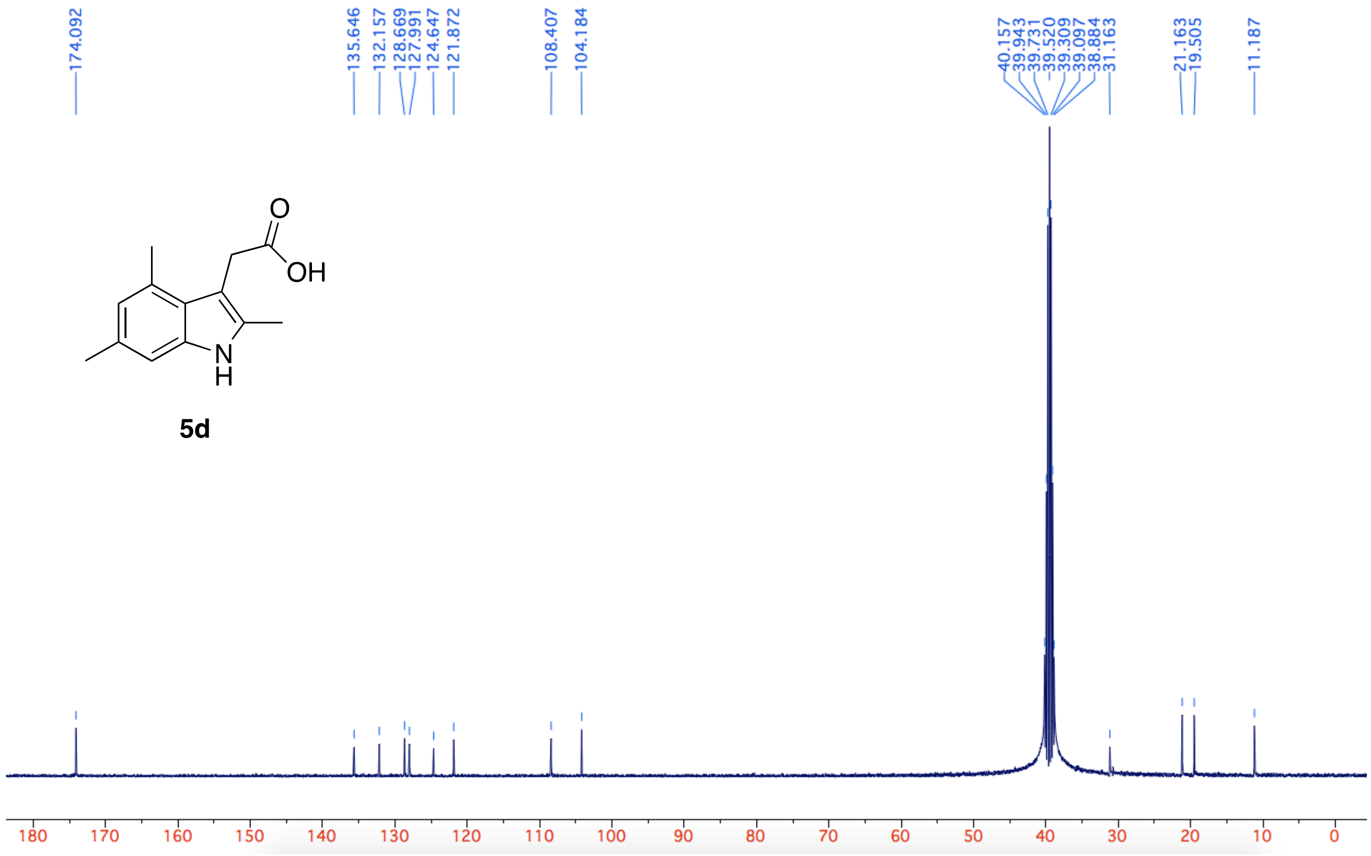

174.092
135.646
132.157
128.669
127.991
124.647
121.872
108.407
104.184
40.157
39.943
39.731
39.520
39.309
39.097
38.884
31.163
21.163
19.505
11.187
O
OH
N
H
5d
180
170
160
150
140
130
120
110
100
90
80
70
60
50
40
30
20
10
0

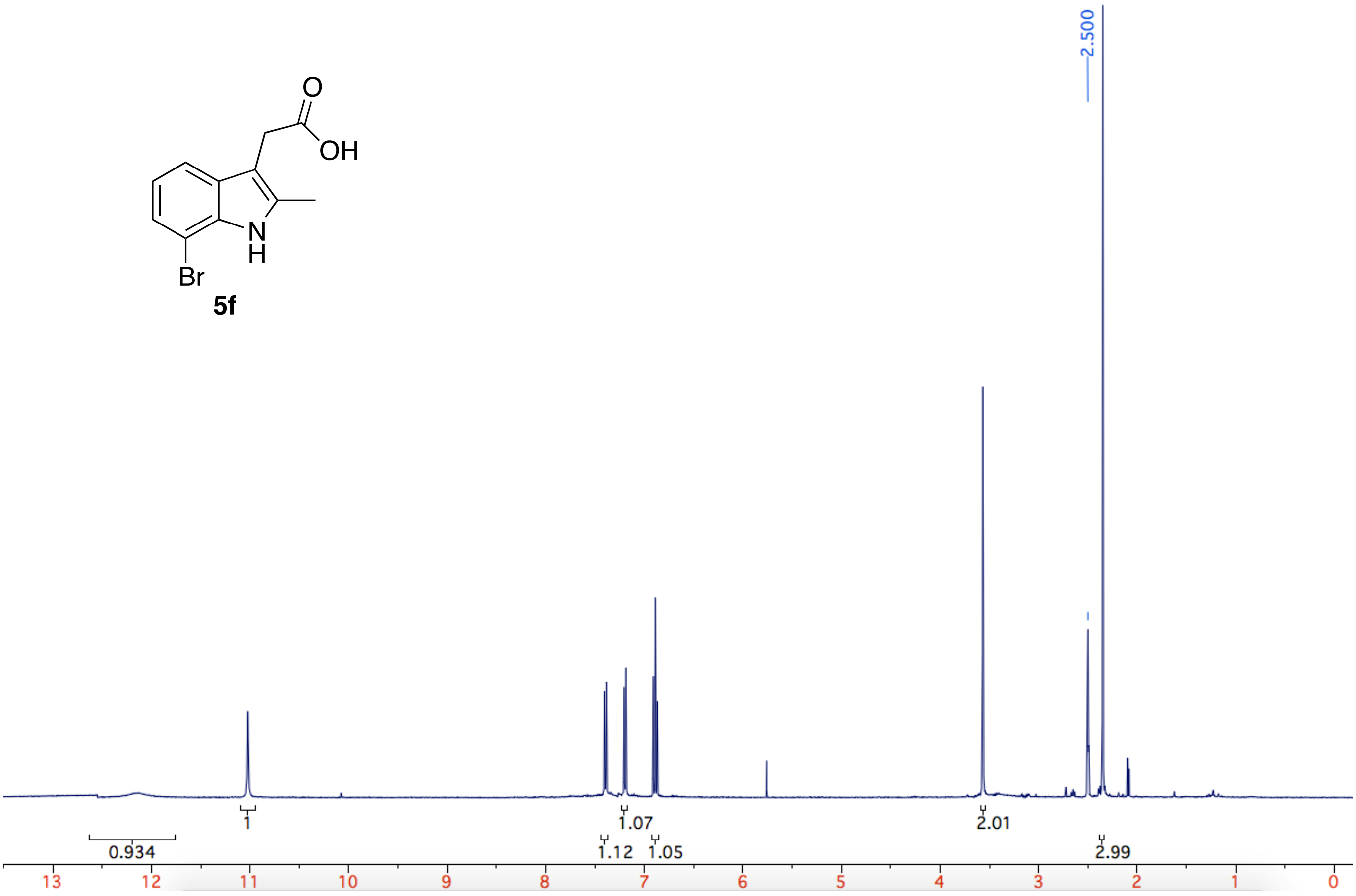
O
OH
N
H
Br
5f
2.500
1
1.07
2.01
0.934
1.12
1.05
2.99
13
12
11
10
9
8
7
6
5
4
3
2
1
0

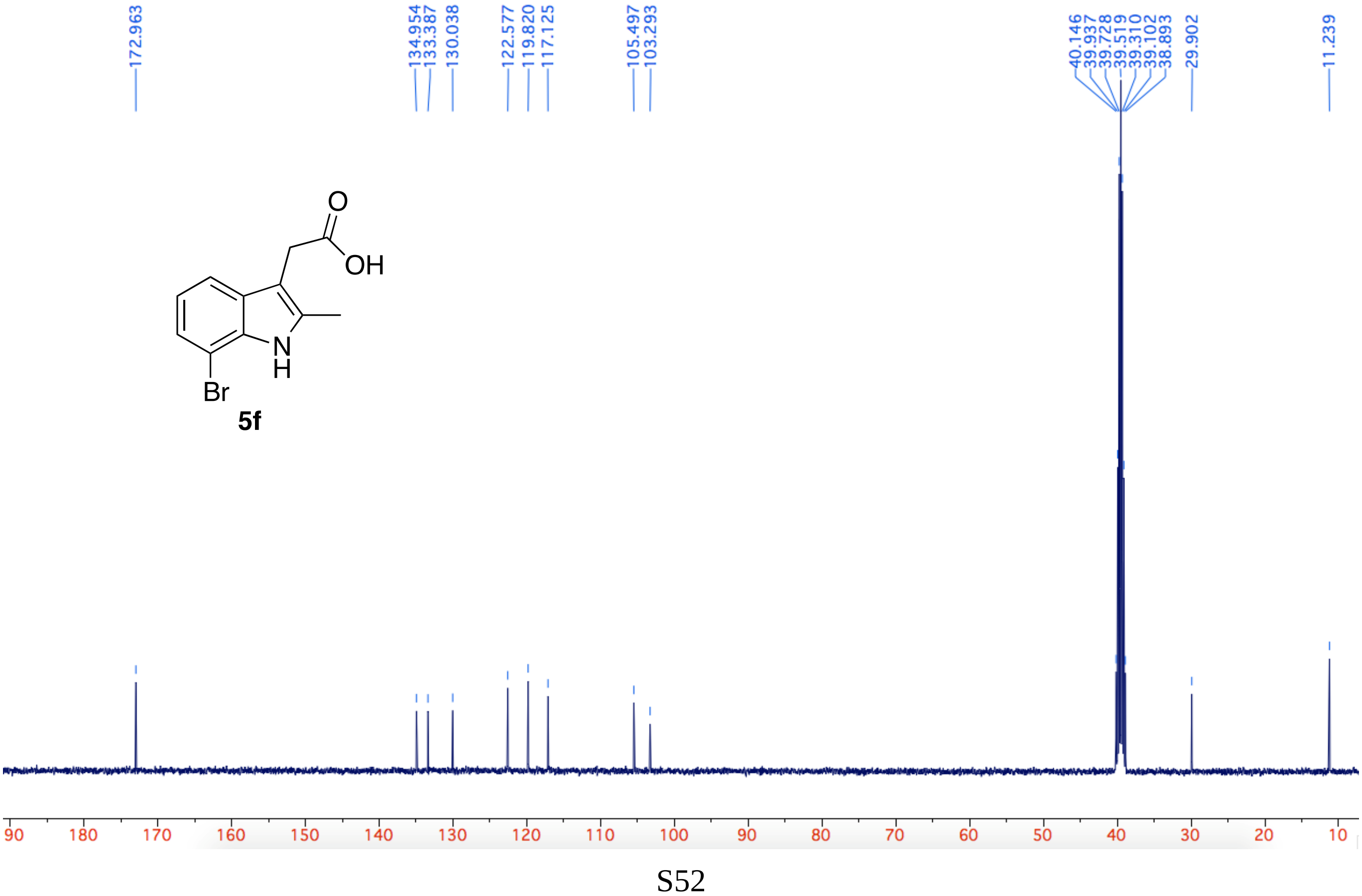
172.963
134.954
133.387
130.038
122.577
119.820
117.125
105.497
103.293
40.146
39.937
39.728
39.519
39.310
39.102
38.893
29.902
11.239
O
OH
N
H
Br
5f
90
180
170
160
150
140
130
120
110
100
90
80
70
60
50
40
30
20
10

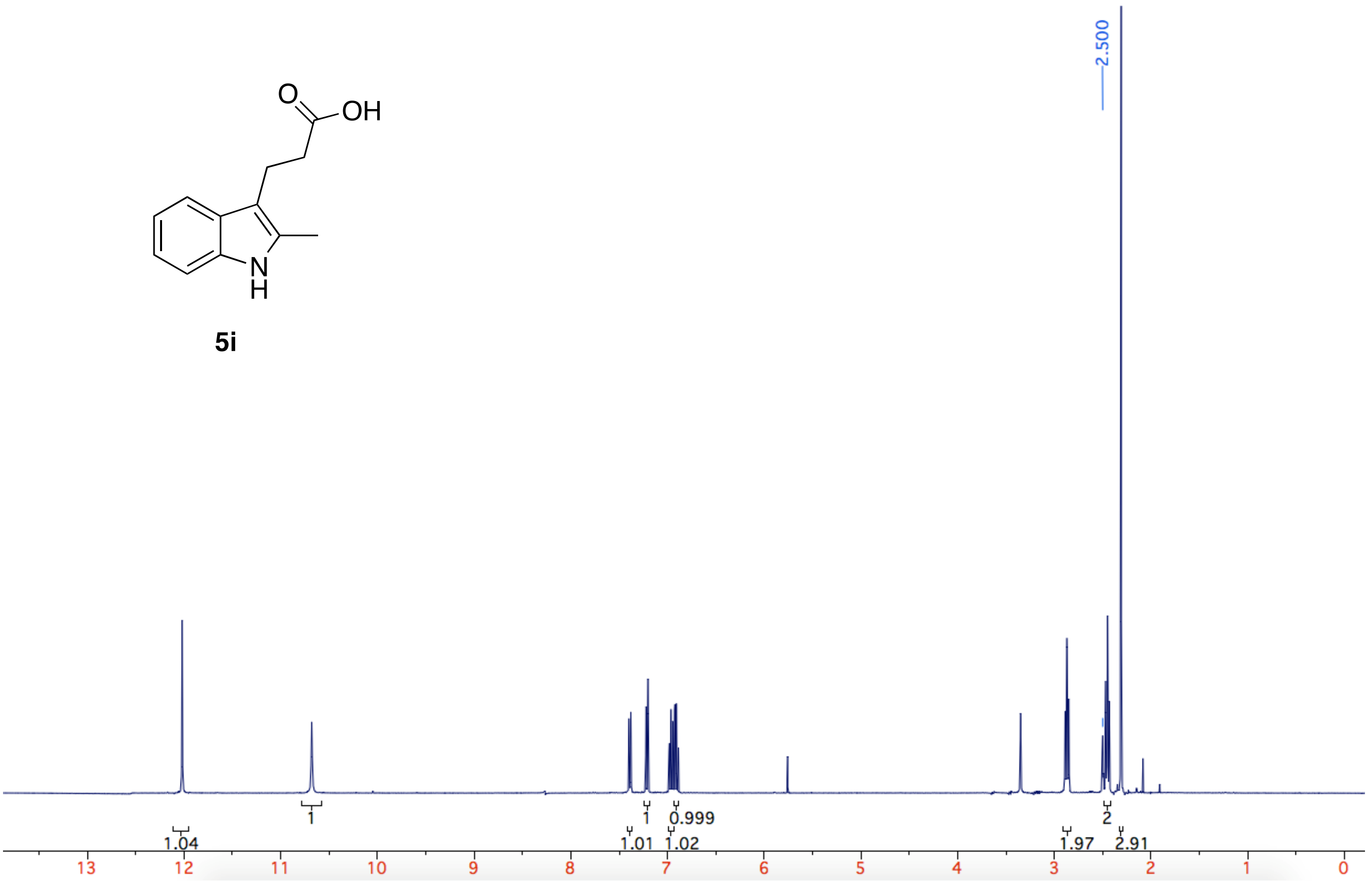

OH
O
N
H
5i
2.500
1.04
1
1
1.01
0.999
1.02
1.97
2
2.91
13 12 11 10 9 8 7 6 5 4 3 2 1 0

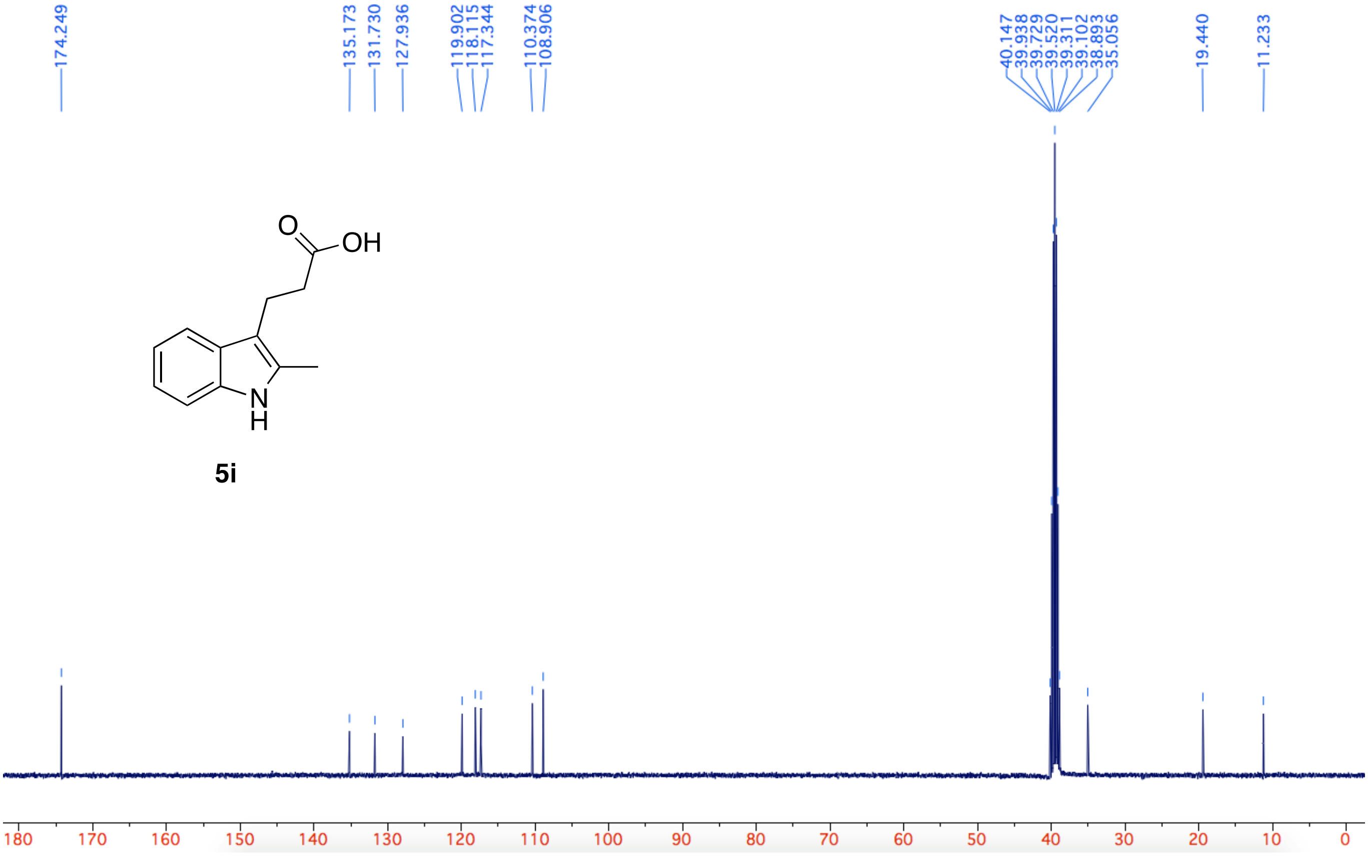

174.249
135.173
131.730
127.936
119.902
118.115
117.344
110.374
108.906
40.147
39.938
39.729
39.520
39.311
39.102
38.893
35.056
19.440
11.233
OH
O
N
H
5i
180 170 160 150 140 130 120 110 100 90 80 70 60 50 40 30 20 10 0

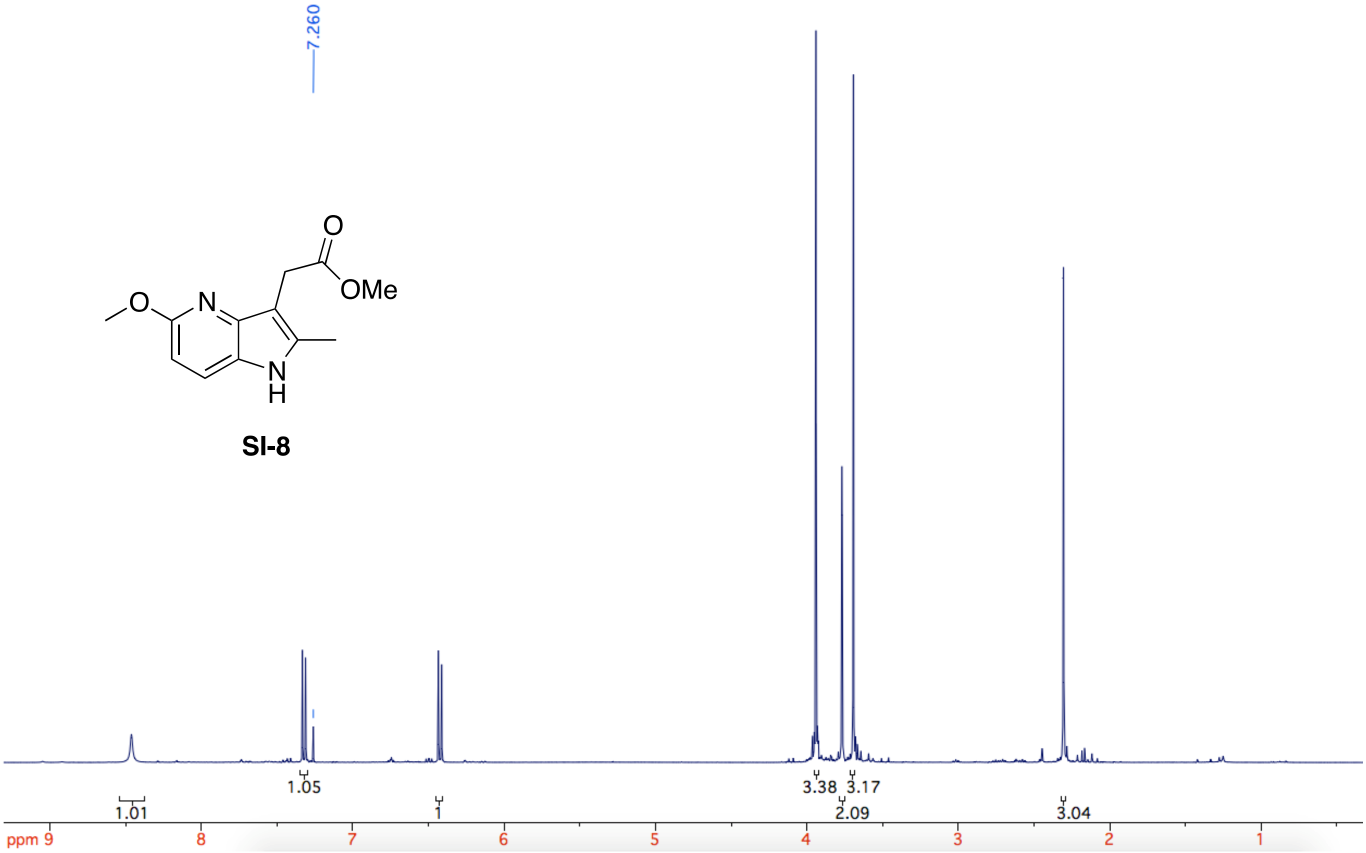

7.260
O
OMe
O
N
N
H
SI-8
1.01
1.05
1
3.38
3.17
2.09
3.04
ppm 9
8
7
6
5
4
3
2
1

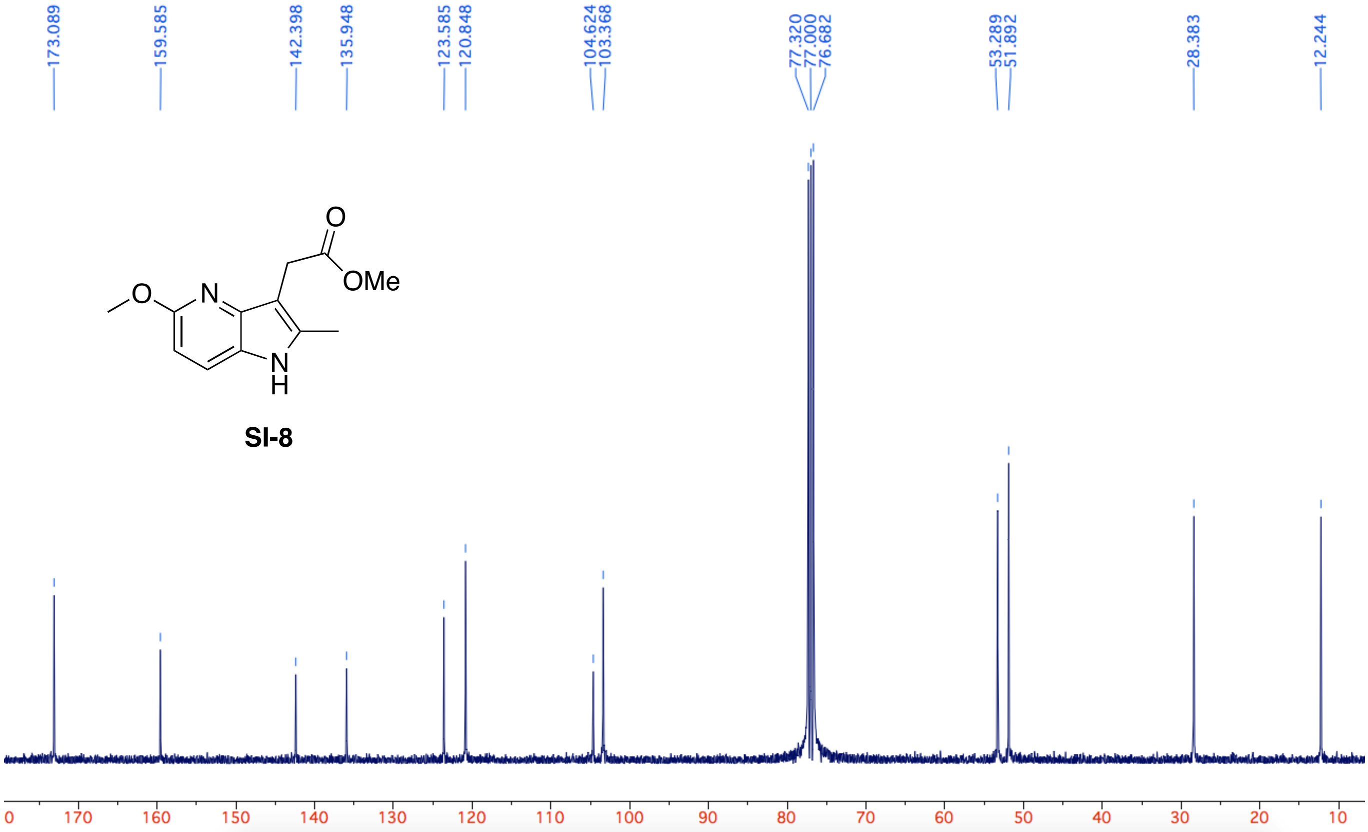

173.089
159.585
142.398
135.948
123.585
120.848
104.624
103.368
77.320
77.000
76.682
53.289
51.892
28.383
12.244
O
OMe
O
N
N
H
SI-8
0
170
160
150
140
130
120
110
100
90
80
70
60
50
40
30
20
10

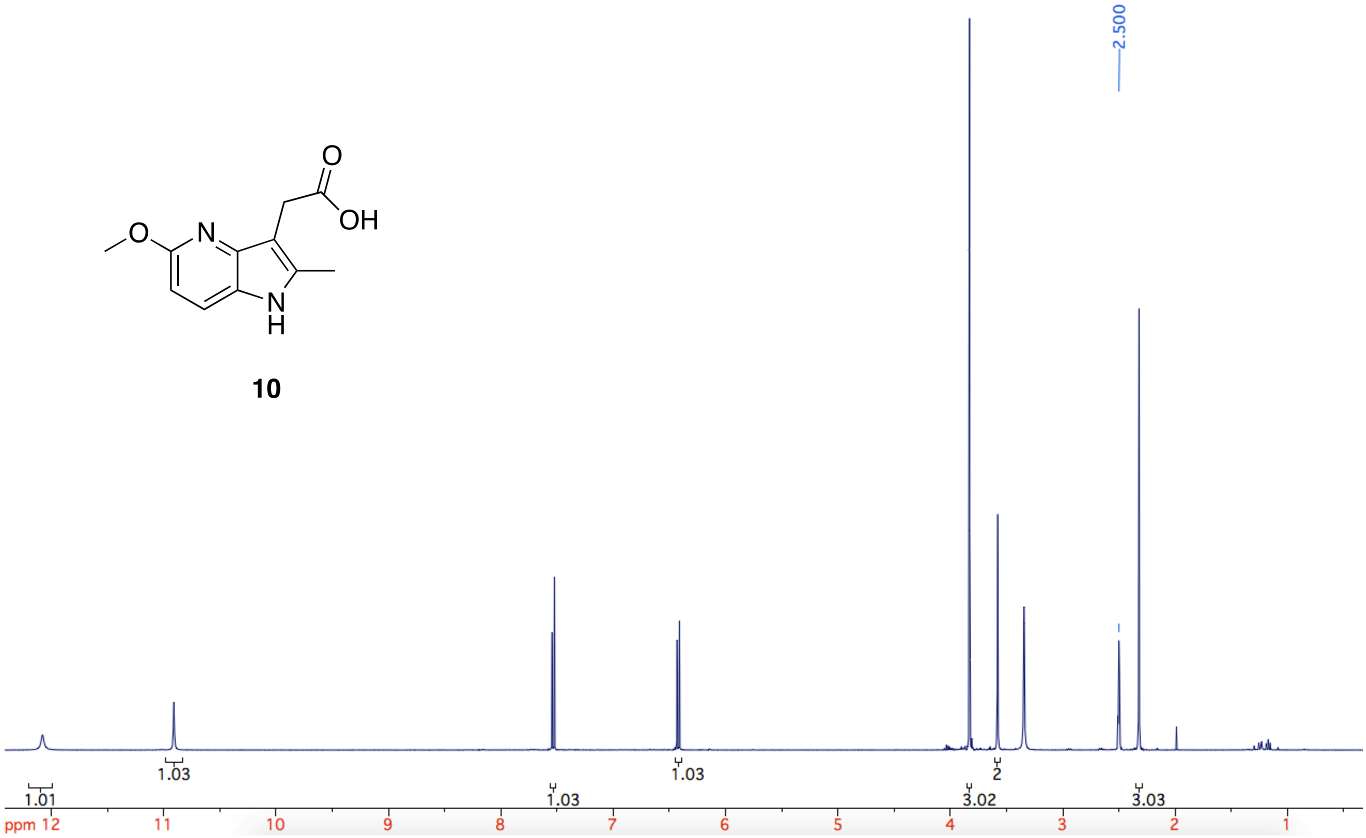
OH
O
N
N
H
10
2.500
1.01
1.03
1.03
1.03
3.02
2
3.03
ppm 12
11
10
9
8
7
6
5
4
3
2
1

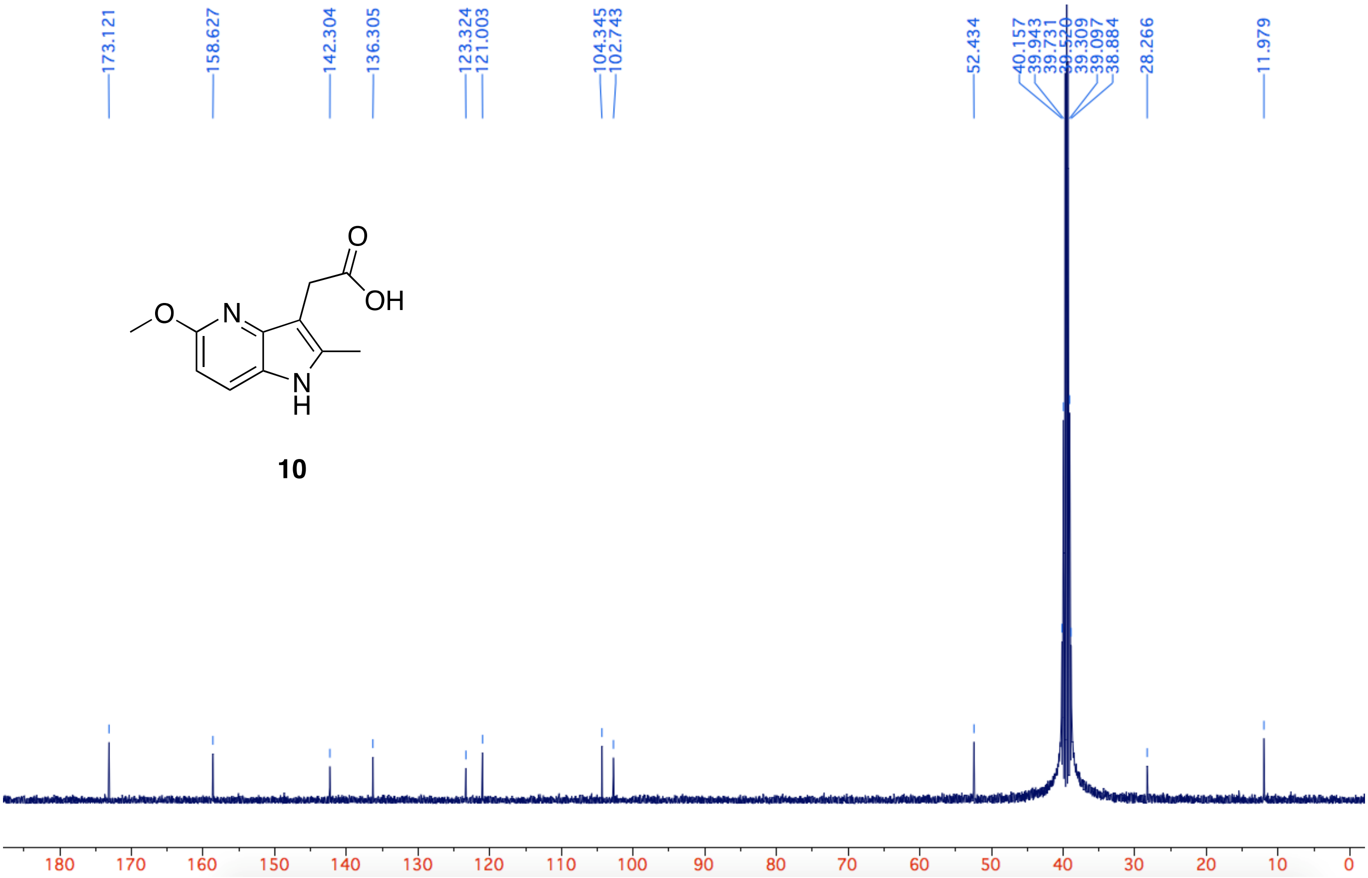
173.121
158.627
142.304
136.305
123.324
121.003
104.345
102.743
52.434
40.157
39.943
39.731
39.520
39.309
39.097
38.884
28.266
11.979
OH
O
N
N
H
10
180
170
160
150
140
130
120
110
100
90
80
70
60
50
40
30
20
10
0

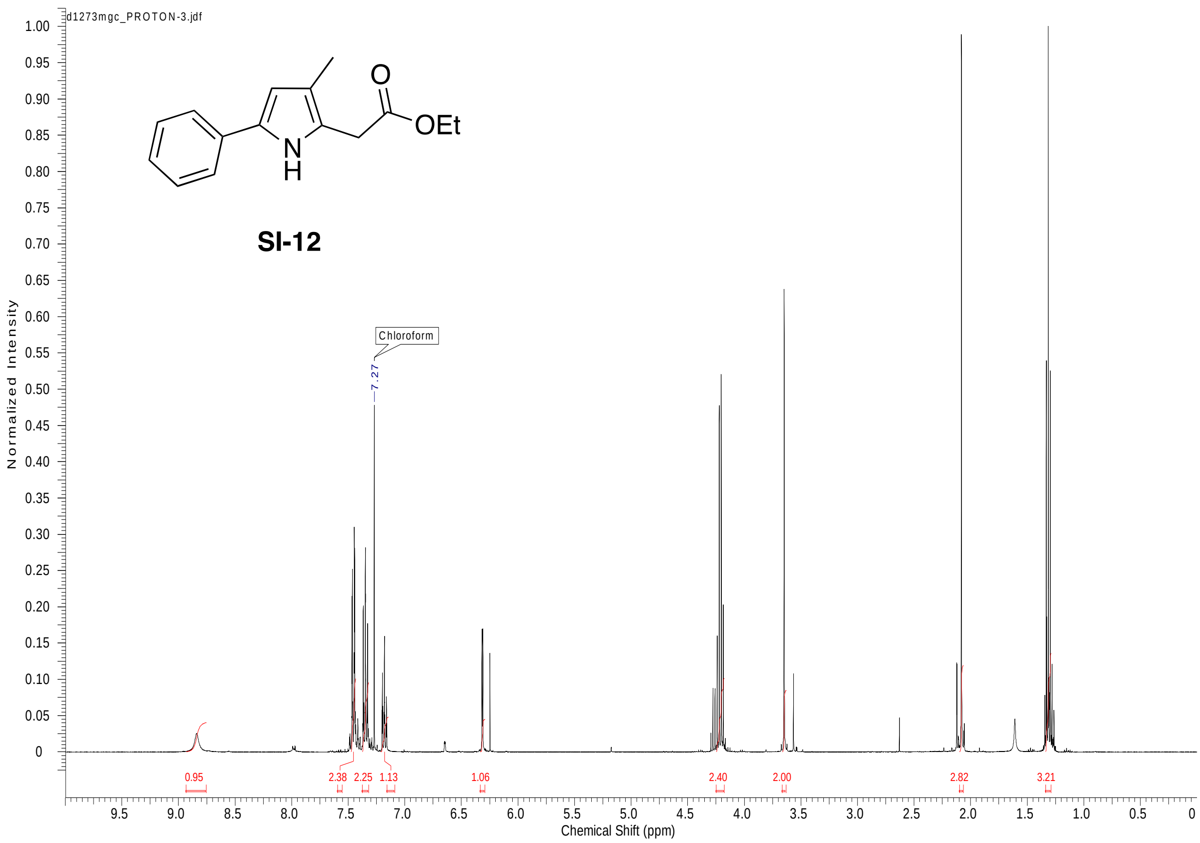
d1273mgc_PROTON-3.jdf
SI-12
OEt
O
N
H
Chloroform
7.27
0.95
2.38
2.25
1.13
1.06
2.40
2.00
2.82
3.21
Normalized Intensity
Chemical Shift (ppm)

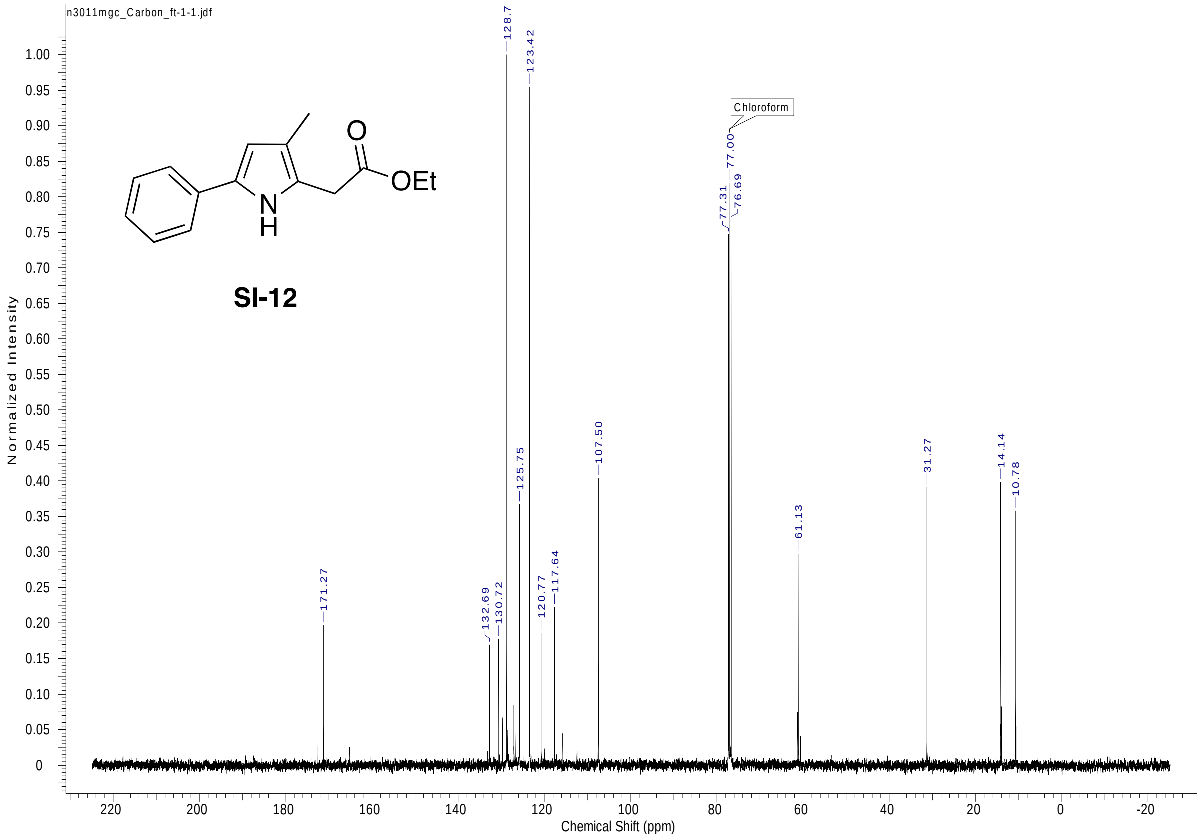
n3011mgc_Carbon_ft-1-1.jdf
SI-12
OEt
O
N
H
Chloroform
171.27
132.69
130.72
128.7
125.75
123.42
120.77
117.64
107.50
77.31
77.00
76.69
61.13
31.27
14.14
10.78
Normalized Intensity
Chemical Shift (ppm)

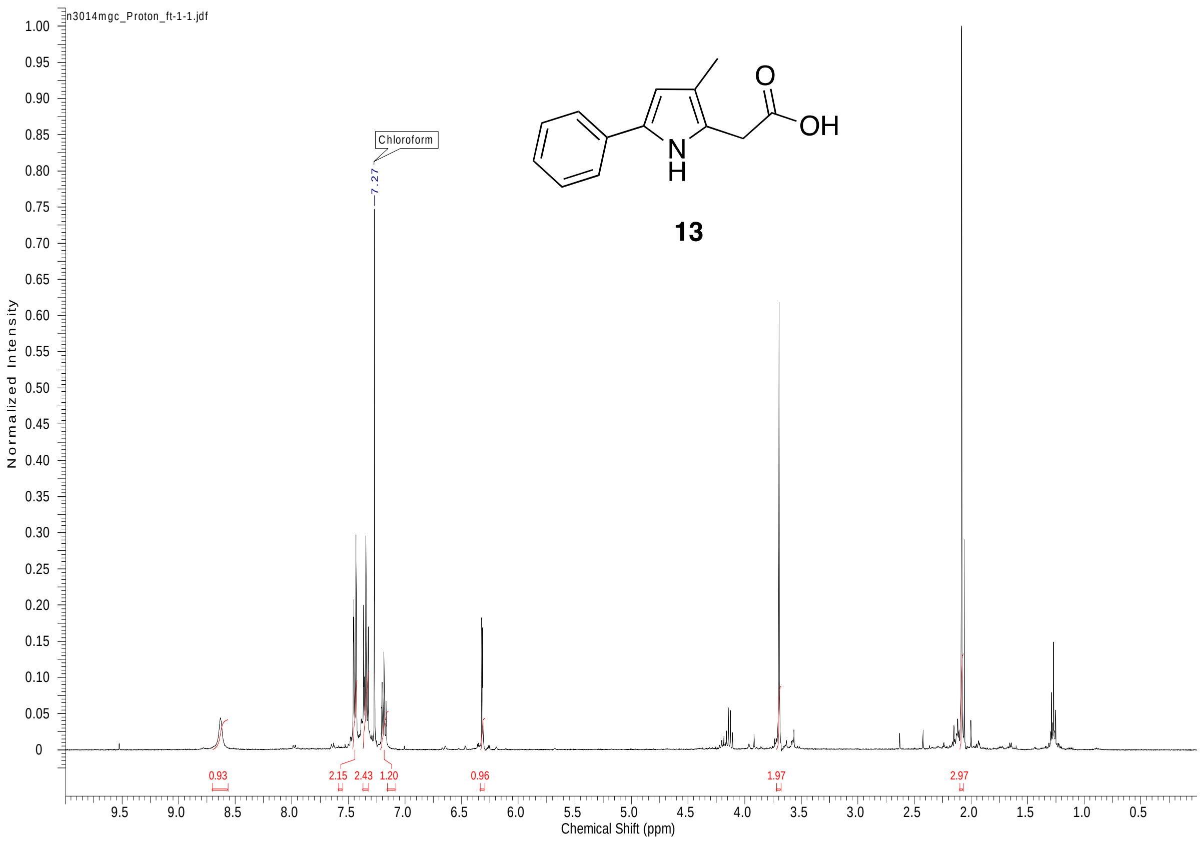
n3014mgc_Proton_ft-1-1.jdf
Normalized Intensity
Chemical Shift (ppm)
Chloroform
7.27
O
OH
N
H
13
0.93
2.15
2.43
1.20
0.96
1.97
2.97

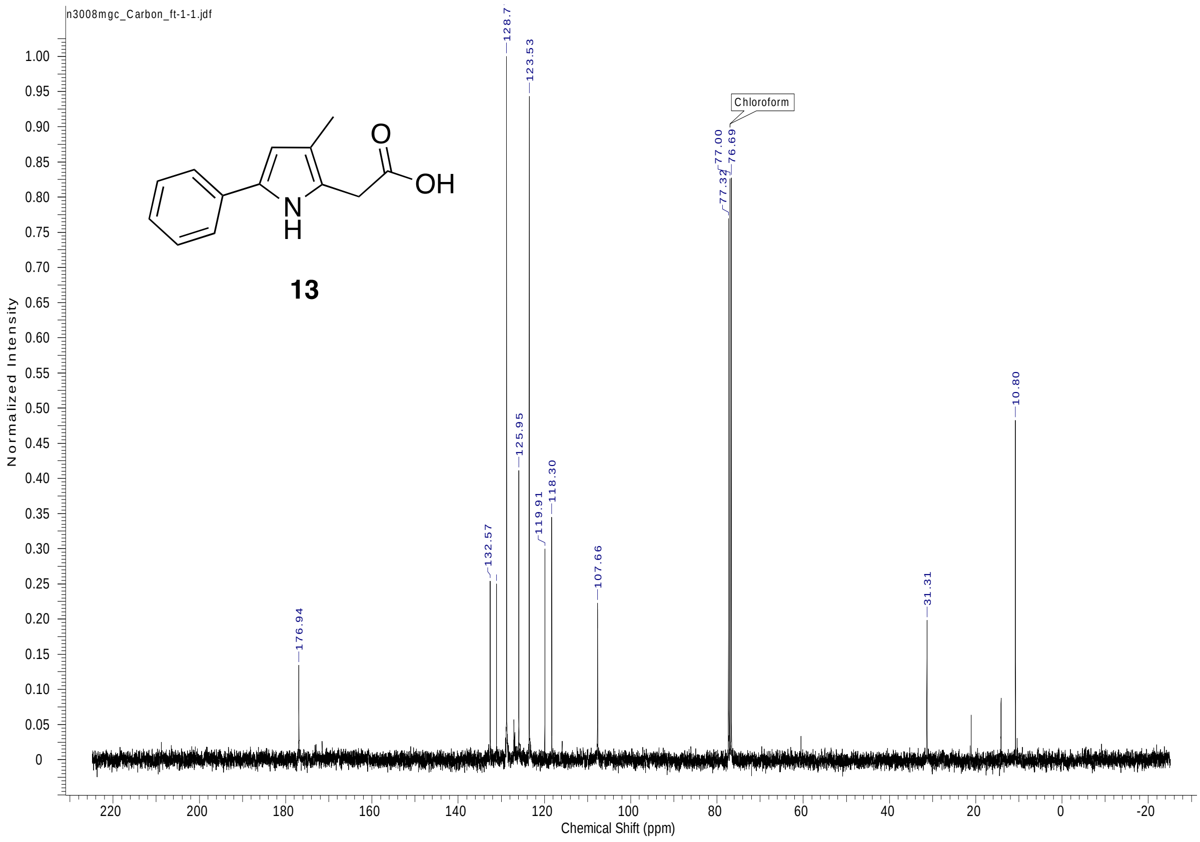
n3008mgc_Carbon_ft-1-1.jdf
Normalized Intensity
Chemical Shift (ppm)
Chloroform
O
OH
N
H
13
176.94
132.57
128.7
125.95
123.53
119.91
118.30
107.66
77.32
77.00
76.69
31.31
10.80

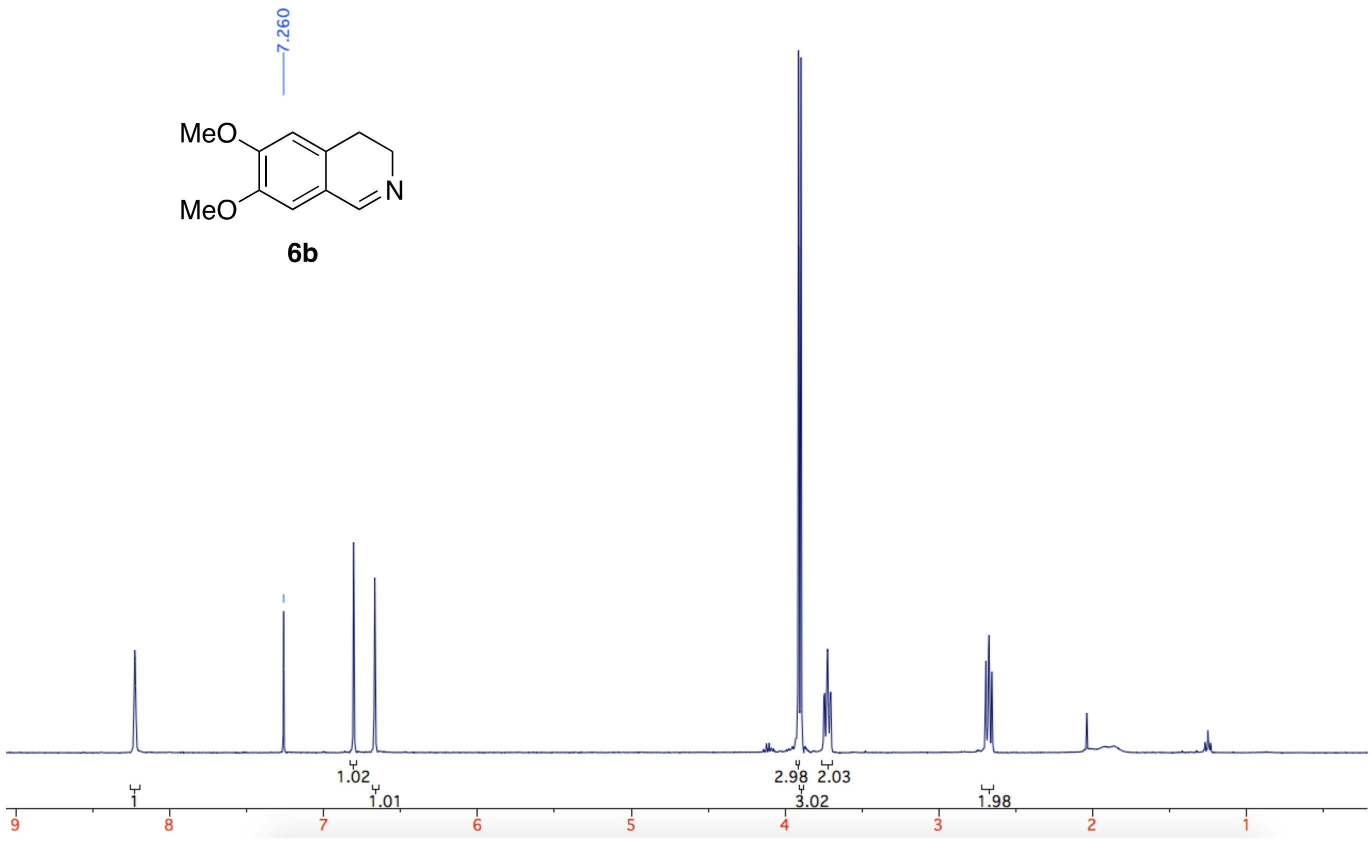
7.260
MeO
MeO
N
6b
1.02
1.01
1
2.98
3.02
2.03
1.98
9
8
7
6
5
4
3
2
1

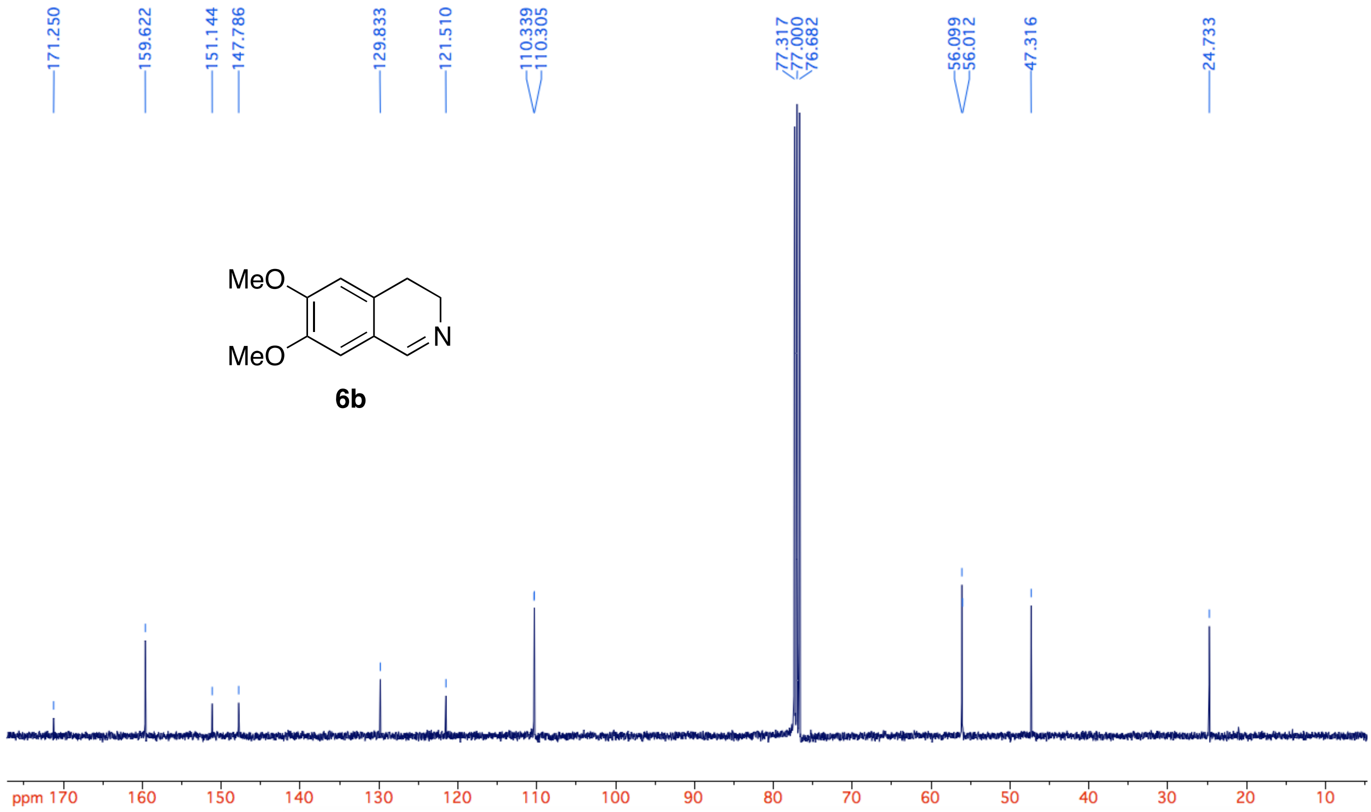
171.250
159.622
151.144
147.786
129.833
121.510
110.339
110.305
77.317
77.000
76.682
56.099
56.012
47.316
24.733
MeO
MeO
N
6b
ppm 170
160
150
140
130
120
110
100
90
80
70
60
50
40
30
20
10

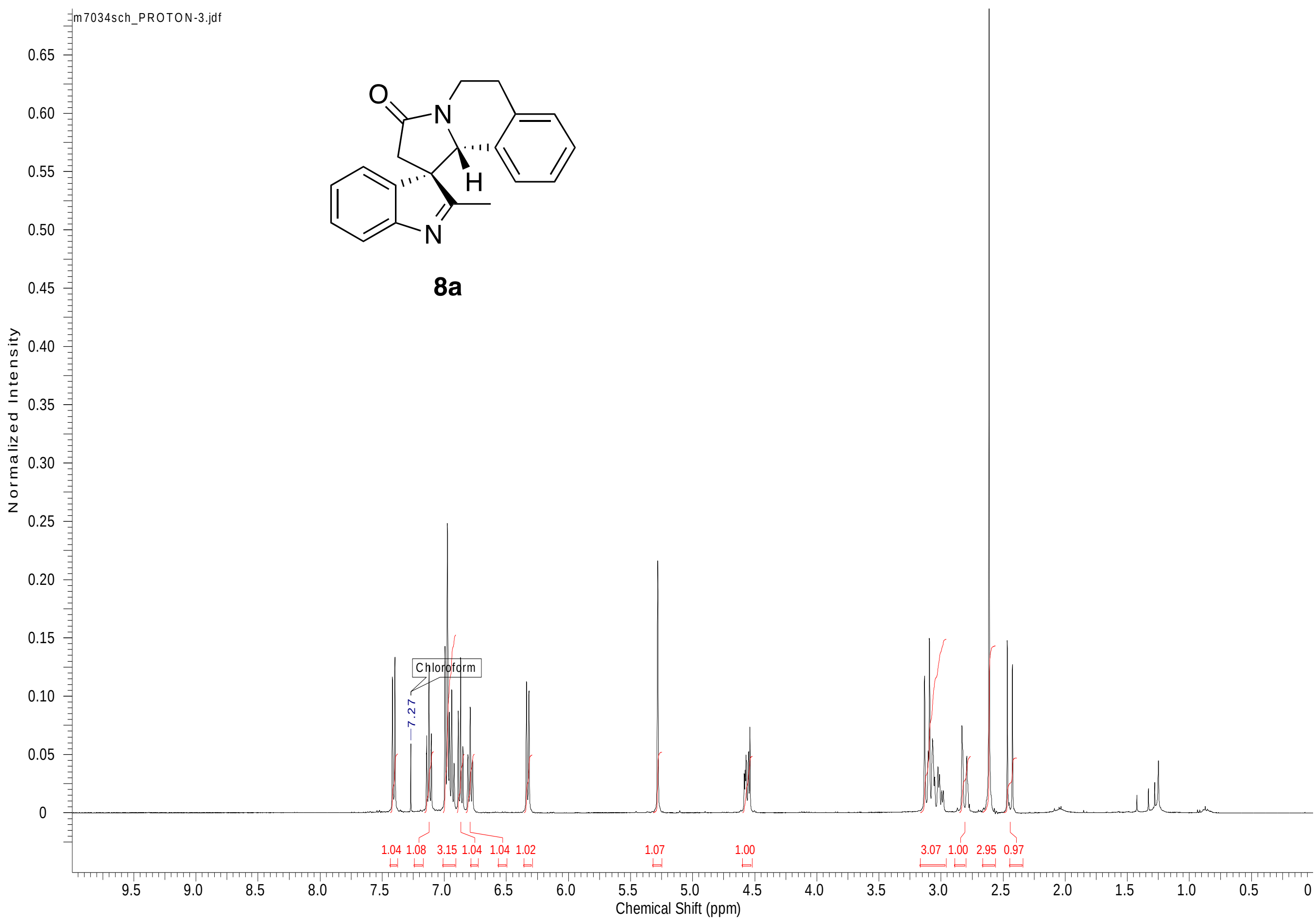

m7034sch_PROTON-3.jdf
Normalized Intensity
Chemical Shift (ppm)
Chloroform
7.27
8a
1.04 1.08 3.15 1.04 1.04 1.02 1.07 1.00 3.07 1.00 2.95 0.97

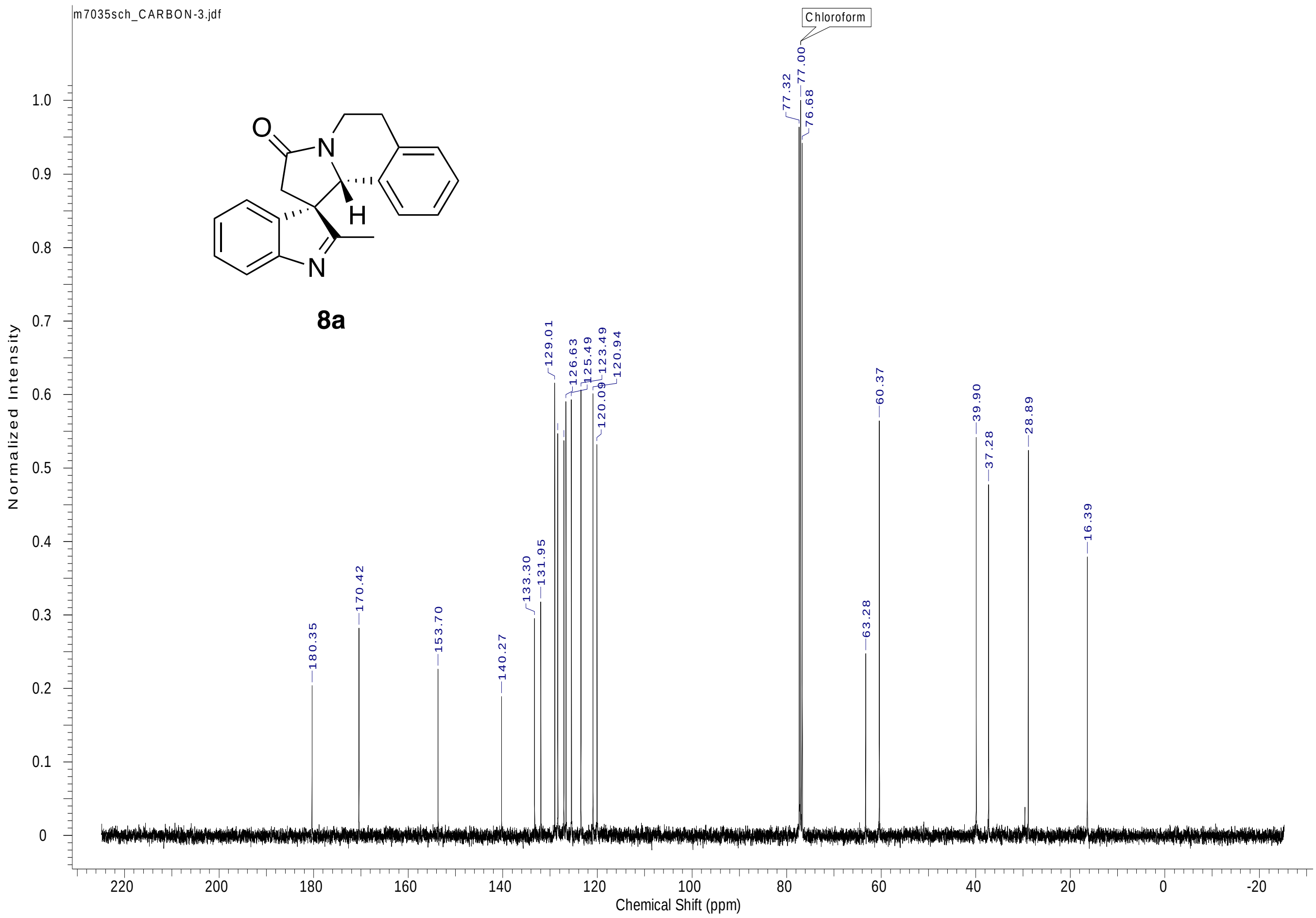

m7035sch_CARBON-3.jdf
Normalized Intensity
Chemical Shift (ppm)
Chloroform
77.32
77.00
76.68
8a
180.35
170.42
153.70
140.27
133.30
131.95
129.01
126.63
125.49
123.49
120.94
120.09
63.28
60.37
39.90
37.28
28.89
16.39

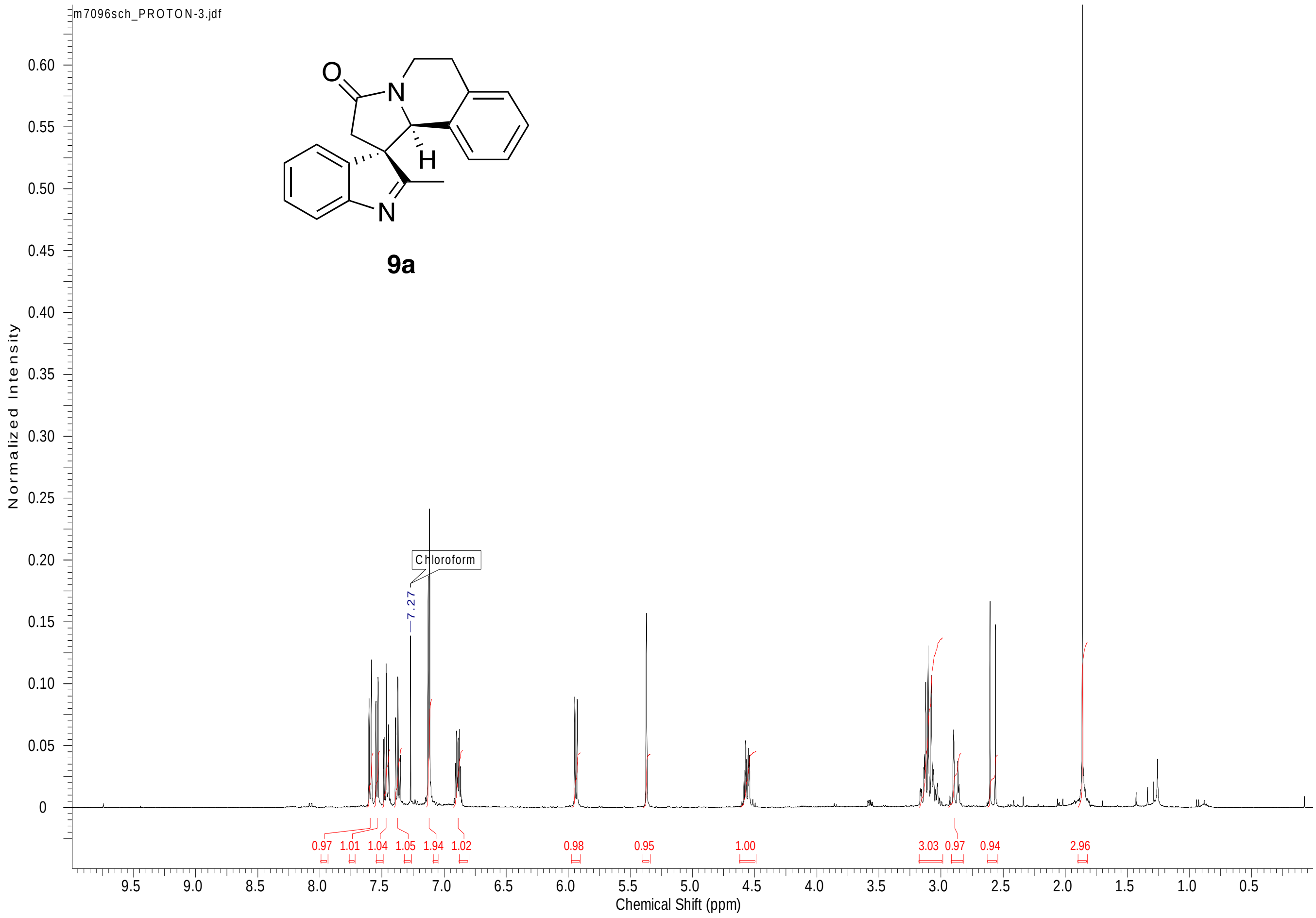
m7096sch_PROTON-3.jdf
O
N
H
N
9a
Chloroform
7.27
0.97
1.01
1.04
1.05
1.94
1.02
0.98
0.95
1.00
3.03
0.97
0.94
2.96
Normalized Intensity
Chemical Shift (ppm)

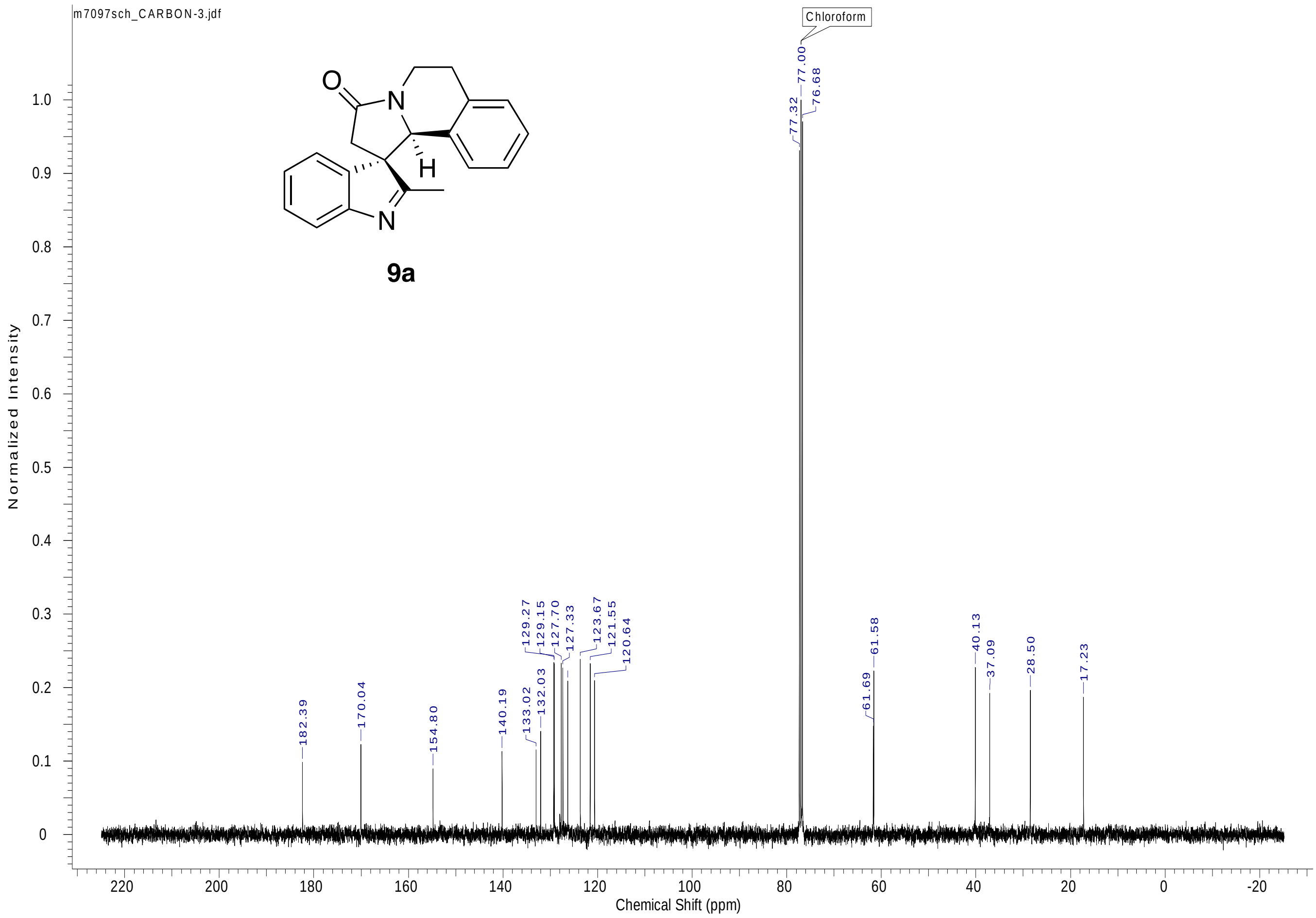
m7097sch_CARBON-3.jdf
O
N
H
N
9a
Chloroform
77.32
77.00
76.68
182.39
170.04
154.80
140.19
133.02
132.03
129.27
129.15
127.70
127.33
123.67
121.55
120.64
61.69
61.58
40.13
37.09
28.50
17.23
Normalized Intensity
Chemical Shift (ppm)

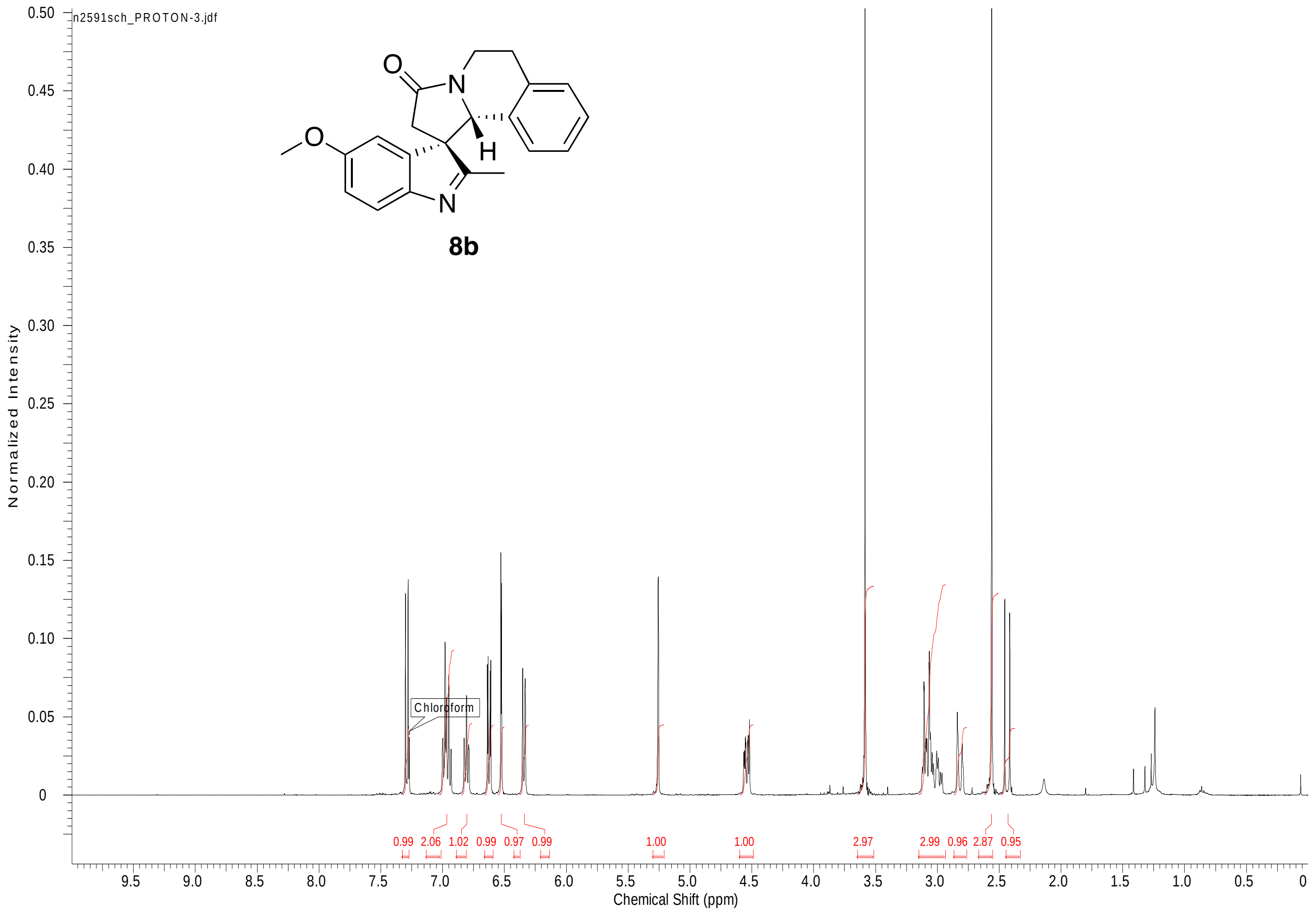
n2591sch_PROTON-3.jdf
8b
Chloroform
0.99 2.06 1.02 0.99 0.97 0.99 1.00 1.00 2.97 2.99 0.96 2.87 0.95
Normalized Intensity
Chemical Shift (ppm)

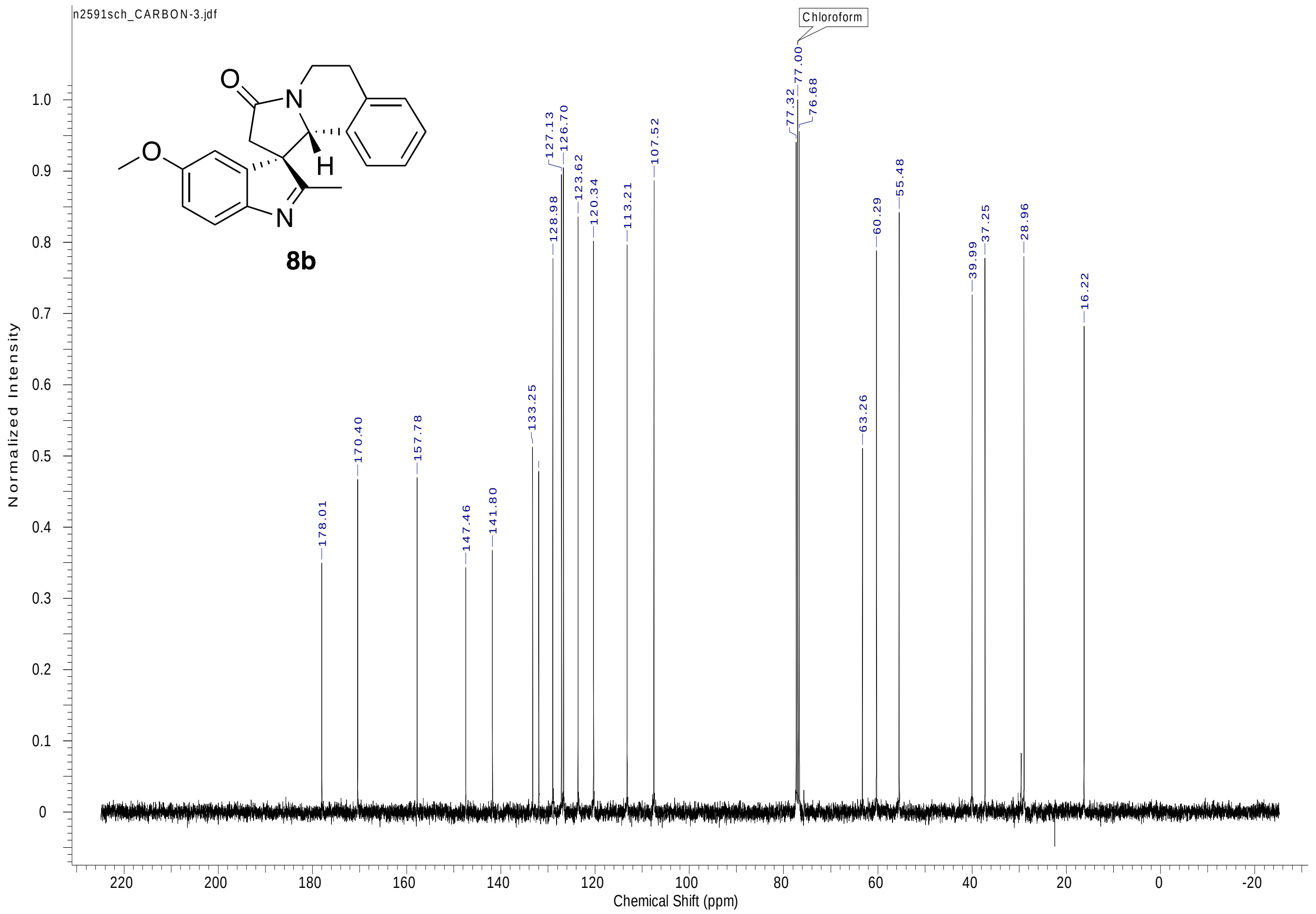
n2591sch_CARBON-3.jdf
8b
Chloroform
178.01
170.40
157.78
147.46
141.80
133.25
128.98
127.13
126.70
123.62
120.34
113.21
107.52
77.32
77.00
76.68
63.26
60.29
55.48
39.99
37.25
28.96
16.22
Normalized Intensity
Chemical Shift (ppm)

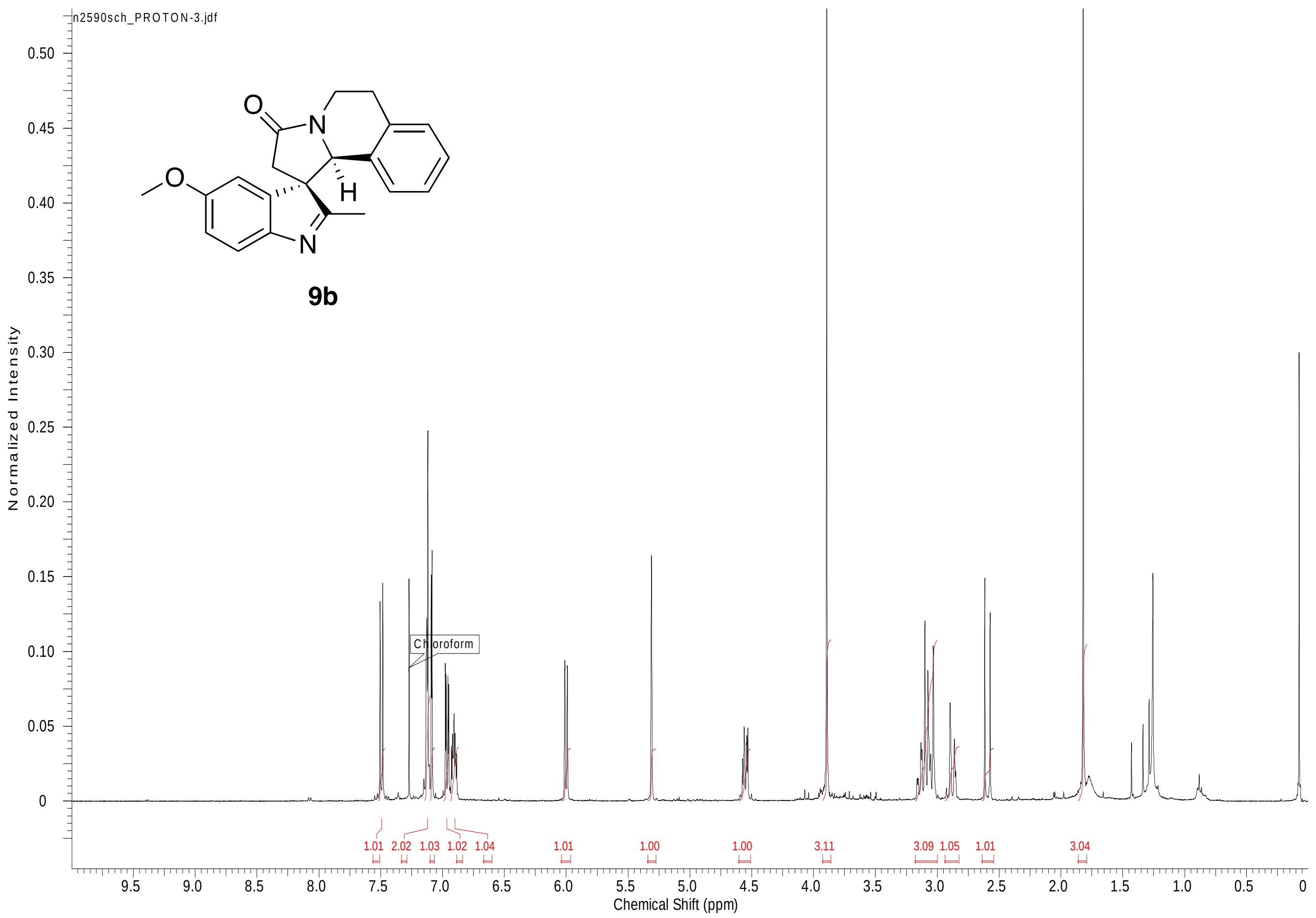
n2590sch_PROTON-3.jdf
9b
Chloroform
1.01
2.02
1.03
1.02
1.04
1.01
1.00
1.00
3.11
3.09
1.05
1.01
3.04
Normalized Intensity
Chemical Shift (ppm)

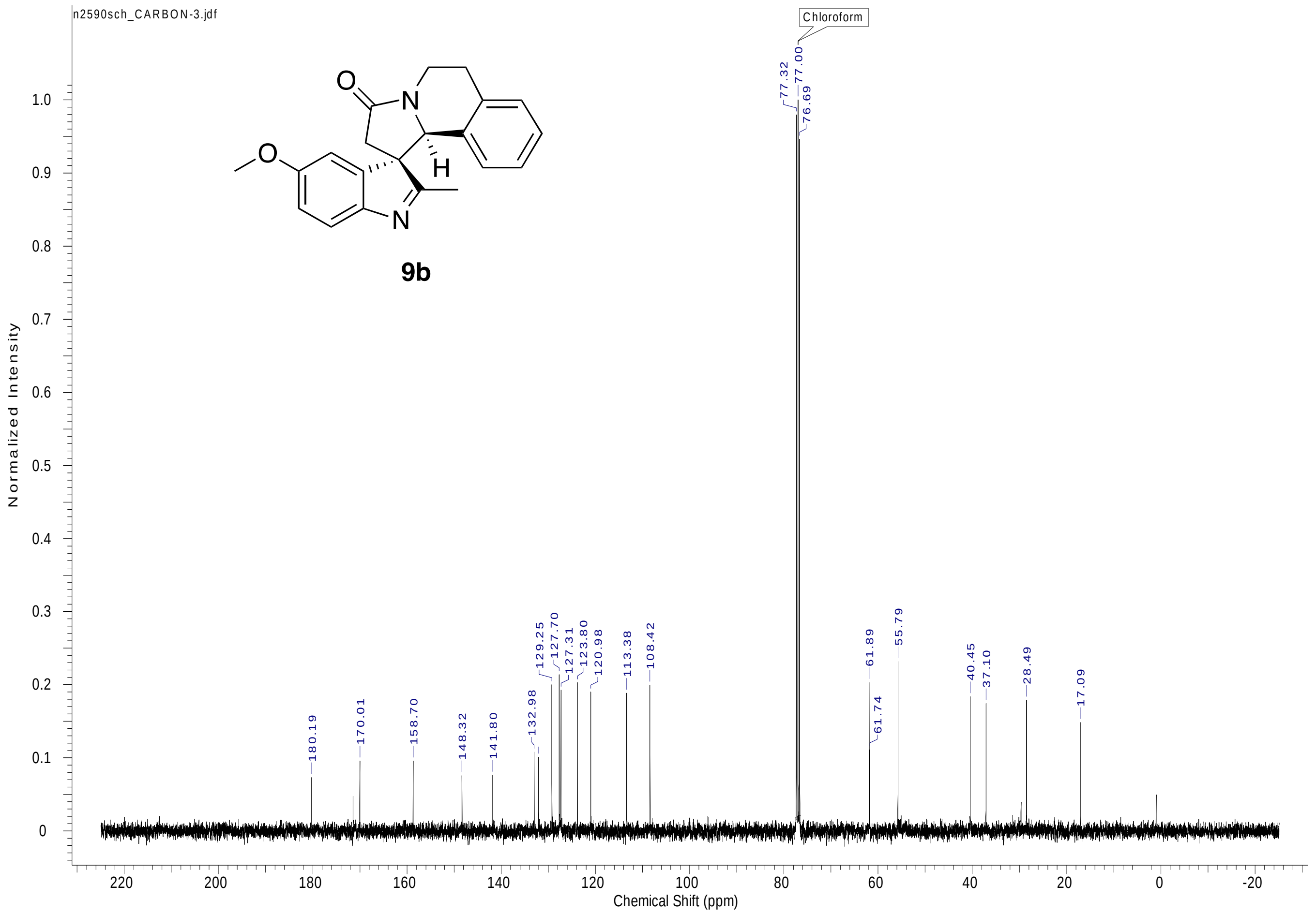
n2590sch_CARBON-3.jdf
9b
Chloroform
77.32
77.00
76.69
180.19
170.01
158.70
148.32
141.80
132.98
129.25
127.70
127.31
123.80
120.98
113.38
108.42
61.89
61.74
55.79
40.45
37.10
28.49
17.09
Normalized Intensity
Chemical Shift (ppm)

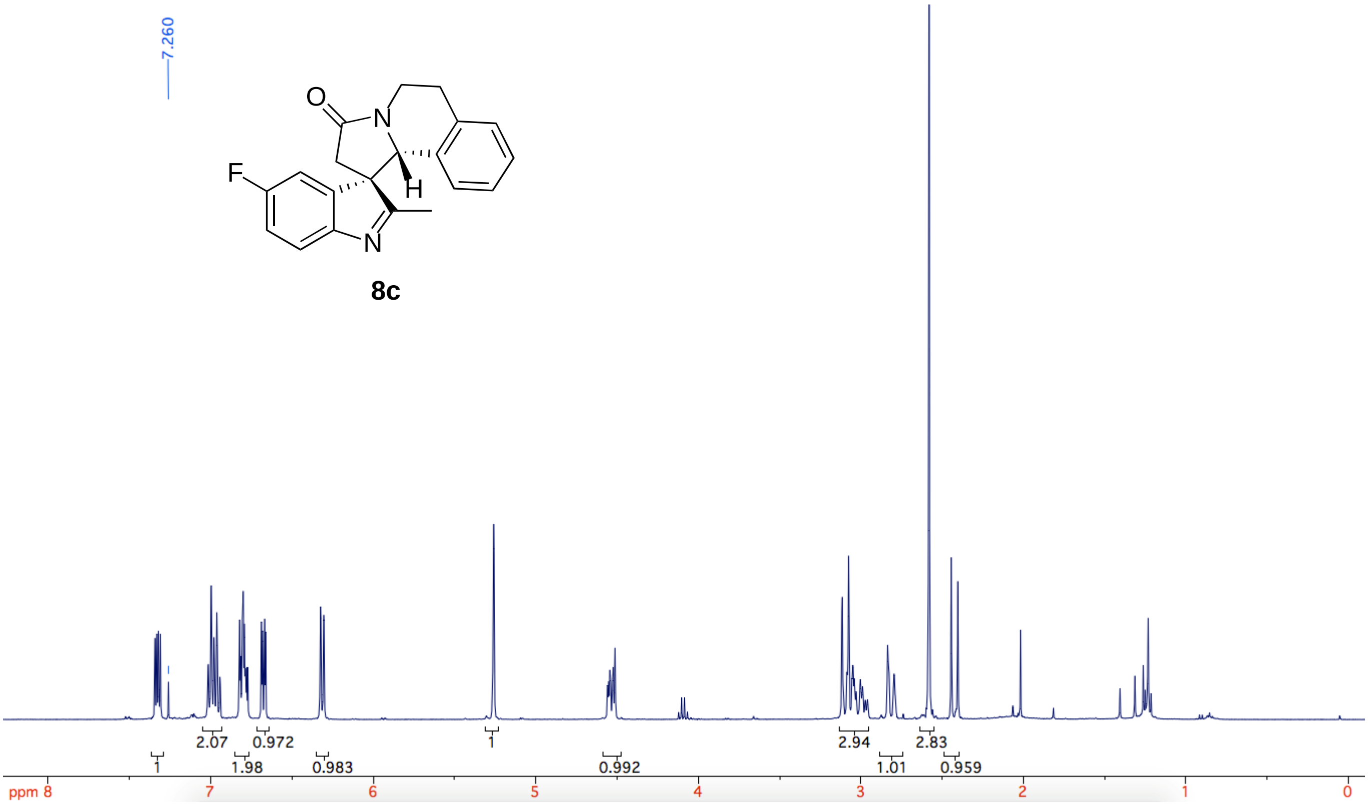
7.260
8c
1
2.07
1.98
0.972
0.983
1
0.992
2.94
1.01
2.83
0.959
ppm 8
7
6
5
4
3
2
1
0

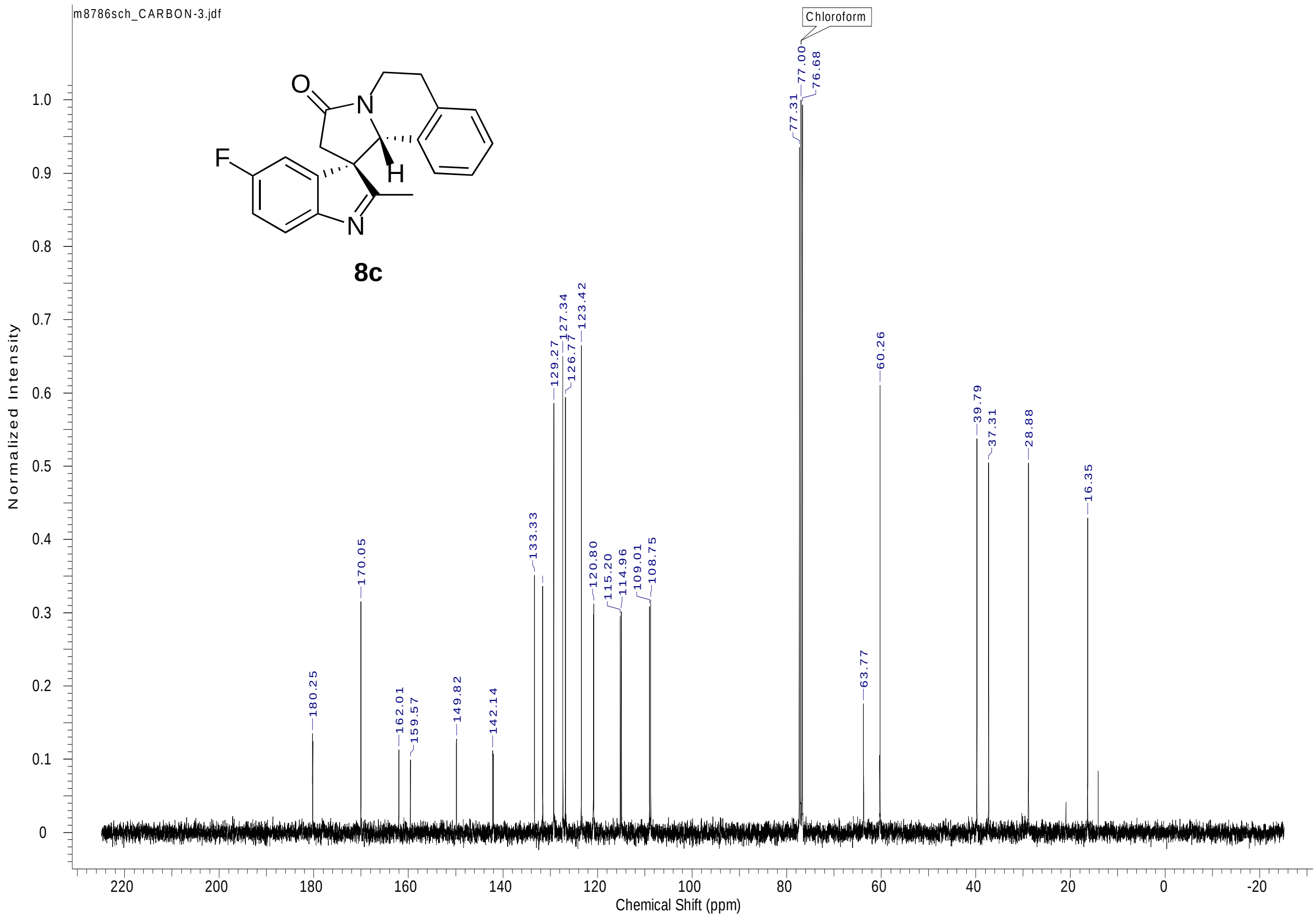
m8786sch_CARBON-3.jdf
8c
Chloroform
77.31
77.00
76.68
180.25
170.05
162.01
159.57
149.82
142.14
133.33
129.27
127.34
126.77
123.42
120.80
115.20
114.96
109.01
108.75
63.77
60.26
39.79
37.31
28.88
16.35
Normalized Intensity
Chemical Shift (ppm)

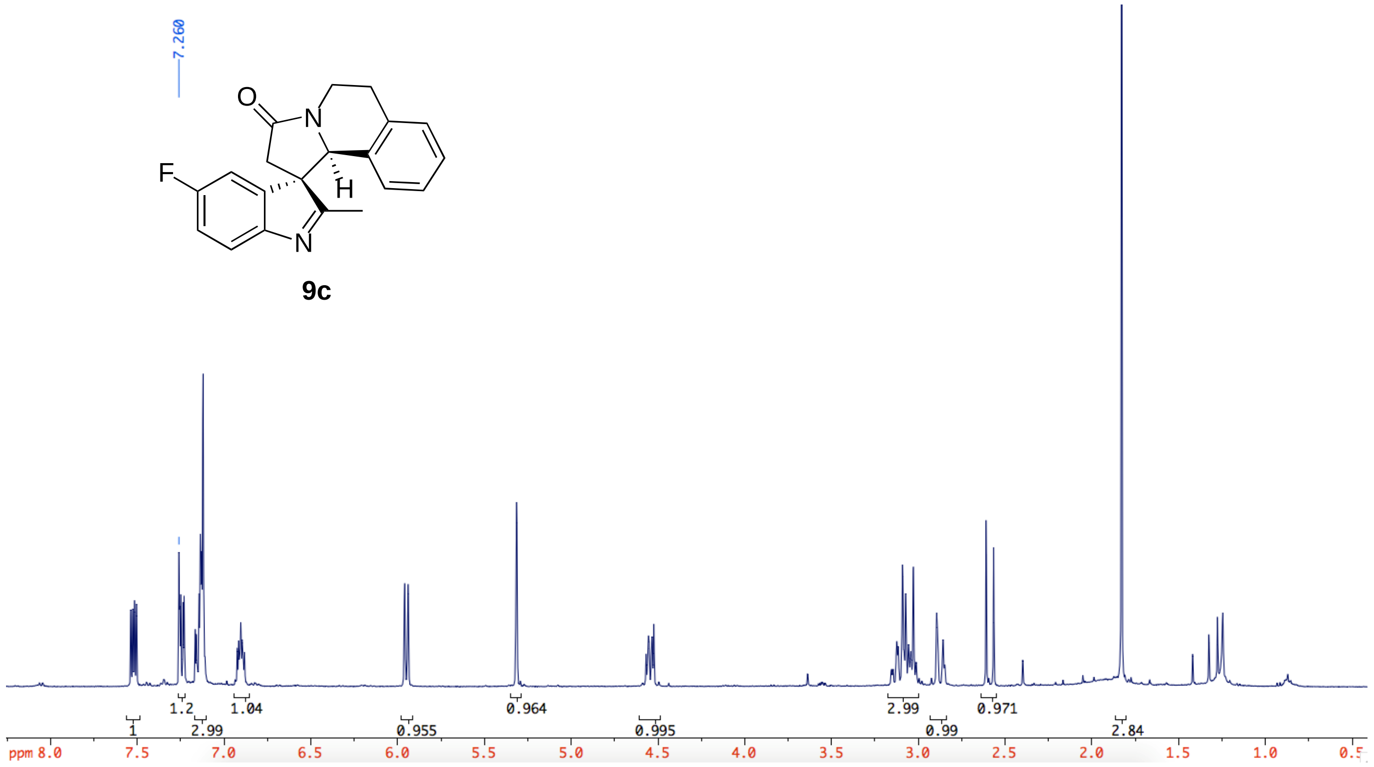
7.260
O
N
F
H
N
9c
1
1.2
2.99
1.04
0.955
0.964
0.995
2.99
0.99
0.971
2.84
ppm 8.0
7.5
7.0
6.5
6.0
5.5
5.0
4.5
4.0
3.5
3.0
2.5
2.0
1.5
1.0

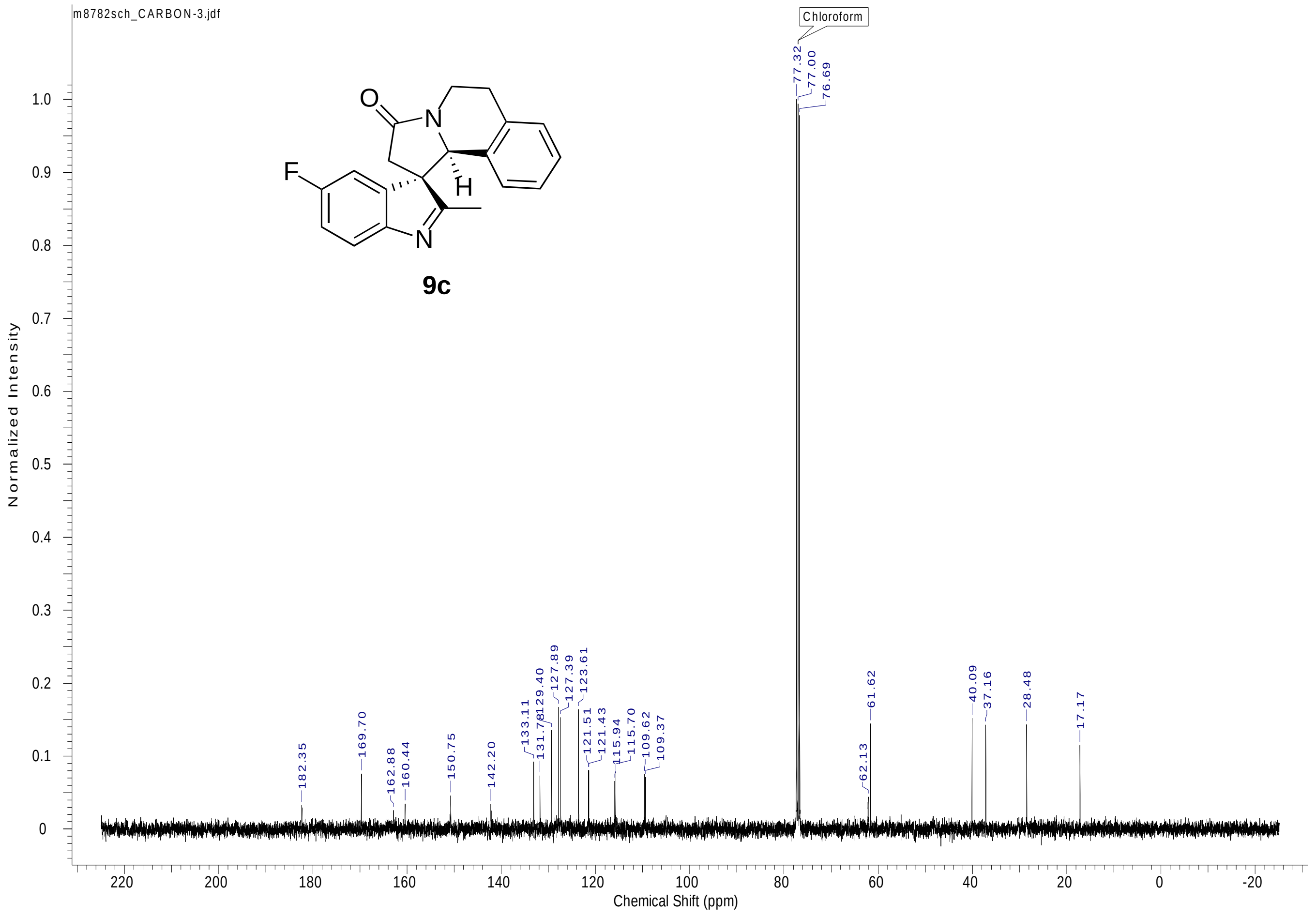
m8782sch_CARBON-3.jdf
Chloroform
77.32
77.00
76.69
O
N
F
H
N
9c
182.35
169.70
162.88
160.44
150.75
142.20
133.11
131.78
129.40
127.89
127.39
123.61
121.51
121.43
115.94
115.70
109.62
109.37
62.13
61.62
40.09
37.16
28.48
17.17
Normalized Intensity
Chemical Shift (ppm)
220
200
180
160
140
120
100
80
60
40
20
0
-20

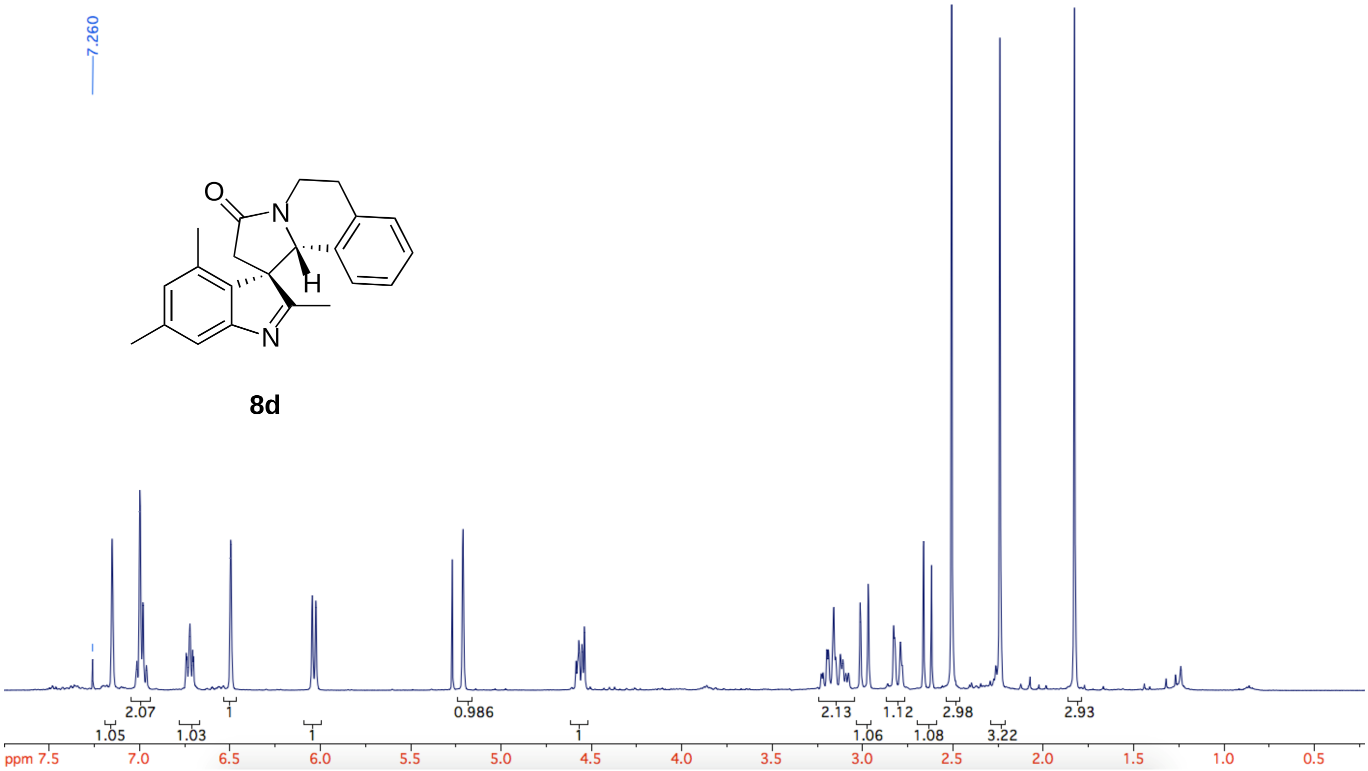
7.260
8d
2.07
1.05
1.03
1
1
0.986
1
2.13
1.06
1.12
1.08
2.98
3.22
2.93
ppm 7.5 7.0 6.5 6.0 5.5 5.0 4.5 4.0 3.5 3.0 2.5 2.0 1.5 1.0 0.5

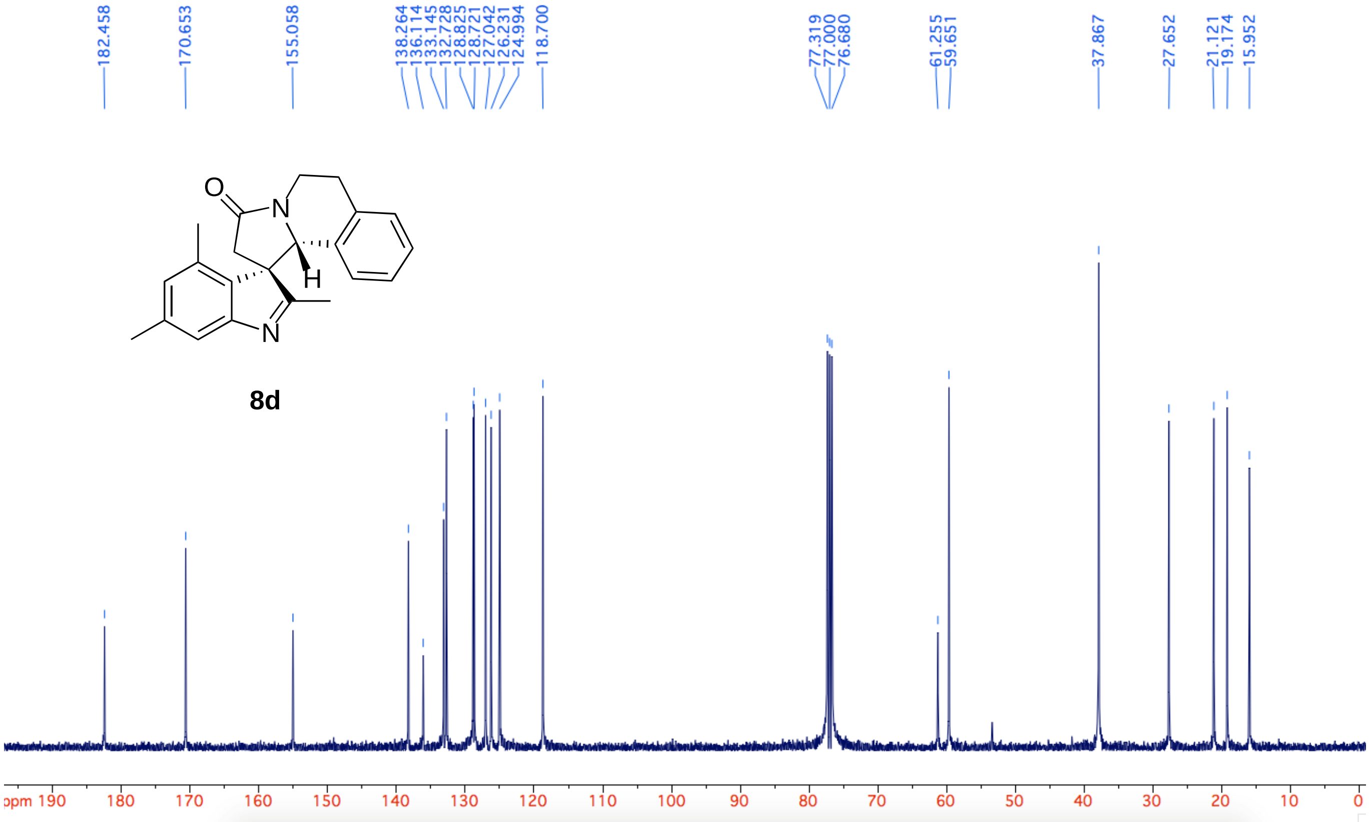
182.458
170.653
155.058
138.264
136.114
133.145
132.728
128.825
128.721
127.042
126.231
124.994
118.700
77.319
77.000
76.680
61.255
59.651
37.867
27.652
21.121
19.174
15.952
8d
ppm 190 180 170 160 150 140 130 120 110 100 90 80 70 60 50 40 30 20 10 0

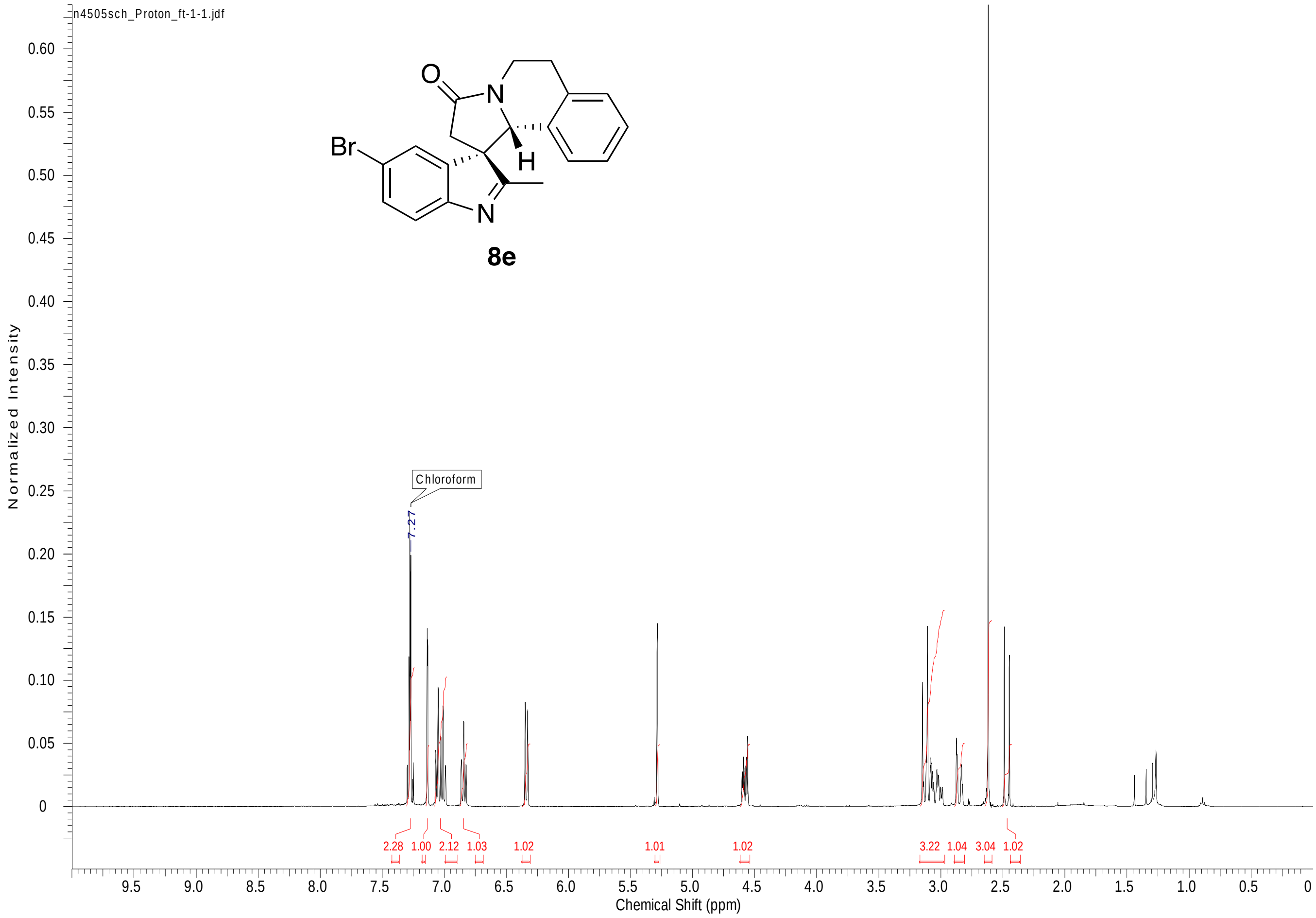
n4505sch_Proton_ft-1-1.jdf
O
N
Br
H
N
8e
Chloroform
7.27
2.28 1.00 2.12 1.03 1.02 1.01 1.02 3.22 1.04 3.04 1.02
Normalized Intensity
Chemical Shift (ppm)

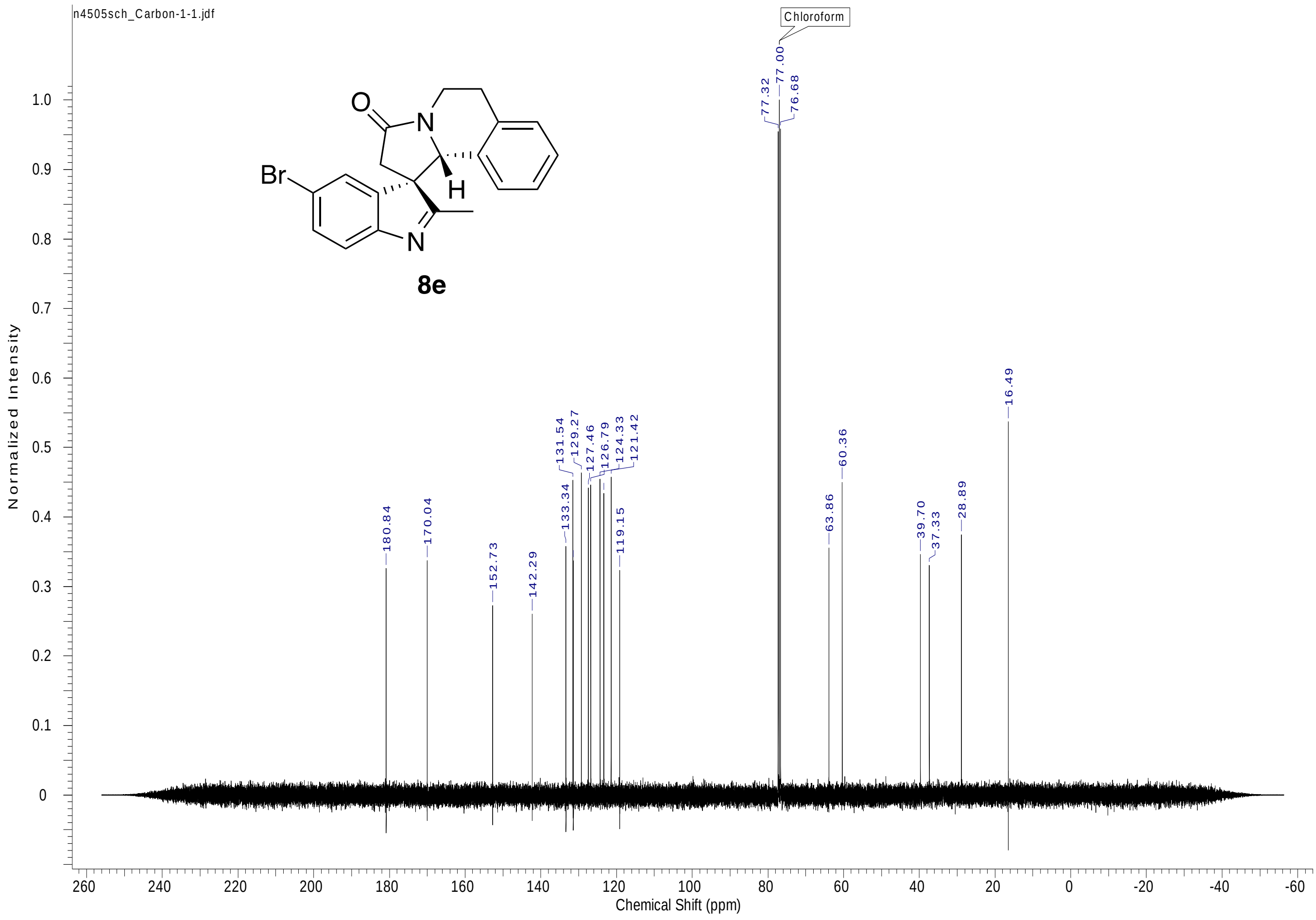
n4505sch_Carbon-1-1.jdf
O
N
Br
H
N
8e
Chloroform
77.32
77.00
76.68
180.84
170.04
152.73
142.29
133.34
131.54
129.27
127.46
126.79
124.33
121.42
119.15
63.86
60.36
39.70
37.33
28.89
16.49
Normalized Intensity
Chemical Shift (ppm)

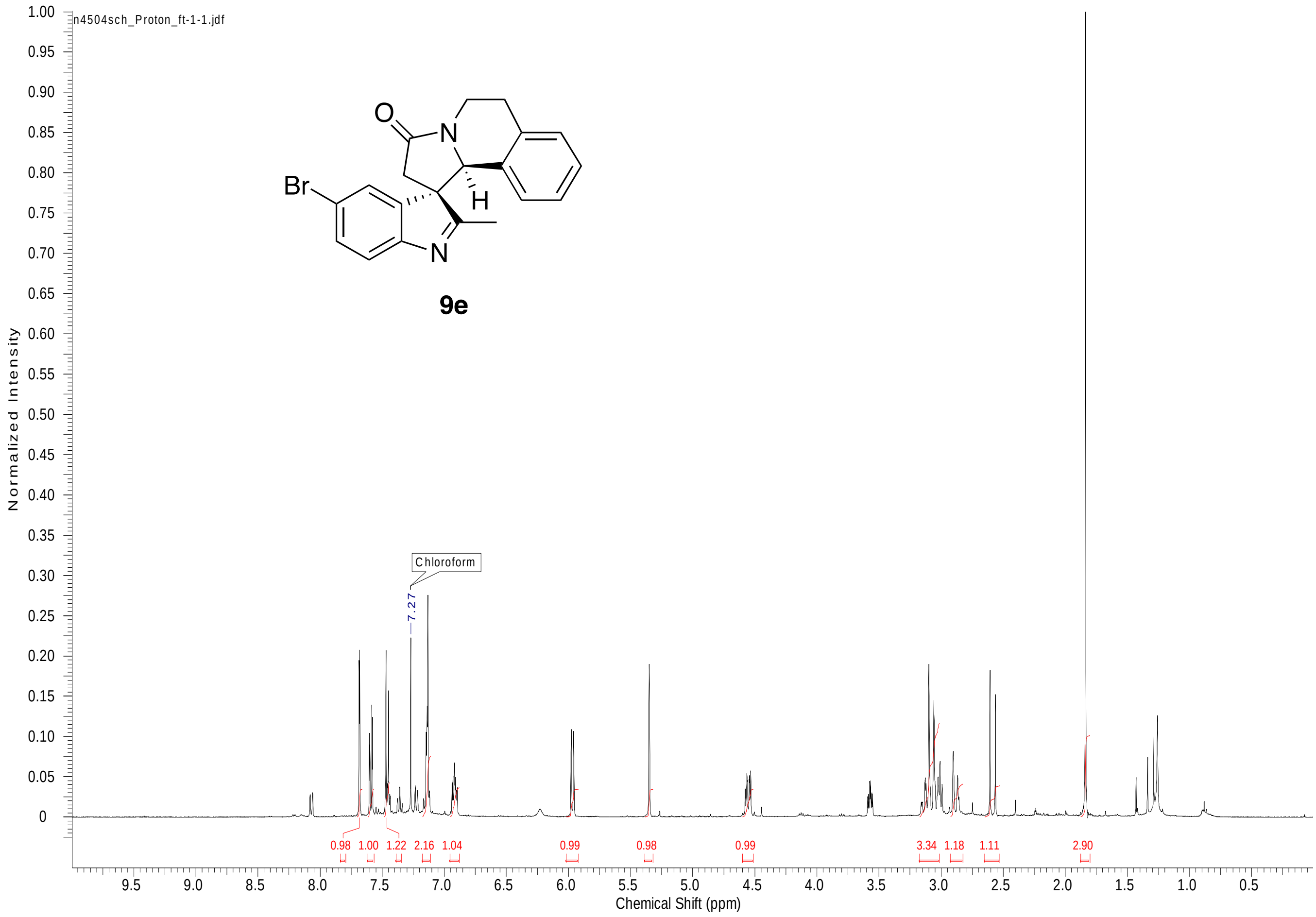
n4504sch_Proton_ft-1-1.jdf
Br
O
N
H
N
9e
Chloroform
7.27
0.98
1.00
1.22
2.16
1.04
0.99
0.98
0.99
3.34
1.18
1.11
2.90
Normalized Intensity
Chemical Shift (ppm)

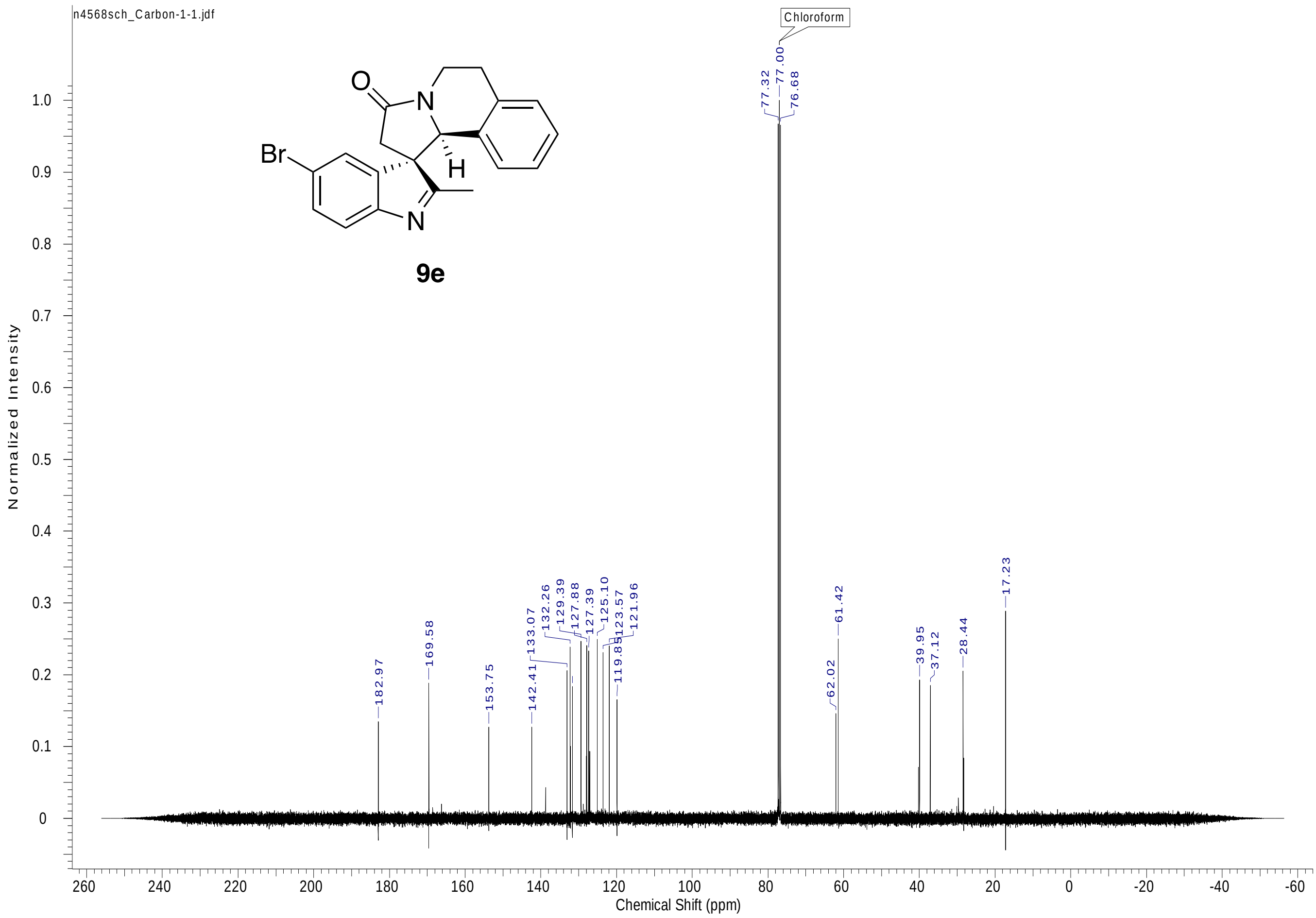
n4568sch_Carbon-1-1.jdf
Br
O
N
H
N
9e
Chloroform
77.32
77.00
76.68
182.97
169.58
153.75
142.41
133.07
132.26
129.39
127.88
127.39
125.10
123.57
121.96
119.85
62.02
61.42
39.95
37.12
28.44
17.23
Normalized Intensity
Chemical Shift (ppm)

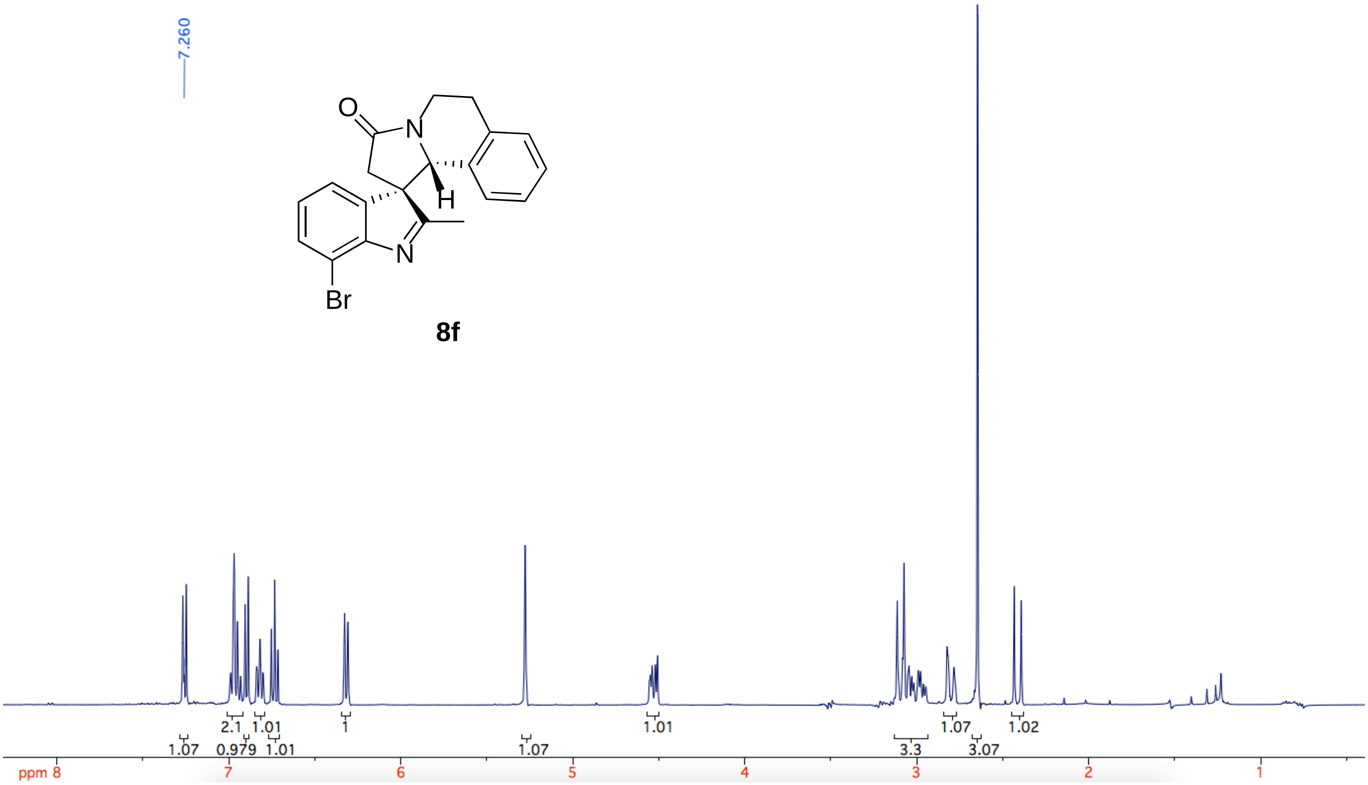
7.260
8f
ppm 8
7
6
5
4
3
2
1

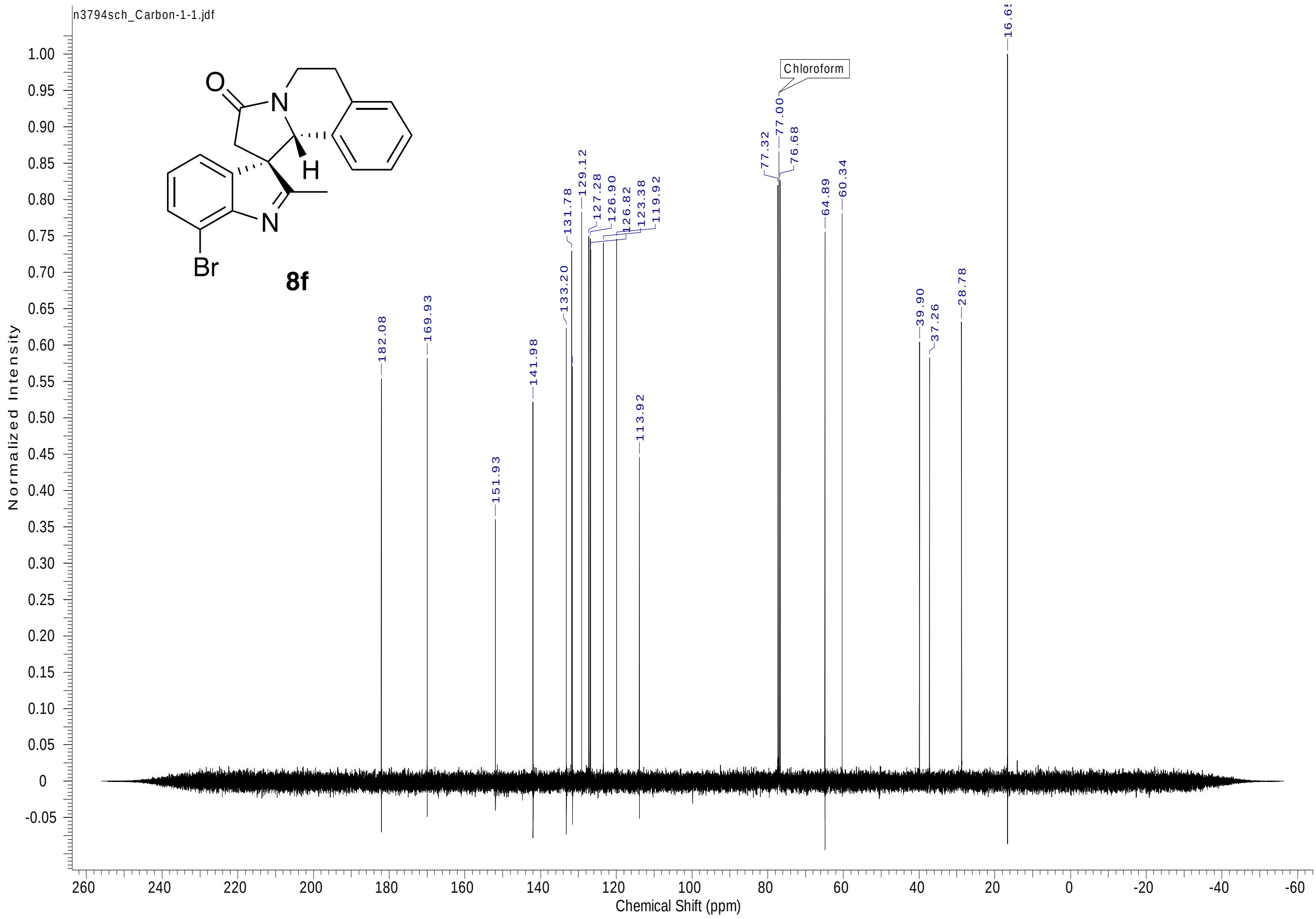
n3794sch_Carbon-1-1.jdf
8f
Chloroform
Normalized Intensity
Chemical Shift (ppm)
182.08
169.93
151.93
141.98
133.20
131.78
129.12
127.28
126.90
126.82
123.38
119.92
113.92
77.32
77.00
76.68
64.89
60.34
39.90
37.26
28.78

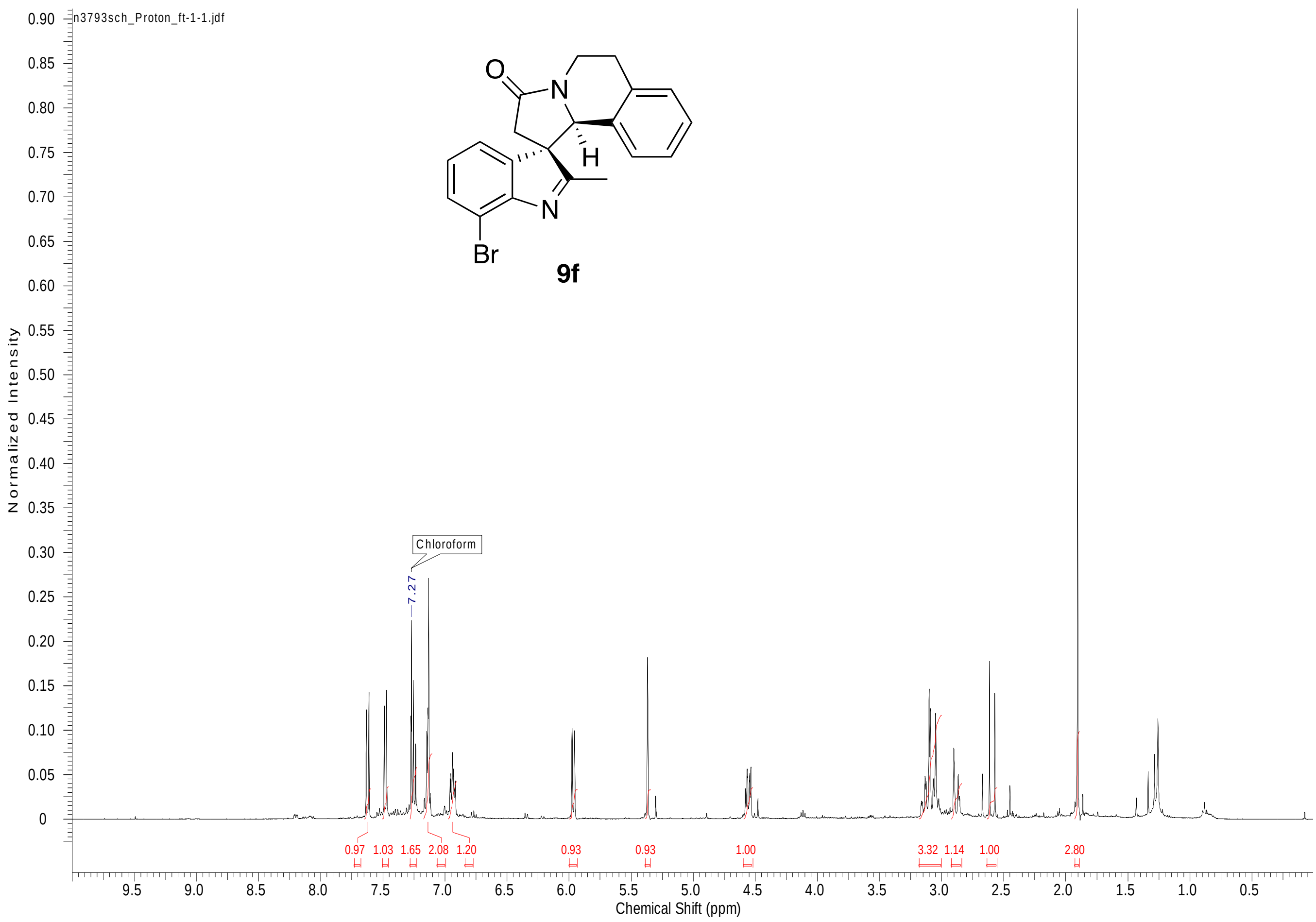
n3793sch_Proton_ft-1-1.jdf
9f
Br
Chloroform
7.27
0.97
1.03
1.65
2.08
1.20
0.93
0.93
1.00
3.32
1.14
1.00
2.80
Normalized Intensity
Chemical Shift (ppm)

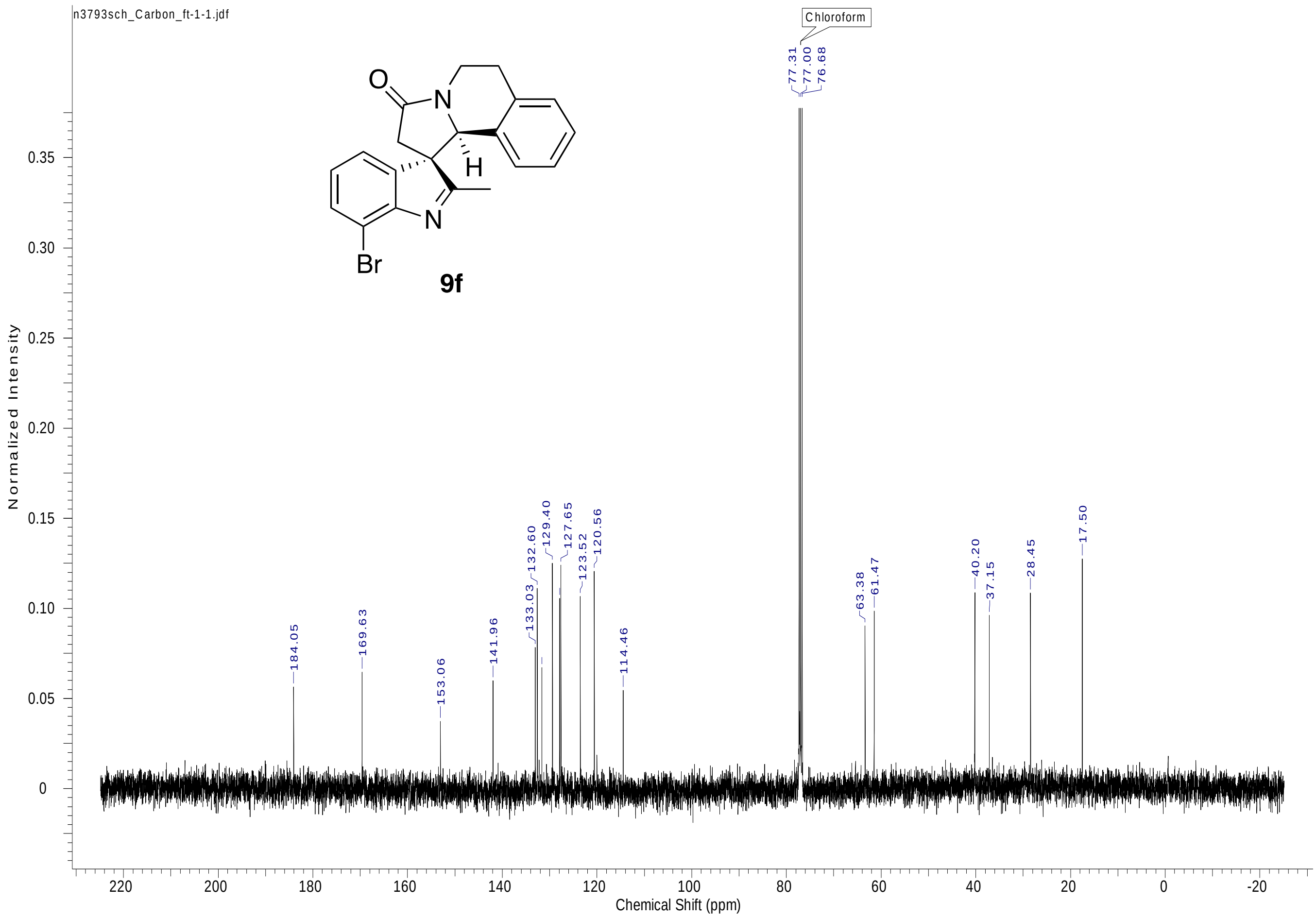
n3793sch_Carbon_ft-1-1.jdf
9f
Br
Chloroform
77.31
77.00
76.68
184.05
169.63
153.06
141.96
133.03
132.60
129.40
127.65
123.52
120.56
114.46
63.38
61.47
40.20
37.15
28.45
17.50
Normalized Intensity
Chemical Shift (ppm)

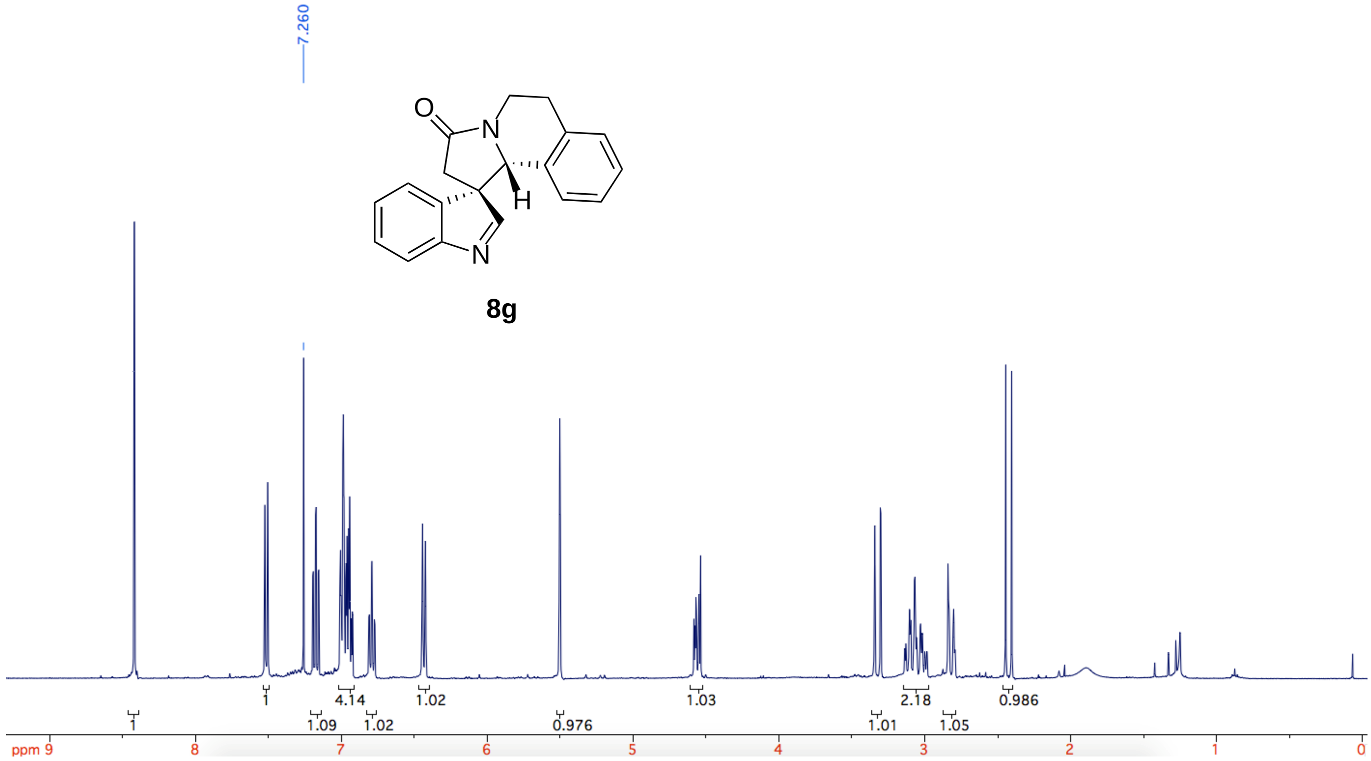
7.260
O
N
H
N
8g
1
1
4.14
1.09
1.02
1.02
0.976
1.03
1.01
2.18
1.05
0.986
ppm 9
8
7
6
5
4
3
2
1
0

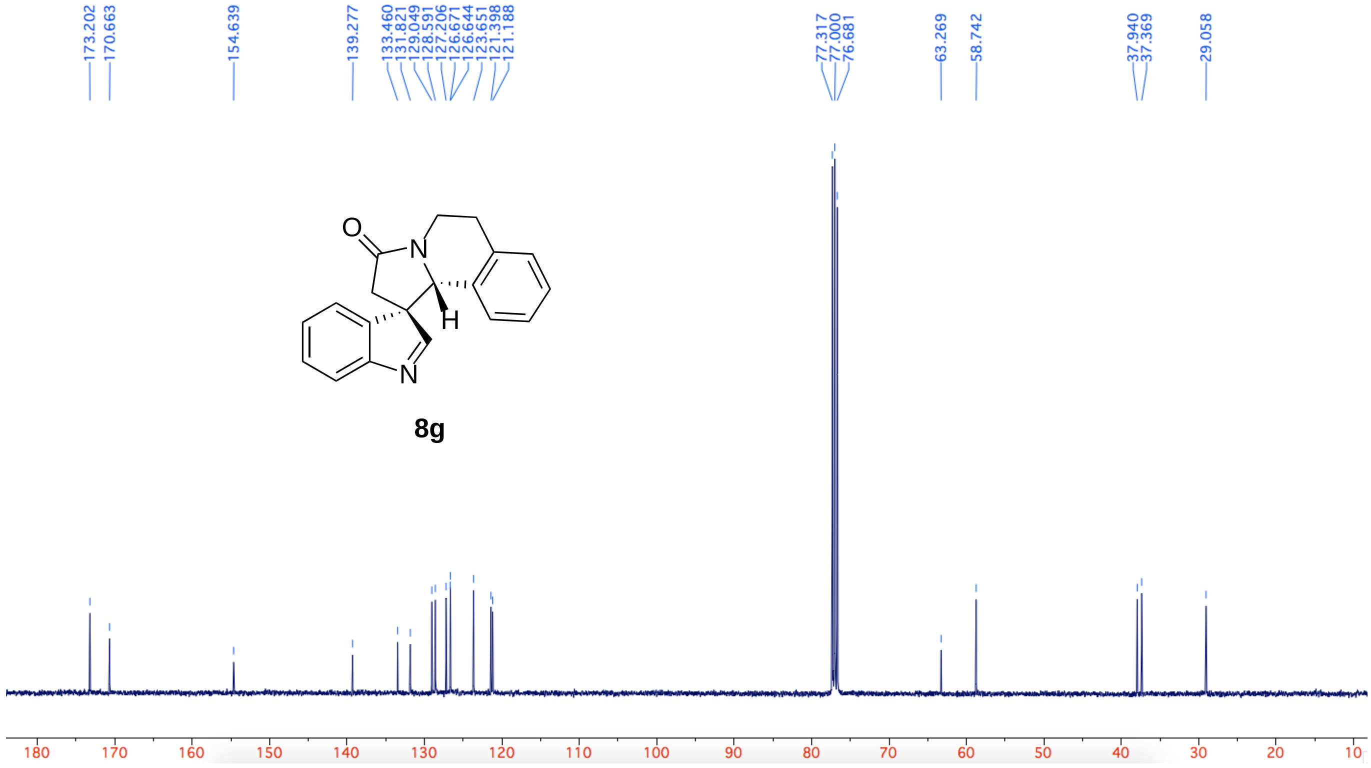
173.202
170.663
154.639
139.277
133.460
131.821
129.049
128.591
127.206
126.671
126.644
123.651
121.398
121.188
77.317
77.000
76.681
63.269
58.742
37.940
37.369
29.058
O
N
H
N
8g
180
170
160
150
140
130
120
110
100
90
80
70
60
50
40
30
20
10

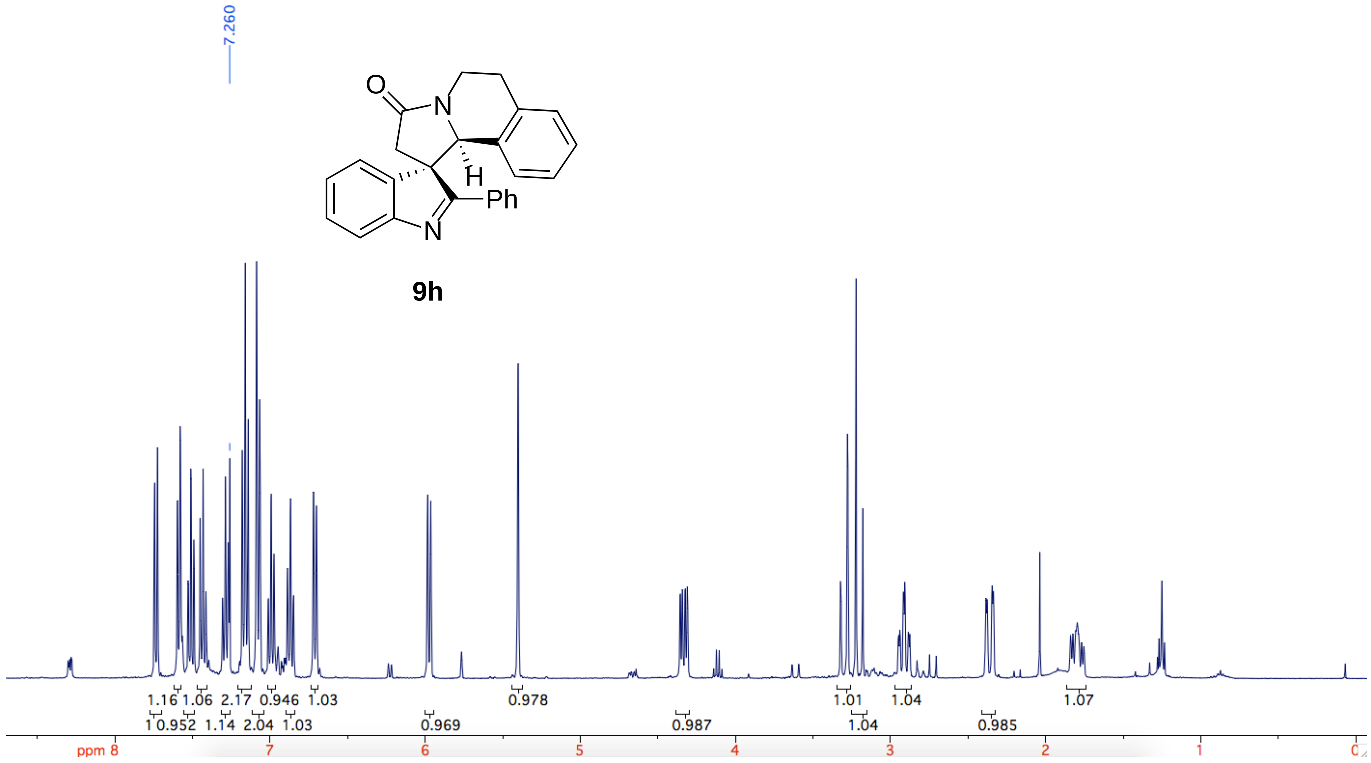
7.260
9h
1.16 1.06 2.17 0.946 1.03
1 0.952 1.14 2.04 1.03
0.969
0.978
0.987
1.01
1.04
1.04
0.985
1.07
ppm 8 7 6 5 4 3 2 1 0

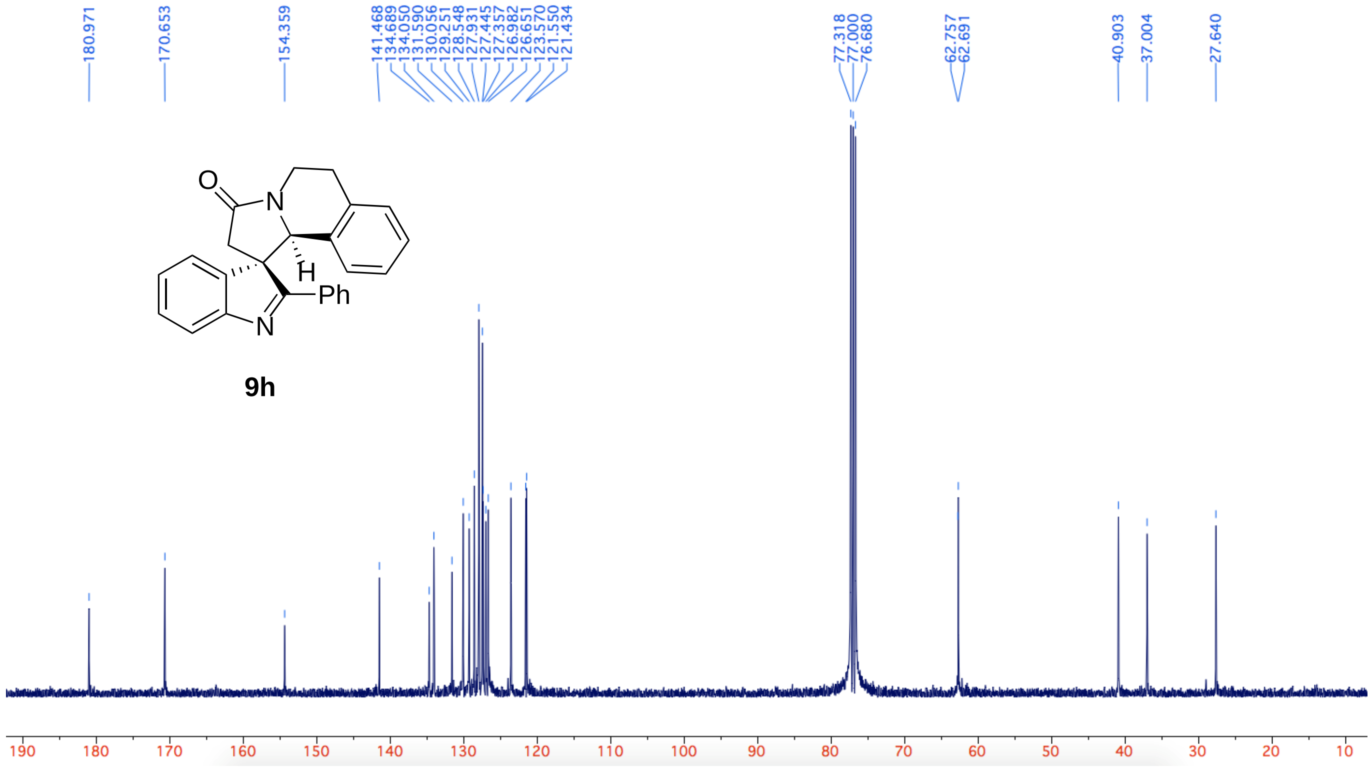
180.971
170.653
154.359
141.468
134.689
134.050
131.590
130.056
129.251
128.548
127.931
127.445
127.357
126.982
126.651
123.570
121.550
121.434
77.318
77.000
76.680
62.757
62.691
40.903
37.004
27.640
9h
190 180 170 160 150 140 130 120 110 100 90 80 70 60 50 40 30 20 10

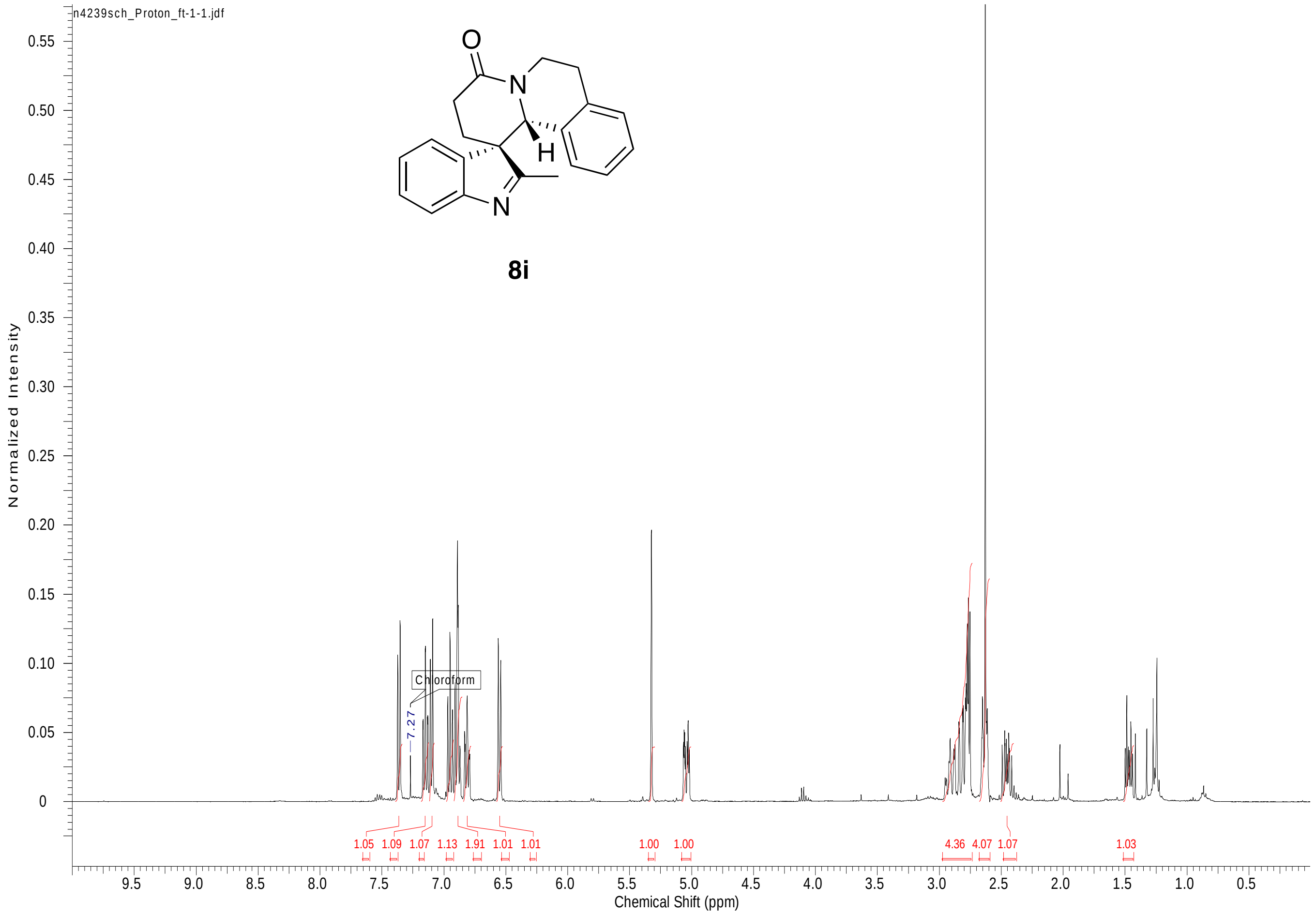

n4239sch_Proton_ft-1-1.jdf
Normalized Intensity
Chemical Shift (ppm)
Chloroform
7.27
1.05 1.09 1.07 1.13 1.91 1.01 1.01 1.00 1.00 4.36 4.07 1.07 1.03

8i

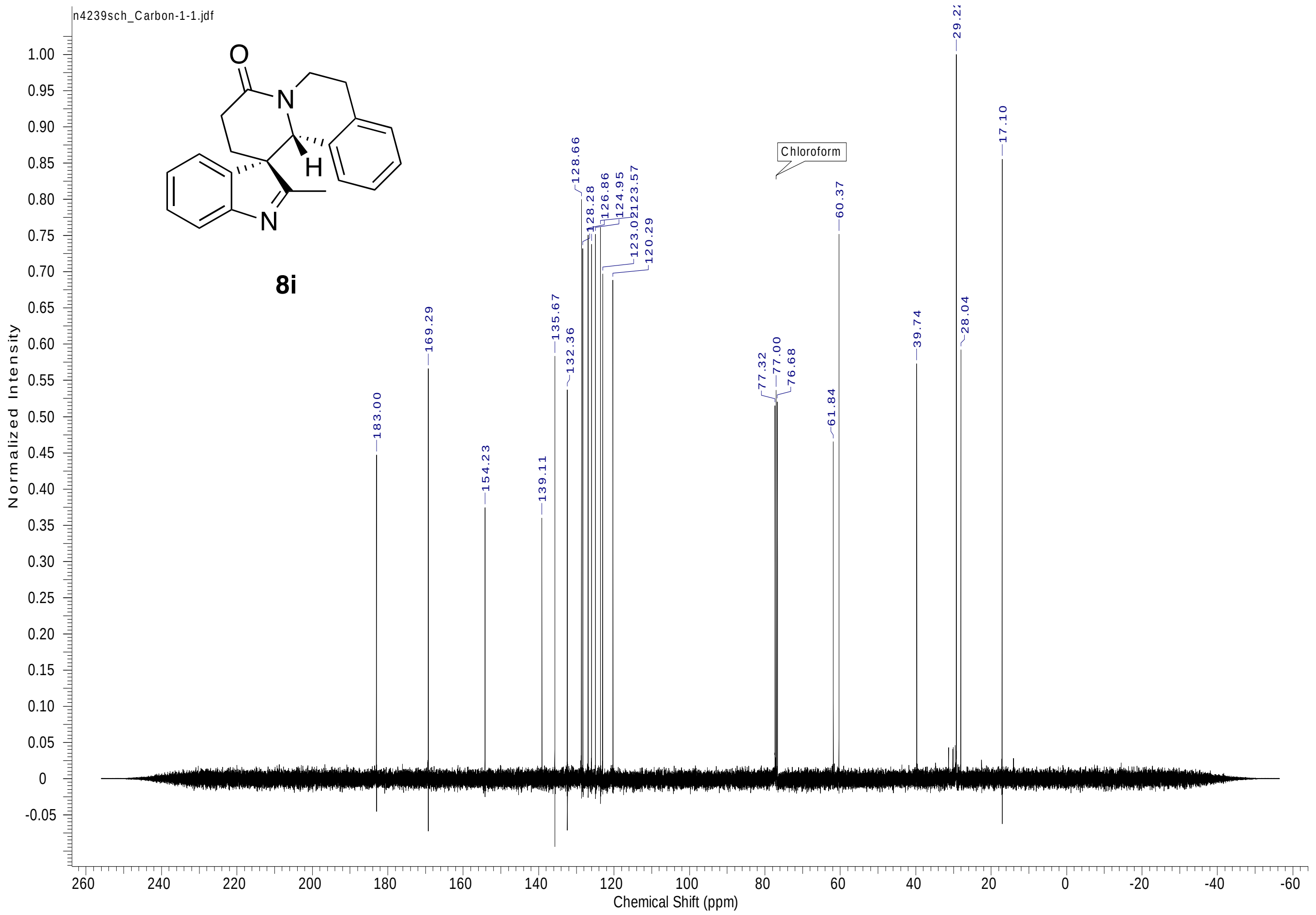

n4239sch_Carbon-1-1.jdf
Normalized Intensity
Chemical Shift (ppm)
Chloroform
183.00
169.29
154.23
139.11
135.67
132.36
128.66
128.28
126.86
124.95
123.02
123.57
120.29
77.32
77.00
76.68
61.84
60.37
39.74
28.04
17.10

8i

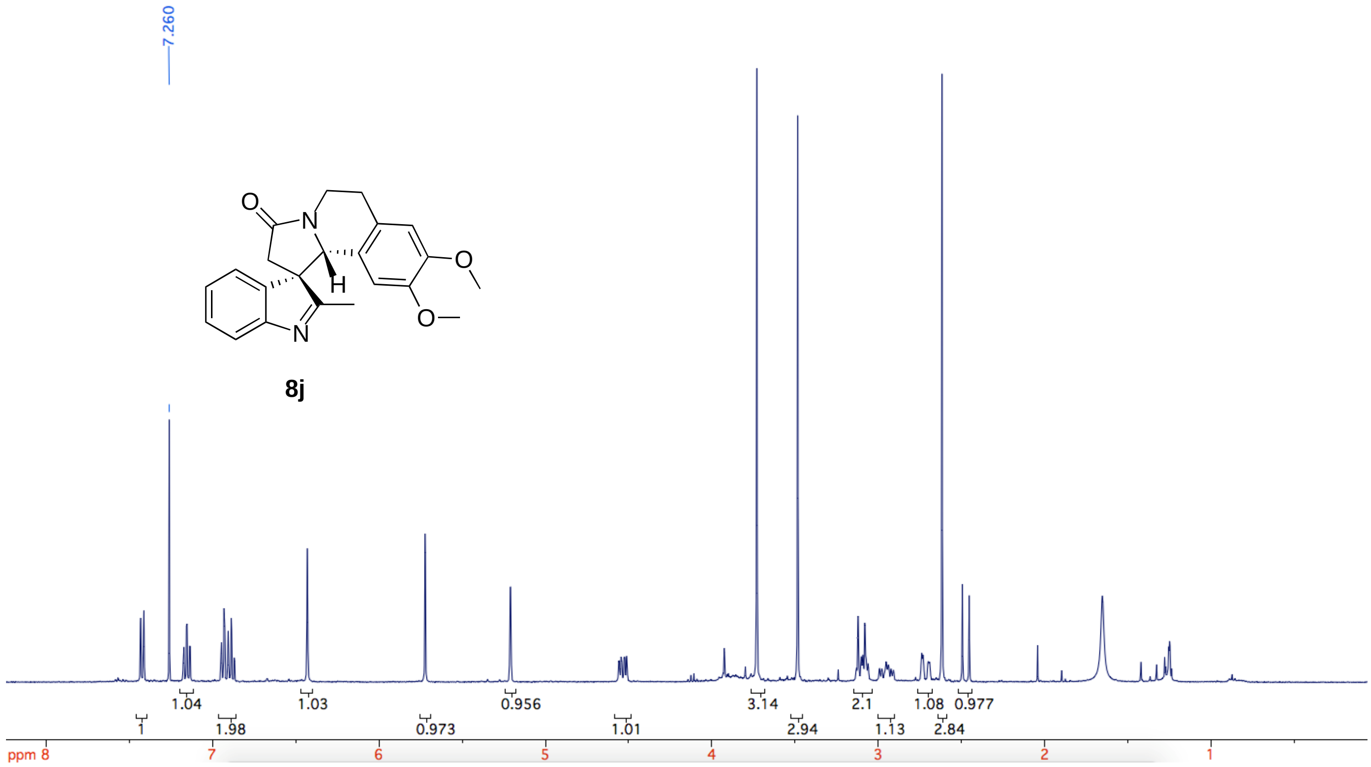

7.260
O
N
H
O
O
N
8j
1
1.04
1.98
1.03
0.973
0.956
1.01
3.14
2.94
2.1
1.13
1.08
2.84
0.977
ppm 8
7
6
5
4
3
2
1

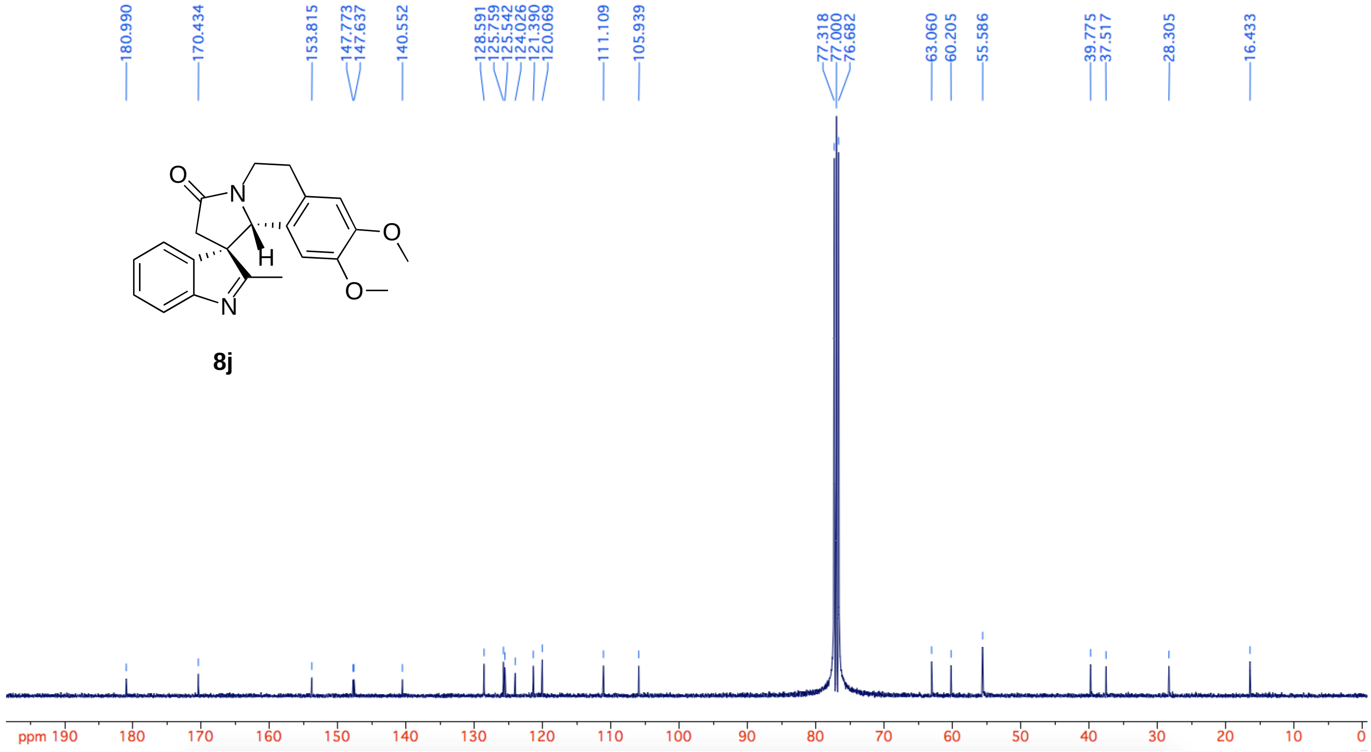

180.990
170.434
153.815
147.773
147.637
140.552
128.591
125.759
125.542
124.026
121.390
120.069
111.109
105.939
77.318
77.000
76.682
63.060
60.205
55.586
39.775
37.517
28.305
16.433
O
N
H
O
O
N
8j
ppm 190
180
170
160
150
140
130
120
110
100
90
80
70
60
50
40
30
20
10
0

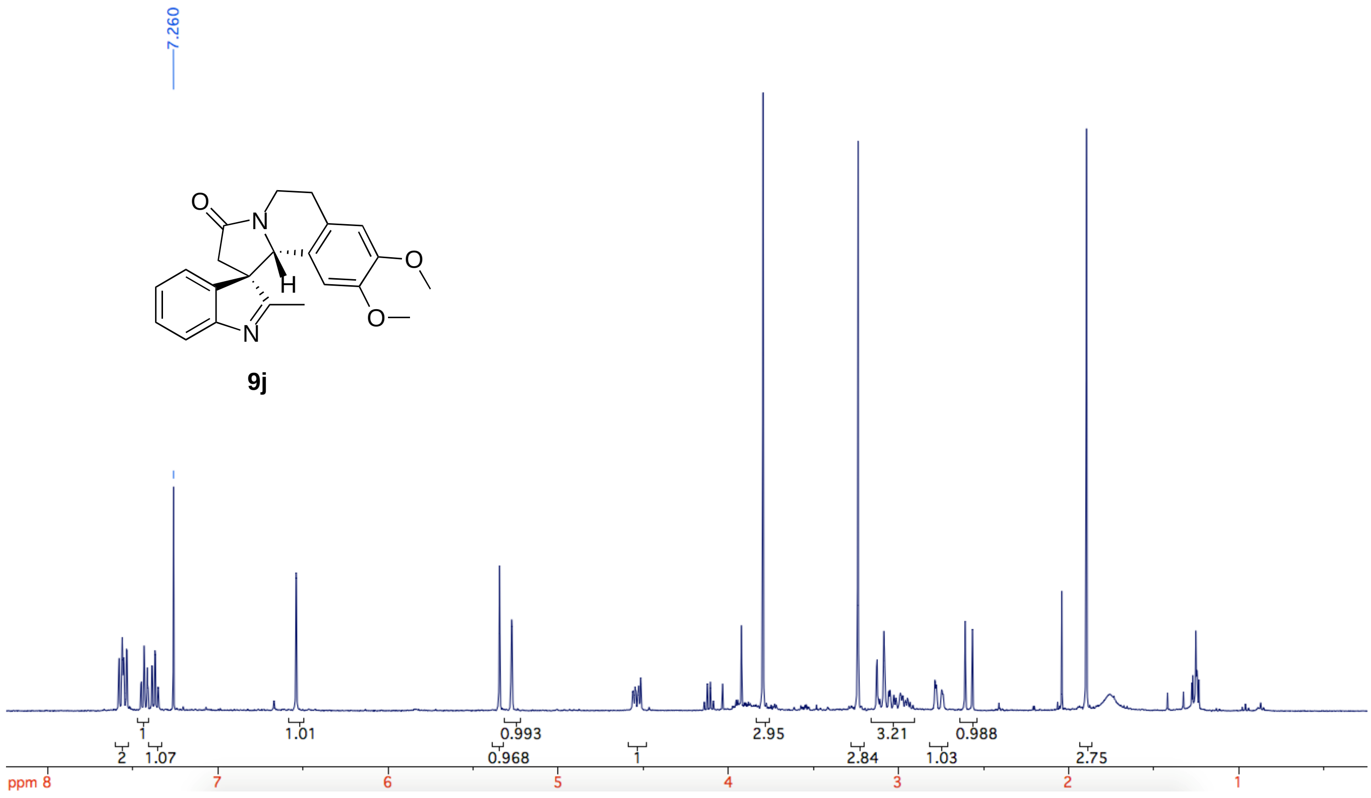
7.260
9j
2
1
1.07
1.01
0.993
0.968
1
2.95
2.84
3.21
1.03
0.988
2.75
ppm 8
7
6
5
4
3
2
1

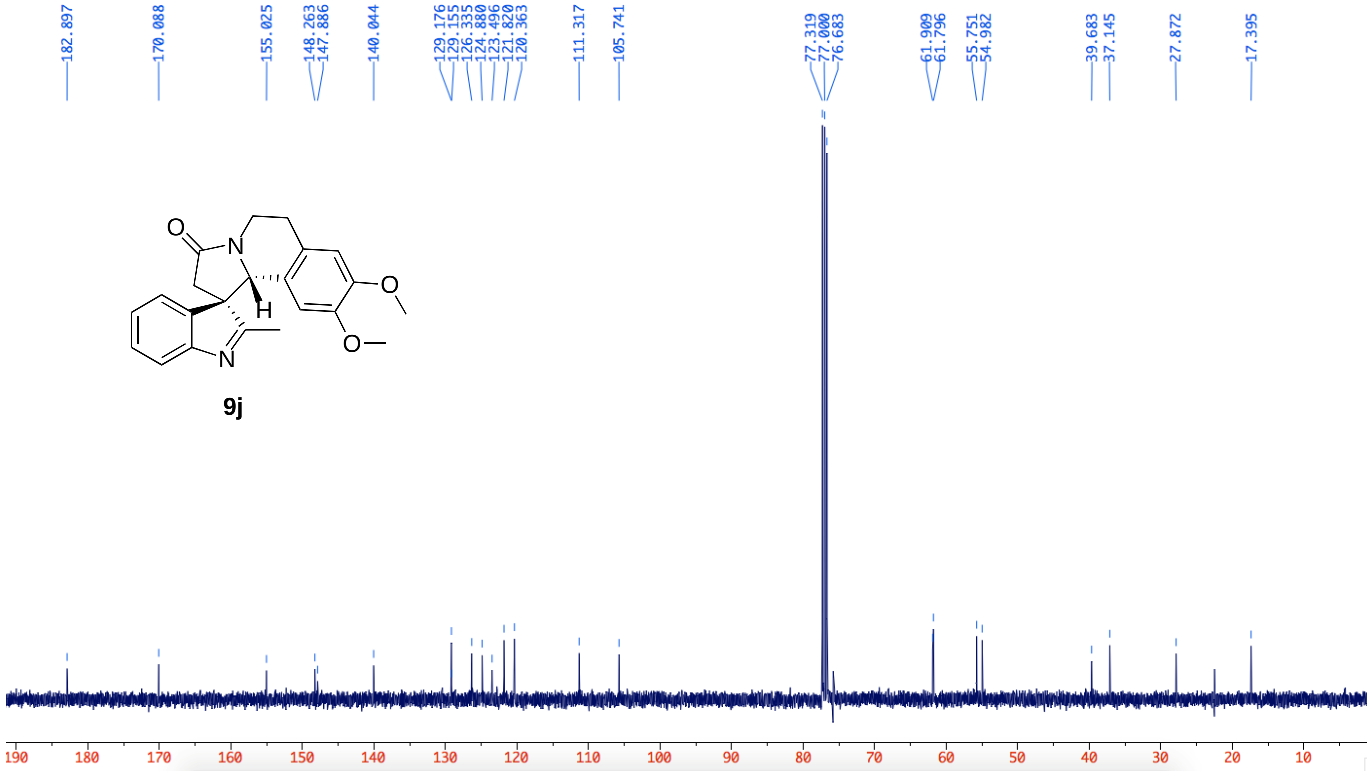
182.897
170.088
155.025
148.263
147.886
140.044
129.176
129.155
126.335
124.880
123.496
121.820
120.363
111.317
105.741
77.319
77.000
76.683
61.909
61.796
55.751
54.982
39.683
37.145
27.872
17.395
9j
190
180
170
160
150
140
130
120
110
100
90
80
70
60
50
40
30
20
10

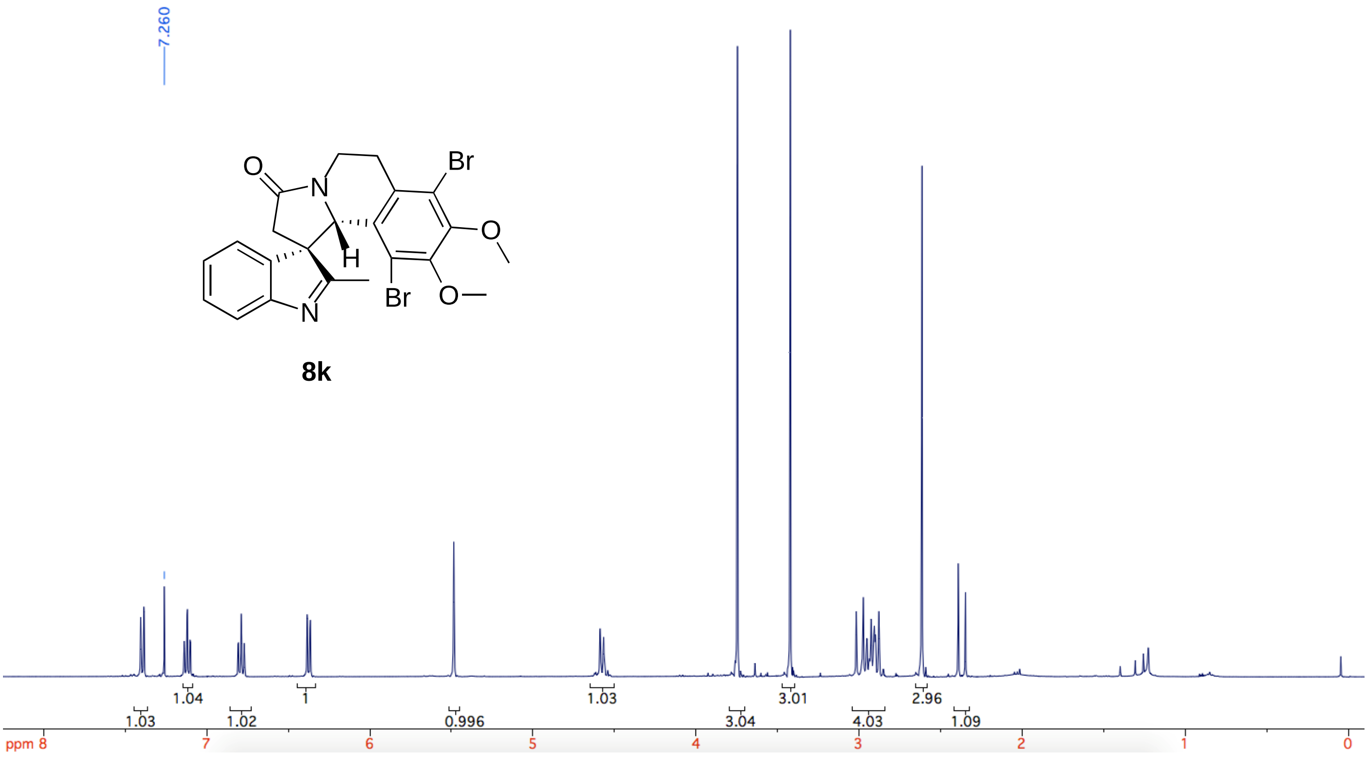
7.260
O
N
Br
O
H
Br
O
N
8k
1.03
1.04
1.02
1
0.996
1.03
3.04
3.01
4.03
2.96
1.09
ppm 8
7
6
5
4
3
2
1
0

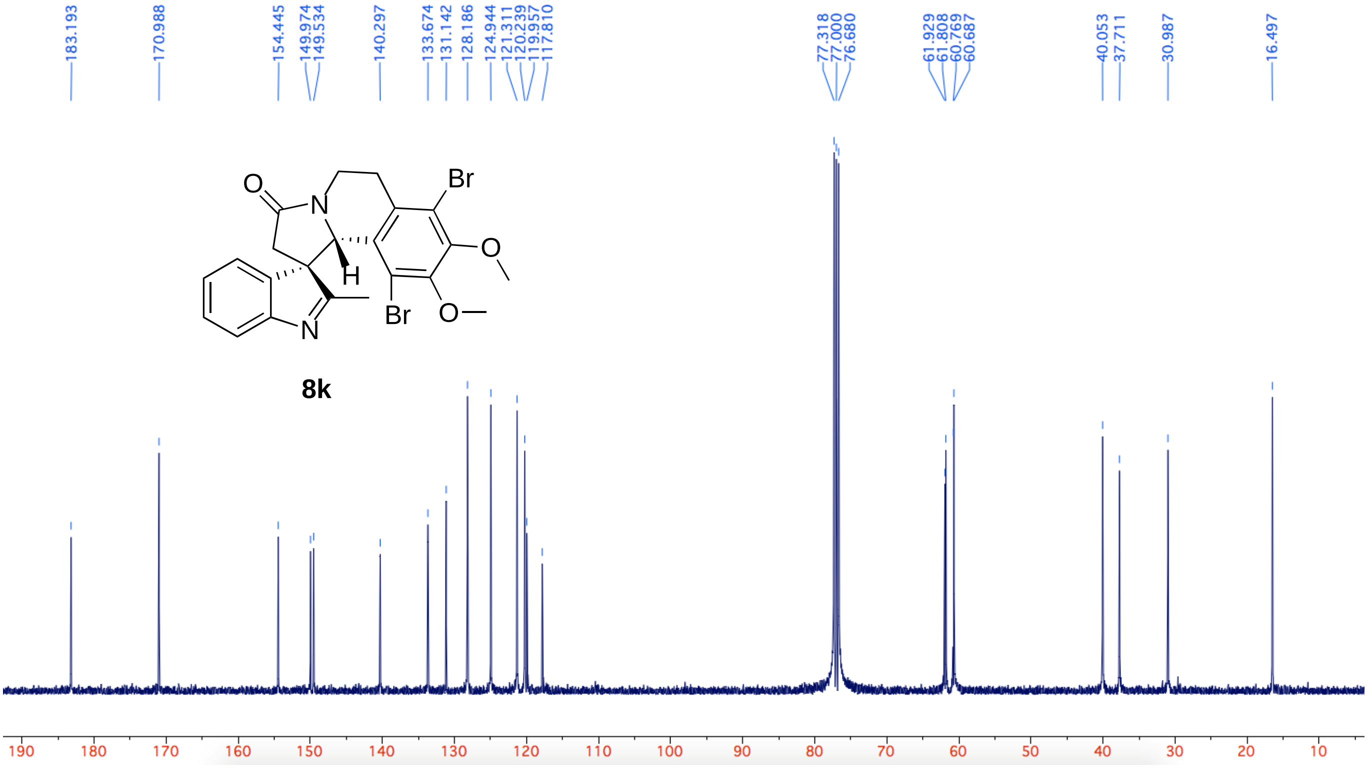
183.193
170.988
154.445
149.974
149.534
140.297
133.674
131.142
128.186
124.944
121.311
120.239
119.957
117.810
77.318
77.000
76.680
61.929
61.808
60.769
60.687
40.053
37.711
30.987
16.497
O
N
Br
O
H
Br
O
N
8k
190
180
170
160
150
140
130
120
110
100
90
80
70
60
50
40
30
20
10

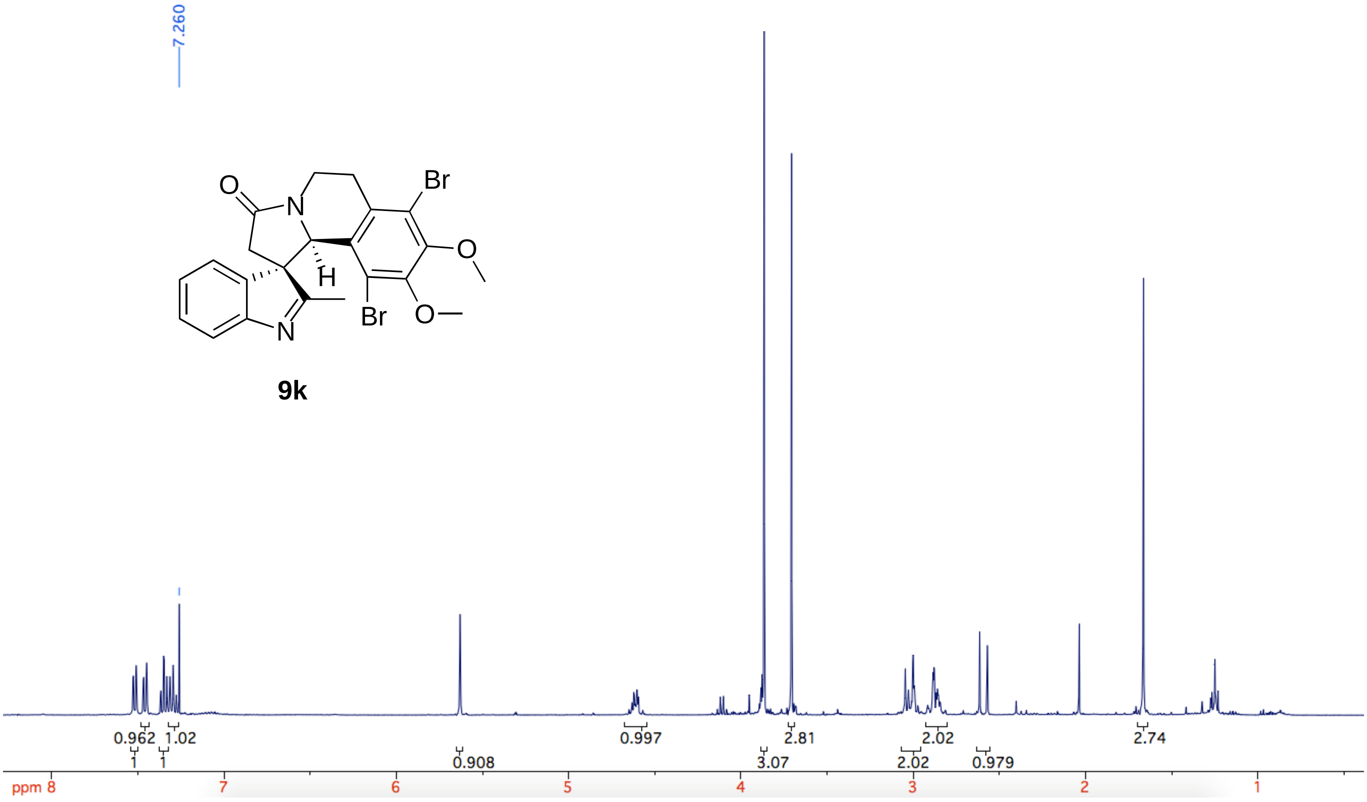
7.260
Br
O
N
H
O
N
Br
O
9k
0.962
1
1.02
1
0.908
0.997
3.07
2.81
2.02
2.02
0.979
2.74
ppm 8
7
6
5
4
3
2
1

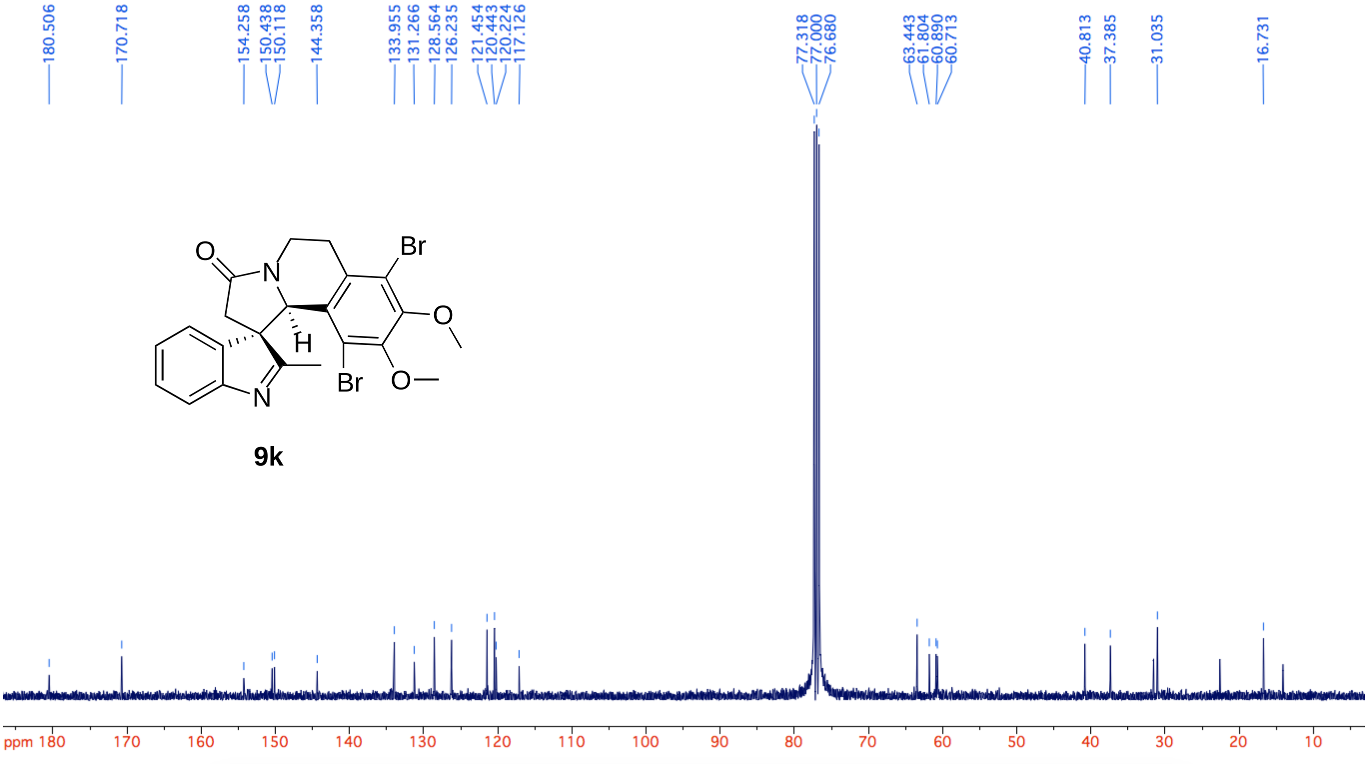
180.506
170.718
154.258
150.438
150.118
144.358
133.955
131.266
128.564
126.235
121.454
120.443
120.224
117.126
77.318
77.000
76.680
63.443
61.804
60.890
60.713
40.813
37.385
31.035
16.731
9k
ppm 180
170
160
150
140
130
120
110
100
90
80
70
60
50
40
30
20
10

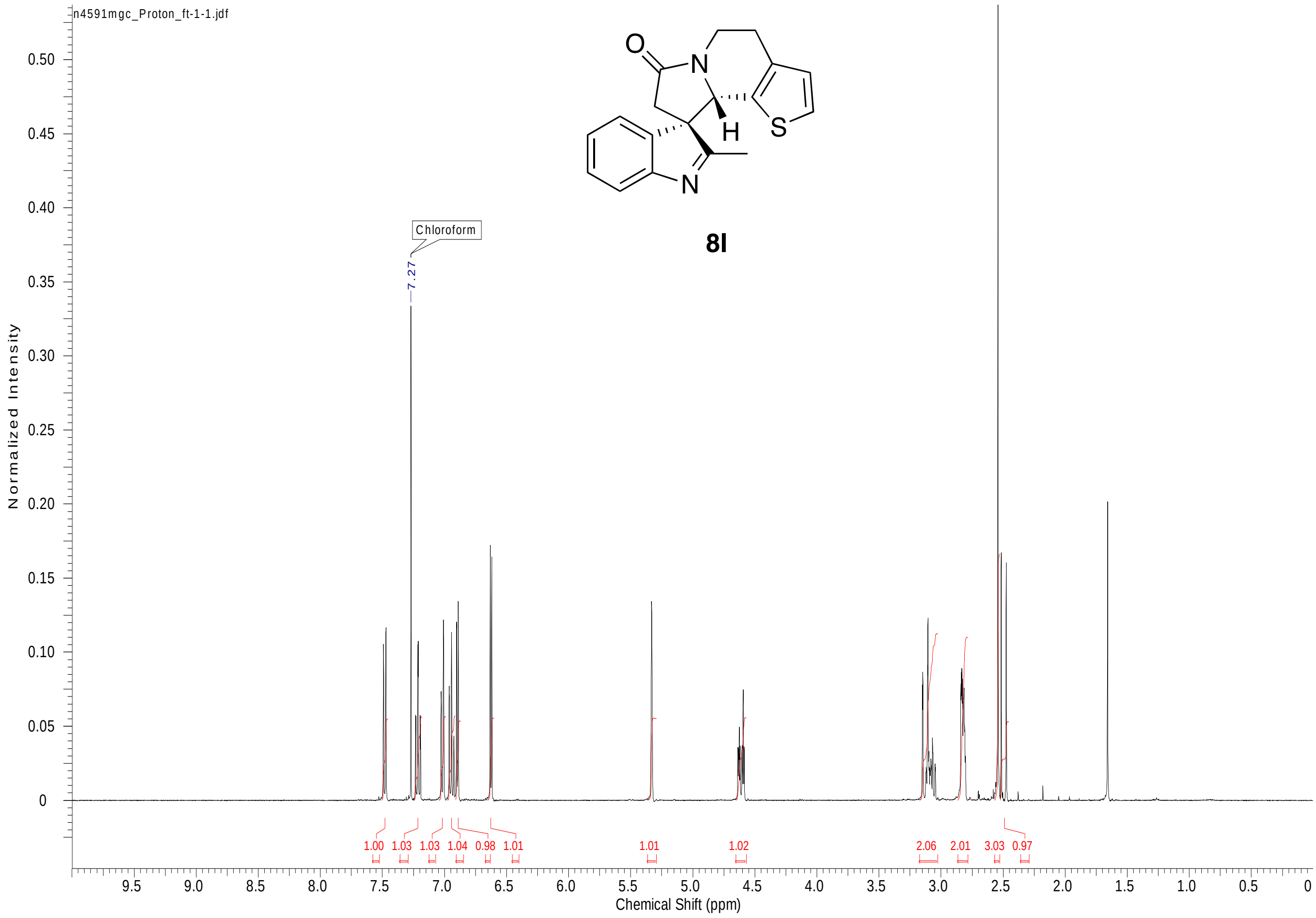
n4591mgc_Proton_ft-1-1.jdf
Chloroform
7.27
8l
Normalized Intensity
Chemical Shift (ppm)
1.00
1.03
1.03
1.04
0.98
1.01
1.01
1.02
2.06
2.01
3.03
0.97

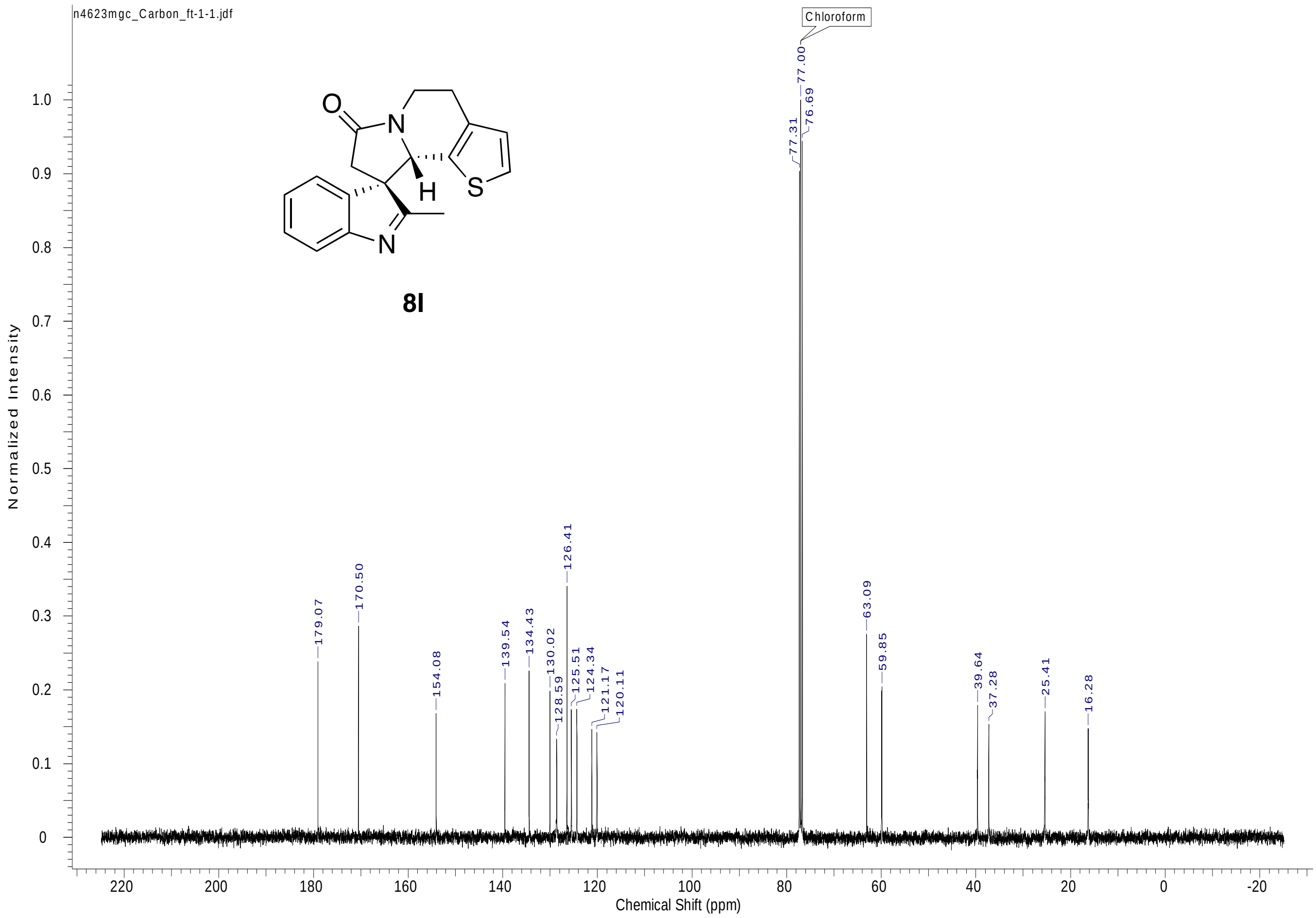
n4623mgc_Carbon_ft-1-1.jdf
Chloroform
77.31
77.00
76.69
8l
Normalized Intensity
Chemical Shift (ppm)
179.07
170.50
154.08
139.54
134.43
130.02
128.59
126.41
125.51
124.34
121.17
120.11
63.09
59.85
39.64
37.28
25.41
16.28

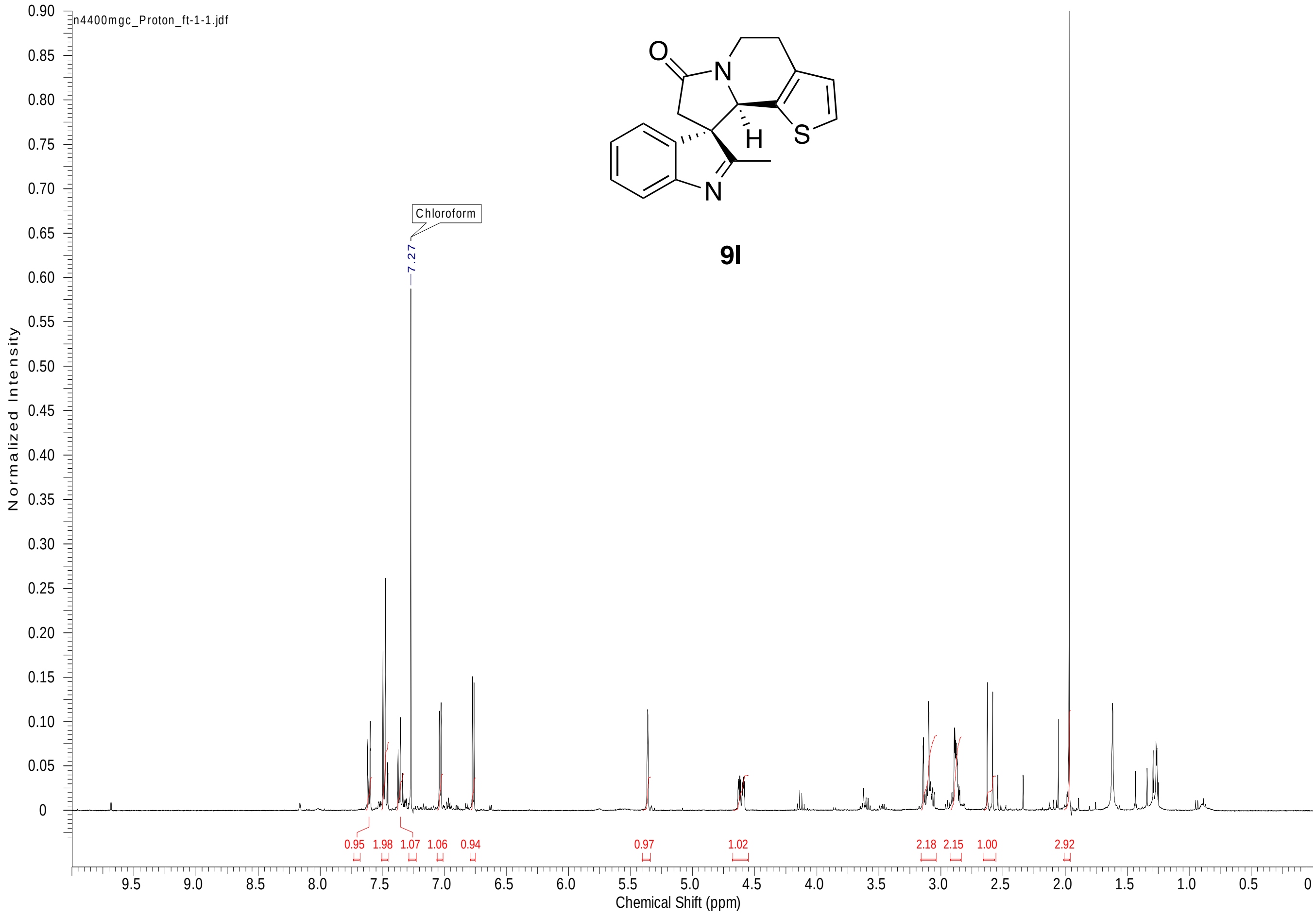
n4400mgc_Proton_ft-1-1.jdf
Chloroform
7.27
9l
Normalized Intensity
Chemical Shift (ppm)
0.95
1.98
1.07
1.06
0.94
0.97
1.02
2.18
2.15
1.00
2.92

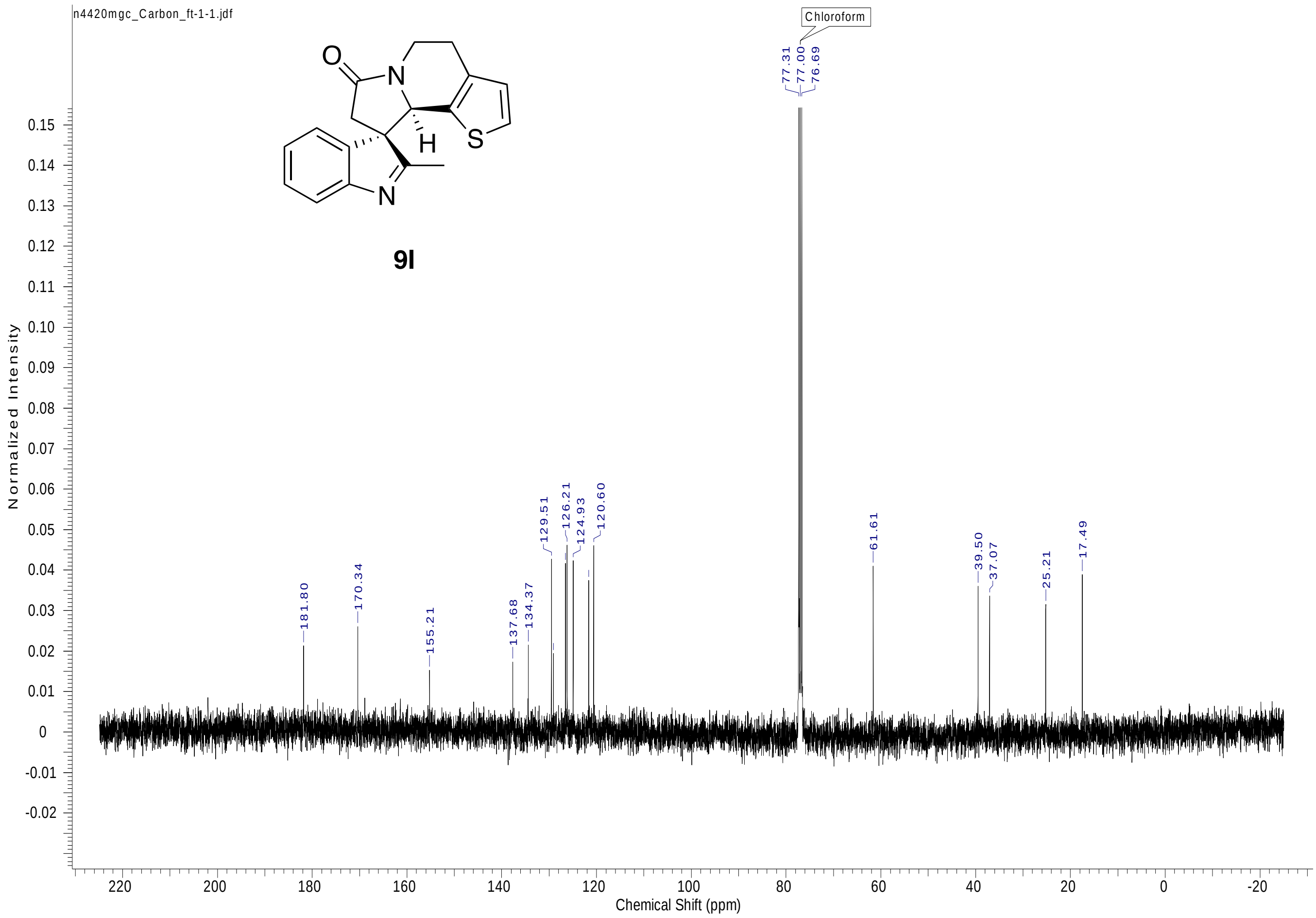
n4420mgc_Carbon_ft-1-1.jdf
Chloroform
77.31
77.00
76.69
9l
Normalized Intensity
Chemical Shift (ppm)
181.80
170.34
155.21
137.68
134.37
129.51
126.21
124.93
120.60
61.61
39.50
37.07
25.21
17.49

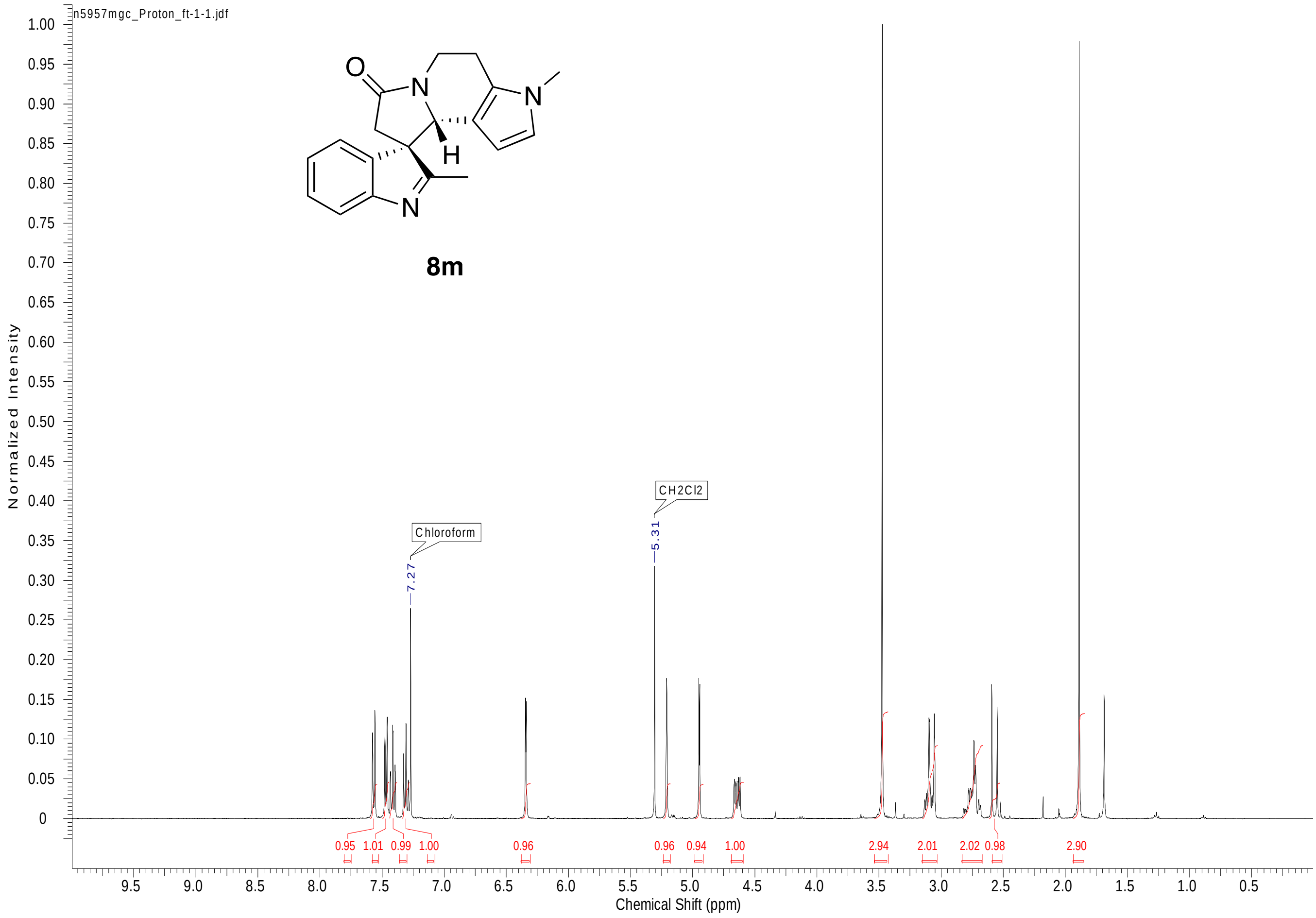
n5957mgc_Proton_ft-1-1.jdf
8m
Chloroform
CH2Cl2
7.27
5.31
0.95 1.01 0.99 1.00 0.96 0.96 0.94 1.00 2.94 2.01 2.02 0.98 2.90
Normalized Intensity
Chemical Shift (ppm)

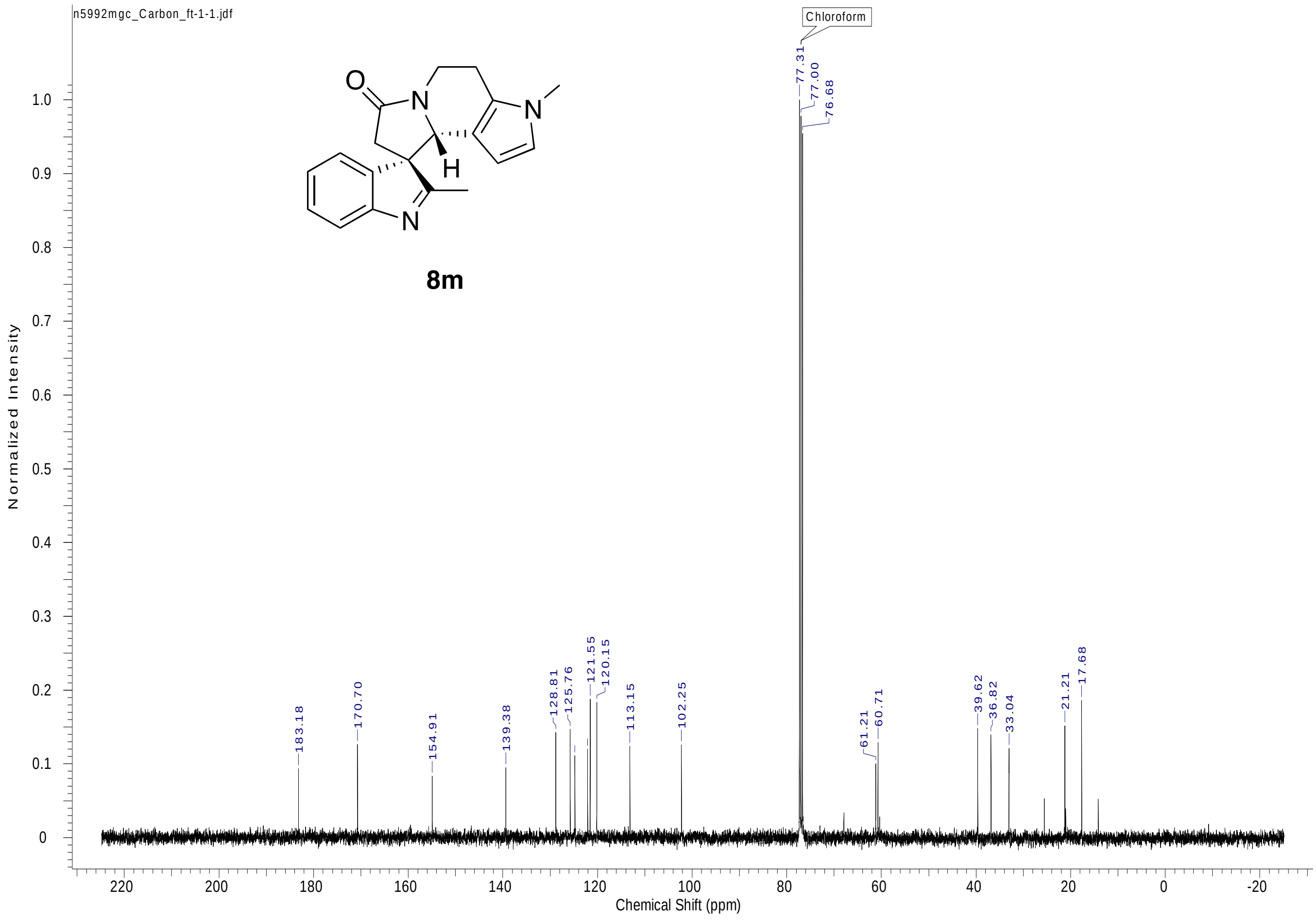
n5992mgc_Carbon_ft-1-1.jdf
8m
Chloroform
77.31
77.00
76.68
183.18
170.70
154.91
139.38
128.81
125.76
121.55
120.15
113.15
102.25
61.21
60.71
39.62
36.82
33.04
21.21
17.68
Normalized Intensity
Chemical Shift (ppm)

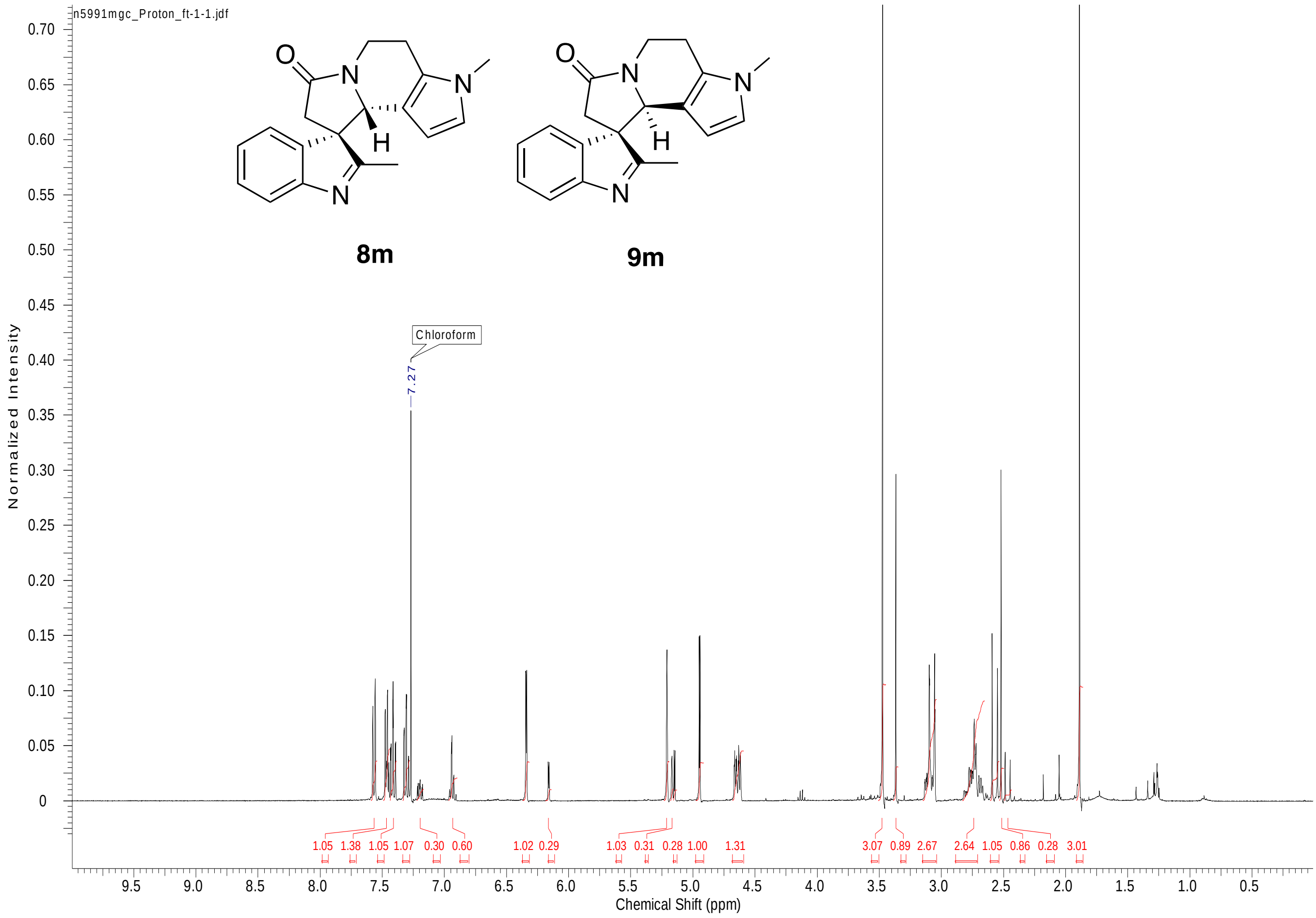

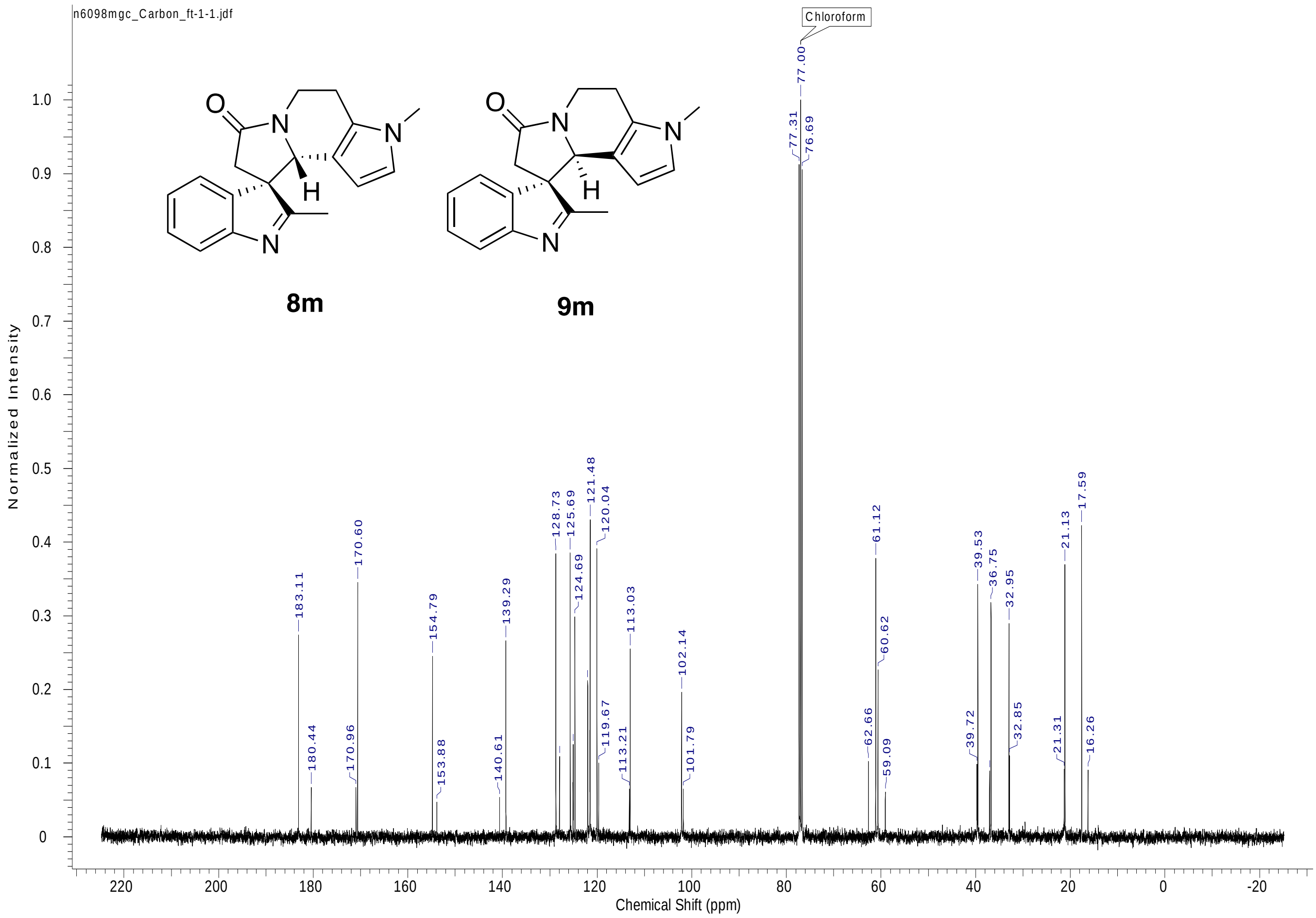

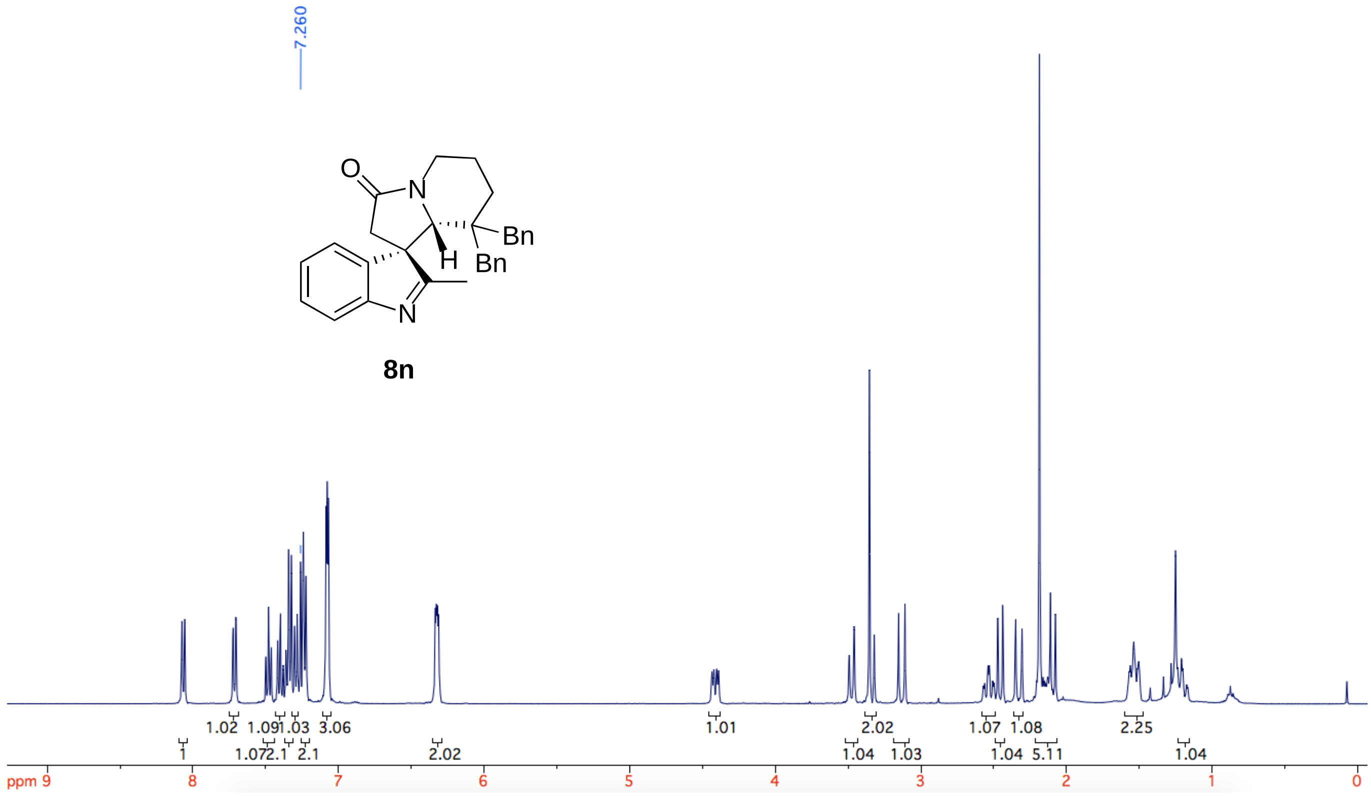

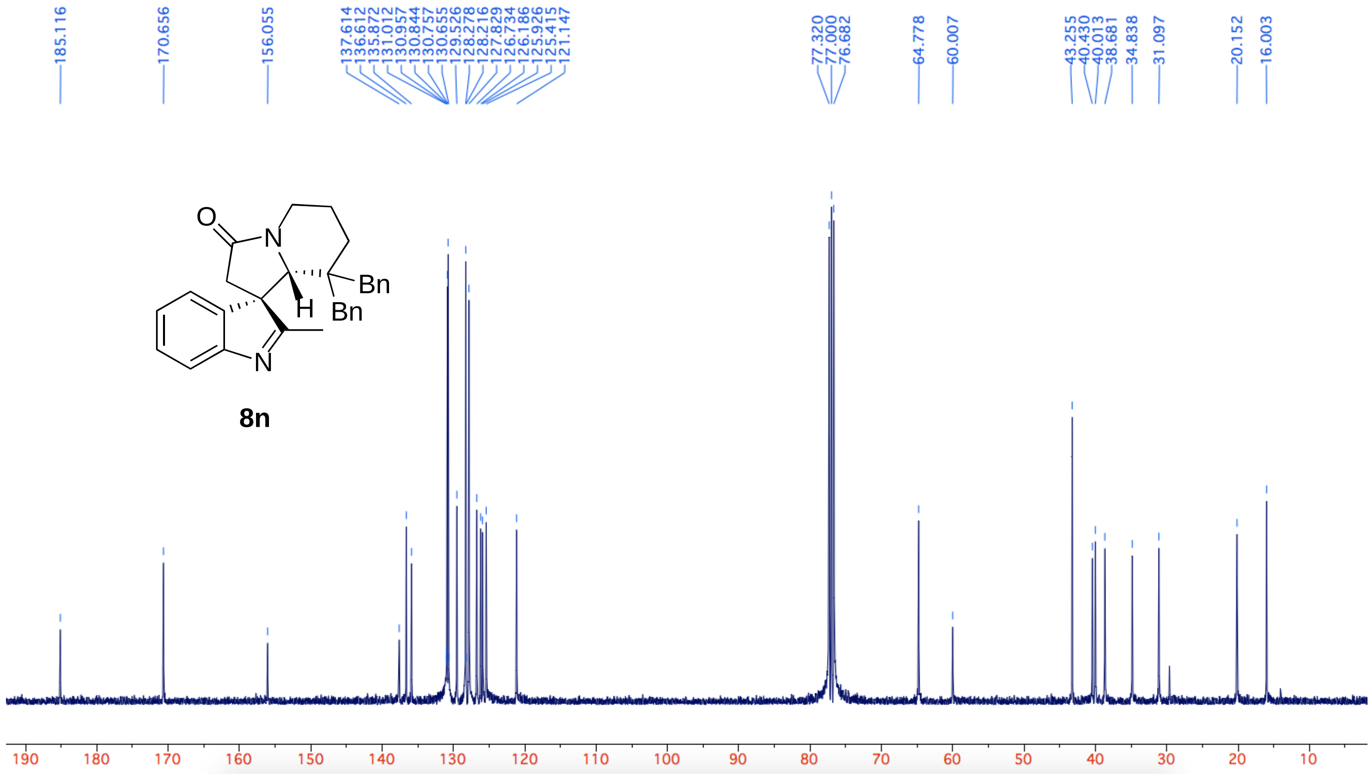

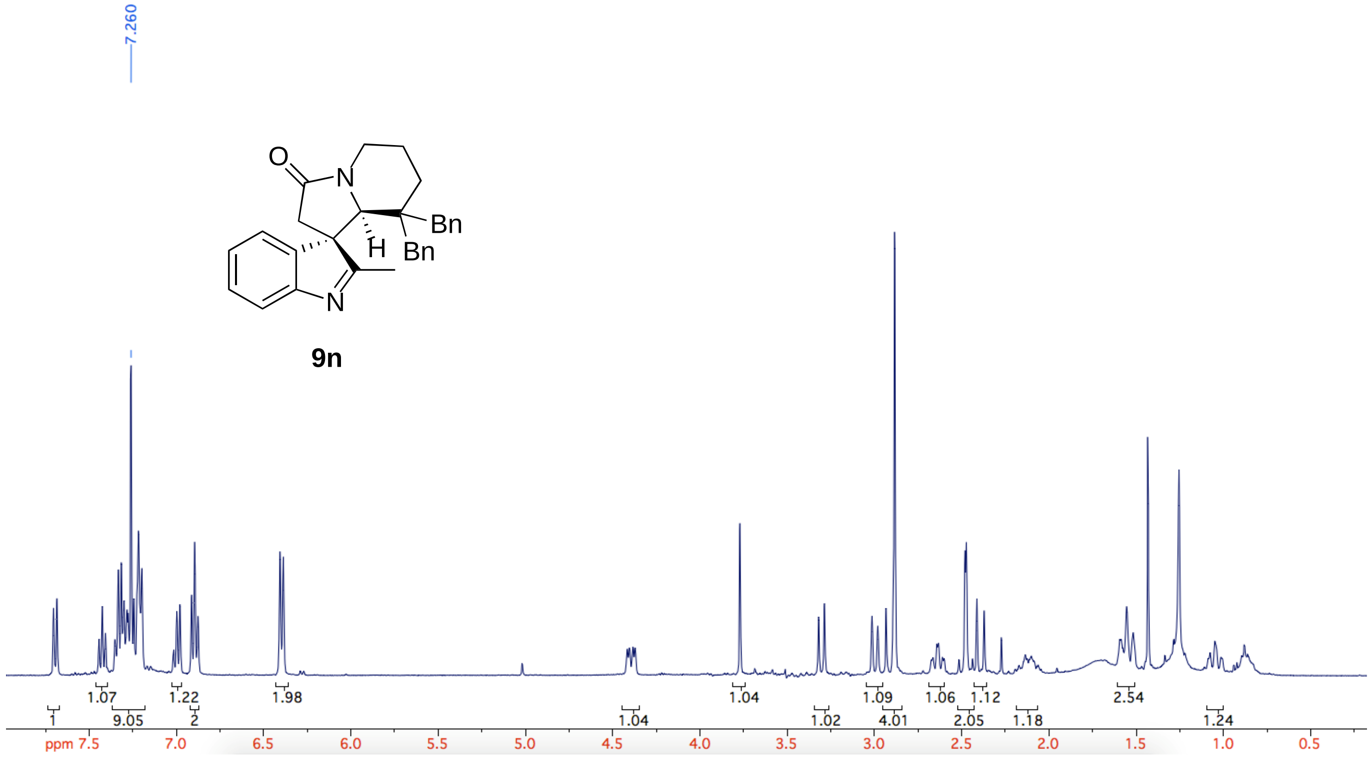
7.260
9n
Bn
Bn
H
1.07
9.05
1.22
2
1
1.98
1.04
1.04
1.02
1.09
4.01
1.06
2.05
1.12
1.18
2.54
1.24
ppm 7.5
7.0
6.5
6.0
5.5
5.0
4.5
4.0
3.5
3.0
2.5
2.0
1.5
1.0
0.5

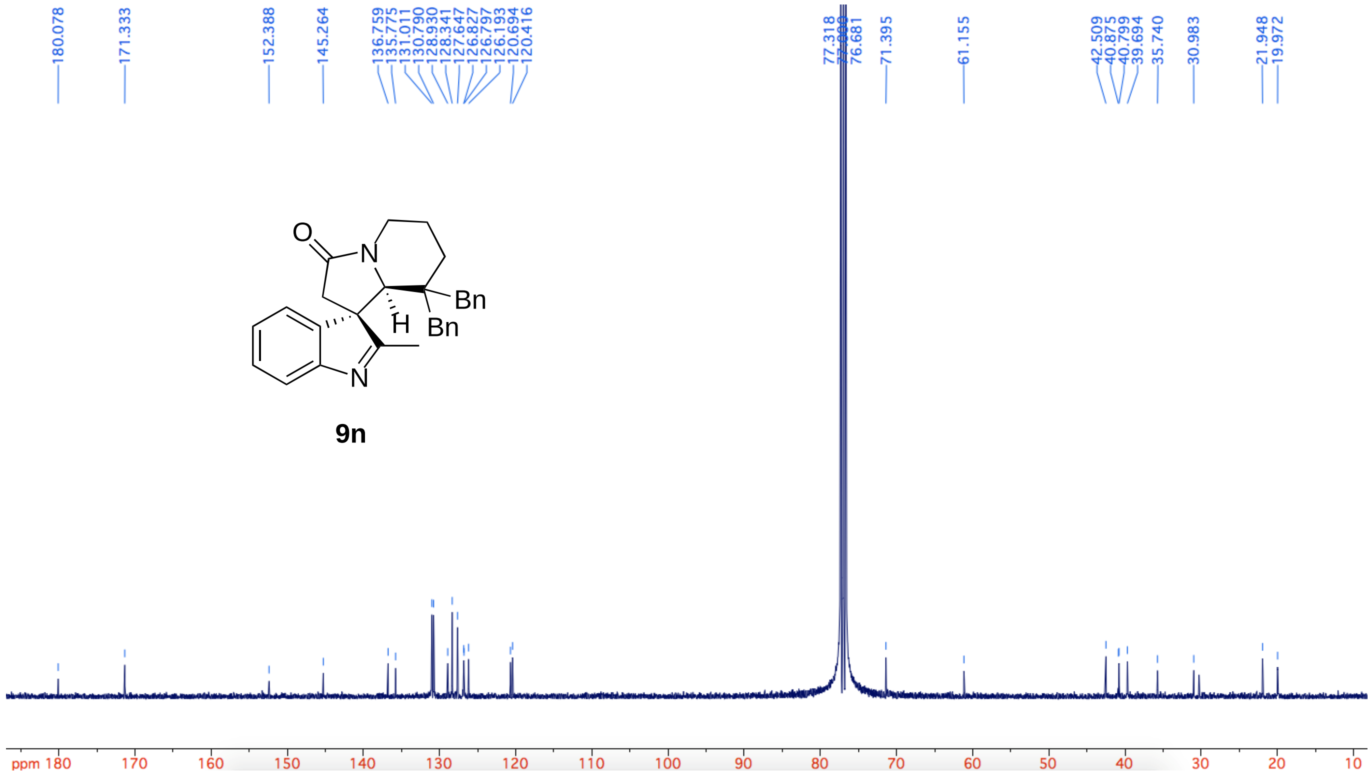
180.078
171.333
152.388
145.264
136.759
135.775
131.011
130.790
128.930
128.341
127.647
126.827
126.797
126.193
120.694
120.416
77.318
76.681
71.395
61.155
42.509
40.875
40.799
39.694
35.740
30.983
21.948
19.972
9n
Bn
Bn
H
ppm 180
170
160
150
140
130
120
110
100
90
80
70
60
50
40
30
20
10

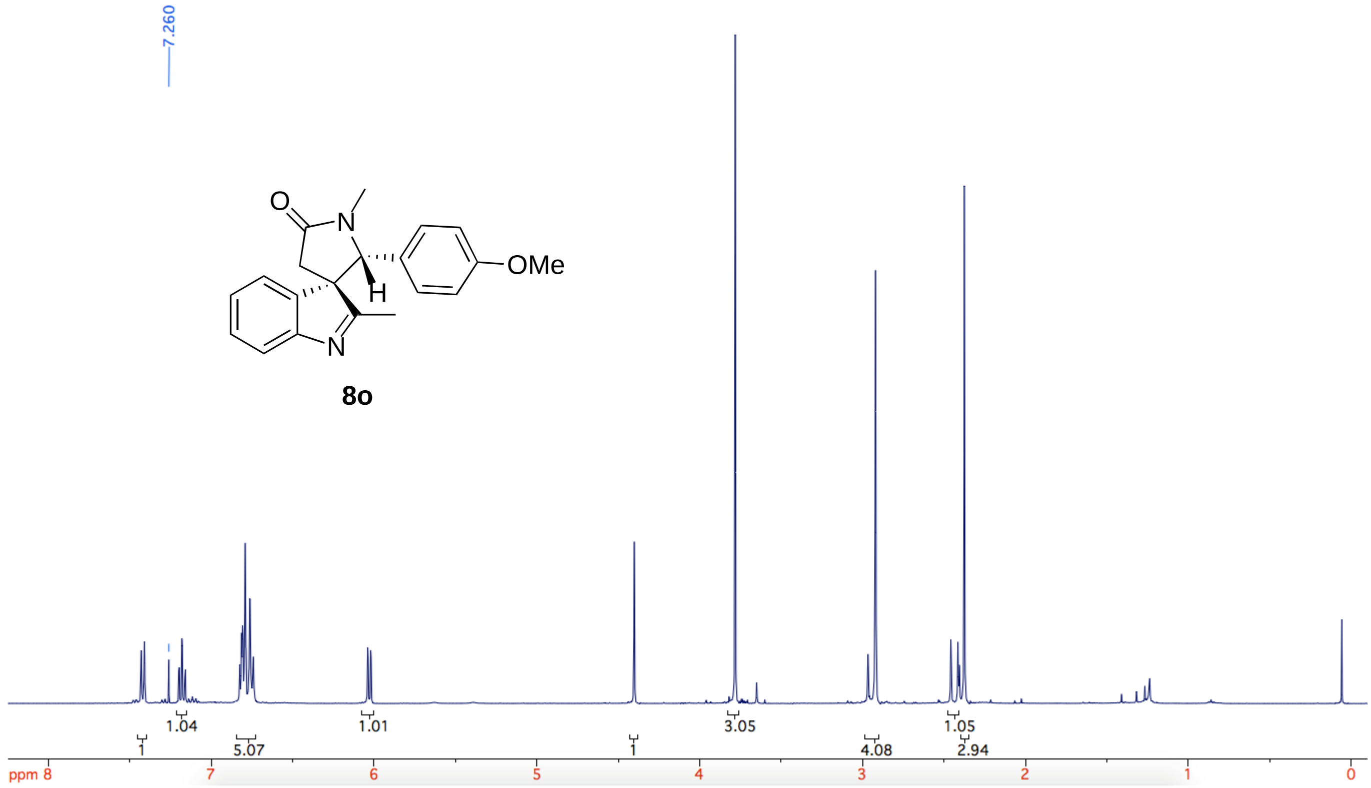
7.260
O
N
OMe
H
N
8o
1.04
1.01
3.05
1.05
1
5.07
1
4.08
2.94
ppm 8
7
6
5
4
3
2
1
0

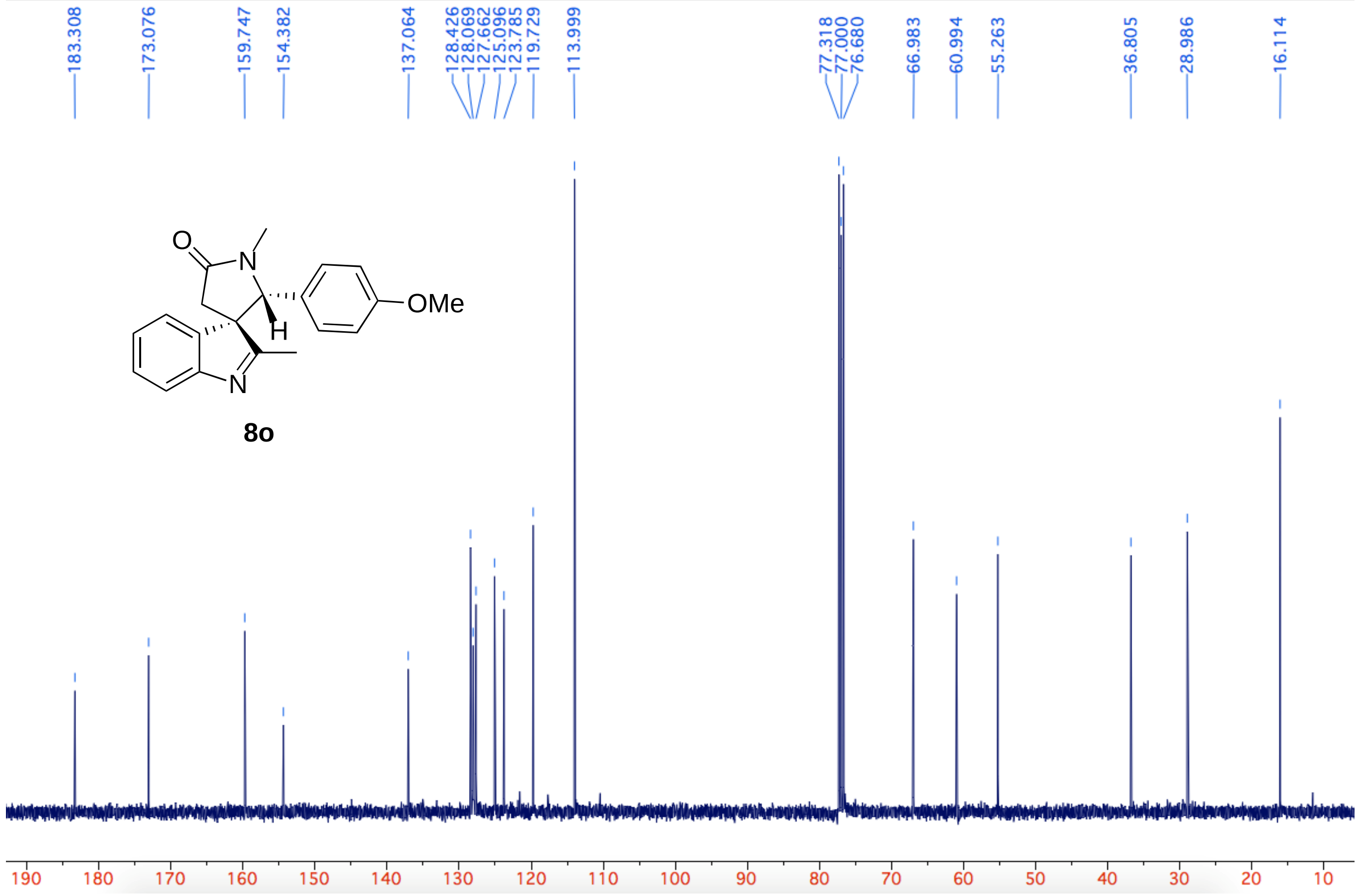
183.308
173.076
159.747
154.382
137.064
128.426
128.069
127.662
125.096
123.785
119.729
113.999
77.318
77.000
76.680
66.983
60.994
55.263
36.805
28.986
16.114
O
N
OMe
H
N
8o
190
180
170
160
150
140
130
120
110
100
90
80
70
60
50
40
30
20
10

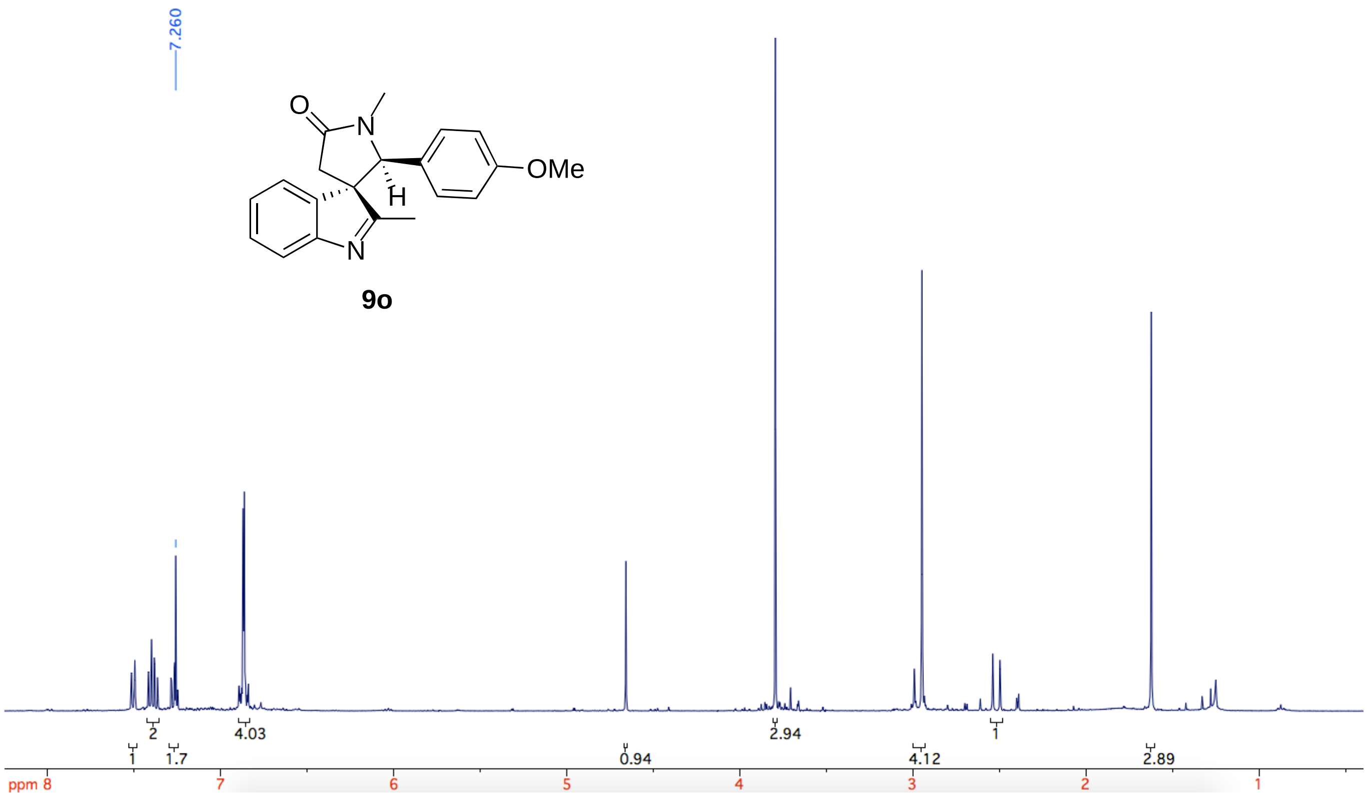

7.260
O
N
OMe
H
N
9o
1
2
1.7
4.03
0.94
2.94
4.12
1
2.89
ppm 8
7
6
5
4
3
2
1

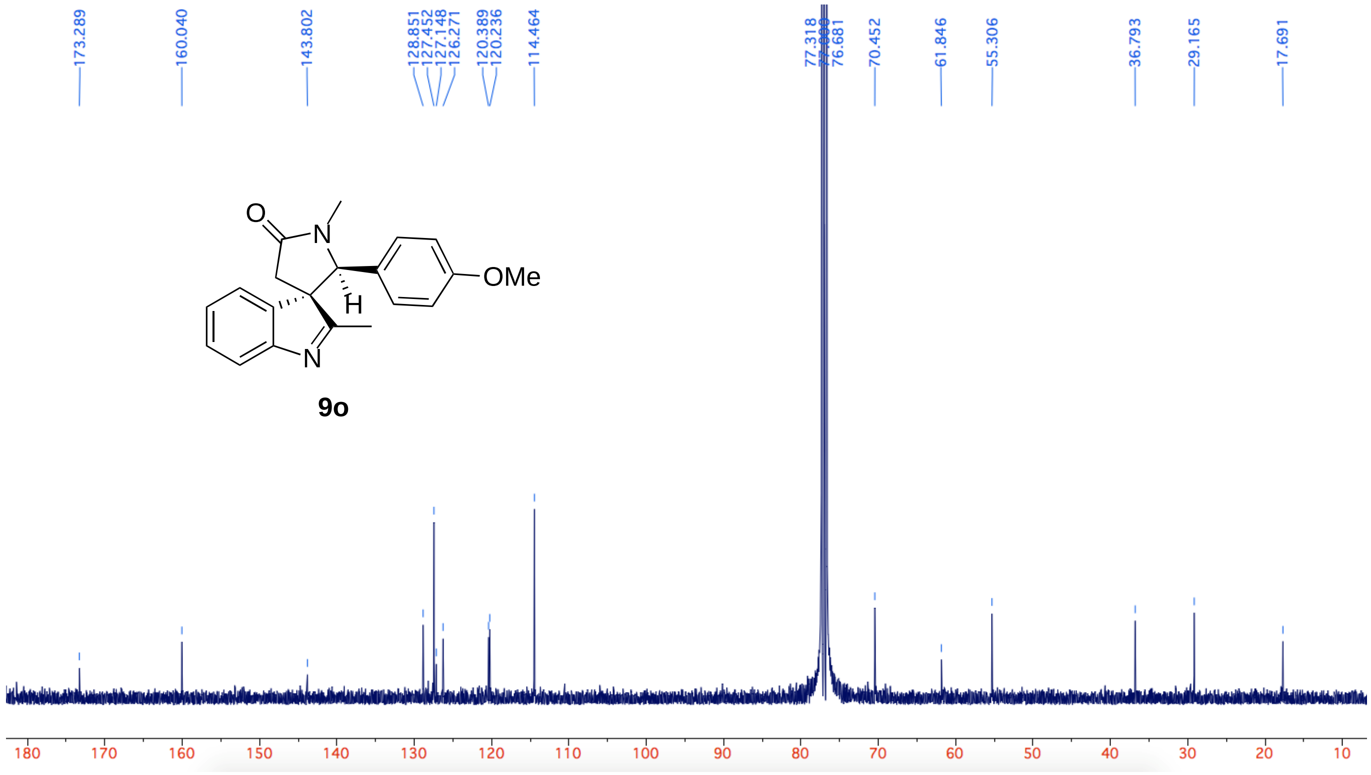

173.289
160.040
143.802
128.851
127.452
127.148
126.271
120.389
120.236
114.464
77.318
76.681
70.452
61.846
55.306
36.793
29.165
17.691
O
N
OMe
H
N
9o
180
170
160
150
140
130
120
110
100
90
80
70
60
50
40
30
20
10

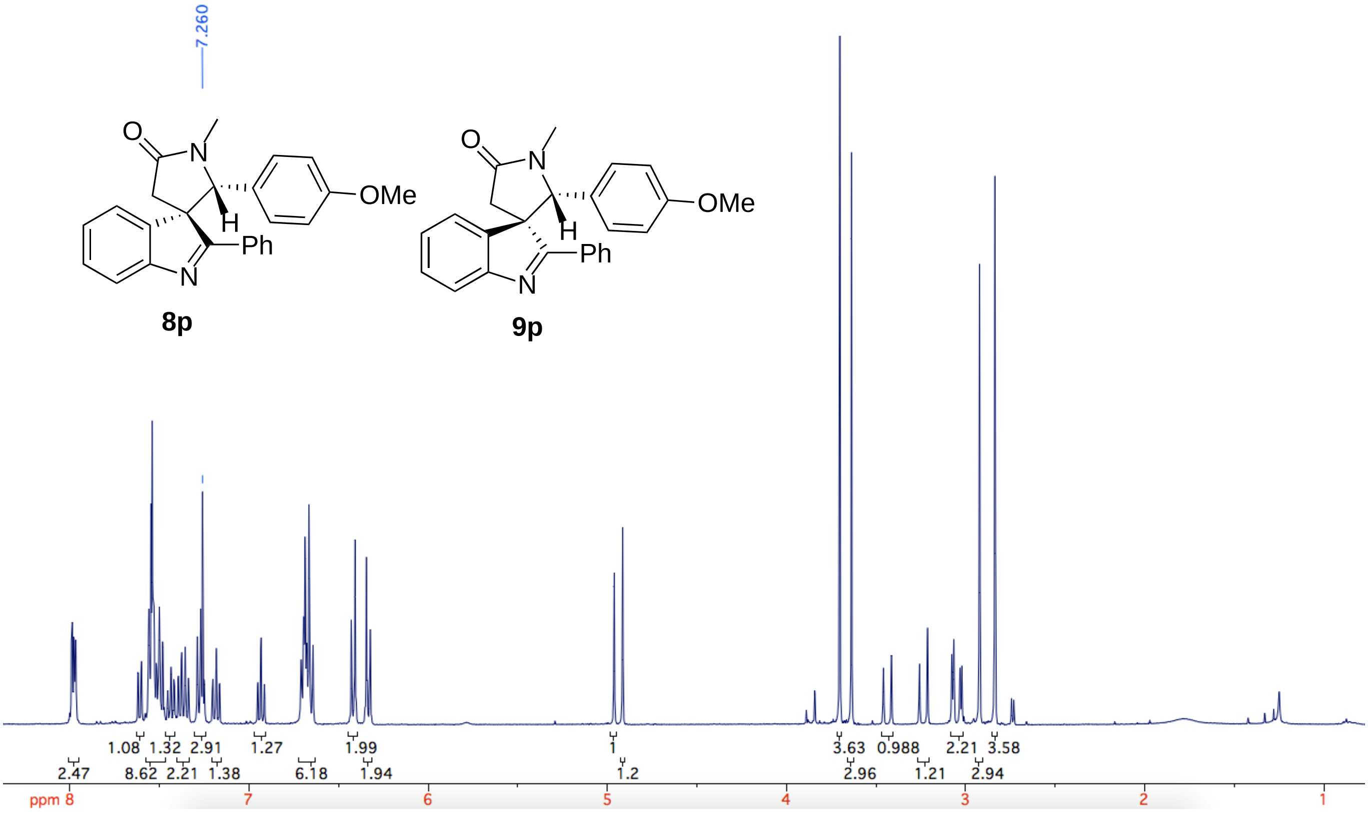
7.260
8p
9p
OMe
Ph
1.08 1.32 2.91 1.27 1.99 1 3.63 0.988 2.21 3.58
2.47 8.62 2.21 1.38 6.18 1.94 1.2 2.96 1.21 2.94
ppm 8 7 6 5 4 3 2 1

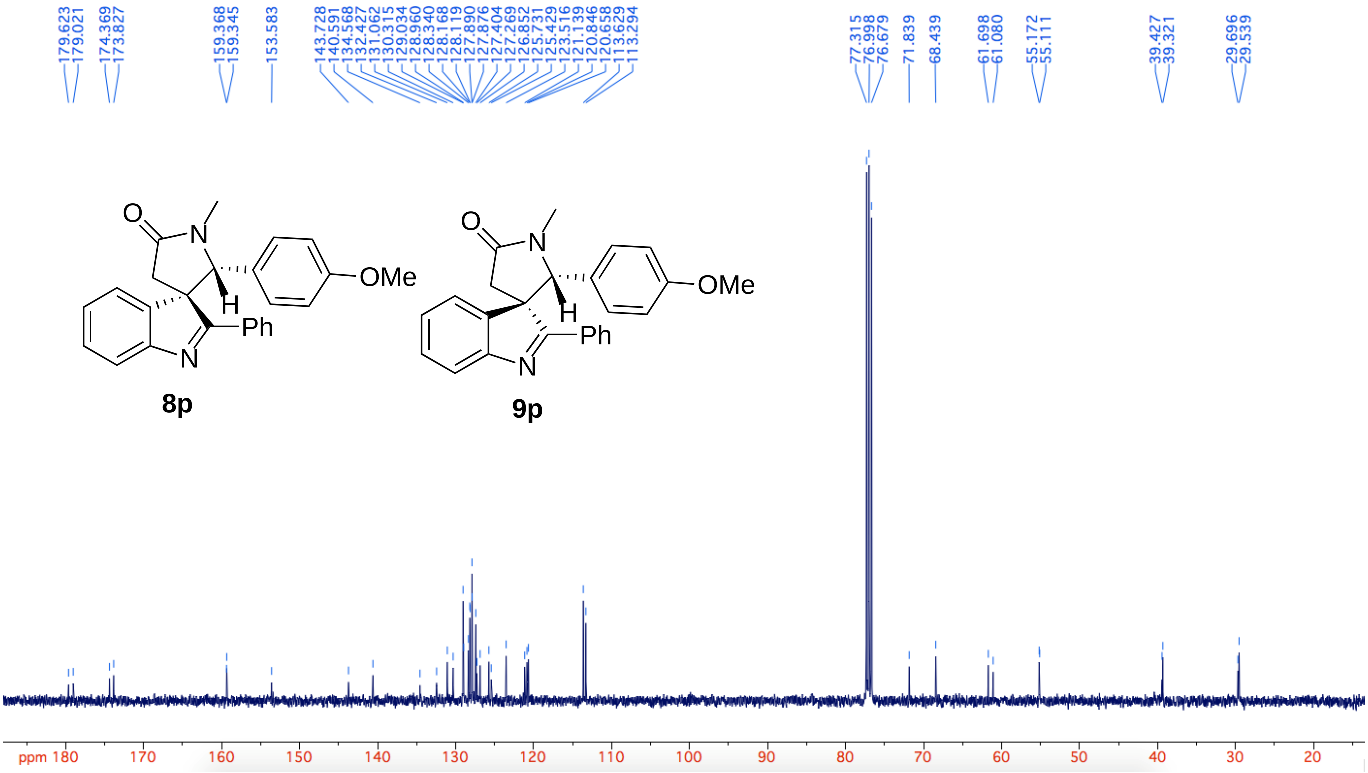
179.623 179.021 174.369 173.827 159.368 159.345 153.583 143.728 140.591 134.568 132.427 131.062 130.315 129.034 128.960 128.340 128.168 128.119 127.890 127.876 127.404 127.269 126.852 125.731 125.429 123.516 121.139 120.846 120.658 113.629 113.294 77.315 76.998 76.679 71.839 68.439 61.698 61.080 55.172 55.111 39.427 39.321 29.696 29.539
8p
9p
OMe
Ph
ppm 180 170 160 150 140 130 120 110 100 90 80 70 60 50 40 30 20

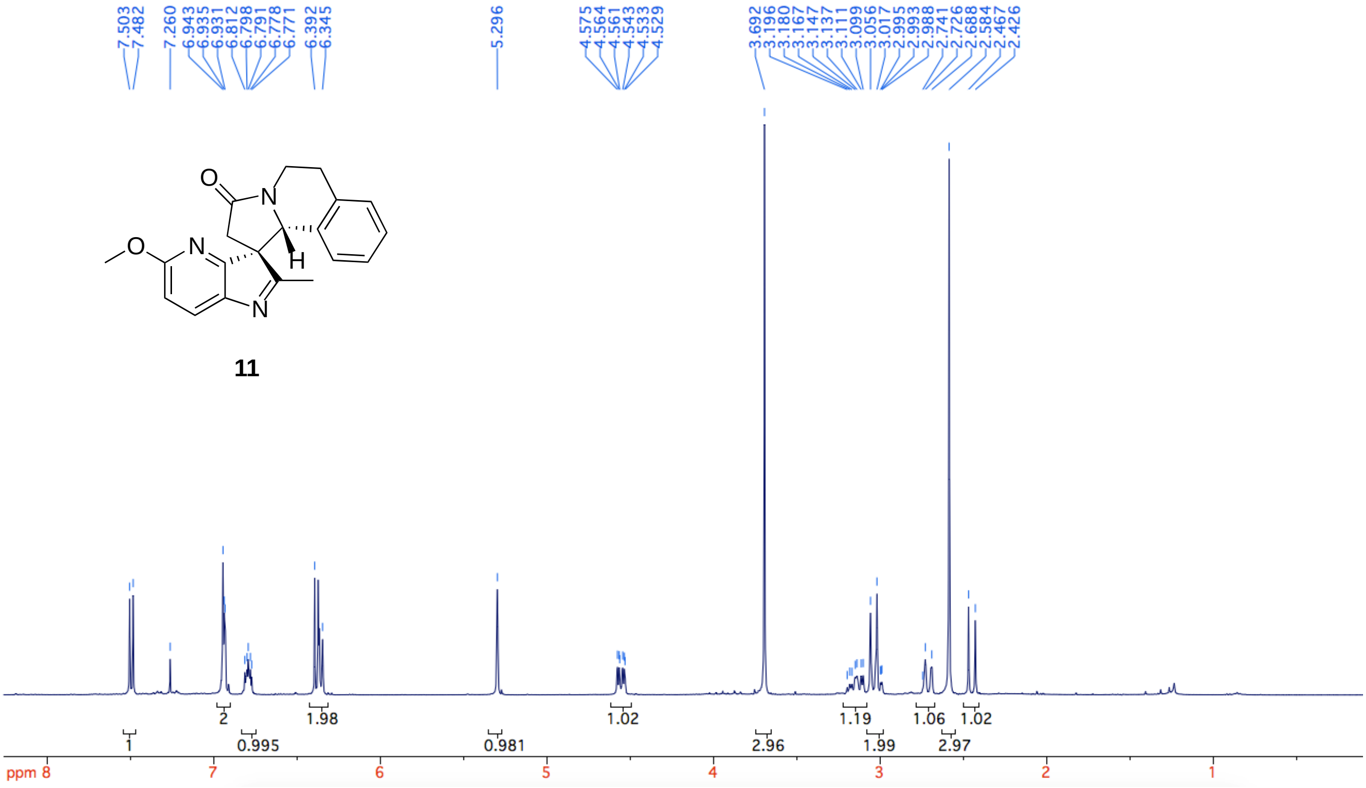
7.503
7.482
7.260
6.943
6.935
6.931
6.812
6.798
6.791
6.778
6.771
6.392
6.345
5.296
4.575
4.564
4.561
4.543
4.533
4.529
3.692
3.196
3.180
3.167
3.147
3.137
3.111
3.099
3.056
3.017
2.995
2.993
2.988
2.741
2.726
2.688
2.584
2.467
2.426
O
N
N
H
O
N
11
1
2
0.995
1.98
0.981
1.02
2.96
1.19
1.99
1.06
2.97
1.02
ppm 8
7
6
5
4
3
2
1

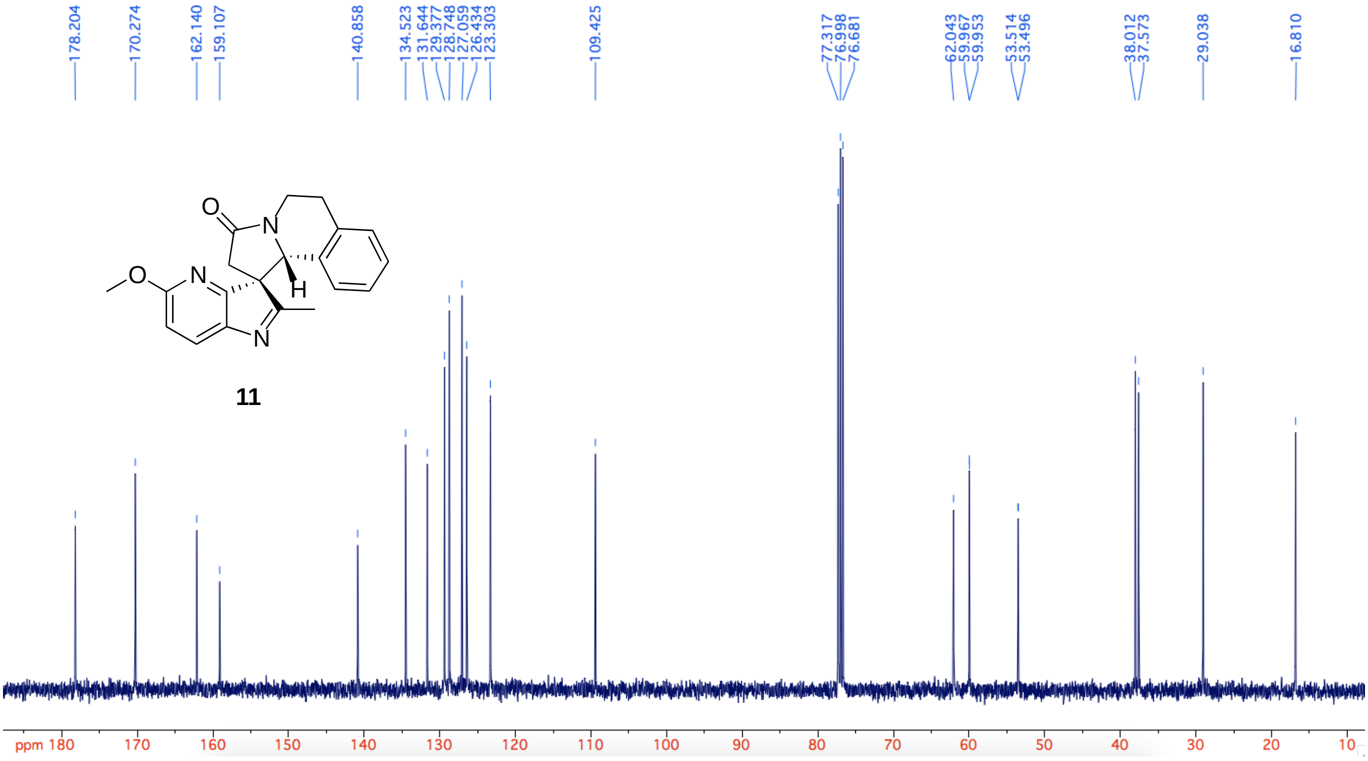
178.204
170.274
162.140
159.107
140.858
134.523
131.644
129.377
128.748
127.059
126.434
123.303
109.425
77.317
76.998
76.681
62.043
59.967
59.953
53.514
53.496
38.012
37.573
29.038
16.810
O
N
N
H
O
N
11
ppm 180
170
160
150
140
130
120
110
100
90
80
70
60
50
40
30
20
10

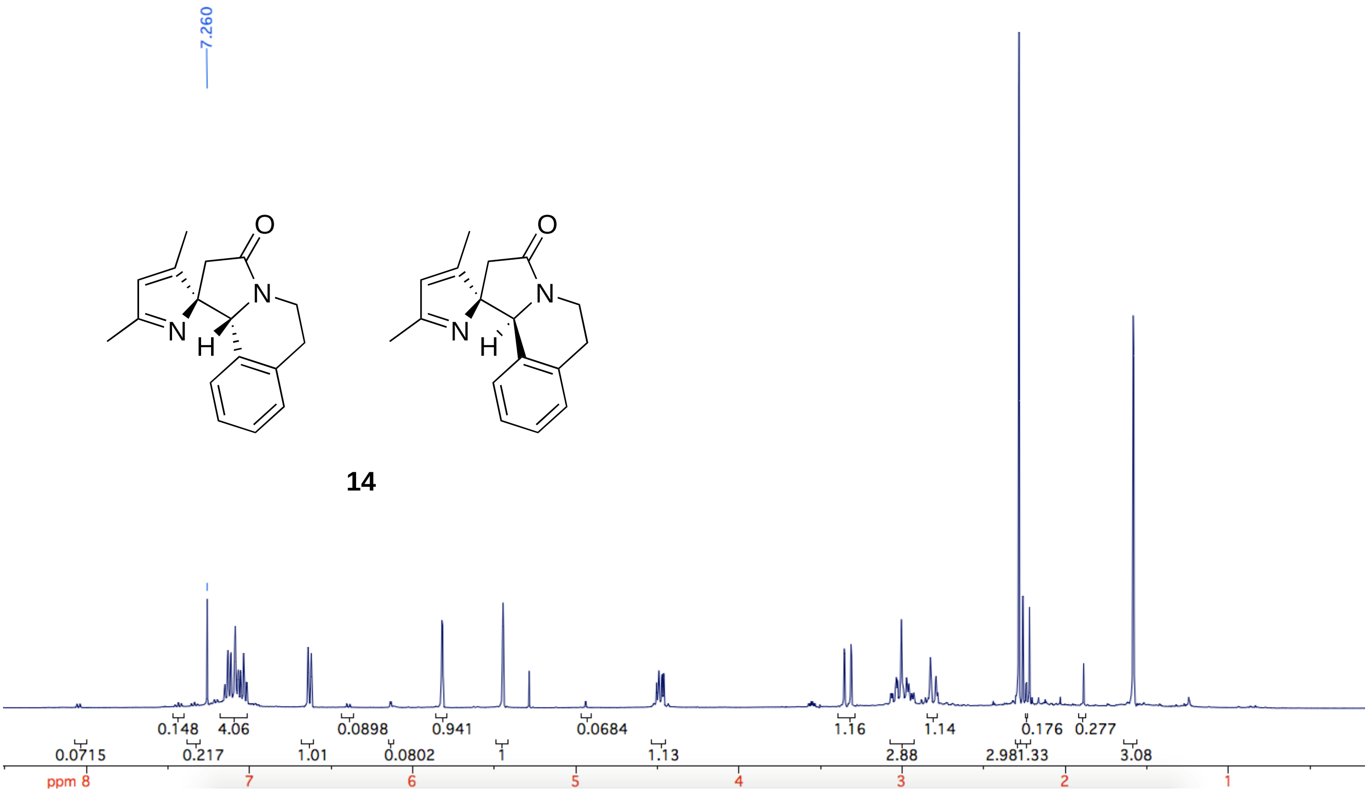
7.260
O
N
N
H
O
N
N
H
14
0.148
4.06
0.0898
0.941
0.0684
1.16
1.14
0.176
0.277
0.0715
0.217
1.01
0.0802
1
1.13
2.88
2.981.33
3.08
ppm 8
7
6
5
4
3
2
1

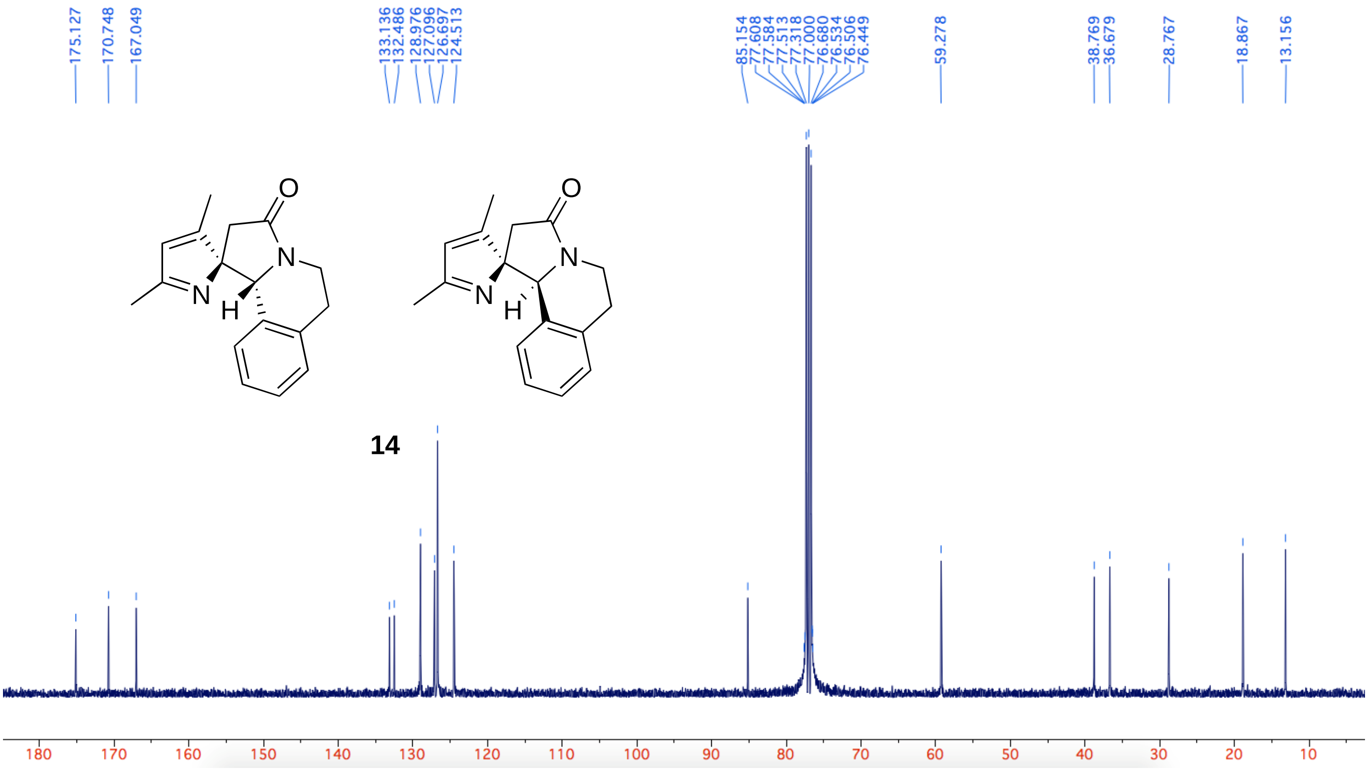
175.127
170.748
167.049
133.136
132.486
128.976
127.096
126.697
124.513
85.154
77.608
77.584
77.513
77.318
77.000
76.680
76.534
76.506
76.449
59.278
38.769
36.679
28.767
18.867
13.156
O
N
N
H
O
N
N
H
14
180
170
160
150
140
130
120
110
100
90
80
70
60
50
40
30
20
10

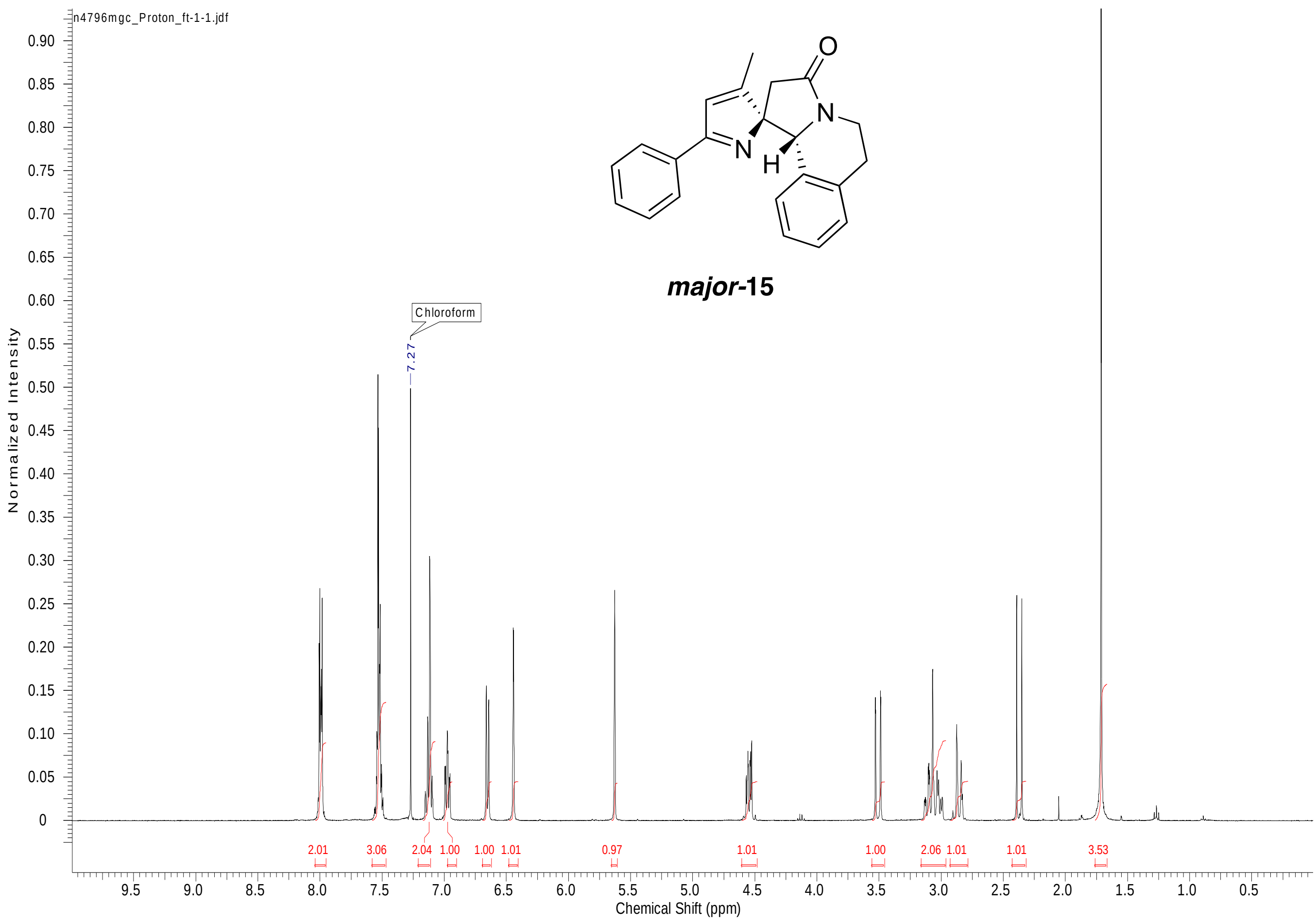
n4796mgc_Proton_ft-1-1.jdf
major-15
Chloroform
7.27
2.01
3.06
2.04
1.00
1.00
1.01
0.97
1.01
1.00
2.06
1.01
1.01
3.53
Normalized Intensity
Chemical Shift (ppm)

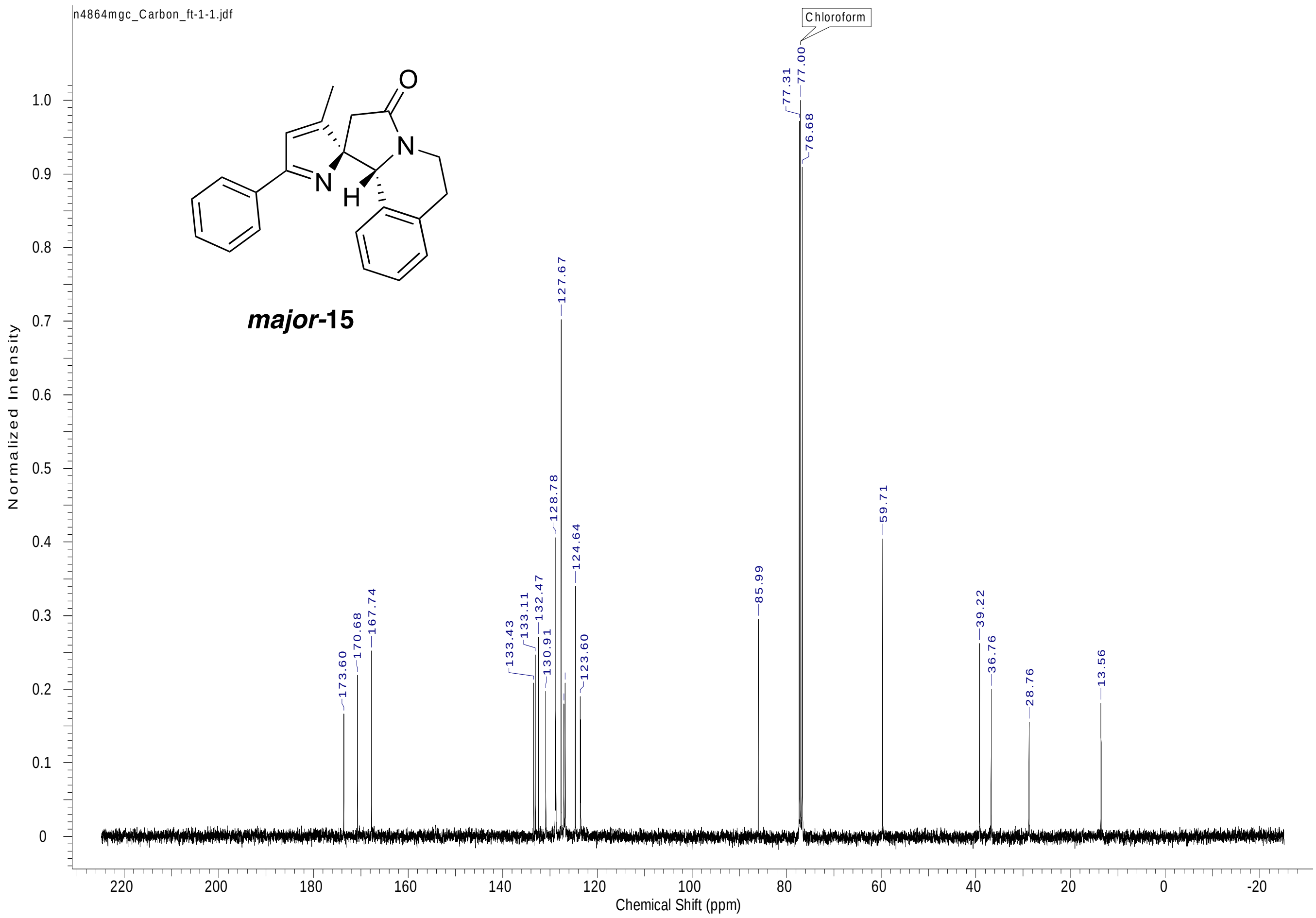
n4864mgc_Carbon_ft-1-1.jdf
major-15
Chloroform
77.31
77.00
76.68
173.60
170.68
167.74
133.43
133.11
132.47
130.91
128.78
127.67
124.64
123.60
85.99
59.71
39.22
36.76
28.76
13.56
Normalized Intensity
Chemical Shift (ppm)

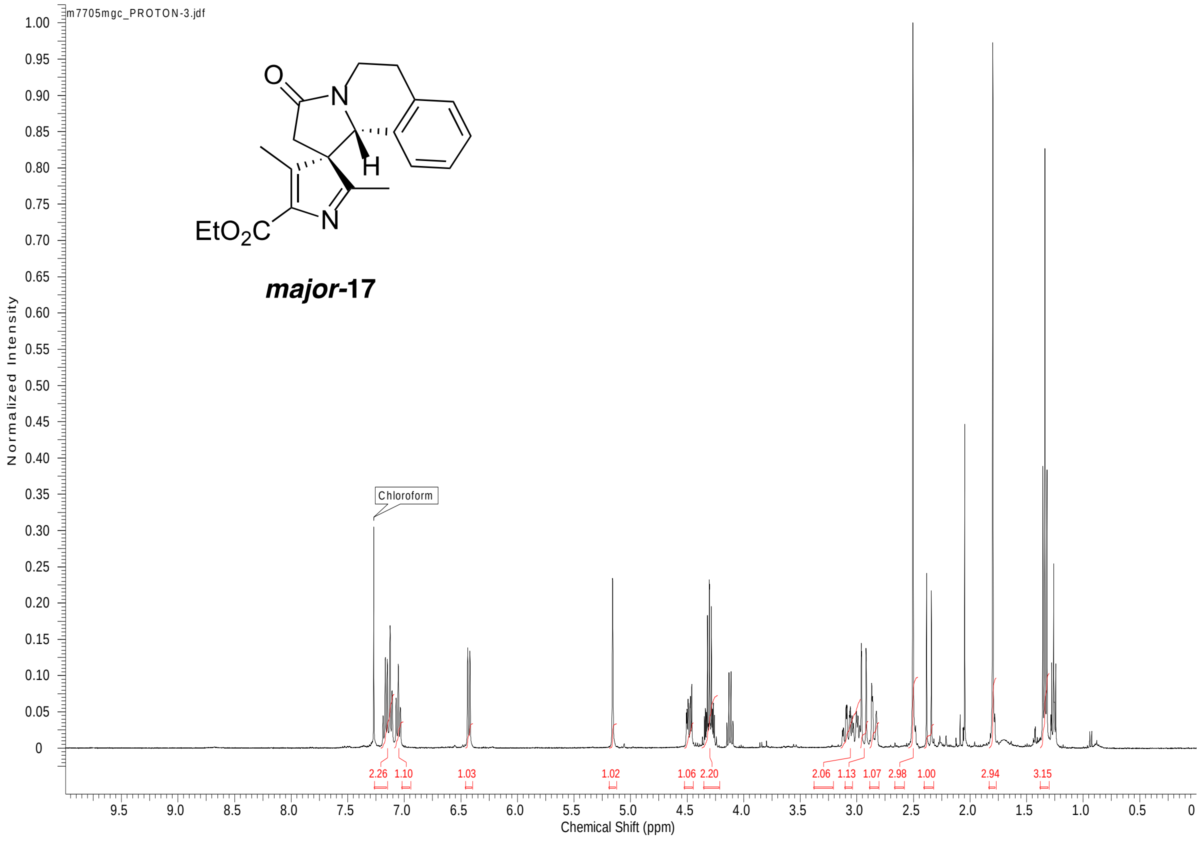
m7705mgc_PROTON-3.jdf
major-17
EtO2C
Chloroform
2.26
1.10
1.03
1.02
1.06
2.20
2.06
1.13
1.07
2.98
1.00
2.94
3.15
Normalized Intensity
Chemical Shift (ppm)

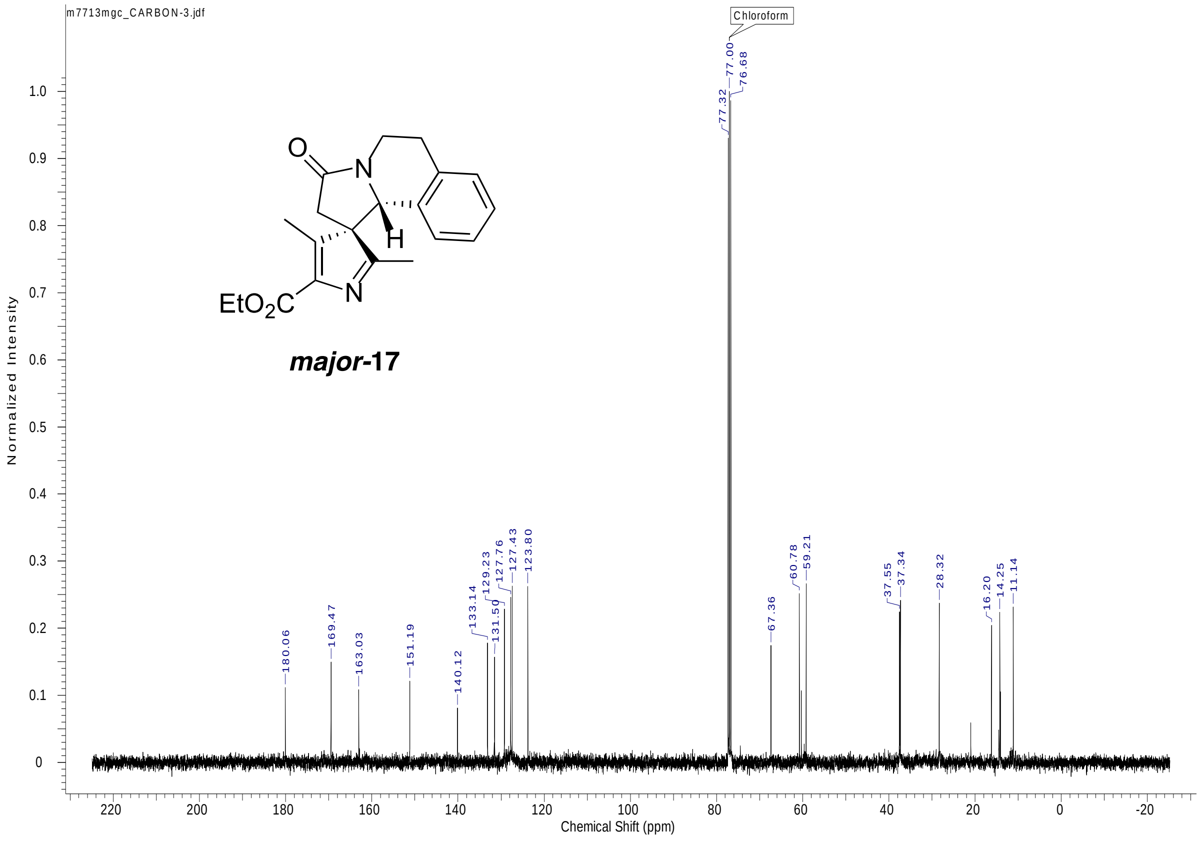
m7713mgc_CARBON-3.jdf
major-17
EtO2C
Chloroform
77.32
77.00
76.68
180.06
169.47
163.03
151.19
140.12
133.14
131.50
129.23
127.76
127.43
123.80
67.36
60.78
59.21
37.55
37.34
28.32
16.20
14.25
11.14
Normalized Intensity
Chemical Shift (ppm)

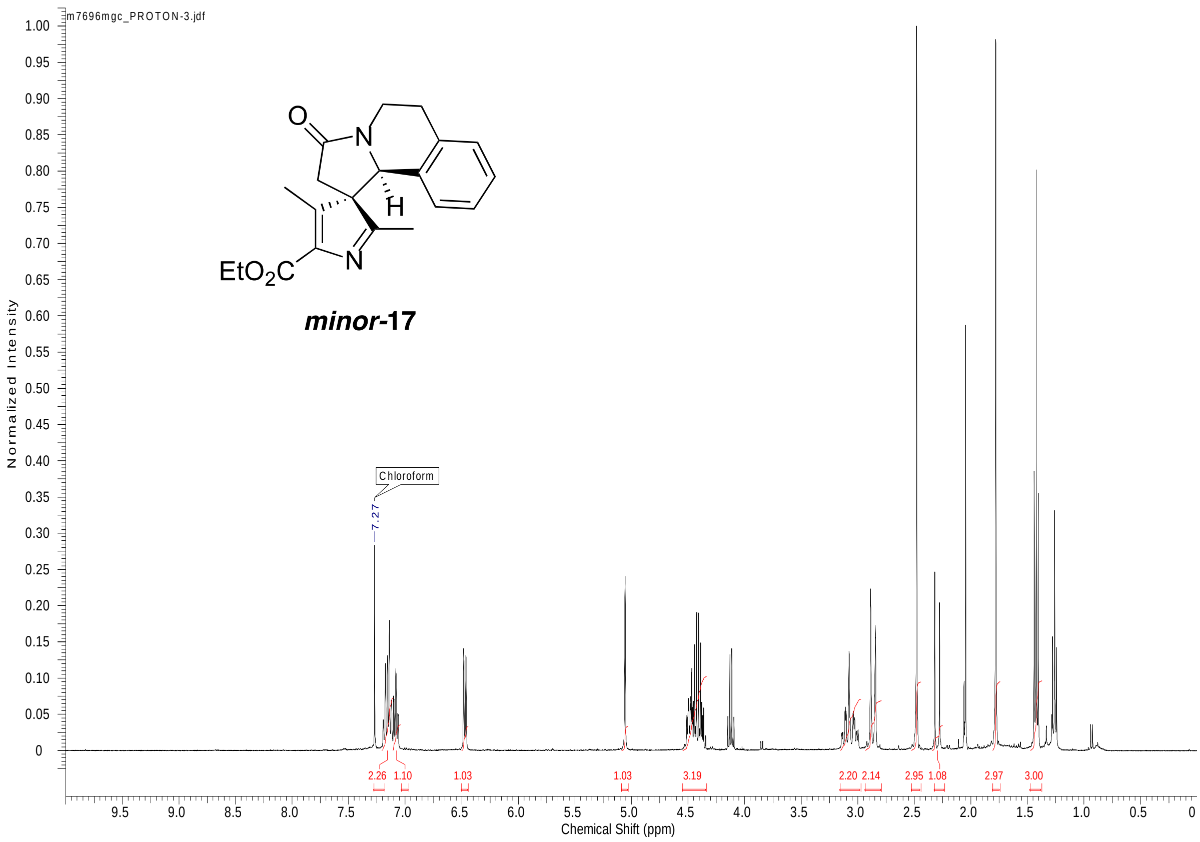
m7696mgc_PROTON-3.jdf
O
N
H
EtO2C
N
minor-17
Chloroform
7.27
2.26 1.10 1.03 1.03 3.19 2.20 2.14 2.95 1.08 2.97 3.00
Normalized Intensity
Chemical Shift (ppm)

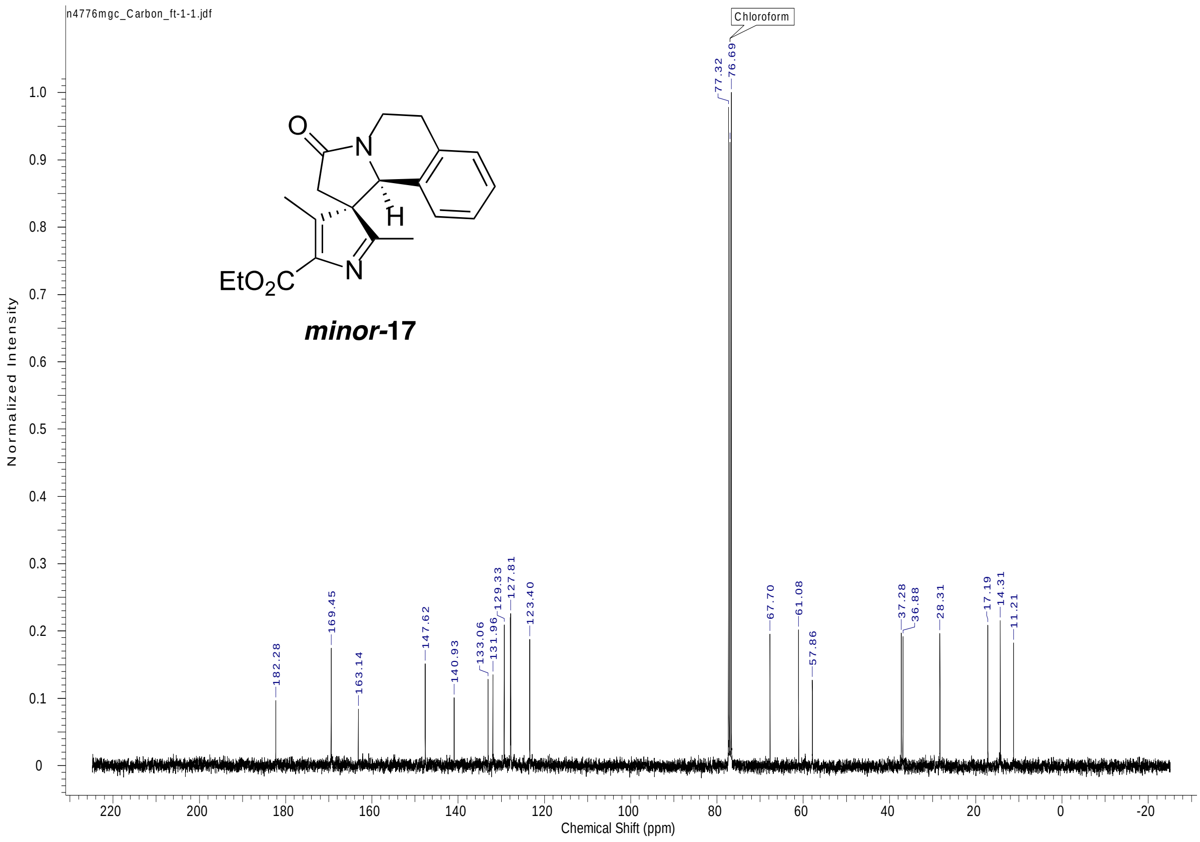
n4776mgc_Carbon_ft-1-1.jdf
Chloroform
77.32
76.69
O
N
H
EtO2C
N
minor-17
182.28
169.45
163.14
147.62
140.93
133.06
131.96
129.33
127.81
123.40
67.70
61.08
57.86
37.28
36.88
28.31
17.19
14.31
11.21
Normalized Intensity
Chemical Shift (ppm)

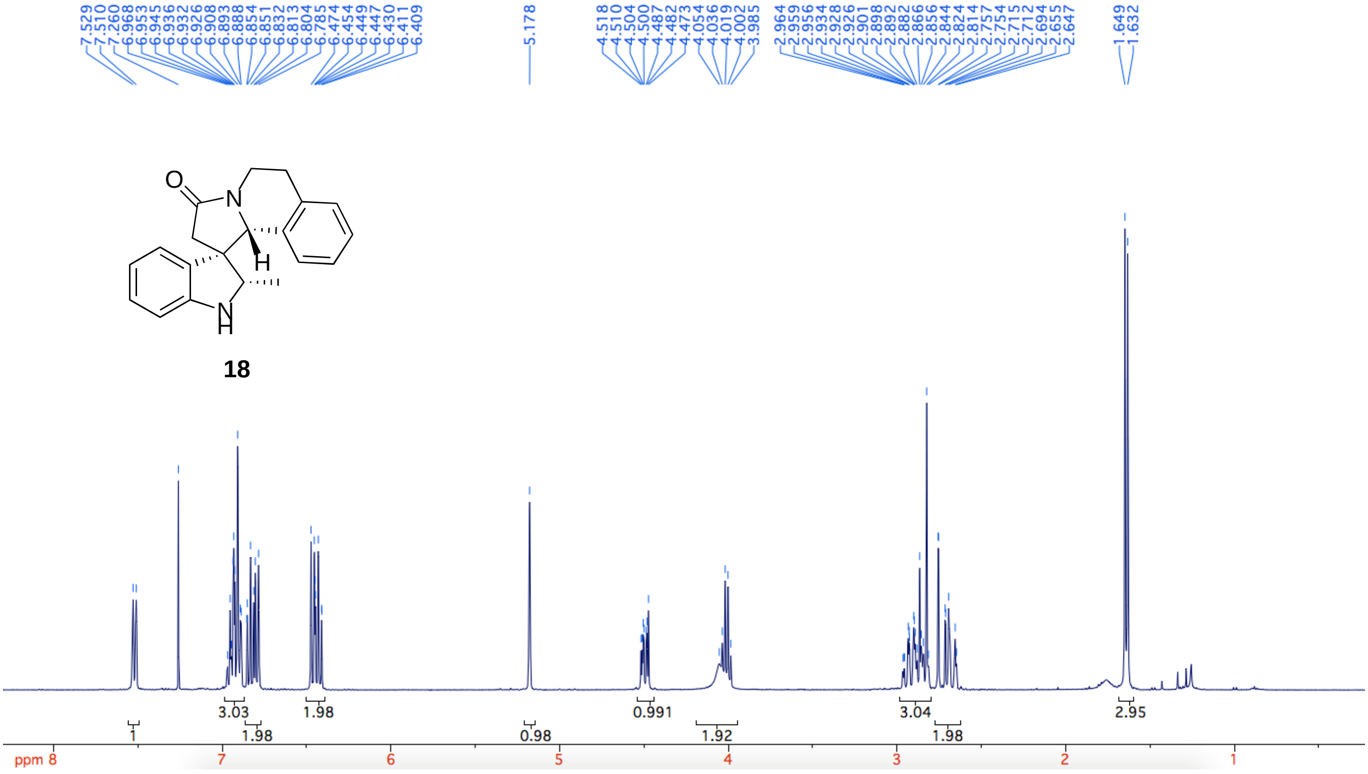
7.529
7.510
7.260
6.968
6.953
6.945
6.936
6.932
6.926
6.908
6.893
6.888
6.854
6.851
6.832
6.813
6.804
6.785
6.474
6.454
6.449
6.447
6.430
6.411
6.409
5.178
4.518
4.510
4.504
4.500
4.487
4.482
4.473
4.054
4.036
4.019
4.002
3.985
2.964
2.959
2.956
2.934
2.928
2.926
2.901
2.898
2.892
2.882
2.866
2.856
2.844
2.824
2.814
2.757
2.754
2.715
2.712
2.694
2.655
2.647
1.649
1.632
18
3.03
1.98
0.991
3.04
2.95
1
1.98
0.98
1.92
1.98
ppm 8
7
6
5
4
3
2
1

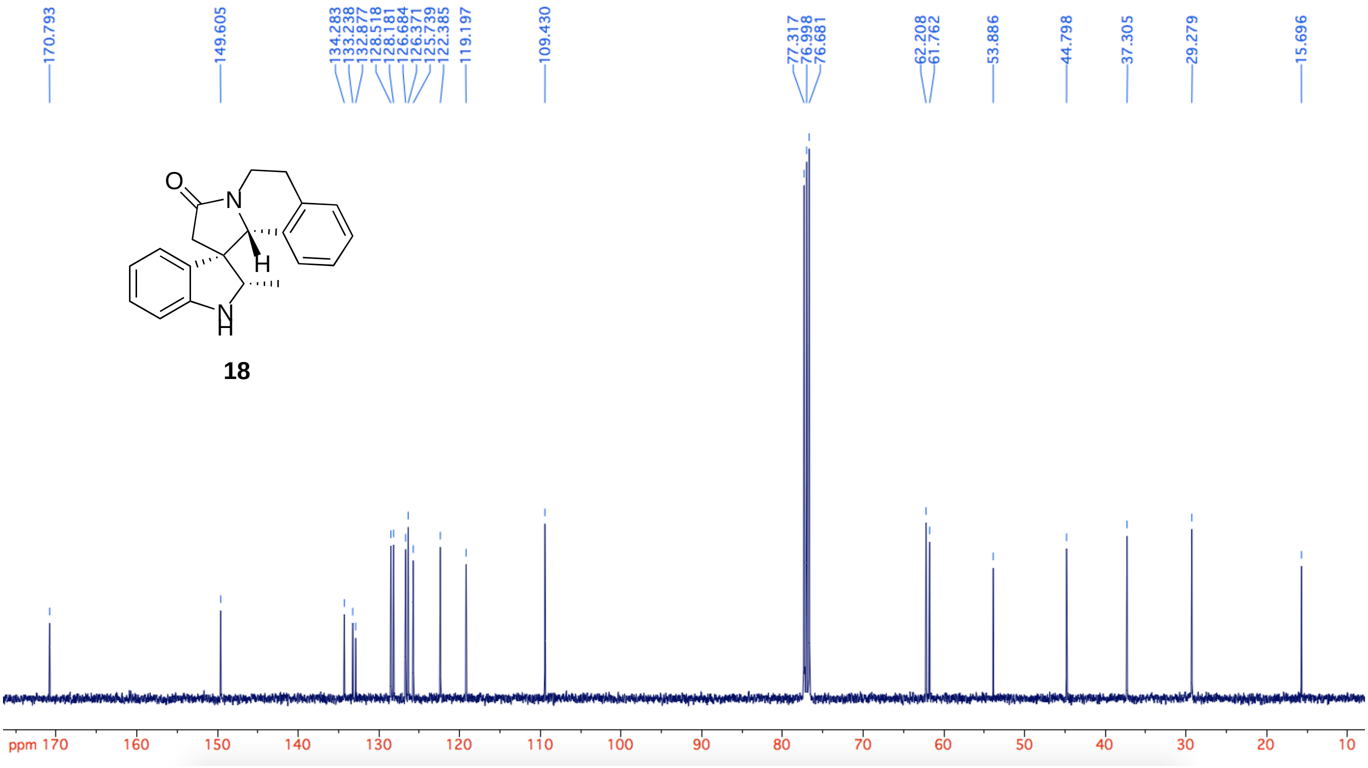
170.793
149.605
134.283
133.238
132.877
128.518
128.181
126.684
126.371
125.739
122.385
119.197
109.430
77.317
76.998
76.681
62.208
61.762
53.886
44.798
37.305
29.279
15.696
18
ppm 170
160
150
140
130
120
110
100
90
80
70
60
50
40
30
20
10

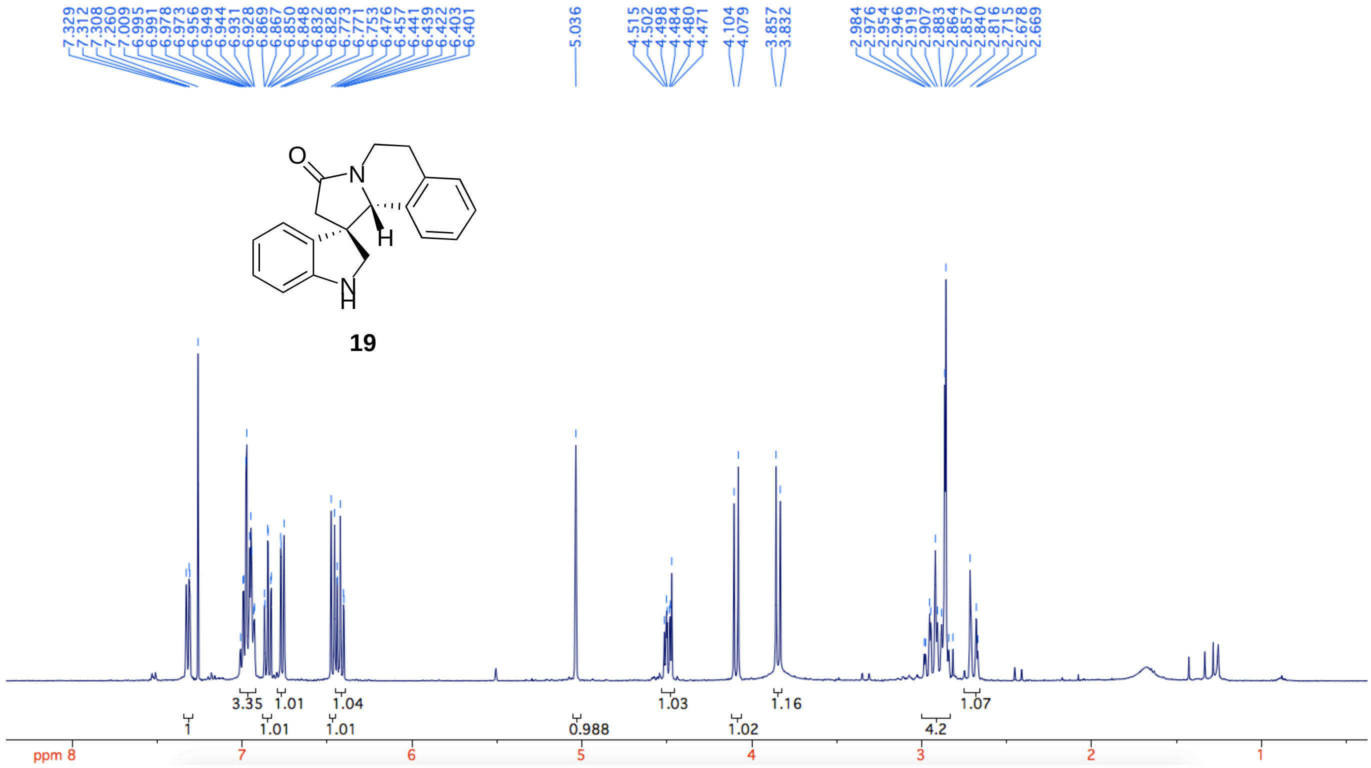
7.329
7.312
7.308
7.260
7.009
6.995
6.991
6.978
6.973
6.956
6.949
6.944
6.931
6.928
6.869
6.867
6.850
6.848
6.832
6.828
6.773
6.771
6.753
6.476
6.457
6.441
6.439
6.422
6.403
6.401
5.036
4.515
4.502
4.498
4.484
4.480
4.471
4.104
4.079
3.857
3.832
2.984
2.976
2.954
2.946
2.919
2.907
2.883
2.864
2.857
2.840
2.816
2.715
2.678
2.669
O
N
H
N
H
19
3.35
1.01
1.04
1.03
1.16
1.07
1
1.01
1.01
0.988
1.02
4.2
ppm 8
7
6
5
4
3
2
1

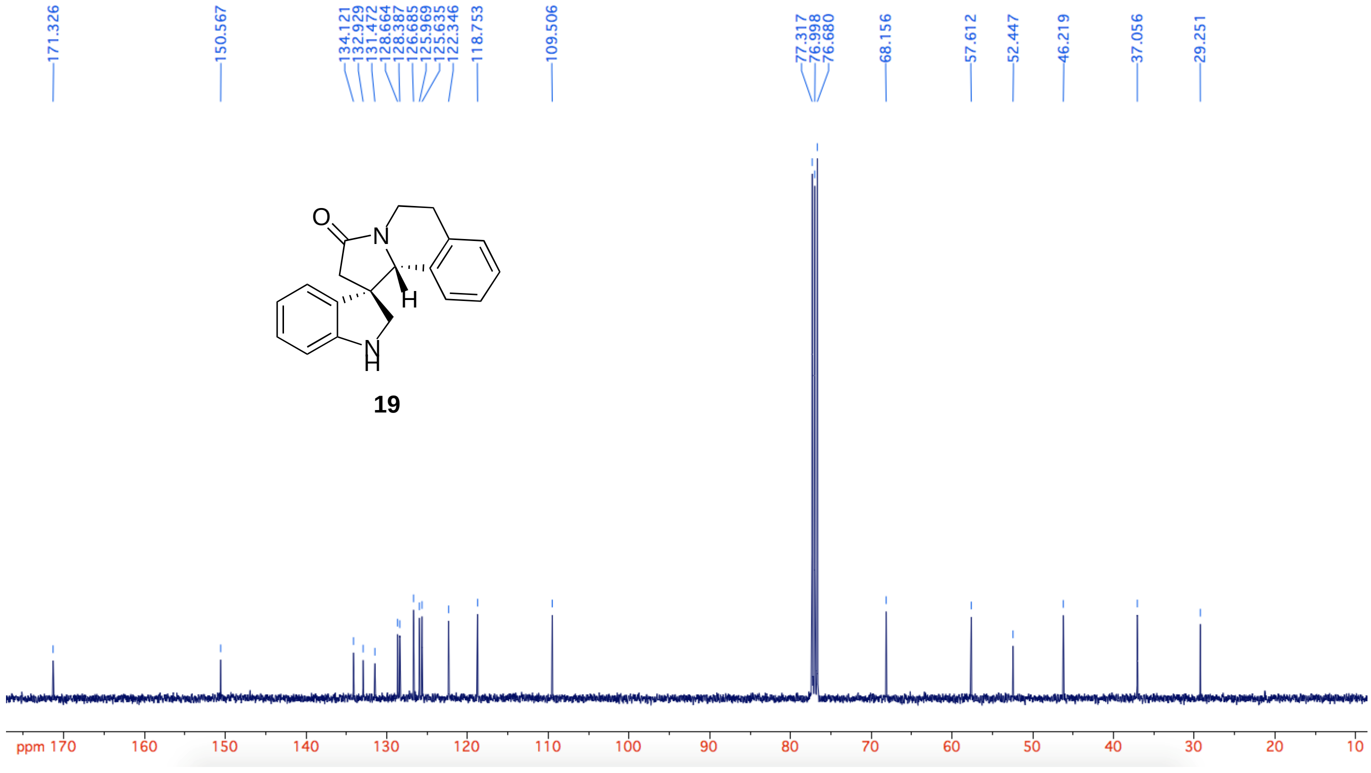
171.326
150.567
134.121
132.929
131.472
128.664
128.387
126.585
125.969
125.635
122.346
118.753
109.506
77.317
76.998
76.680
68.156
57.612
52.447
46.219
37.056
29.251
O
N
H
N
H
19
ppm 170
160
150
140
130
120
110
100
90
80
70
60
50
40
30
20
10

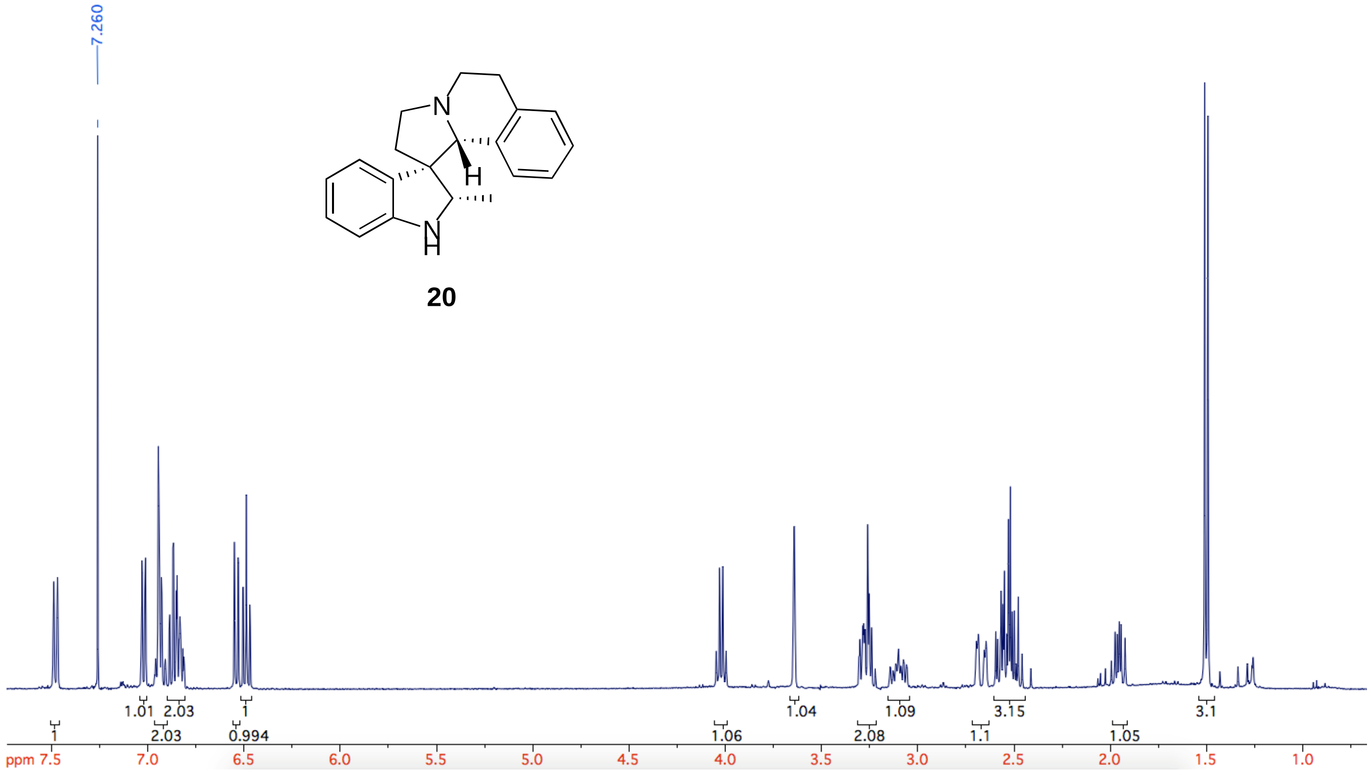
7.260
20
1
1.01
2.03
2.03
1
0.994
1.06
1.04
2.08
1.09
1.1
3.15
1.05
3.1
ppm 7.5 7.0 6.5 6.0 5.5 5.0 4.5 4.0 3.5 3.0 2.5 2.0 1.5 1.0

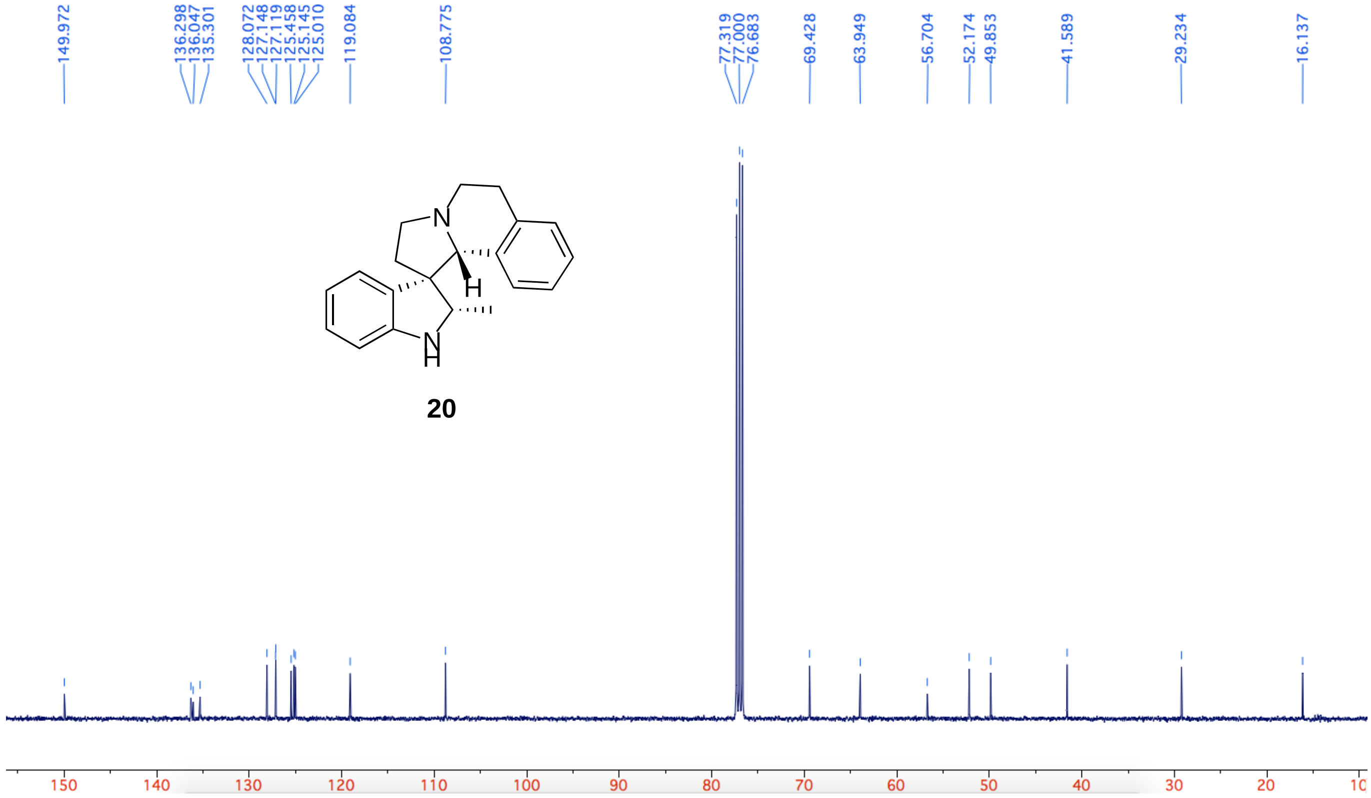
149.972
136.298
136.047
135.301
128.072
127.148
127.119
125.458
125.145
125.010
119.084
108.775
77.319
77.000
76.683
69.428
63.949
56.704
52.174
49.853
41.589
29.234
16.137
20
150 140 130 120 110 100 90 80 70 60 50 40 30 20 10

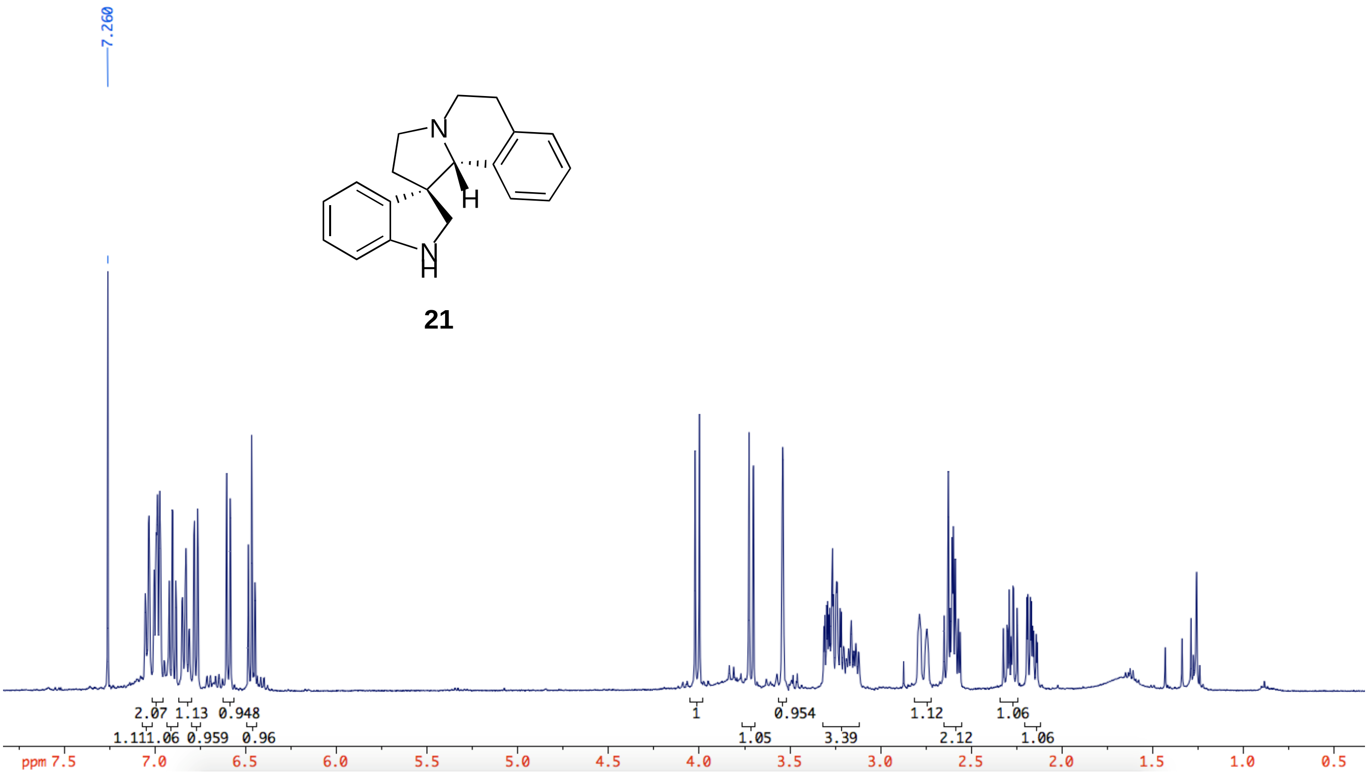
7.260
2.07 1.13 0.948
1.11 1.06 0.959 0.96
1
0.954
1.05
3.39
1.12
2.12
1.06
1.06
ppm 7.5 7.0 6.5 6.0 5.5 5.0 4.5 4.0 3.5 3.0 2.5 2.0 1.5 1.0 0.5

21

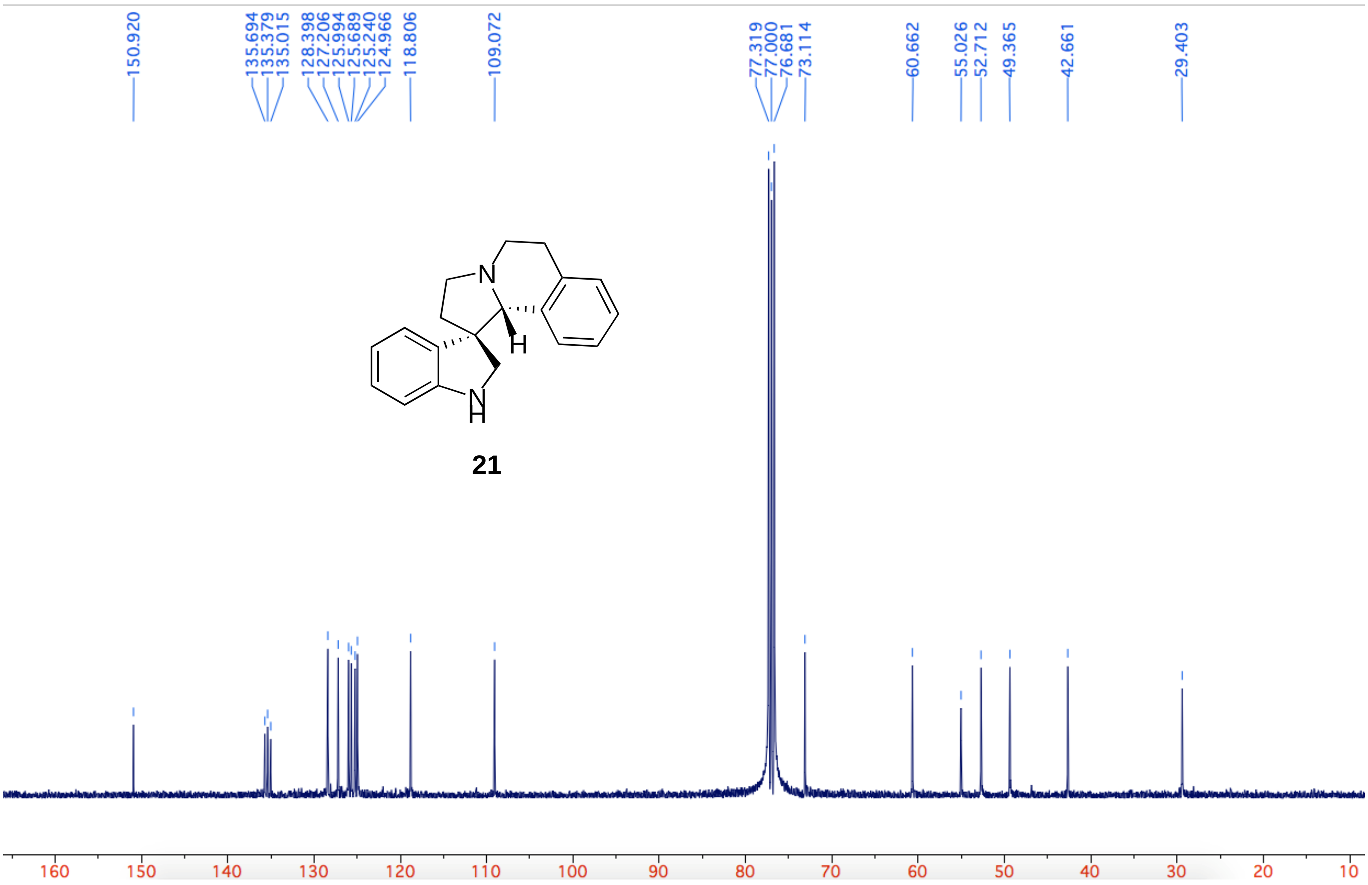
150.920
135.694
135.379
135.015
128.398
127.206
125.994
125.689
125.240
124.966
118.806
109.072
77.319
77.000
76.681
73.114
60.662
55.026
52.712
49.365
42.661
29.403
160 150 140 130 120 110 100 90 80 70 60 50 40 30 20 10

21

22

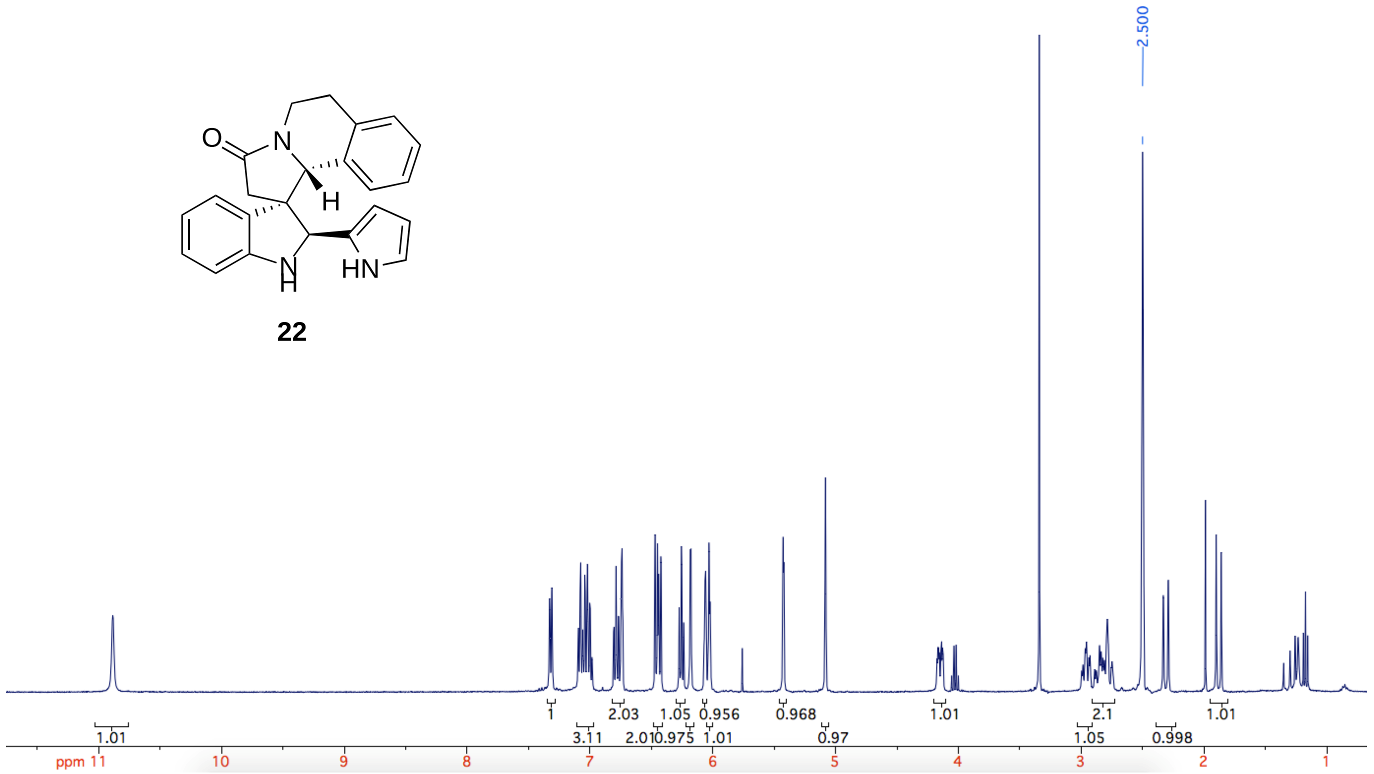
2.500
1.01
1
3.11
2.03
2.01
0.975
1.05
0.956
1.01
0.968
0.97
1.01
1.05
2.1
0.998
1.01
ppm

22

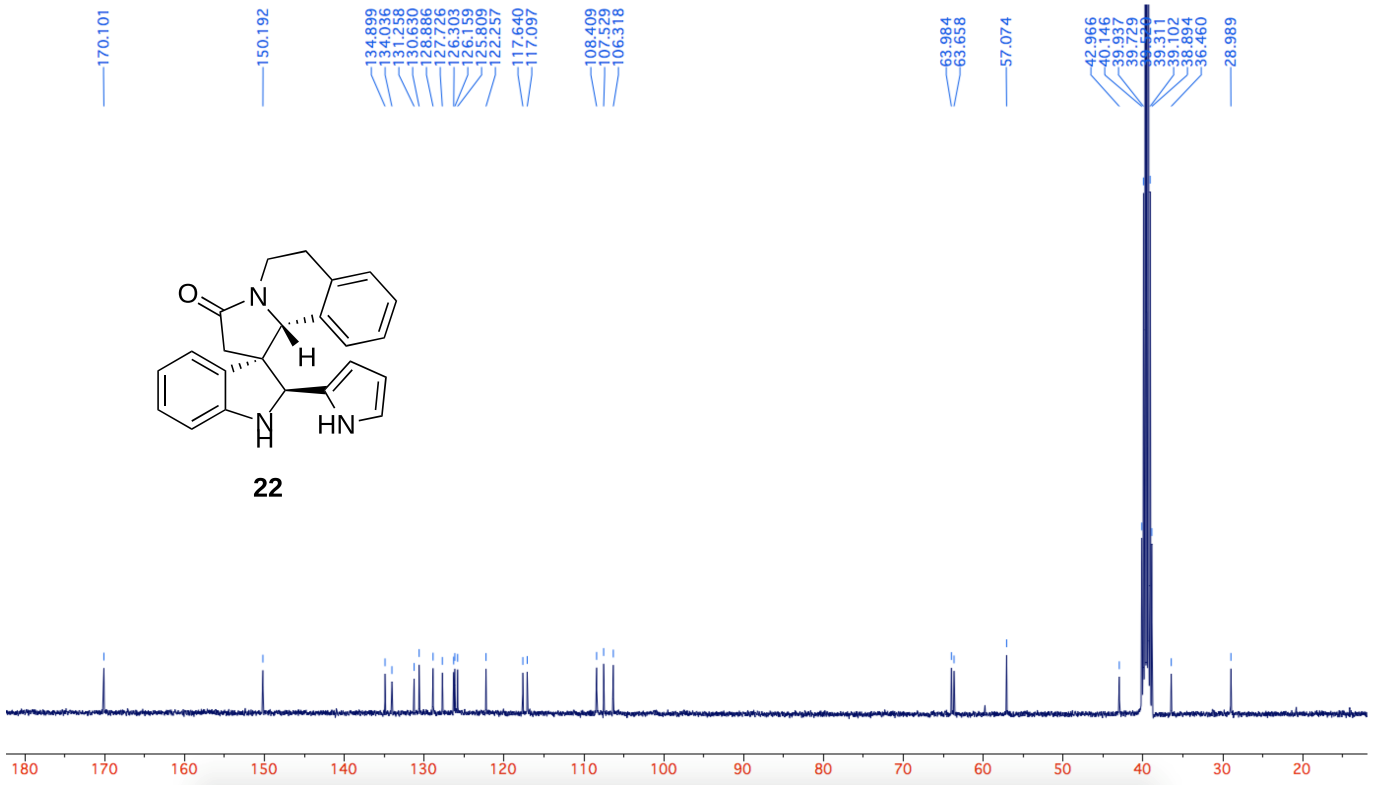
170.101
150.192
134.899
134.036
131.258
130.630
128.886
127.726
126.303
126.159
125.809
122.257
117.640
117.097
108.409
107.529
106.318
63.984
63.658
57.074
42.966
40.146
39.937
39.729
39.311
39.102
38.894
36.460
28.989

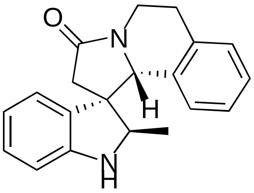

23

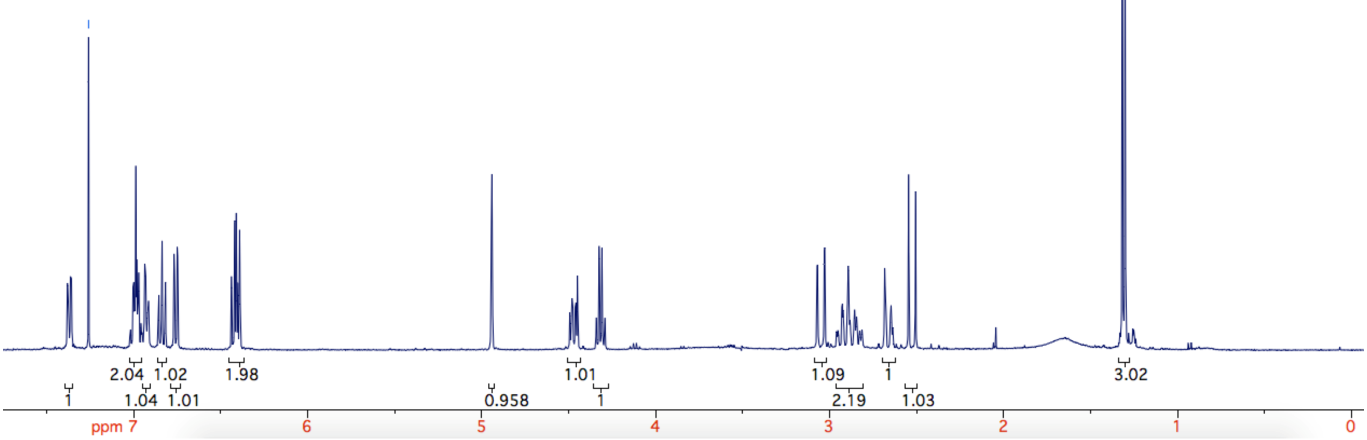
7.260
2.04
1.02
1.98
1.04
1.01
1
0.958
1.01
1
1.09
1
2.19
1.03
3.02
ppm 7
6
5
4
3
2
1
0

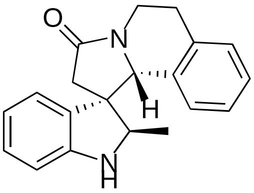

23

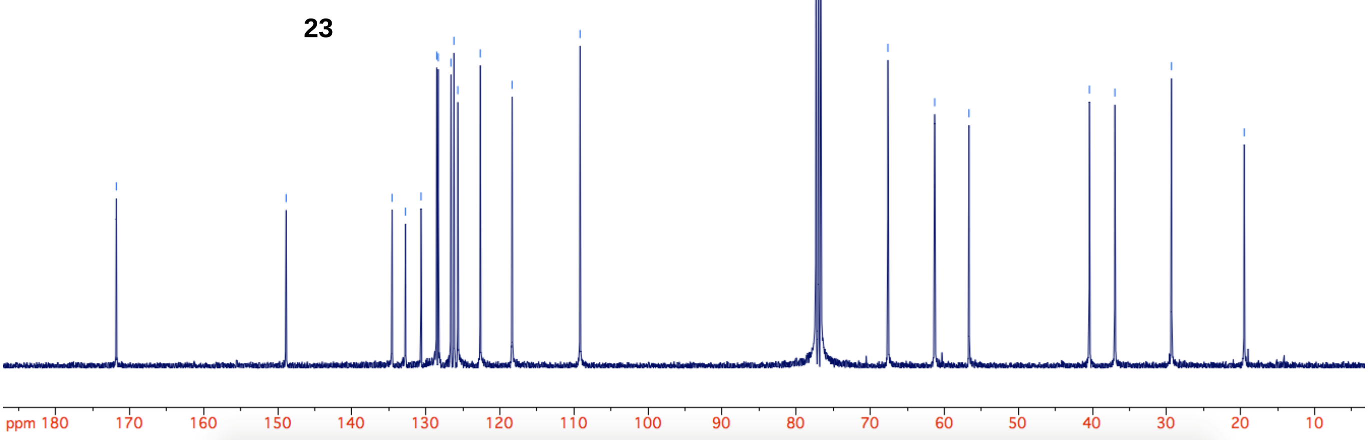
171.785
148.864
134.553
132.751
130.651
128.524
128.324
126.609
126.207
125.681
122.652
118.353
109.178
77.320
77.000
76.682
67.616
61.300
56.669
40.413
36.972
29.333
19.510
ppm 180
170
160
150
140
130
120
110
100
90
80
70
60
50
40
30
20
10

12) 3-Dimensional shape analysis of spirocyclic products and FDA approved drugs

3D structures were generated Pipeline Pilot 8.5.0.200, 2011, Accelrys Software Inc. Prior to conformer generation a wash step was performed, which involved ionising the molecule at pH 7.4, adding explicit hydrogens and outputting the canonical tautomer. Conformers were generated using the BEST method in Catalyst with a maximum relative energy threshold of 20 kcal mol^{-1}. These conformations were then minimised using 1000 steps of Steepest Descent with a RMS gradient tolerance of 3 and 200 steps of Conjugate Gradient with an RMS gradient tolerance of 0.1. Minimisation was performed using the CHARMm forcefield with Momany-Rone partial charge estimation and a Generalised Born implicit solvent model. The lowest energy conformer was selected. The generated conformations were used to generate the three Principal Moments of Inertia (I1, I2 and I3) which were then normalised by dividing the two lower values by the largest (I1/I3 and I2/I3) using Pipeline Pilot built-in components.

Principal moments of inertia (PMI) about the principal axes of a molecule were calculated according to the following rules:

1. The moments of inertia are computed for a series of straight lines through the centre of mass.

2. Distances are established along each line proportional to the reciprocal of the square root of I on either side of the centre of mass. The locus of these distances forms an ellipsoidal surface. The principal moments are associated with the principal axes of the ellipsoid.

3. If all three moments are equal, the molecule is considered to be a symmetrical top. If no moments are equal, the molecule is considered to be an unsymmetrical top.

The PMI plots were then generated with this data in Excel 2013.

PMI plot of FDA approved drugs

The PMI plot of 1439 FDA approved small molecule drugs is shown in Figure 1. The compound data was taken from the DrugBank database.[35] 23% of the compounds are found within the (NPR1 + NPR2) > 1.2 region *i.e.* in the blue triangle (Figure 1).

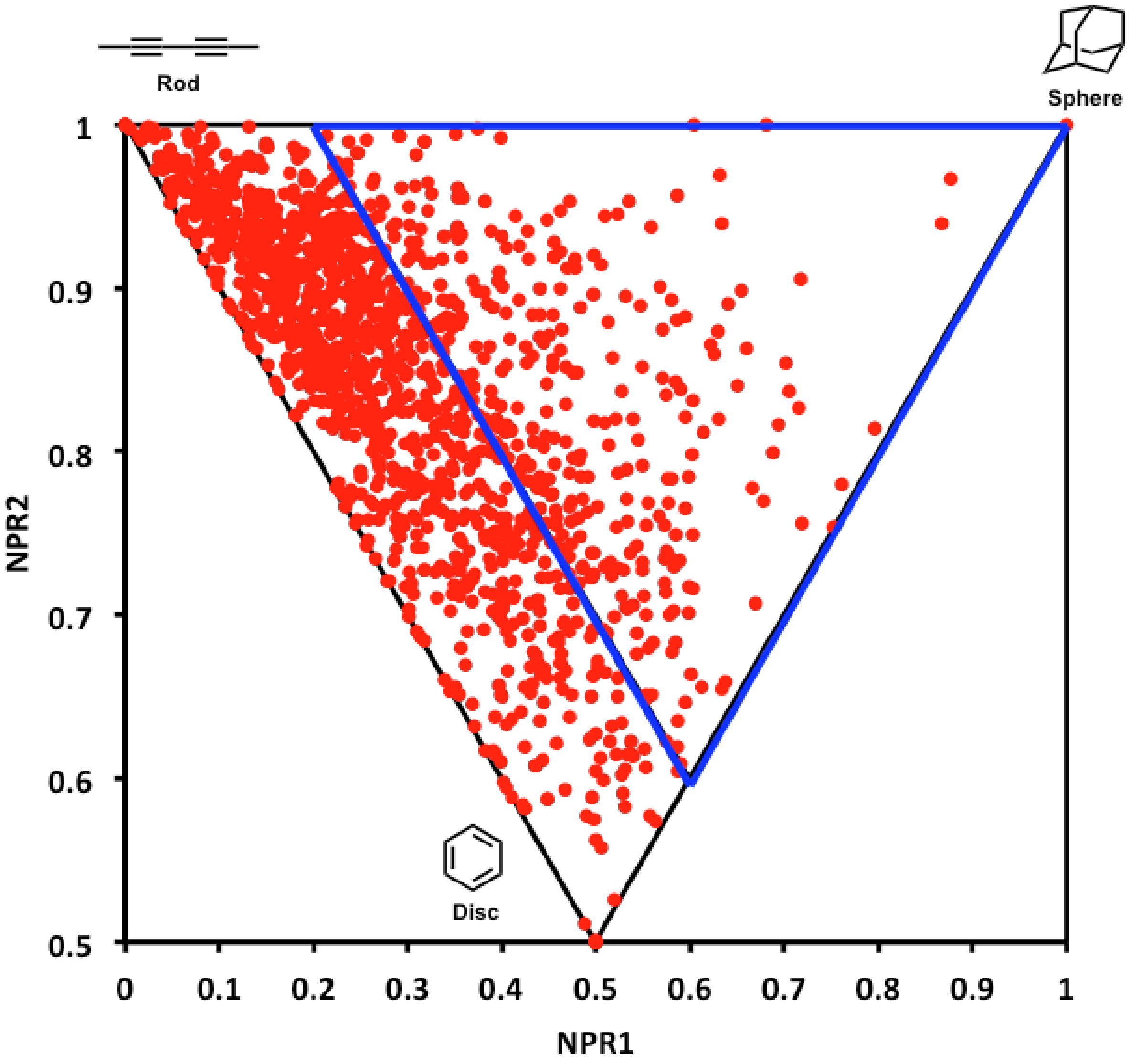

Figure 1 – PMI plot of 1439 FDA approved small molecule drugs

(13) References

(1) Liedtke, A. J.; Kim, K.; Stec, D. F.; Sulikowski, G. A.; Marnett, L. J. *Tetrahedron* **2012**, *68*, 10049.
(2) Preciado, S.; Mendive-Tapia, L.; Albericio, F.; Lavilla, R. *J. Org. Chem.* **2013**, *78*, 8129.
(3) Shi, J.; Manolikakes, G.; Yeh, C.-H.; Guerrero, C. A.; Shenvi, R. A.; Shigehisa, H.; Baran, P. S. *J. Am. Chem. Soc.* **2011**, *133*, 8014.
(4) Okano, K.; Tokuyama, H.; Fukuyama, T. *J. Am. Chem. Soc.* **2006**, *128*, 7136.
(5) Unsworth, W. P.; Kitsiou, C.; Taylor, R. J. K. *Org. Lett.* **2012**, *15*, 258.
(6) Piazzi, L.; Belluti, F.; Bisi, A.; Gobbi, S.; Rizzo, S.; Bartolini, M.; Andrisano, V.; Recanatini, M.; Rampa, A. *Bioorg. Med. Chem.* **2007**, *15*, 575.
(7) Shanker, K.; Agarwal, V. K.; Selveraj, R. J.; Parmar, S. S. *J. Med. Chem.* **1969**, *12*, 324.
(8) Haag, B. A.; Zhang, Z.-G.; Li, J.-S.; Knochel, P. *Angew. Chem. Int. Ed.* **2010**, *49*, 9513.
(9) Mérour, J. Y.; J. Y. Coadou; Tatibouët, F. *Synthesis* **1982**, *12*, 1053.
(10) Lanzilotti, A. E.; Littell, R.; Fanshawe, W. J.; McKenzie, T. C.; Lovell, F. M. *J. Org. Chem.* **1979**, *44*, 4809.
(11) Menciu, C.; Duflos, M.; Fouchard, F.; Le Baut, G.; Emig, P.; Achterrath, U.; Szelenyi, I.; Nickel, B.; Schmidt, J.; Kutscher, B.; Günther, E. *J. Med. Chem.* **1999**, *42*, 638.
(12) Walton, E.; Stammer, C. H.; Nutt, R. F.; Jenkins, S. R.; Holly, F. W. *J. Med. Chem.* **1965**, *8*, 204.
(13) Stevens, F. J.; Su, H. C.-F. *J. Org. Chem.* **1962**, *27*, 500.
(14) Fanshawe, W. J.; Mckenzie, T. C.; Crawley, L. S. *US4302589 (A)* **1981**.
(15) Dillard, R. D.; Bach, N. J.; Draheim, S. E.; Berry, D. R.; Carlson, D. G.; Chirgadze, N. Y.; Clawson, D. K.; Hartley, L. W.; Johnson, L. M.; Jones, N. D.; McKinney, E. R.; Mihelich, E. D.; Olkowski, J. L.; Schevitz, R. W.; Smith, A. C.; Snyder, D. W.; Sommers, C. D.; Wery, J.-P. *J. Med. Chem.* **1996**, *39*, 5119.
(16) McKew, J. C.; Foley, M. A.; Thakker, P.; Behnke, M. L.; Lovering, F. E.; Sum, F.-W.; Tam, S.; Wu, K.; Shen, M. W. H.; Zhang, W.; Gonzalez, M.; Liu, S.; Mahadevan, A.; Sard, H.; Khor, S. P.; Clark, J. D. *J. Med. Chem.* **2006**, *49*, 135.
(17) Elkady, M.; Nieß, R.; Schaible, A. M.; Bauer, J.; Luderer, S.; Ambrosi, G.; Werz, O.; Laufer, S. A. *J. Med. Chem.* **2012**, *55*, 8958.
(18) Company, A. C. 1981; Vol. US4302589 A1 1981, p 7.
(19) Itadani, S.; Yashiro, K.; Aratani, Y.; Sekiguchi, T.; Kinoshita, A.; Moriguchi, H.; Ohta, N.; Takahashi, S.; Ishida, A.; Tajima, Y.; Hisaichi, K.; Ima, M.; Ueda, J.; Egashira, H.; Sekioka, T.; Kadode, M.; Yonetomi, Y.; Nakao, T.; Inoue, A.; Nomura, H.; Kitamine, T.; Fujita, M.; Nabe, T.; Yamaura, Y.; Matsumura, N.; Imagawa, A.; Nakayama, Y.; Takeuchi, J.; Ohmoto, K. *J. Med. Chem.* **2015**, *58*, 6093.
(20) Stevens, F. J.; Higginbotham, D. H. *J. Am. Chem. Soc.* **1954**, *76*, 2206.
(21) Jirousek, M. R.; Paal, M.; Ruhter, G.; Schotten, T.; Takeuchi, K.; Stenzel, W. *EP1266897 (A2)* **2002**.
(22) Patil, N. T.; Konala, A. *Eur. J. Org. Chem.* **2010**, *2010*, 6831.
(23) Jeanty, M.; Blu, J.; Suzenet, F.; Guillaumet, G. *Org. Lett.* **2009**, *11*, 5142.
(24) Chen, Z.; Lu, B.; Ding, Z.; Gao, K.; Yoshikai, N. *Org. Lett.* **2013**, *15*, 1966.
(25) Holmes, D. L.; Lightner, D. A. *Tetrahedron* **1995**, *51*, 1607.
(26) Kenny, M.; Christensen, J.; Coles, S. J.; Franckevičius, V. *Org. Lett.* **2015**, *17*, 3926.
(27) Paine, J. B.; Dolphin, D. *J. Org. Chem.* **1985**, *50*, 5598.
(28) Wendlandt, A. E.; Stahl, S. S. *J. Am. Chem. Soc.* **2013**, *136*, 506.
(29) Tinnis, F.; Lundberg, H.; Adolfsson, H. *Adv. Synth. Catal.* **2012**, *354*, 2531.
(30) Saavedra, J. Z.; Resendez, A.; Rovira, A.; Eagon, S.; Haddenham, D.; Singaram, B. *J. Org. Chem.* **2012**, *77*, 221.

(31) Huff, J. R.; Baldwin, J. J.; DeSolms, S. J.; Guare, J. P.; Hunt, C. A.; Randall, W. C.; Sanders, W. S.; Smith, S. J.; Vacca, J. P.; Zrada, M. M. *J. Med. Chem.* **1988**, *31*, 641.
(32) Rostom, S. A. F.; Farghaly, A. M.; Soliman, F. S. G.; El-Semary, M. M.; Elz, S.; Lehmann, J. *Arch. Pharm.* **2001**, *334*, 241.
(33) Khorana, N.; Smith, C.; Herrick-Davis, K.; Purohit, A.; Teitler, M.; Grella, B.; Dukat, M.; Glennon, R. A. *J. Med. Chem.* **2003**, *46*, 3930.
(34) de Carné-Carnavalet, B.; Krieger, J.-P.; Folléas, B.; Brayer, J.-L.; Demoute, J.-P.; Meyer, C.; Cossy, J. *Eur. J. Org. Chem.* **2015**, *2015*, 1273.
(35) Wishart, D. S.; Knox, C.; Guo, A. C.; Shrivastava, S.; Hassanali, M.; Stothard, P.; Chang, Z.; Woolsey, J. *Nucleic Acids Res.* **2006**, *34*, 668.
